# Placebo-Induced Somatic Sensations: A Multi-Modal Study of Three Different Placebo Interventions

**DOI:** 10.1371/journal.pone.0124808

**Published:** 2015-04-22

**Authors:** Florian Beissner, Franziska Brünner, Maria Fink, Karin Meissner, Ted J. Kaptchuk, Vitaly Napadow

**Affiliations:** 1 Somatosensory and Autonomic Therapy Research, Institute of Neuroradiology, Hannover Medical School, 30625, Hannover, Germany; 2 Athinoula A. Martinos Center for Biomedical Imaging, Department of Radiology, Massachusetts General Hospital, Charlestown, MA, 02129, United States of America; 3 Pain & Autonomics – Integrative Research (PAIR), Department of Psychiatry and Psychotherapy, Jena University Hospital, 07743, Jena, Germany; 4 Institute of Medical Psychology, Ludwig-Maximilians-University, 80336, Munich, Germany; 5 Program in Placebo Studies, Beth Israel Deaconess Medical Center, Harvard Medical School, Boston, MA, 02215, United States of America; 6 Department of Biomedical Engineering, Kyunghee University, Yongin, Korea; University Medical Center Goettingen, GERMANY

## Abstract

Somatic sensations induced by placebos are a frequent phenomenon whose etiology and clinical relevance remains unknown. In this study, we have evaluated the quantitative, qualitative, spatial, and temporal characteristics of placebo-induced somatic sensations in response to three different placebo interventions: (1) placebo irritant solution, (2) placebo laser stimulation, and (3) imagined laser stimulation. The quality and intensity of evoked sensations were assessed using the McGill pain questionnaire and visual analogue scales (VAS), while subjects’ sensation drawings processed by a geographic information system (GIS) were used to measure their spatial characteristics. We found that all three interventions are capable of producing robust sensations most frequently described as “tingling” and “warm” that can reach consider-able spatial extent (≤ 205mm^²^) and intensity (≤ 80/100 VAS). Sensations from placebo stimulation were often referred to areas remote from the stimulation site and exhibit considerable similarity with referred pain. Interestingly, there was considerable similarity of qualitative features as well as spatial patterns across subjects and placebos. However, placebo laser stimulation elicited significantly stronger and more widespread sensations than placebo irritant solution. Finally, novelty seeking, a character trait assessed by the Temperament and Character Inventory and associated with basal dopaminergic activity, was less pronounced in subjects susceptible to report placebo-induced sensations. Our study has shown that placebo-induced sensations are frequent and can reach considerable intensity and extent. As multiple somatosensory subsystems are involved despite the lack of peripheral stimulus, we propose a central etiology for this phenomenon.

## Introduction

Placebo effects have established their veracity both in clinical trials and experimental studies [1]. Recent studies have also delineated a neurobiological substrate for placebo effects [2,3]. Moreover, several psychological constructs have been posited to explain placebo effects, such as verbal expectations, classical conditioning, anxiety reduction and social observation [4,5]. There is also a long history of experiments providing evidence that different placebo treatments such as dummy devices, sham surgery and placebo pills produce different clinical outcomes [6–13].

While previous studies have predominantly seen placebos as a form of therapeutic intervention (cf. Shapiro’s definition from 1968 [14]), here we study sham procedures similar to those commonly used in placebo studies (lasers, topical medication) without explicitly referring to any therapeutic potential. Using this broader definition of placebos, we study a little-researched phenomenon that may complement current views, namely somatic sensations induced by placebo interventions. Although they are frequently reported in the literature, little is known about their etiology.

Interventions reported to elicit sensations include placebo injections [15], placebo transcutaneous electrical nerve stimulation [16], placebo acupuncture [17,18], and placebo laser stimulation [19,20]. While the first two studies only reported that sensations were elicited, the latter four explored them in some detail, focusing on incidence [17,18], descriptor frequency, and intensity [19,20]. To our knowledge, no study has explored spatial and temporal characteristics of placebo-induced sensations. Furthermore, all previous studies investigating placebo-induced sensations compared active exposure (e.g. acupuncture) versus placebo exposure as a primary question, which may have led to specific expectations in subjects and, hence, influenced the outcome. Therefore, we designed our study to avoid any reference to placebos or any therapeutic interventions.

We conducted three experiments. In Experiments 1 and 2, placebos with different tactile components were tested for the somatic sensations they evoke. We hypothesized that placebo-induced sensations are a phenomenon that can be triggered by a variety of different placebos, and that a common etiology underlies these sensation reports. Therefore, we expected quantitative (e.g. intensity and extent) but not qualitative differences (e.g. descriptor profiles) between the different tested placebos. In Experiment 3, we aimed at minimizing possible influences from the experimenter or laboratory setting by replacing “actual” placebo stimulation with laser stimulation that was only imagined by subjects at home.

Placebo-induced sensations were studied using the McGill questionnaire [21] and visual analogue scales. Spatial characteristics were assessed by analyzing subjects’ sensation drawings on body outlines with a geographic information system (GIS) [22]. Since a number of previous studies have linked placebo analgesia to basal dopaminergic regulation [23], we assessed novelty seeking as a character trait linked to dopamine [24] and investigated possible correlations between novelty seeking and subjects’ susceptibility to develop placebo-induced sensations.

Finally, we compared the spatial patterns of placebo-induced sensations to that of referred pain and to sensation patterns from our previous study using true low-level laser stimulation [22].

## Materials & Methods

### Experimental design

We performed three experiments to quantify the susceptibility of healthy subjects to placebo-induced sensations. Experiments 1 and 2 took place under laboratory conditions (room temperature of ~20°C controlled by AC, no disturbances by noise) and were identical in design except for the stimulus modality. All measurements took place between 12 a.m. and 7 p.m. Experiment 3 was conducted as a questionnaire study to eliminate any influence a laboratory setting might have on placebo-induced sensations. Experiment 1 used a placebo with a small but noticeable tactile component (water drenched cotton bud on the skin), Experiment 2 had no tactile component (inactive laser held over the skin). Experiment 3 aimed at minimizing possible influences from the experimenter or laboratory setting by replacing “actual” placebo stimulation with laser stimulation that was imagined by subjects at home.

### Subjects

All subjects taking part in the study were healthy on the day of the measurement and did not have scars or injuries at any of the stimulation sites. None of them had any history of neurological disease or took any kind of medication on a regular basis other than oral contraceptives. The intake of analgesics as rescue medication was prohibited during the five days before the measurement. All subjects were right-handed (self-report). For Experiments 1 and 2, two groups of 30 subjects each were included in the study. Subjects of Experiment 1 (12 male) had a mean age (± S.D.) of 23.6 ± 3.5 years, while those of Experiment 2 (8 male) had a mean age of 24.5 ± 7.4 years. For Experiment 3, a questionnaire was sent out by mail to 104 healthy subjects that had previously been recruited and screened for taking part in an unrelated study. 56 subjects (21 male) returned a complete and valid questionnaire. All subjects were at least 18 years old, which is the legal age in Germany. The mean age was 25.3 ± 6.6 years. All three subject cohorts were independent and there were no differences in gender balance between them (Fisher’s exact test: p = 0.4118 for exp. 1 vs. 2, p = 0.8207 for exp. 1 vs. 3, and p = 0.3481 for exp. 2 vs. 3). The study was conducted according to the Declaration of Helsinki and all participants gave written informed consent following the guidelines of the ethics committee of the University Hospital Jena, which had approved the study.

### Stimulation loci and duration

**Fig 1:**
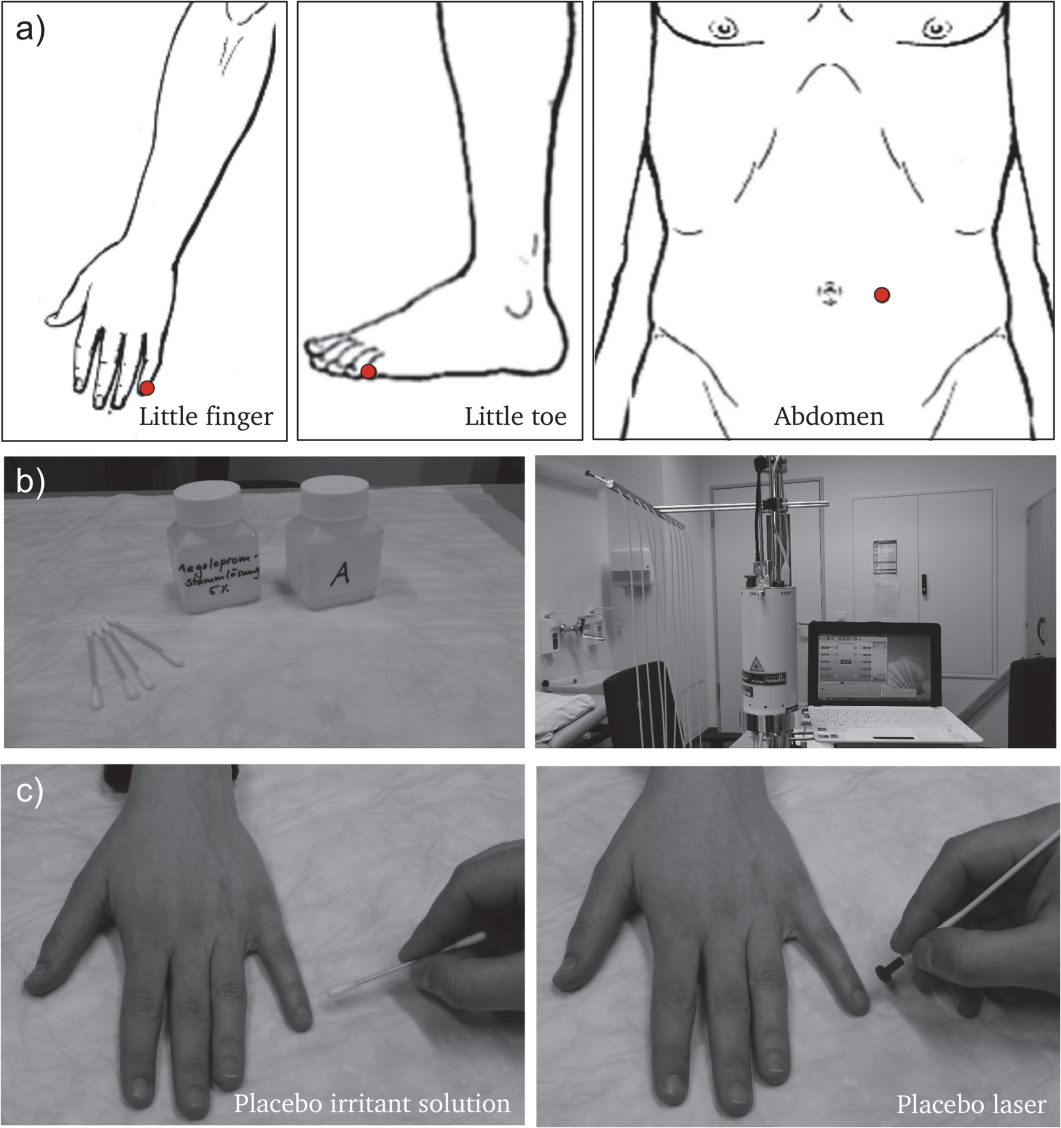
Stimulation loci and placebo interventions. a) Locations stimulated in the experiment. b) Placebos used in the study. Left side: Placebo irritant solution (water), right side: Placebo laser (switched off). c) Application of the two placebos to the little finger of the hand.

Placebo stimulation for Experiments 1 and 2 took place at three different loci (**Fig 1**), each of them being stimulated with both placebos. The first location was on the dorsal aspect of the little finger, approximately 1 mm from the proximal ulnar corner of the nail. The second was on the dorsal aspect of the little toe, approximately 1 mm from the proximal lateral corner of the nail. The third was on the abdomen, approximately 3 cm lateral to the navel. Each stimulation run had a baseline of 30 seconds followed by three minutes during which subjects were asked to focus on their sensations. We chose this rather long period since we wanted to leave enough time for possible processes like temporal summation to fully develop. Points were stimulated in pseudo-randomized order that was repeated once (i.e. six runs in total) and only the left side of the body was stimulated. All measurements were carried out by one of three different female experimenters.

### Body Outline

To assess and compare bodily sensations experienced by the subjects, we used a semi-structured approach based on subjects’ drawings over a pictorial body outline. Body outlines were adapted from the illustrations in Head (1893) [25] (**S1 Fig**) and were successfully used in our previous study [22]. A new paper copy of these body outlines was handed out to the subjects after each stimulation run (subjects’ blindfold was removed for this). The instructions given to the subjects in Experiments 1 and 2 were (translated to English): “Draw a point for every point-like sensation, a line for every line-like sensation and shade in a region for every area-like sensation.” Please note that here and in the following, “point-like”, “line-like” and “area-like” refer to the spatial and not temporal characteristics of the sensations. In Experiment 3, the same body outlines were used, albeit with different instructions (see below).

### Sensory descriptors and intensity rating

For a comprehensive list of sensory descriptors, the German version of the McGill pain questionnaire [21,26] was used. The order of its 77 descriptors (sensory, affective, and evaluative) was randomized for each subject. Due to our experience from an earlier study [22], we added the descriptor “warm”. In Experiments 1 and 2, subjects were asked to choose any number of descriptors that matched their sensations and to rate the intensity of each chosen descriptor on a 100-point visual analogue scale ranging from “not felt” (0) to “maximal tolerable intensity” (100). They were also free to add descriptors of their own. The VAS score of the descriptor with the highest rating was used to assess the overall intensity of the sensation. The raw data for this and all other collected data can be found in the supporting information (S1 Raw Data).

### Experiment 1

#### Placebo

In the first experiment, a placebo irritant solution, which consisted of ordinary tap water at room temperature, was used and applied to the skin with a cotton bud. We invented the name “Aegoloprom” as a neutral term after preliminary experiments had shown that a placebo solution named “Capsitol” mainly induced local burning sensations as some subjects suspected it to contain capsaicin.

#### Procedure

To reduce anxiety, subjects were told that the active ingredient of the solution was a food additive and that stimulation with a weak concentration of this substance should not induce significant side effects. To increase credibility, we suggested that the following side effects may occur when the solution was applied over a longer time period: allergic reactions, transient sensations and local reddening of the skin. Subjects were instructed not to bring any of the substance into their eyes as this might cause local irritation. The cotton bud was drenched in water and subsequently applied to the skin for ten seconds. Care was taken to apply only a minimal amount of water that would not run down the body. Subjects were blindfolded during each of the six experimental runs.

The following instructions were read aloud to the subjects (comments in italic are added for clarity and were not part of the text): “This is a perception experiment. We are interested in the sensations that can be elicited by stimulation with a weak solution *(Experiment 1 only)* / a weak laser *(Experiment 2 only)*. The name of the solution is Aegoloprom *(sentence deleted in Experiment 2)*. I will reveal its mechanism of action to you at the end of the experiment. Please monitor sensations in your body during the whole measurement, especially at the points stimulated. At the end of the experiment, report all sensations that you attribute to the stimulation. Draw these sensations on the body outlines. Draw a point for every point-like sensation, a line for every a line-like sensation and hachures for every area-like sensation. Subsequently, chose any number of words from the list that best describe your sensation. If none of the words describe your sensation adequately, you may also use words of your own. Finally, mark the intensity of your sensation on this visual analogue scale. The left end of the scale stands for no sensation, the right end for a strong sensation on the verge of intolerable. Some people have a sensation, when they are stimulated, others do not. One is not better or worse than the other.”

After lying down on the stretcher, subjects were further instructed: “For the next few moments, please do nothing and concentrate on what you feel, e.g. how it feels like to lie on this stretcher. Any sensations you may just have had are not those that are important when I will later ask you what you felt.”

Right before the experiment started, subjects were instructed to report the onset of every new sensation they may have to the experimenter. The onset of the first reported sensation was measured with a stopwatch. After subjects had completed the experiment, they were asked what they thought/believed the purpose of the study was.

### Experiment 2

#### Placebo

In the second experiment on an independent cohort, a low-level (20mW) laser device, emitting red light (Laserneedle micro, Laserneedle EG GmbH, Wehrden, Germany) was used as the placebo. During the stimulation phase, the optode at the end of the fiber optical cable of the laser was held over the point that was stimulated without touching the skin or skin hair. The laser, however, was switched off during the whole procedure. Subjects were blindfolded during each of the six experimental runs.

#### Procedure

Before the experiment started, the laser was switched on and the red laser light was briefly shone on the subjects’ right hand to reduce their anxiety. The instructions given to the subject were the same as in Experiment 1. The side effect “allergic reaction” was replaced by “possible damage to the eyes, when directly exposed to the laser light”. Again, the onset of the first reported sensation was measured with a stopwatch and after completing the experiment, subjects were asked for what they thought was the purpose of the study.

### Experiment 3

#### Imagined stimulation

In the third experiment run on an independent cohort of subjects, no actual placebo was used. Instead, we asked subjects to imagine being stimulated with a red laser and to draw the sensations that may result from this stimulation. As we aimed at eliminating any laboratory context and influence of the experimenter, we used a questionnaire that was sent out to subjects by mail and filled out at home.

#### Procedure

The questionnaire was based on the same body outlines used in Experiments 1 and 2 and contained 8 pages (only front and back views of the body). On each page, the stimulation locus to be imagined was marked with a red dot. Stimulation loci were taken from a previous study [22], where subjects had also reported sensations during placebo stimulation that were predominantly line-like in shape. We only report results from the little finger and toe here, as those locations were also stimulated in Experiments 1 and 2.

The questionnaire came with the following instructions: “On the following pages you will find body outlines. Each outline contains a red dot (**S2 Fig**). Please imagine your own body being stimulated with weak red laser light at the marked position. The laser light is shone directly onto your skin using a fiber optical cable. The laser stimulation shall last for some minutes and elicits a line-like sensation. How you imagine this sensation is the object of this study.”

Subjects then received instructions on how to draw their imagined sensations: “On every page you will find a green arrow next to the body outline. This arrow represents the line-like sensation. Please draw a line into the body outline that has approximately the same length and that best describes your imagined sensations. The line does not have to be straight and may follow any course but is not allowed to intersect with itself. Please make sure that the line does not leave the borders of the body outline.”

On its last page, the questionnaire contained six items in the form of self-referential statements that subjects could rate on a five point scale (from “strongly disagree” to “strongly agree”). The central statement was “I had actual sensations at the marked locations.” The other five items were added to distract the subject from the true object of the study. Subjects that answered with “agree” or “strongly agree” were then asked to describe their sensations using their own words. These subjects are called “susceptible” in the rest of the manuscript, all others “non-susceptible”.

#### Assessment of character traits

To assess whether the susceptibility for placebo-induced sensation under imagined stimulation was associated with certain character traits, all subjects that took part in Experiment 3 also filled out the Temperament and Character Inventory, TCI [27] in its German version 9 (240 items, true/false scoring). The TCI operates with four temperaments (novelty seeking, harm avoidance, reward dependence, persistence) and three character traits (self-directedness, cooperativeness, self-transcendence) as personality traits. It is based on a psychobiological model that attributes high scores in novelty seeking, harm avoidance, and reward dependence, to low basal dopaminergic activity, high serotonergic activity, and low basal noradrenergic activity, respectively [24]. Validity of these attributions has been shown using positron emission tomography [28] as well genetic polymorphisms [29].

### Data analysis

#### Frequency of placebo-induced sensations

To assess a possible interrelation between the placebo type and the overall number of trials that elicited sensations, a two-tailed Fisher’s exact test was used. Possible associations between the different stimulation sites and sensation frequencies were also assessed using a two-tailed Chi-squared test for the pooled data of Experiments 1 and 2. Here and in all following tests (unless stated otherwise), a p-value below 0.05 was considered significant.

#### Intensity of placebo-induced sensations

Two-sided two-sample t-tests were calculated to compare placebo irritant solution sensation intensity vs. placebo laser sensation intensity for all three stimulation sites.

#### Geometric features of placebo-induced sensations

Following a previously validated approach [22] sensation drawings were analyzed using a geographic information system (GIS, [30]). To compare the localization and extent of the sensations drawn by subjects from different study groups, the body outline was digitized and scaled to a body height of 175cm to use within the GIS body-map template in ArcGIS 10.0 (esri GmbH, Kranzberg, Germany). All body sketches were scanned, transferred into the GIS database, and geo-referenced to the body-map template. The database was then populated with the subjects’ mapped sensations in point, polyline, and polygon format by manually digitizing them on-screen from the scanned sketches.

For sensations of line-like and two-dimensional shape, the line length and polygon area were calculated. Possible differences in the overall length or area between placebos were assessed using two-sided two-sample t-tests of the data pooled from all stimulation sites and repetitions.

For illustration purposes, lines were converted to 1.5 cm wide swaths of polygons by a buffering algorithm to overlay multiple line-like and/or two-dimensional sensations in a single plot. Finally, all sensations were overlaid and thresholded to display only areas that had been drawn by at least two different subjects.

#### Comparison of sensation patterns evoked by placebo laser versus referred pain

To explore possible parallels between the observed referral of placebo-induced sensations and the phenomenon of referred pain, we compared the spatial patterns from Experiments 2 and 3 to that of referred pain from the literature. As a reference, we chose[31]. This group used hypertonic saline injections into paravertebral muscles at different segments to elicit referred pain. As we had observed similar patterns in a previous study using low-level laser stimulation [22], we also included these patterns in the comparison.

#### Verbal descriptors of placebo-induced sensations

The average number of descriptors in Experiment 1 and 2 that were chosen by susceptible subjects to rate their sensations was calculated by averaging over all stimulation sites and repetitions. Wilcoxon’s signed-rank test was used to test for differences between the experiments as well as between different points. For the latter, data from Experiments 1 and 2 were pooled. For each descriptor, we calculated the percentage of subjects that chose it. Descriptors that were chosen several times by the same subject were only counted once. For the most common descriptors, mean VAS scores were calculated. For the same descriptors Mann-Whitney tests were used to detect possible differences between placebo types. P-values were Bonferroni corrected for multiple comparisons.

#### Onset of placebo-induced sensations

The onset of a sensation was defined as the time between the beginning of stimulation (i.e. the moment, when the cotton bud was placed on the skin or subjects were told that the laser was switched on) and the moment, when the first sensation was verbally reported by the subject. It was measured by the experimenter with a stop watch. To increase power, data were pooled for all stimulation sites and repetitions. Differences between the two placebos were assessed using a Kolmogorov-Smirnov test, as the data were not normally distributed (Anderson-Darling test: p<0.05).

#### Influence of character traits

T-values were calculated from the raw TCI results based on the instructions in the questionnaire’s manual. A one-sided two-sample t-test was used to test the hypothesis of lower novelty seeking values in subjects susceptible to placebo-induced sensations. Additional two-sided two-sample t-tests were calculated for the other three temperaments “harm avoidance”, “reward dependence”, and “persistence”. A multiple comparisons corrected p-value of 0.0167 or lower (0.05 corrected for 3 comparisons) was considered significant.

## Results

### Susceptibility to placebo-induced sensation and drop-outs

Subjects that reported any sensations during one of the experimental runs after filling out the questionnaire are henceforth called susceptible, all others non-susceptible.

The percentage of susceptible subjects was 90.0%, 86.7% and 27.6% for Experiments 1, 2 and 3, respectively. One subject dropped out during a stimulation run in Experiment 2, because the sensation became unbearably strong. She later resumed the experiment.

### Frequency of placebo-induced sensations

The frequency of sensations for the different placebos and stimulation sites were as follows. Subjects reported sensations during 124 of the 180 stimulation trials (30 subjects x 3 points x 2 runs) for placebo irritant solution and during 106 of the 180 trials for placebo laser. There was a trending significant association between placebo type and sensation frequency (p = 0.062), i.e. a trend for more frequent reported sensations for placebo solution (**S3 Fig**).

### Intensity of placebo-induced sensations

Susceptible subjects reported moderate to strong sensations under placebo stimulation. On a 0–100 VAS, maximum sensation intensities related to placebo irritant solution were as high as 82 / 87 / 75, for stimulation at the abdomen / finger / toe, respectively (maximum values reported by any subject). For placebo laser stimulation, the values were 85 / 82 / 83, respectively. The mean intensities (±S.D.) were 22.2 ± 17.2 / 26.6 ± 20.5 / 23.0 ± 17.8 for placebo irritant solution and 35.5 ± 24.1 / 40.5 ± 24.7 / 39.2 ± 24.6 for placebo laser, for the 3 loci, respectively. For all stimulation sites, placebo laser elicited significantly stronger sensations than placebo irritant solutions (abdomen: p = 0.010, finger: p = 0.0084, toe: p = 0.0018).

### Geometric features of placebo-induced sensations

**Fig 2:**
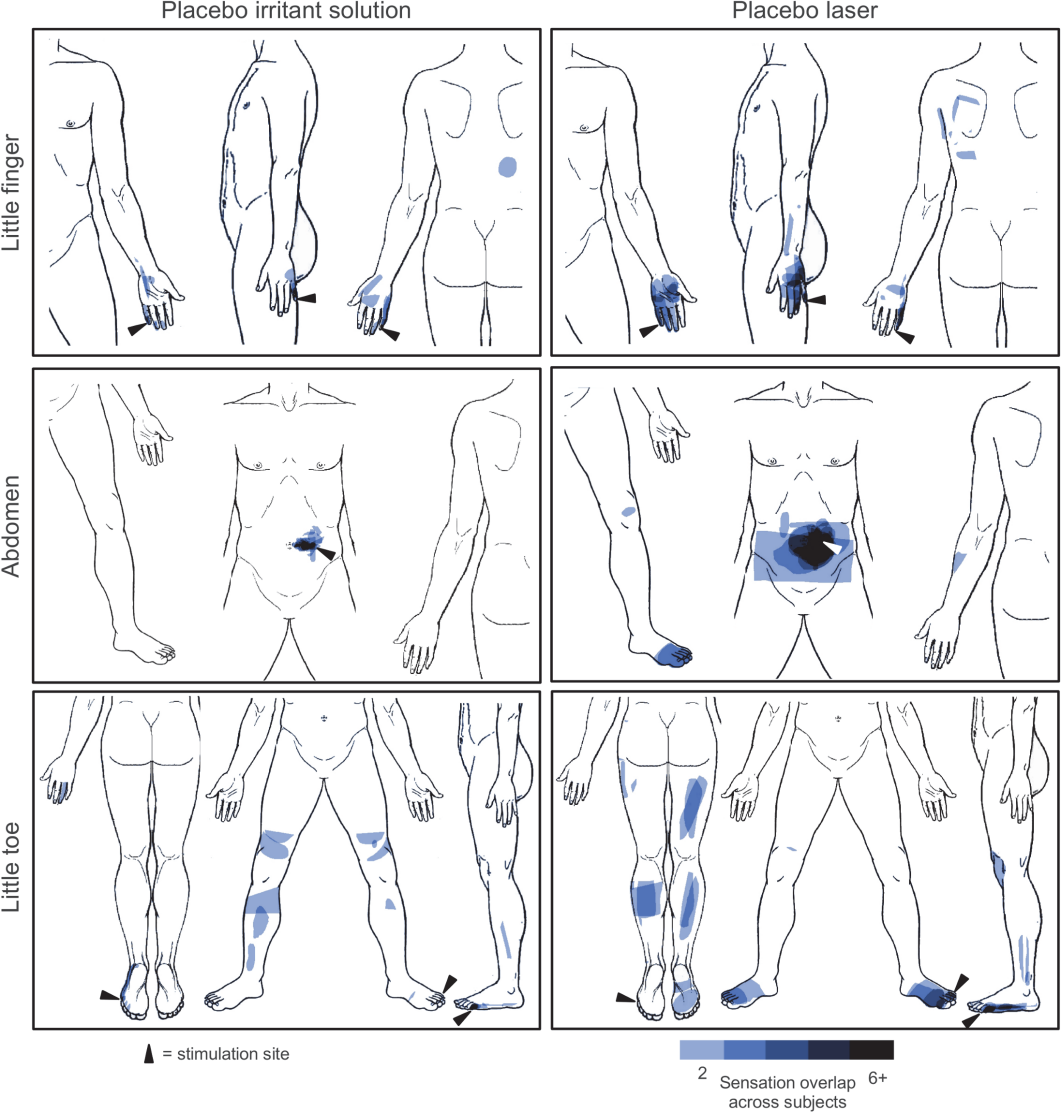
Spatial patterns of placebo-induced sensations. Sensations elicited by placebo irritant solution (left) and placebo laser (right). The stimulation site is marked with an arrowhead. Only areas that were reported by two or more subjects are shown. Note the referral of sensations to areas remote from the stimulation site as well as to the contralateral side of the body.

Sensation maps derived from subjects’ drawings in Experiments 1 and 2 showed that placebo stimulation elicited sensations that were not confined to the point of stimulation, but were often referred to remote body areas and even to the contralateral side of the body (**Fig 2**). In some subjects, sensations from stimulation of the little toe were felt up to the thigh area, whereas sensations from stimulation of the little finger reached to the upper arm and shoulder region.

Sensations were significantly more widespread under placebo laser (205.1 ± 313.4 mm²) than under placebo irritant solution (73.3 ± 178.9 mm², p = 0.010), whereas the overall length of line-like sensations only showed a tendency (30.2 ± 29.0 mm vs. 11.4 ± 16.4 mm, p = 0.070).

**Fig 3:**
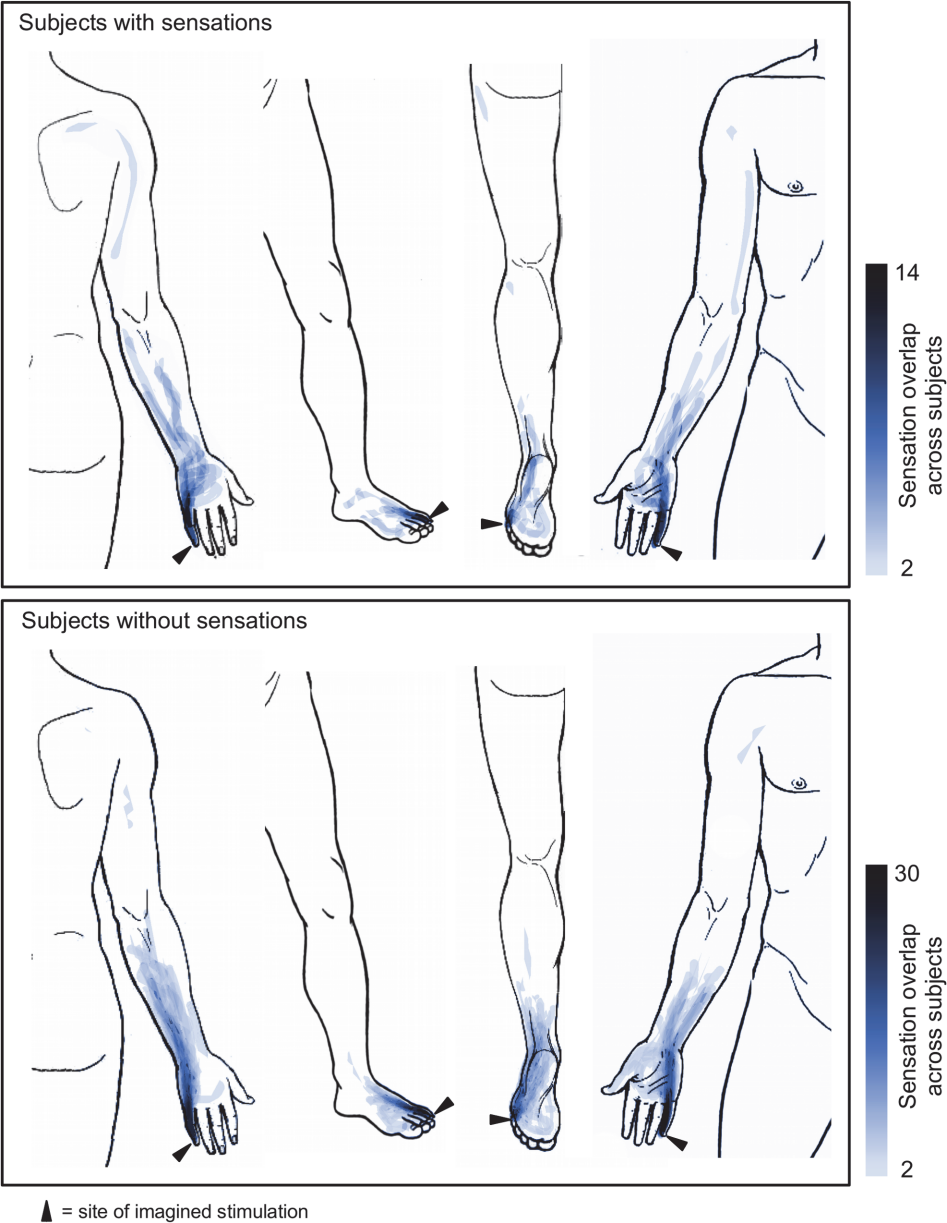
Spatial patterns of placebo-induced sensations as imagined by subjects to result from laser stimulation. The color in each point represents the number of subjects that imagined having sensations in that location. Only areas that were reported by two or more subjects are shown. The site of (imagined) stimulation is marked with an arrowhead. The upper image shows the results from subjects that actually experienced sensations during imagination, the lower of those who did not. Note the remarkable amount of overlap of sensations in both groups as well as the divergence of lines into an ulnar and a median line at the wrist.

Sensation maps derived from subject’s drawings in Experiment 3 revealed that the localization of imagined sensations was remarkably similar across subjects (**Fig 3**). This was despite the fact that subjects were free to choose any location on the body to draw their line. Visual inspection revealed that the averaged patterns were very similar for susceptible and non-susceptible subjects, although susceptible subjects seemed to show more sensation referral to proximal areas, like the upper arm and shoulder. Interestingly, imagined sensation paths diverged at the wrist in both groups and further followed the median and ulnar aspects of the inner forearm.

### Comparison of sensation patterns with laser-evoked sensations and referred pain

**Fig 4:**
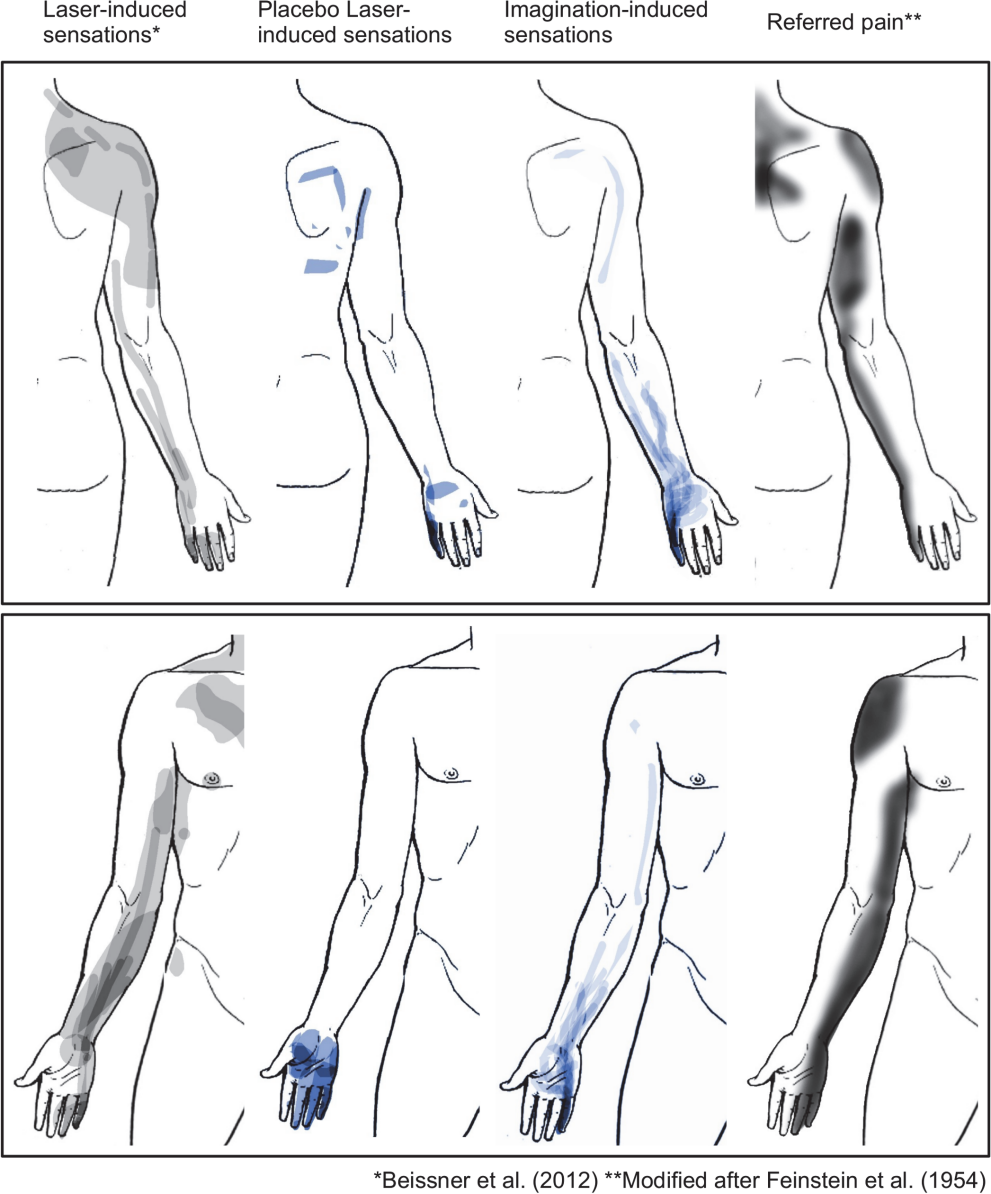
Similarities of placebo-induced sensations and referred pain. Spatial comparison showing similarities of placebo-induced and imagination-induced sensation patterns (center) with patterns from low-level laser stimulation (left) and referred pain induced by hypertonic saline injection into paravertebral muscles (right) as reported in [31].

The spatial patterns of placebo-induced sensations from Experiments 2 and 3 were remarkably similar to those of referred pain and sensations from low-level laser stimulation (**Fig 4**). In the back view of the body outline, all four patterns involved some parts of the little finger, the ulnar aspect of the forearm, the area over the triceps as well as the area of the shoulder blade. In the front view of the body outline, sensations from placebo-laser stimulation were restricted to the hand, while all other three patterns involved the little finger, the ulnar aspect of the forearm, and the inner side of the upper arm.

### Verbal descriptors for placebo-induced sensations

In Experiments 1 and 2, susceptible subjects chose an average (±S.D.) of 4.10 ± 2.40 different McGill questionnaire descriptors for sensations elicited by placebo irritant solution and 4.20 ± 3.46 descriptors for those elicited by placebo laser (no significant difference, p = 0.976).

**Fig 5:**
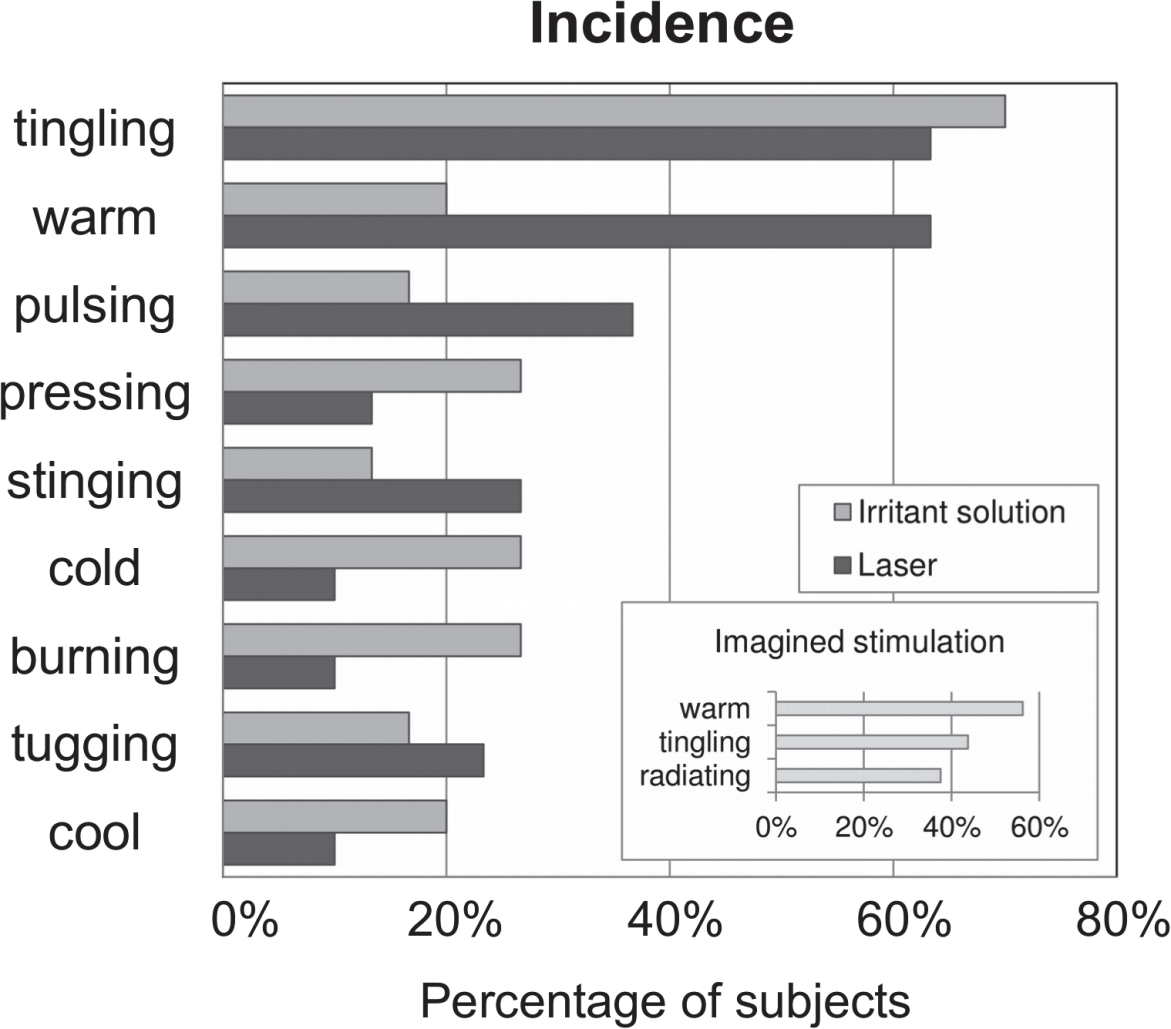
Verbal descriptors for sensations. Incidence of the nine most common verbal descriptors for placebo-induced sensations. Of all 78 possible descriptors, these were the only ones chosen by 20% or more of the subjects. Together, they accounted for 59.9% of all reported sensations, which illustrates the similarity of sensations across subjects. Results from the imagined stimulation (experiment 3) are shown in the small box. In addition to the two most common descriptors from experiments 1 and 2, subjects frequently reported radiating sensations for imagined stimulation.

Although half of the descriptors (39 of 78) were chosen at least once in either experiment, only nine descriptors were chosen by more than 20% of the subjects. Intensity information (VAS scores) for these descriptors are reported in **S4 Fig**. In terms of absolute frequencies, these descriptors together explained 59.9% of all choices – a value that was even larger (71.7%), when we accounted for repeated entries by the same subjects. These descriptors were “tingling” (70%/63.3% of subjects for irritant solution/laser), “warm” (20.0%/63.3%), “pulsing” (16.7%/36.7%), “pressing” (26.7%/13.3%), “stinging” (13.3%/26.7%), “cold” (26.7%/10.0%), “burning” (26.7%/10.0%), “tugging” (16.7%/23.3%), and “cool” (20.0%/10.0%) (**Fig 5**). The descriptor “warm” showed the only significant difference between placebos (U = 255, z = 2.88, p = 0.004).

Subjects added a total of 13 descriptors of their own. However, only one of them (“schwer”, English: “heavy”), was chosen by multiple subjects (1 for irritant solution, 3 for laser).

In Experiment 3, a total of eight descriptors were reported by susceptible subjects: “warm” (50.0%), “tingling” (43.8%), “radiating” (37.5%), “pricking”, “numbing”, “pressing”, “as if the limb had gone to sleep”, and “wanted to pull the limb away” (all 6.3%, i.e. once).

Comparison of the pooled data from Experiments 1 and 2 for different stimulation sites (**S5 Fig**) revealed that “tingling” was the only descriptor to show significantly different frequencies: It was chosen more frequently under stimulation of the finger (W = 140, n_s/r_ = 19, z = 2.81, p = 0.005) and toe (W = 165, n_s/r_ = 19, z = 3.31, p = 0.0009) than for the abdomen.

### Onset of placebo-induced sensations

**Fig 6:**
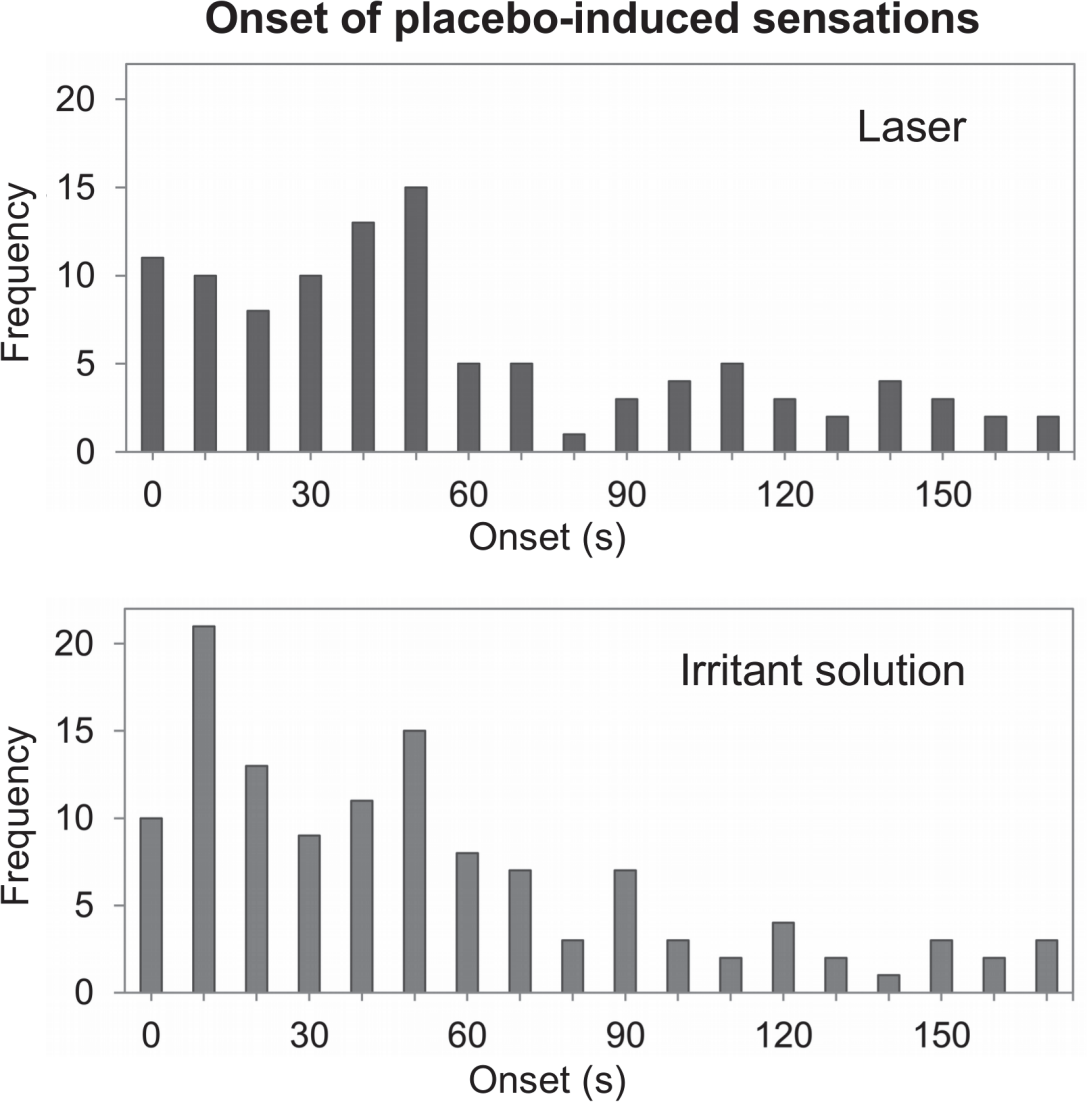
Onset of sensations. Histograms of placebo-induced sensation onset time (defined as the time between the announced placebo stimulation and the onset of the first sensation reported by the subject). Median values were 38s for placebo irritant solution and 42.5s for placebo laser. No difference between distributions was found.

Onsets ranged from instantaneous to 170s and showed a highly skewed distribution for both placebos (**Fig 6**). The median values were 38 s for placebo irritant solution and 43 s for placebo laser stimulation. There was no significant difference between the distributions (p = 0.464).

### Influence of character traits

**Fig 7:**
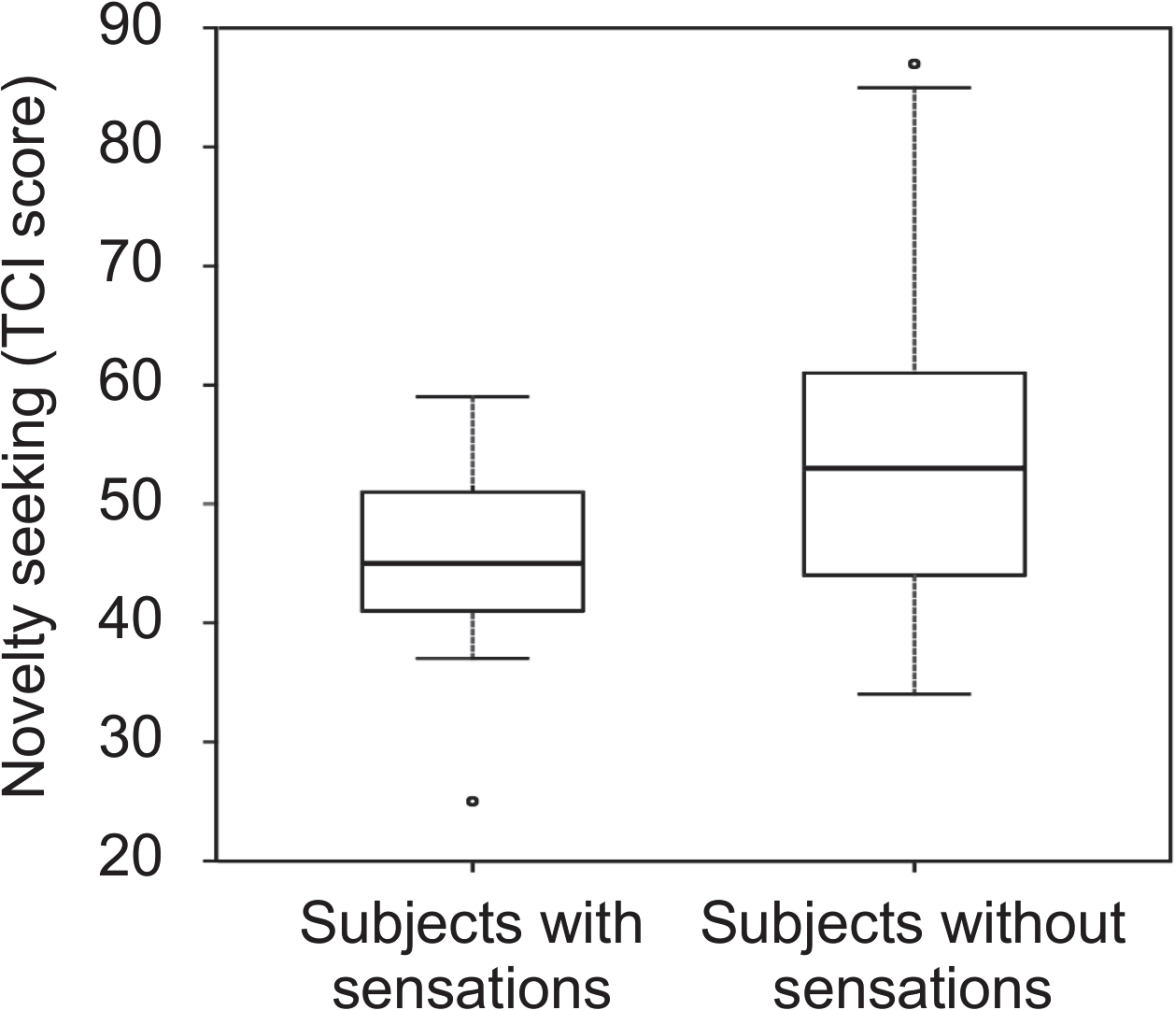
Differences in character traits of susceptible and non-susceptible subjects. Results from a comparison of character traits in subjects with and without placebo-induced sensation during imagined stimulation (experiment 3). Susceptible subjects showed a significantly lower TCI score in the “novelty seeking” category (p = 0.0071) pointing to higher basal dopaminergic activity in these subjects [24].

Subjects that were susceptible to placebo-induced sensations had significantly lower TCI scores in novelty seeking compared to non-susceptible subjects (45.2 ± 8.5 vs. 53.4 ± 11.9, p = 0.0071, see **Fig 7**) pointing to a higher basal dopaminergic activity in susceptible subjects [24]. No other temperament of the TCI showed significant differences.

### Credibility of the placebos

When asked for the purpose of the study, only a minority of subjects suspected a placebo experiment (3 in Experiment 1 and 1 in Experiment 2). The other answers for Experiment 1 were: “Perception experiment” (8 subjects), “drug test” (6 subjects), “general reaction to drug / physiological effects” (5), “pain experiment" (4), not specified (4). For Experiment 2, subjects answered: “Test of a therapeutic device” (7 subjects), “general reaction to laser / physiological effects” (6), “perception experiment” (6), “pain experiment” (3), not specified (7).

## Discussion

In this study, we have shown that placebo-induced somatic sensations are a frequent phenomenon that can result from a range of different placebo interventions, with or without tactile afference. While there have been reports of placebo-induced somatic sensations before [16,18,20], this is the first paper to quantify their spatial and temporal characteristics and to compare different placebos.

We found that sensations can reach considerable intensity and spatial extent, underlining the clinical relevance of our findings. Specifically, similar sensations can be triggered by many clinical interventions involving spatially localized stimulation. In our opinion, this phenomenon deserves more research attention.

Different placebos demonstrated mostly similarities in their evoked sensations. We were surprised by the high similarity of qualitative features as well as spatial patterns across subjects in Experiments 1 and 2 (in Experiment 3 they were to be expected). Despite the fact that subjects were not conditioned and received no explicit suggestion as to what sensations they were about to feel, only 9 out of 78 possible McGill questionnaire descriptors were chosen by at least 20 percent of the subjects and together explained 71.7 percent of all sensation choices. Moreover, temporal onset showed a rather broad distribution that was similar for both placebo laser and placebo irritant solution, while spatial pattern analysis revealed a high degree of overlap in localization of sensations between the two placebo interventions. However, we also found differences between placebos. Sensation intensity (VAS) was stronger for placebo laser stimulation and sensations were more widespread (GIS). This is in line with previous reports demonstrating stronger placebo effects from device placebos compared to other types [6,7,9].

Although somatic sensations were evoked by placebo intervention in our study, similar sensations have been reported in response to low-level laser stimulation [20,22], acupuncture [32–34], and so called touch healing [35]. In acupuncture, these sensations are part of the characteristic “Deqi” sensation but can also be produced by placebo acupuncture [17,36]. In touch healing, evoked sensations are referred to as “enhanced touch sensations” [35]. Considering the fact that both verum and placebo stimulation are capable of eliciting sensations that patients frequently associate with treatment efficacy [35], one may interpret these sensations as a “placebo enhancer”, i.e. a phenomenon increasing patients’ belief that “something is happening” as a result of the intervention, which may boost expectancy and trigger a (larger) clinical placebo response.

Although the association between placebo-induced sensations and clinically relevant placebo responses have not been investigated yet, there have been reports that sensory suggestibility affects the magnitude of the placebo effect. For instance, subjects prone to develop sensations upon suggestion showed stronger placebo analgesic responses [37]. Furthermore, indirect support for a link between placebo-induced sensations and clinical placebo effects comes from our observation that novelty seeking, a character trait associated with dopaminergic activity [24,28,29], was significantly less pronounced in subjects susceptible to develop placebo-induced sensations. The same character trait together with other dopamine-related traits has been previously reported to correlate with placebo analgesia [23] and general placebo responses [38], although the exact relationship observed in those studies was opposite to ours. In summary, a dopamine-related mechanism may underlie both, placebo-induced sensations and clinical placebo effects.

An interesting observation was the referral of sensations to body areas remote from the stimulation site. This phenomenon is reminiscent of referred pain, where pain from a somatic or visceral region is referred to remote parts of the body [25,31,39]. We found that spatial patterns of placebo-induced sensations were remarkably similar to those known from experimentally evoked referred pain. Although the quality of placebo-induced somatic sensations seems to differ greatly from that of referred pain, a more careful investigation shows that the two phenomena share many descriptors. While “pressing” is one of the most typical referred pain descriptors [40], there is often an additional paresthetic component to it, frequently described as “tingling” and “radiating” [41]. More support for a common mechanism between referred pain and placebo-induced sensations comes from the average onset times. In our study, subjects reported their first sensation 40.0±46.0 seconds after receiving the information that stimulation had started. This is very similar to the typical onset of referred pain after hypertonic saline injections (e.g. 42.3±32.7 seconds in[42]). Future research should directly evaluate placebo sensations and referred pain in the same individuals.

We can further speculate about underlying mechanisms of placebo sensations on the basis of the most frequent descriptors. Of all 78 possible words from the McGill questionnaire, only nine were chosen by more than 20% of the subjects and account for 71.7 percent of all choices. These descriptors reflect the entire bandwidth of possible somatic sensations: “Warm”, “cool” and “cold” express temperature sensations, while “pulsing” describes an enhanced perception of one’s cardiac pulse. Others, like “pressing” and “tugging” are innocuous tactile sensations, whereas “stinging” and “burning” describe nociceptive afference. The peripheral and central processing mechanisms supporting “tingling”, which was the most frequent sensation, are not well understood. Human microneurography experiments have shown that tingling results from aberrant activity of mechano-sensitive neurons [43,44]. More recent studies have applied natural alkylamids that produce a strong tingling sensation and found that they activate a subset of Aβ-, Aδ- and C-fibres that under normal circumstances convey light-touch information from hairs in the skin [45,46]. Interestingly, tingling sensations can also arise in the complete absence of external stimuli [47].

Due to the lack of tactile input for placebo-induced sensations, specifically for placebo laser, our observations point towards a central rather than a peripheral etiology. The fact that placebo sensations develop in the complete absence of tactile or thermal stimuli together with the sheer number of differential somatosensory sub-systems involved, make involvement of peripheral receptors and fibers unlikely. Moreover, previous studies have demonstrated that tingling sensations, in particular, can be elicited by electrical stimulation of central regions, like the primary and secondary somatosensory cortex or the somatosensory thalamus [48,49], and similar sensations can arise during paresthetic epileptiform seizures [50]. Interestingly, such seizures occur much more frequently on the extremities than on the trunk [50] which has an intriguing parallel in our study, as tingling sensations were significantly more often reported from stimulation of the finger and toe as compared to the abdomen.

More evidence for a central etiology comes from evident similarities between placebo-induced sensations and those seen in some forms of synesthesia and in sensory imagery. Synesthesia is the involuntary experiences in one sensory pathway (“concurrent”) upon stimulation of a different sensory pathway (“inducer”) [51,52]. As has been demonstrated by numerous studies, somatic sensations can be induced by sound in so-called auditory-tactile synesthesia [53], by taste in so-called taste-touch synesthesia and by the sight of other people being touched in so-called mirror-touch synesthesia [54,55]. A common explanation for these phenomena is cross-modal activation of adjacent (or distant but connected) brain regions that under normal circumstances process information from each of the sensory pathways separately [56]. On the other hand, sensory imagery has been shown by recent studies applying functional MRI to be perceptually grounded, i.e. the cortical representation of imagined sensations overlap with those that are actually involved in processing external sensory stimuli, like the primary somatosensory and insular cortices [57,58].

The conclusion of a central etiology, however does not explain, how these central sensations are triggered. While a previous study has shown amplification of tactile sensation by placebo [59], our results are different in that there was no external stimulus to be amplified, particularly for placebo laser, except for possible tonic subliminal receptor activity.

However, as the recent literature on sensory processing (reviewed in [60]) shows, the central dogma of information flow from lower to higher cortical areas is gradually being replaced by a paradigm, in which top-down mechanisms, like expectation and prior knowledge, profoundly shape the way that sensory input is processed. As the authors conclude, under such a view, there is no starting point for information flow. Our results seem to support this paradigm. As the results of Experiment 3 show, patterns of expected and perceived sensations under imagined laser stimulation exhibited a high degree of similarity. Thus, subjects had similar expectations about where sensations would arise, but mainly those with low novelty seeking scores actually developed them.

We note that our definition of the word *placebo* may require some clarification. While previous definitions have implicitly or explicitly assumed a clinical or therapeutic context of placebos (cf. Shapiro’s definition from 1968 [14]), here we have studied sham procedures similar to those commonly used in placebo studies (lasers, topical medication) without explicitly referring to any therapeutic potential. A similar approach has recently been applied to study amplification of tactile sensation by placebo [59]. Despite the lack of a clear medical context, our study can nevertheless be seen as a mechanistic placebo study, informing psychophysical aspects of stimulation by inert substances.

We should also note several limitations. Firstly, we did not use a double-blinded design. This means that in Experiment 1 and 2 we cannot exclude a possible influence of the experimenter carrying out the stimulation. Secondly, our study suffers from the limitation that it cannot eliminate report bias, a phenomenon that is common to any placebo study examining subjective sensations while lacking objective measures or neurobiological correlates [61]. Thirdly, the approach used to map subjects’ sensations did not differentiate between qualities. Thus, it is possible that a subject reporting warm and tingling sensations may have felt a tingling in one and warmth in another area. It may be worthwhile to use separate drawings for each sensation, if subjects report differential spatial distributions. Fourthly, the TCI was only used in Experiment 3, thus, limiting respective findings to the case of imagined stimulation. Fifthly, we note that there are design differences that make Experiment 3 a suboptimal control for Experiments 1 and 2. Experimental demand and response bias as potentially induced by the experimenter were probably much weaker in Experiment 3 and subjects did not have an actual placebo intervention before imagining it. Sixthly, since we have not measured participants’ expectations or prior experience with laser or irritant solution, our interpretation of mechanism regarding expectations and/or conditioning must remain speculative. Future studies may want to conduct the study described in our Experiment 3 in the laboratory, using the same placebo interventions as in Experiments 1 and 2, while informing subjects that the interventions are placebos (overt placebo design). Such an approach could help isolate the specific effect of placebo versus the effect of attention to the body part or sensation under study.

To conclude, the present study has shown that placebo-induced sensations are frequent and can reach considerable intensity and extent. Their spatial pattern and sensory quality exhibit high similarity across subjects. Interestingly, different modalities of placebo interventions produce different intensities of placebo sensations. Since a multitude of somatosensory subsystems are involved despite the lack of a peripheral stimulus, we propose a central etiology of the phenomenon. Future studies should distinguish the neural underpinnings of these sensations with functional neuroimaging methods.

## Supporting Information

S1 Fig Body outline used in experiments 1 and 2. (TIF)

S2 Fig Sample page of the questionnaire used in experiment 3. (TIF)

S3 Fig Absolute sensation frequencies for the different placebo types and body regions. (TIF)

S4 Fig Mean intensities for the different descriptors. (TIF)

S5 Fig Relative descriptor frequencies for the different body regions. (TIF)

S1 Raw Data Raw data from this study. (ZIP)
