# Supplementary figures and images for: Placebo-Induced Somatic Sensations: A Multi-Modal Study of Three Different Placebo Interventions (part 1 of 3)
Source: PLoS One. 2015 Apr 22;10(4):e0124808. doi: 10.1371/journal.pone.0124808 (PMC4406515; doi:10.1371/journal.pone.0124808)

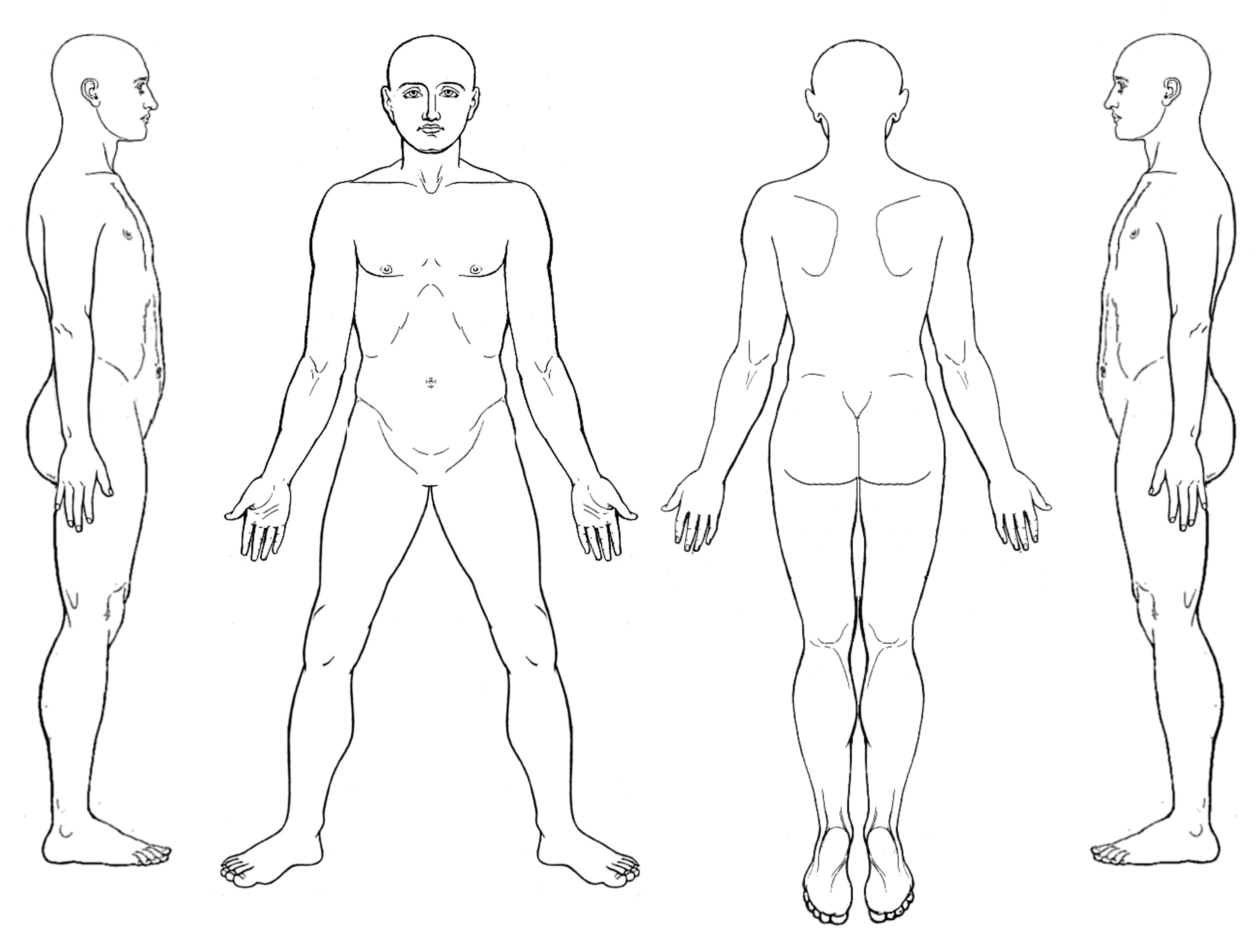

Supplement: S1 Fig — (TIF) [file pone.0124808.s001.tif]

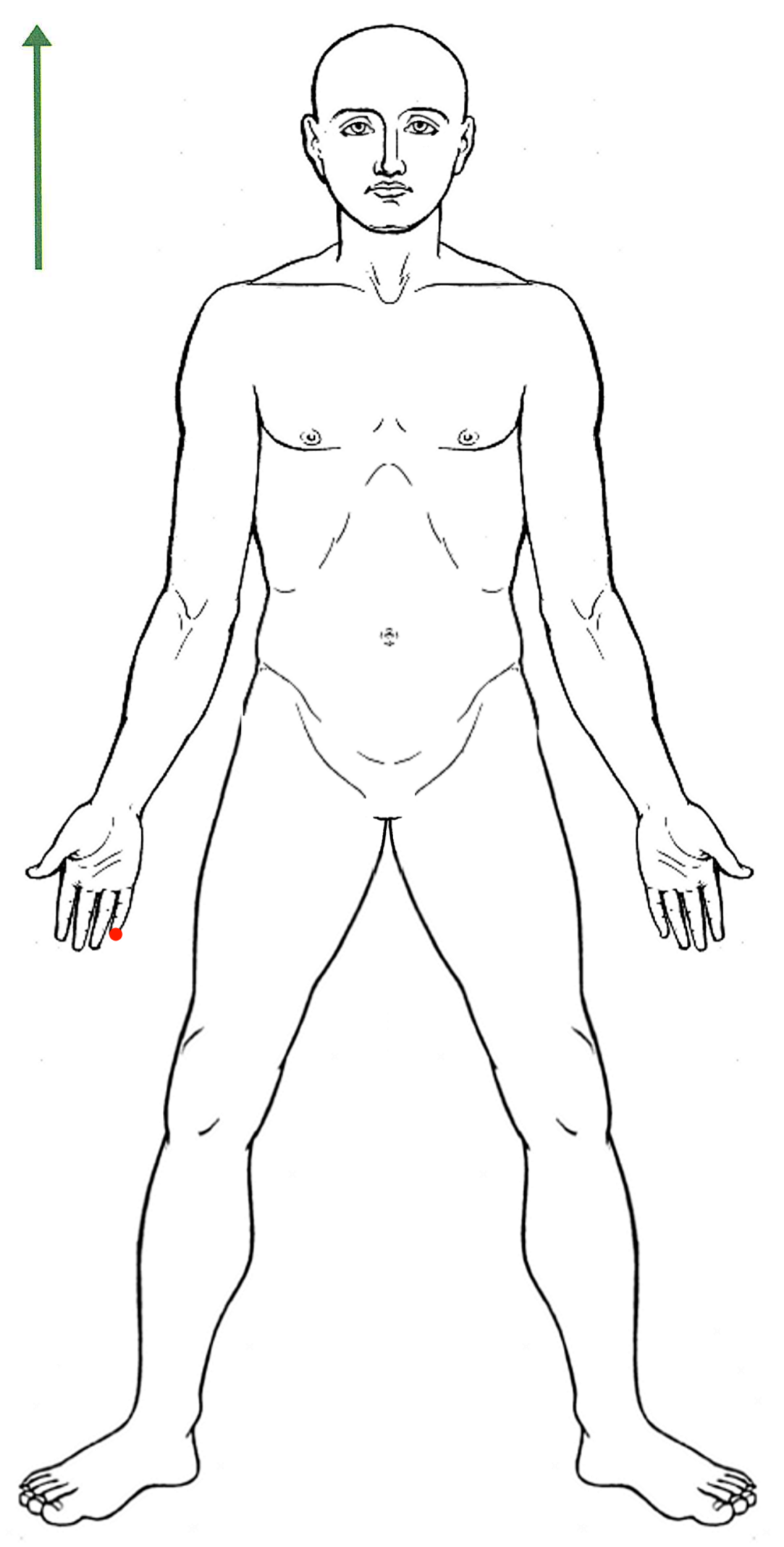

Supplement: S2 Fig — (TIF) [file pone.0124808.s002.tif]

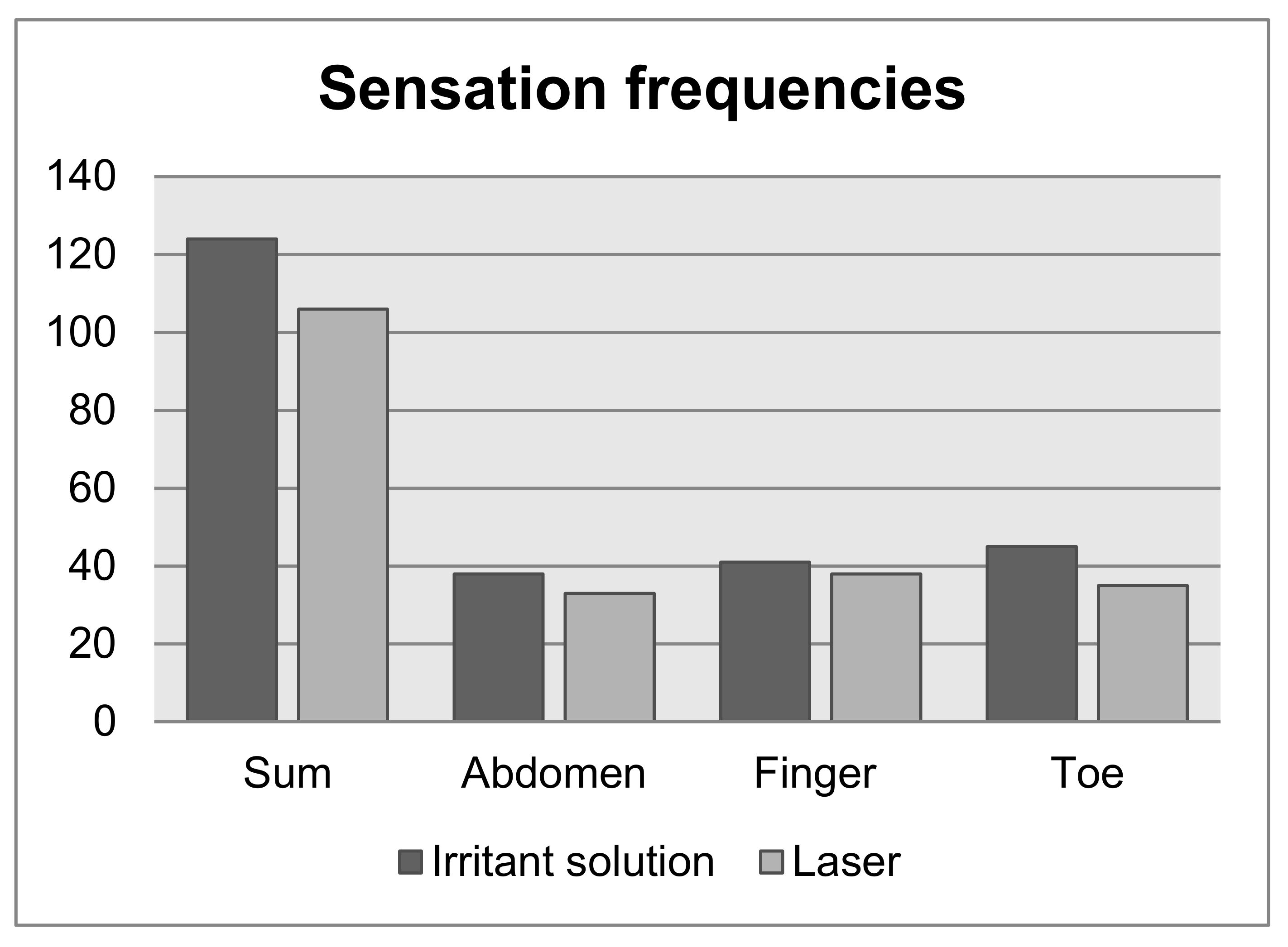

Supplement: S3 Fig — (TIF) [file pone.0124808.s003.tif]

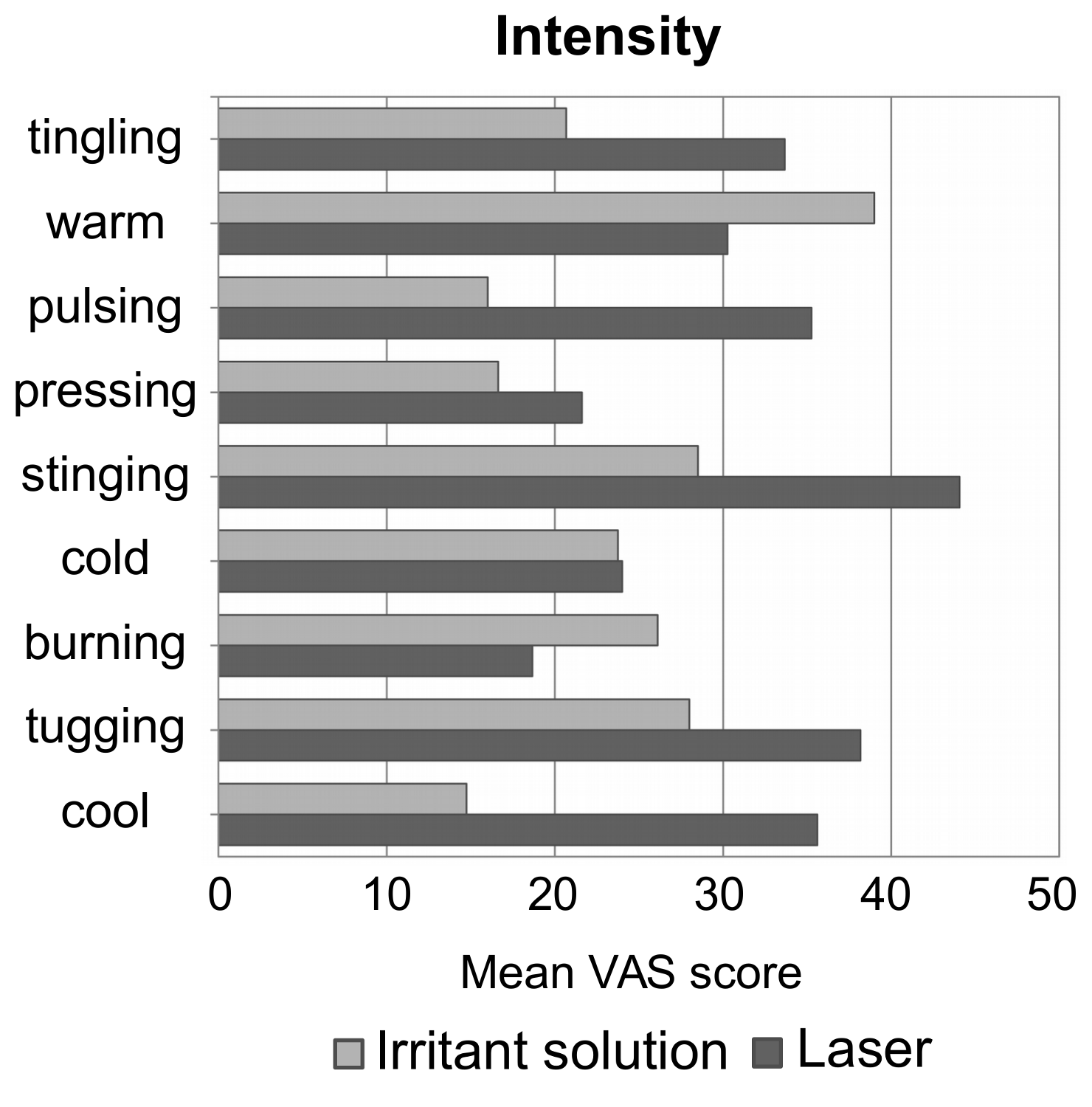

Supplement: S4 Fig — (TIF) [file pone.0124808.s004.tif]

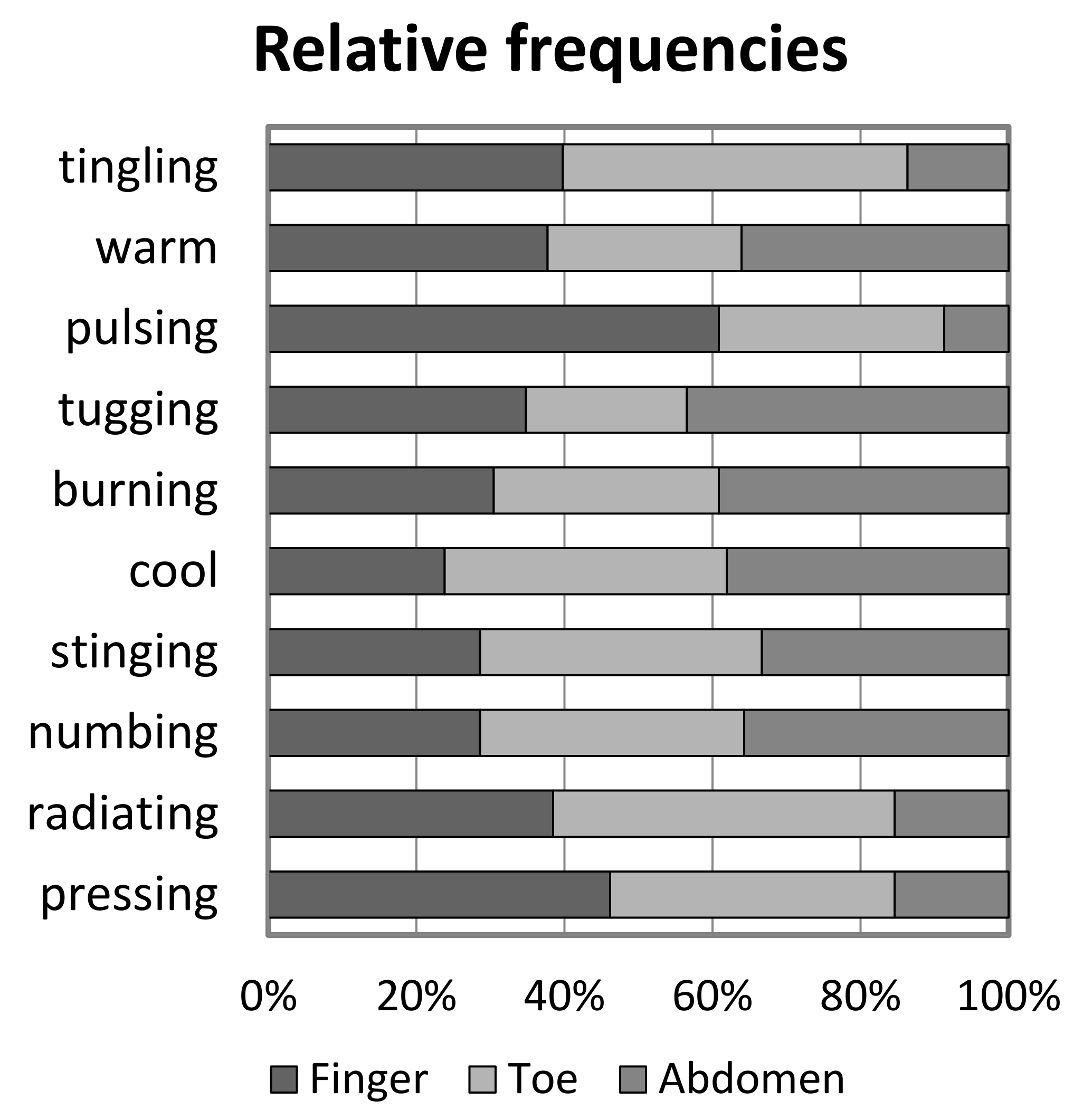

Supplement: S5 Fig — (TIF) [file pone.0124808.s005.tif]

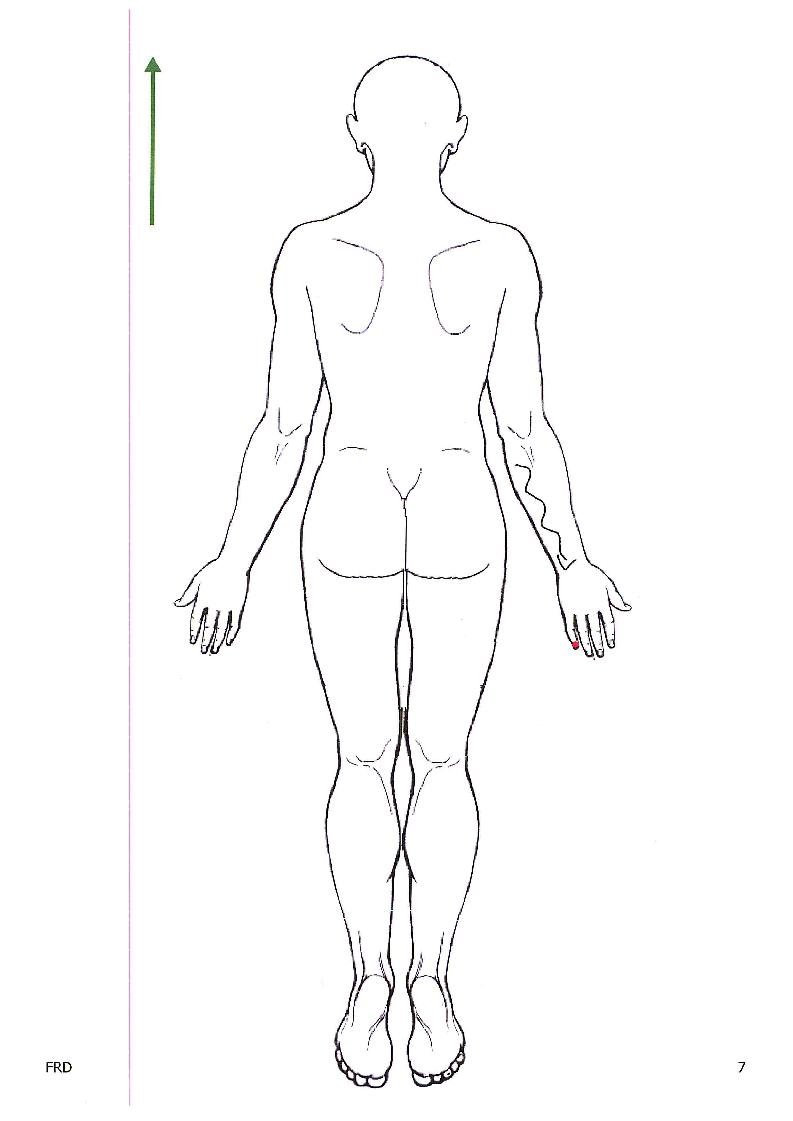

Supplement: S1 Raw Data — (ZIP) [file pone.0124808.s006.zip › Drawings - Imagined stimulation/finger_back/Subject_26_finger_back.jpg]

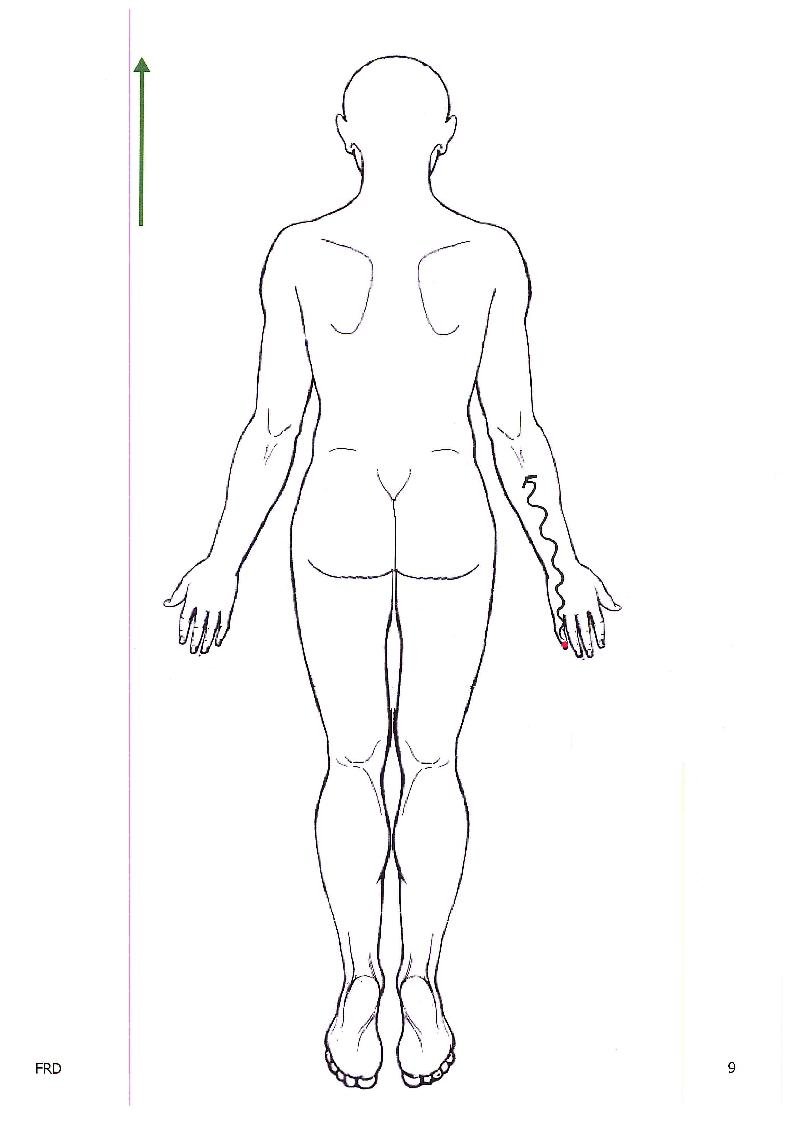

Supplement: S1 Raw Data — (ZIP) [file pone.0124808.s006.zip › Drawings - Imagined stimulation/finger_back/Subject_23_finger_back.jpg]

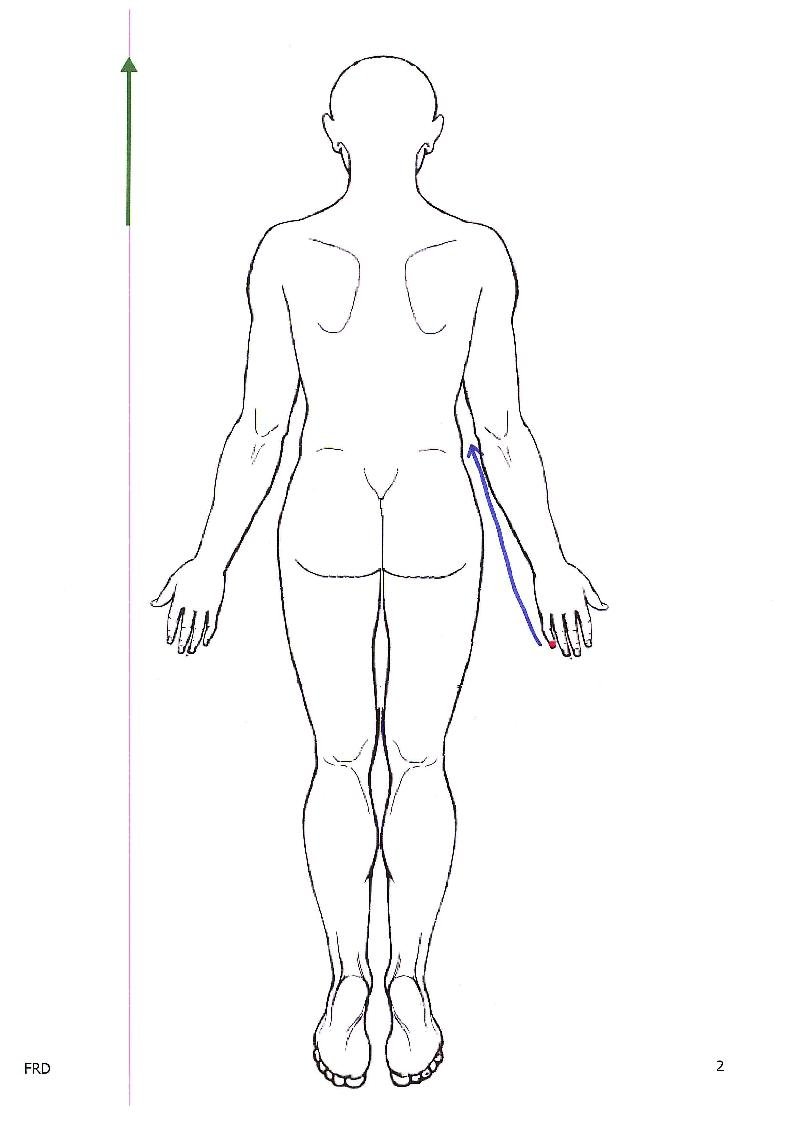

Supplement: S1 Raw Data — (ZIP) [file pone.0124808.s006.zip › Drawings - Imagined stimulation/finger_back/Subject_36_finger_back.jpg]

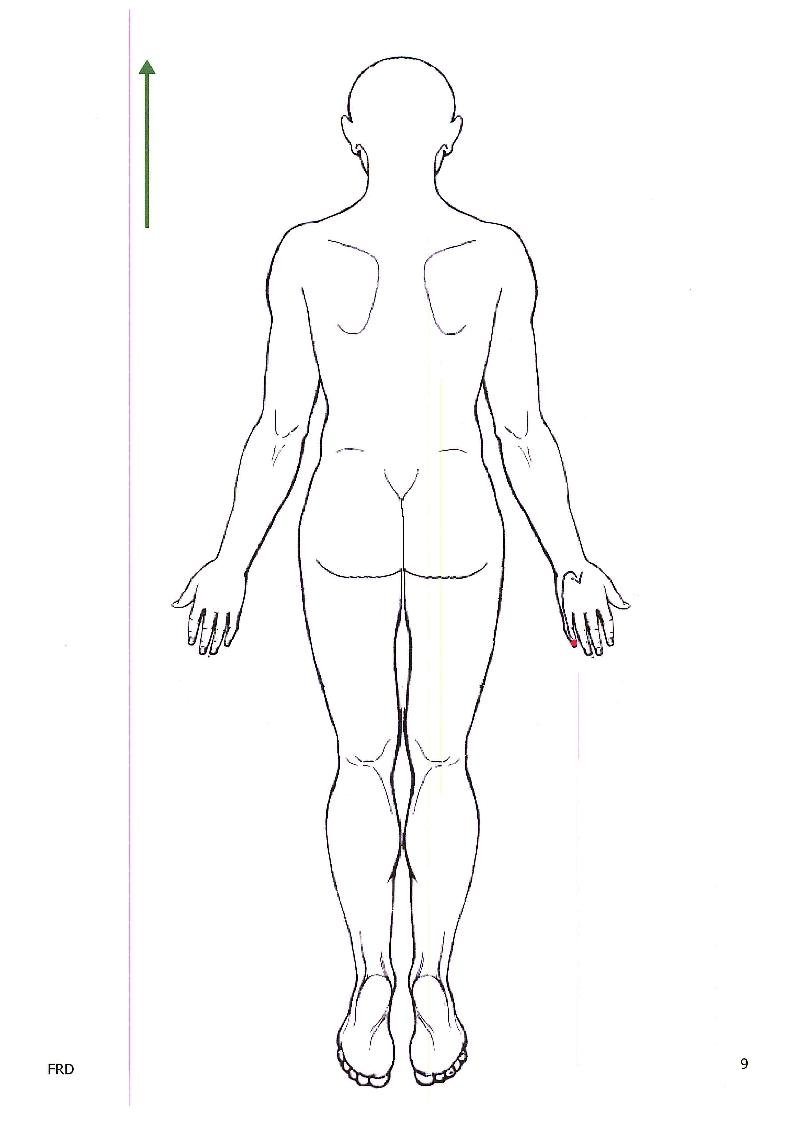

Supplement: S1 Raw Data — (ZIP) [file pone.0124808.s006.zip › Drawings - Imagined stimulation/finger_back/Subject_46_finger_back.jpg]

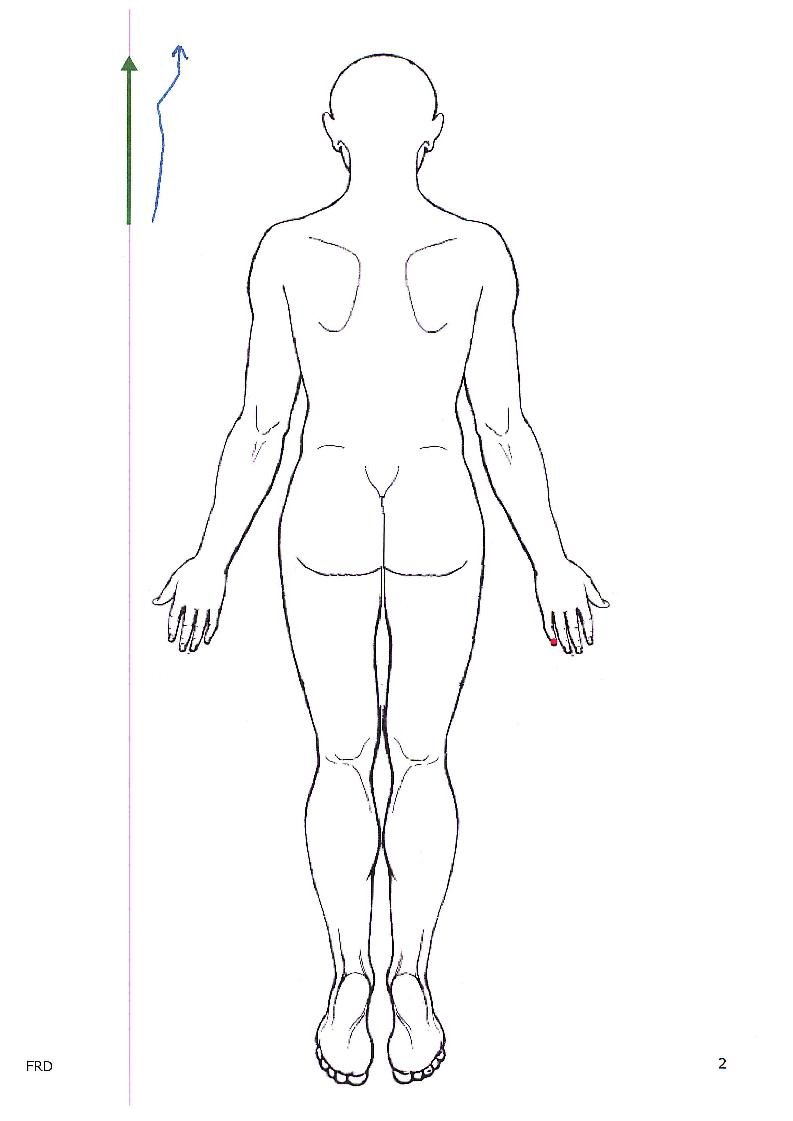

Supplement: S1 Raw Data — (ZIP) [file pone.0124808.s006.zip › Drawings - Imagined stimulation/finger_back/Subject_56_finger_back.jpg]

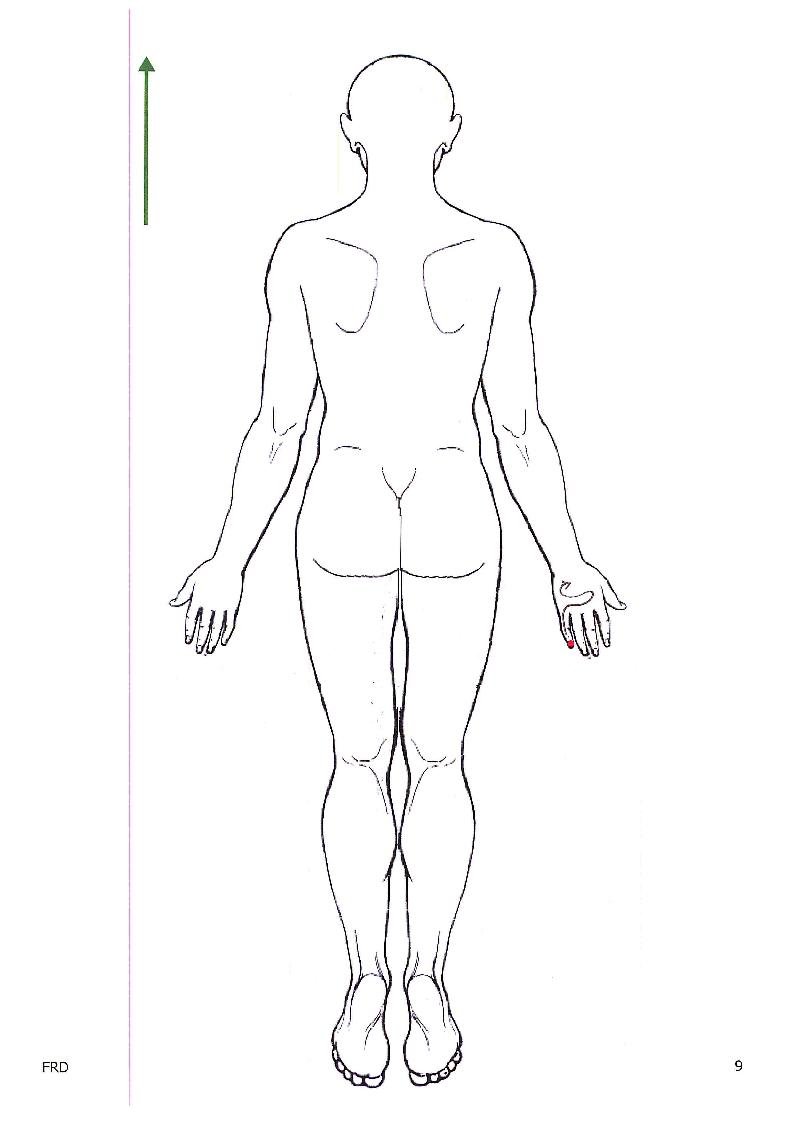

Supplement: S1 Raw Data — (ZIP) [file pone.0124808.s006.zip › Drawings - Imagined stimulation/finger_back/Subject_18_finger_back.jpg]

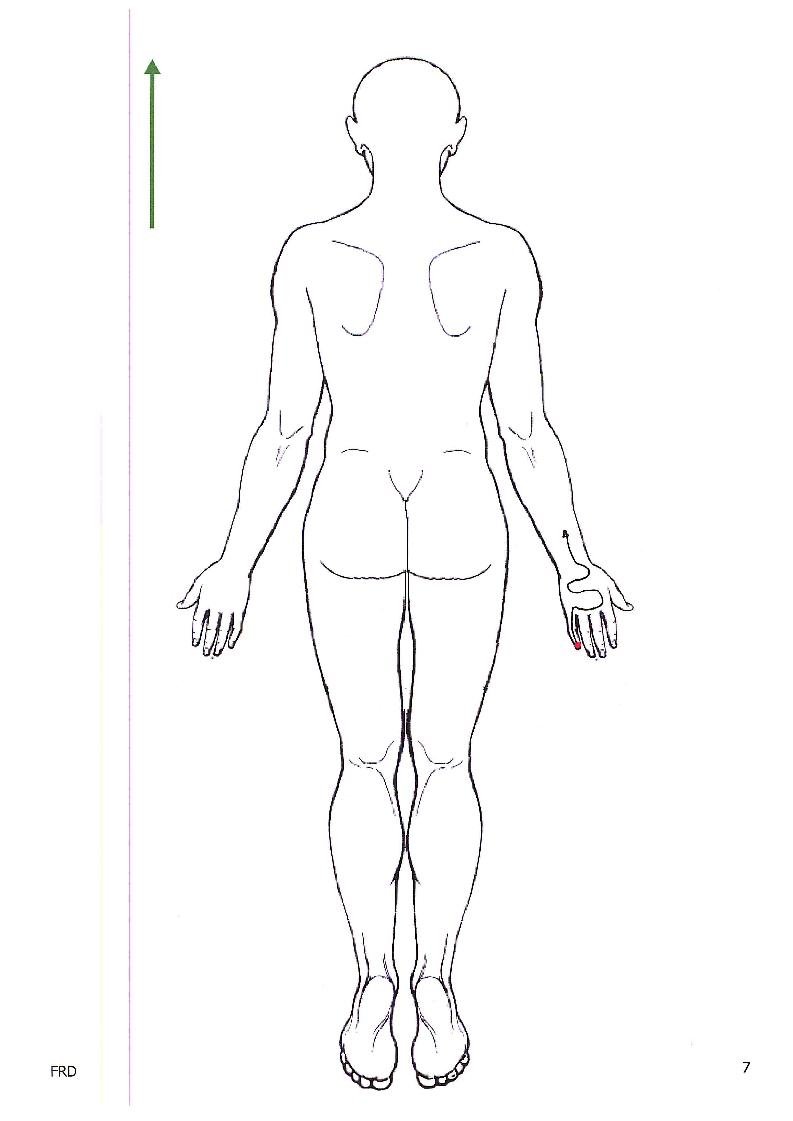

Supplement: S1 Raw Data — (ZIP) [file pone.0124808.s006.zip › Drawings - Imagined stimulation/finger_back/Subject_15_finger_back.jpg]

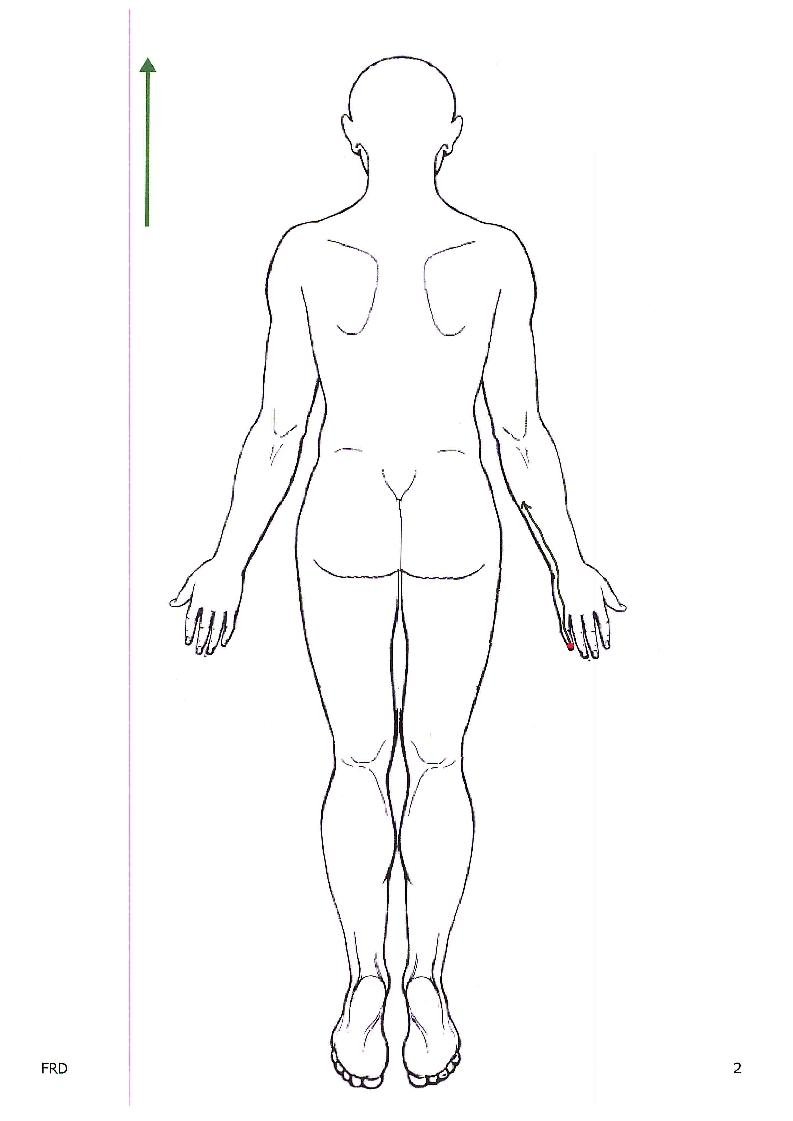

Supplement: S1 Raw Data — (ZIP) [file pone.0124808.s006.zip › Drawings - Imagined stimulation/finger_back/Subject_7_finger_back.jpg]

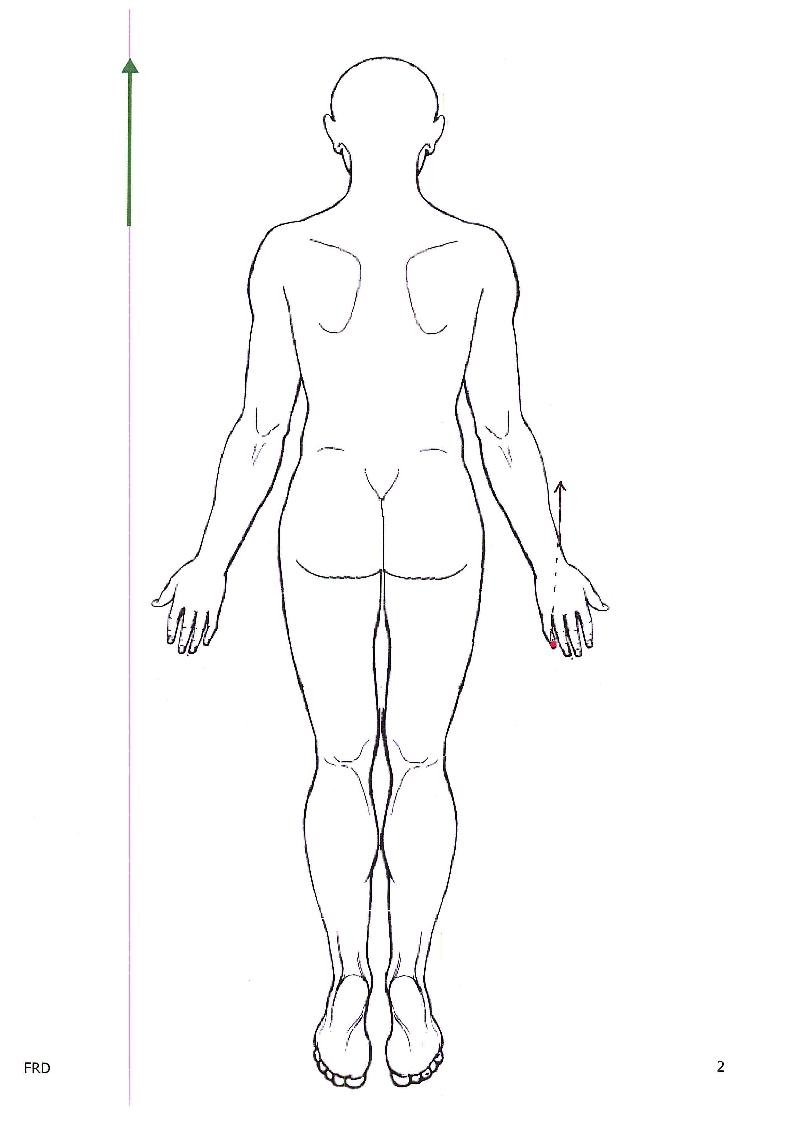

Supplement: S1 Raw Data — (ZIP) [file pone.0124808.s006.zip › Drawings - Imagined stimulation/finger_back/Subject_4_finger_back.jpg]

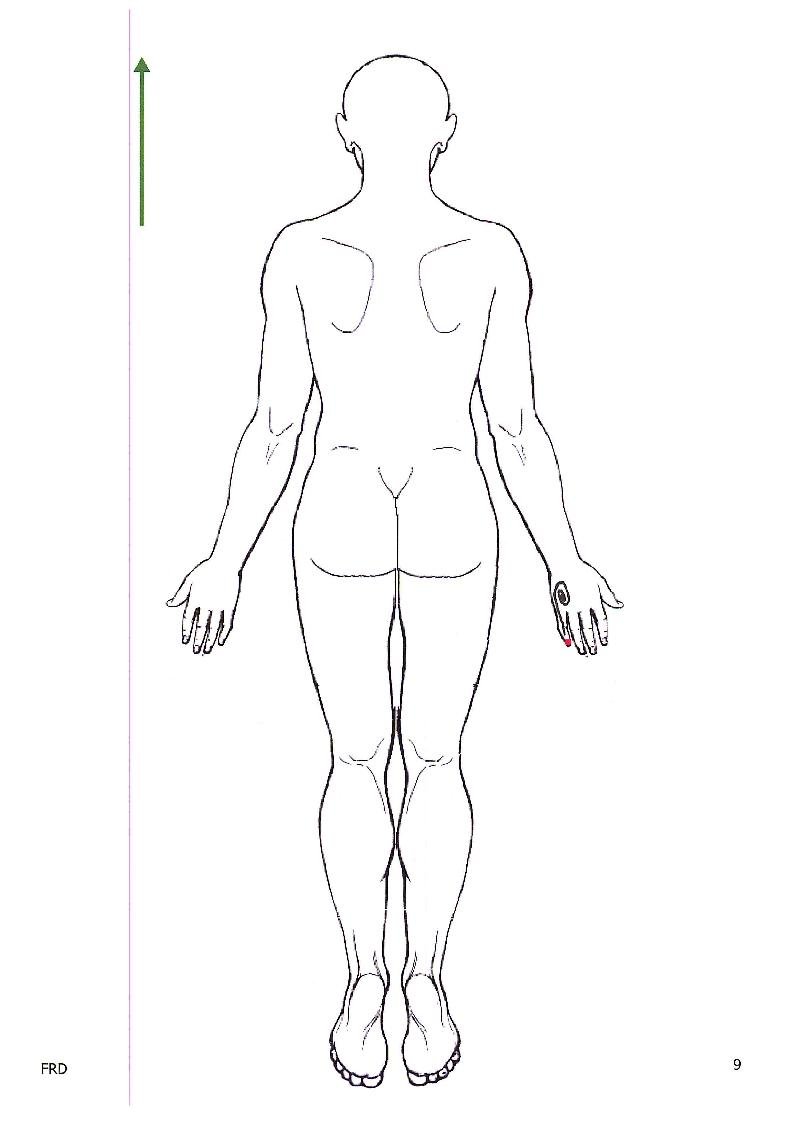

Supplement: S1 Raw Data — (ZIP) [file pone.0124808.s006.zip › Drawings - Imagined stimulation/finger_back/Subject_28_finger_back.jpg]

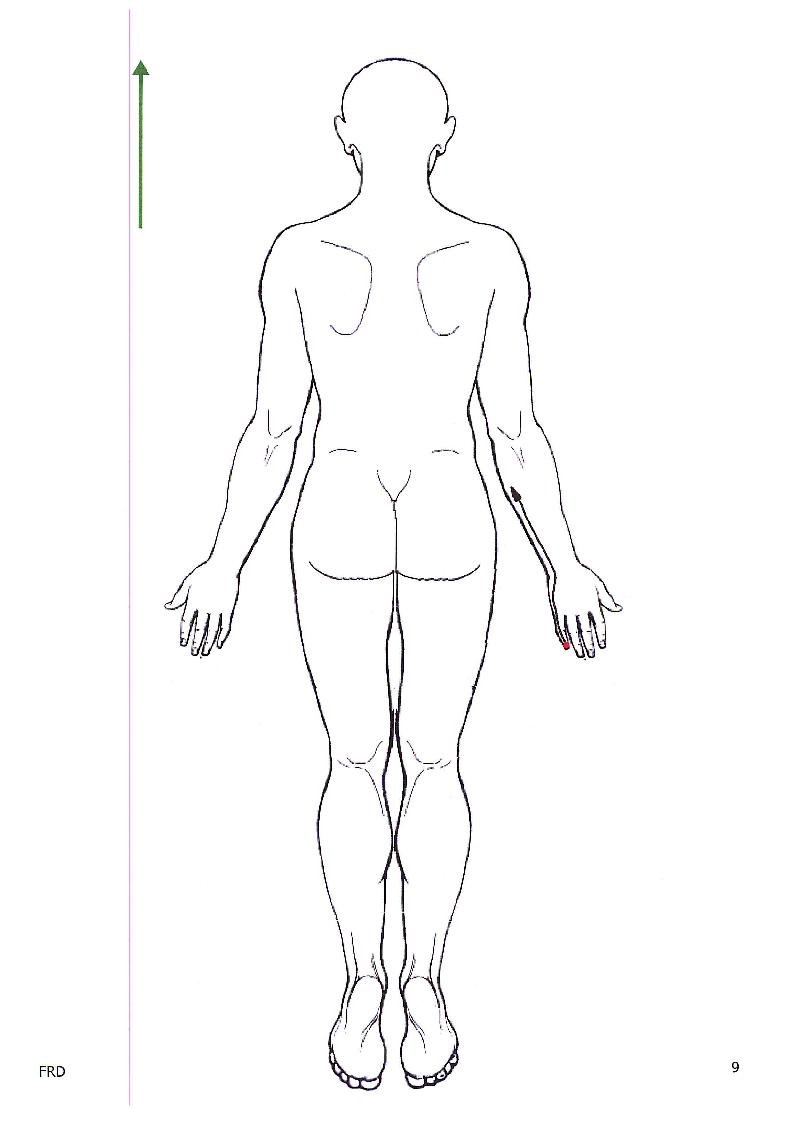

Supplement: S1 Raw Data — (ZIP) [file pone.0124808.s006.zip › Drawings - Imagined stimulation/finger_back/Subject_25_finger_back.jpg]

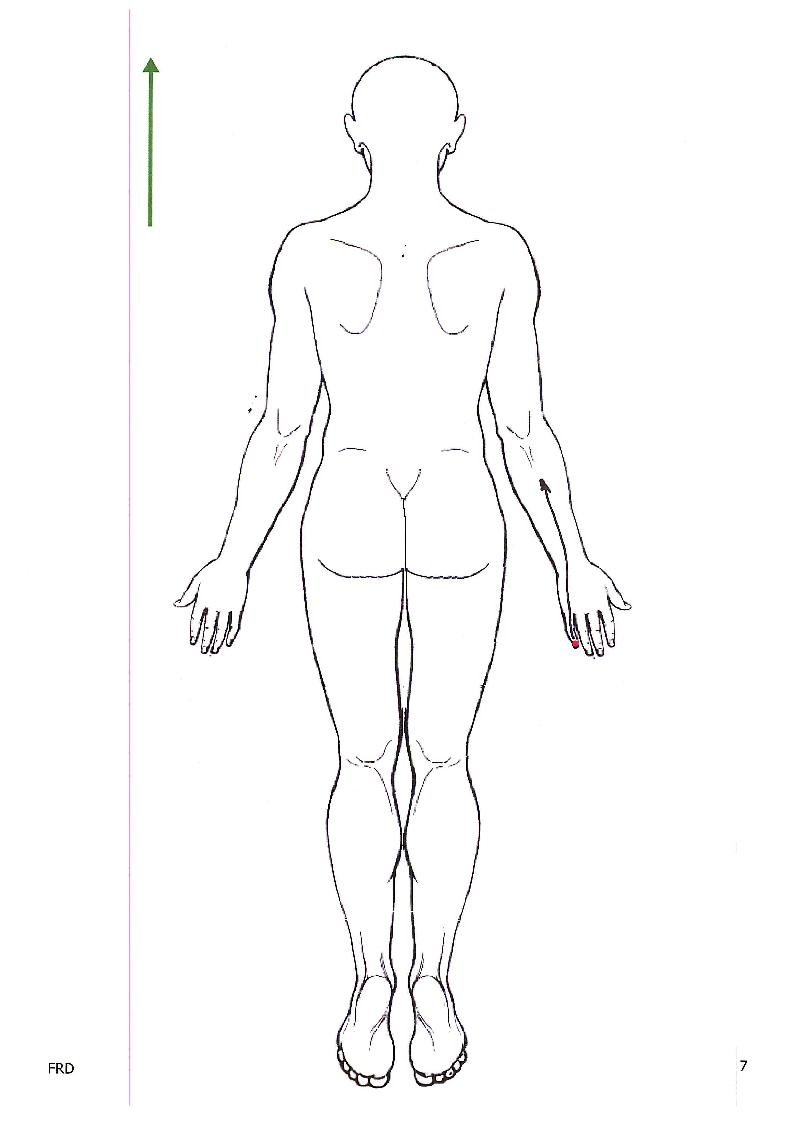

Supplement: S1 Raw Data — (ZIP) [file pone.0124808.s006.zip › Drawings - Imagined stimulation/finger_back/Subject_35_finger_back.jpg]

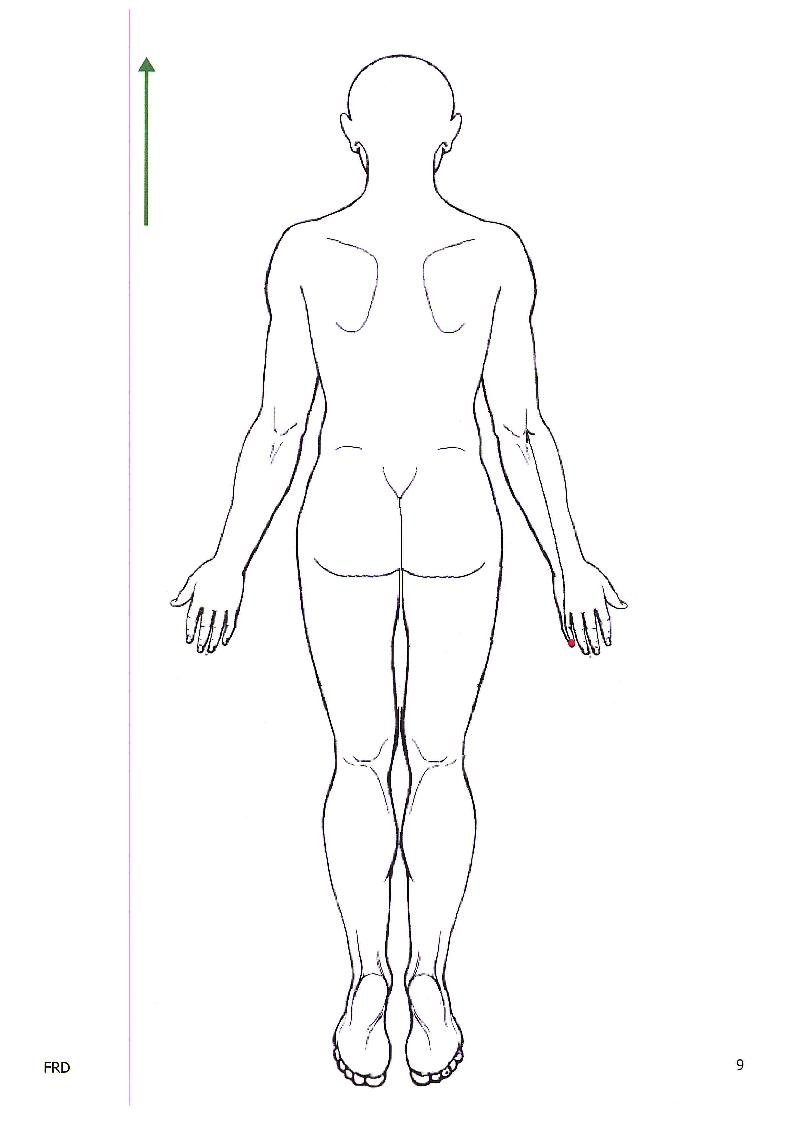

Supplement: S1 Raw Data — (ZIP) [file pone.0124808.s006.zip › Drawings - Imagined stimulation/finger_back/Subject_38_finger_back.jpg]

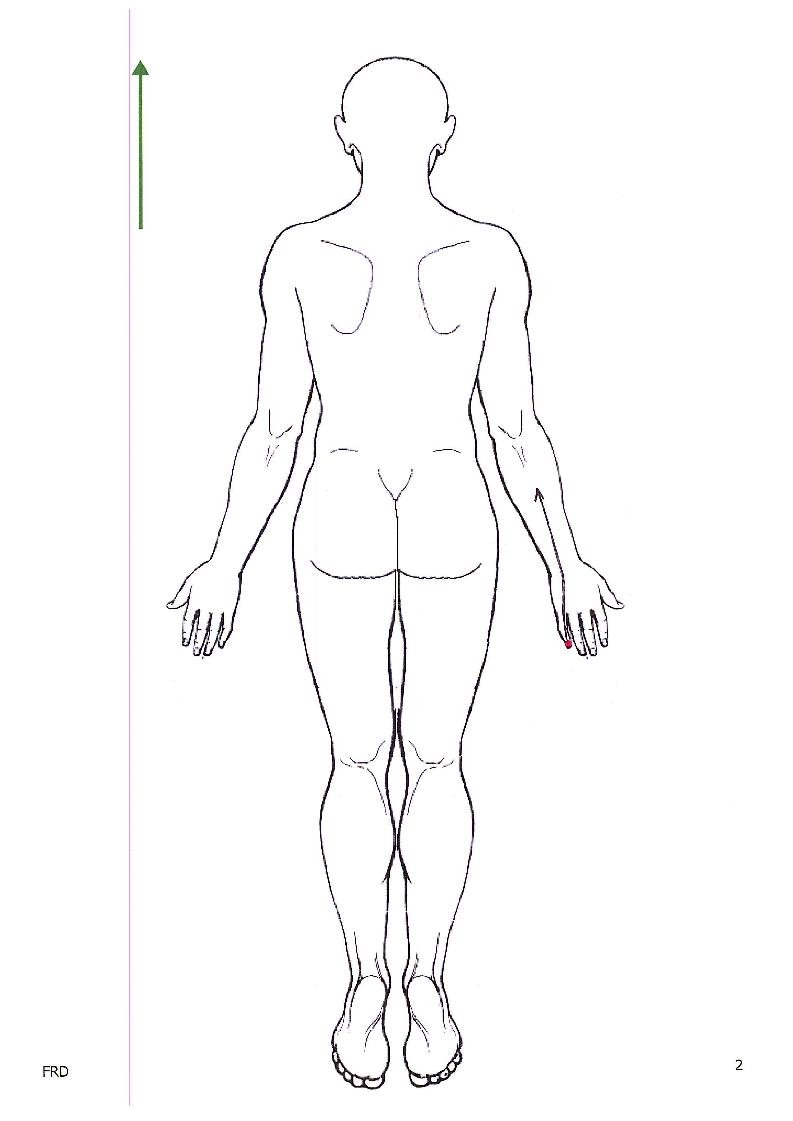

Supplement: S1 Raw Data — (ZIP) [file pone.0124808.s006.zip › Drawings - Imagined stimulation/finger_back/Subject_48_finger_back.jpg]

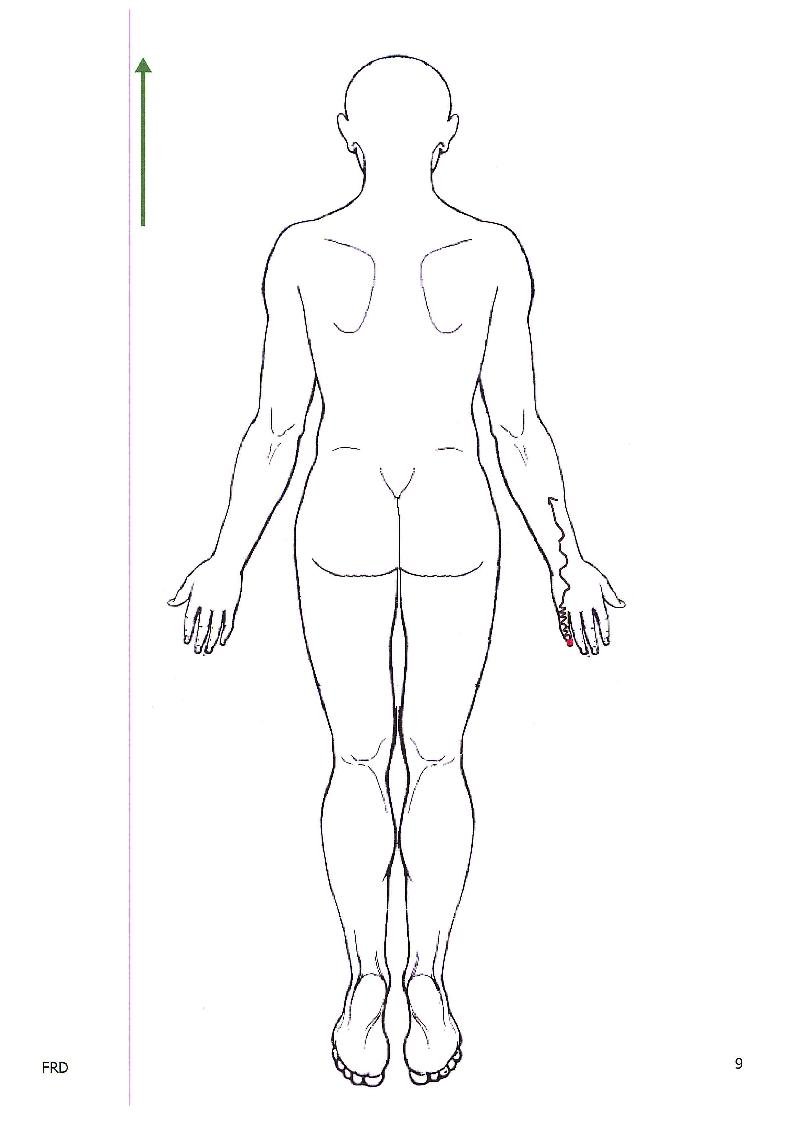

Supplement: S1 Raw Data — (ZIP) [file pone.0124808.s006.zip › Drawings - Imagined stimulation/finger_back/Subject_45_finger_back.jpg]

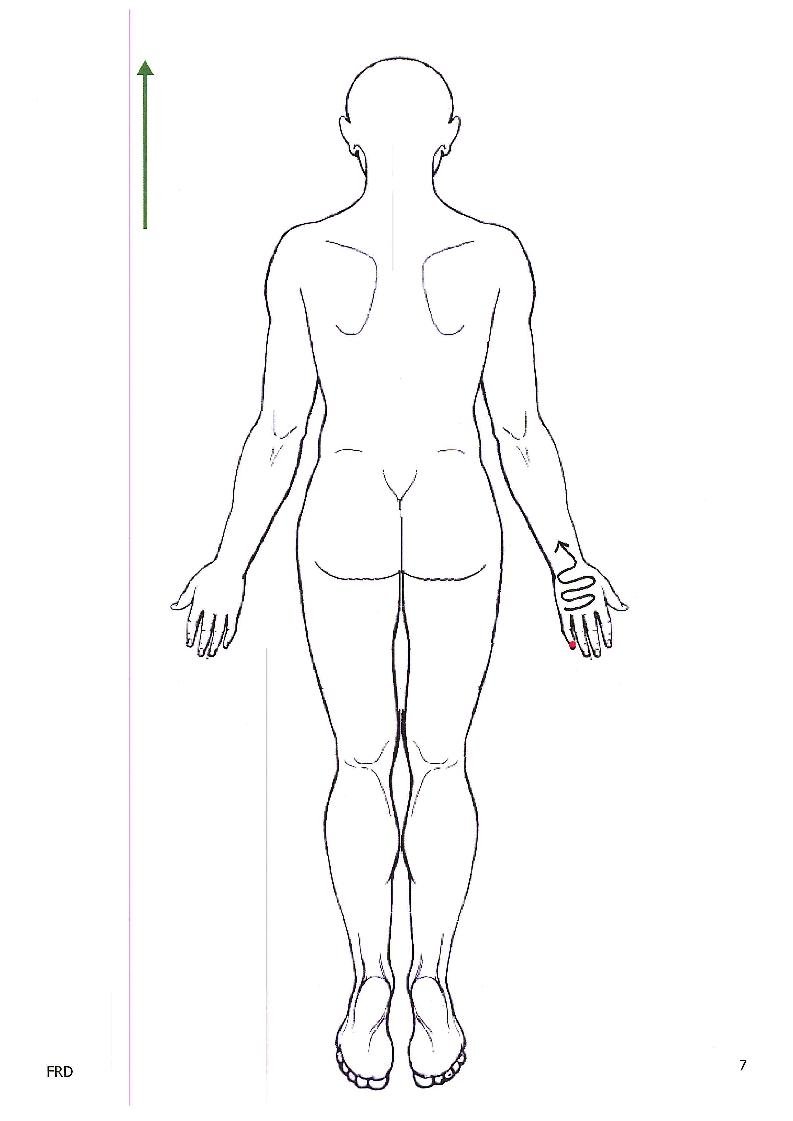

Supplement: S1 Raw Data — (ZIP) [file pone.0124808.s006.zip › Drawings - Imagined stimulation/finger_back/Subject_11_finger_back.jpg]

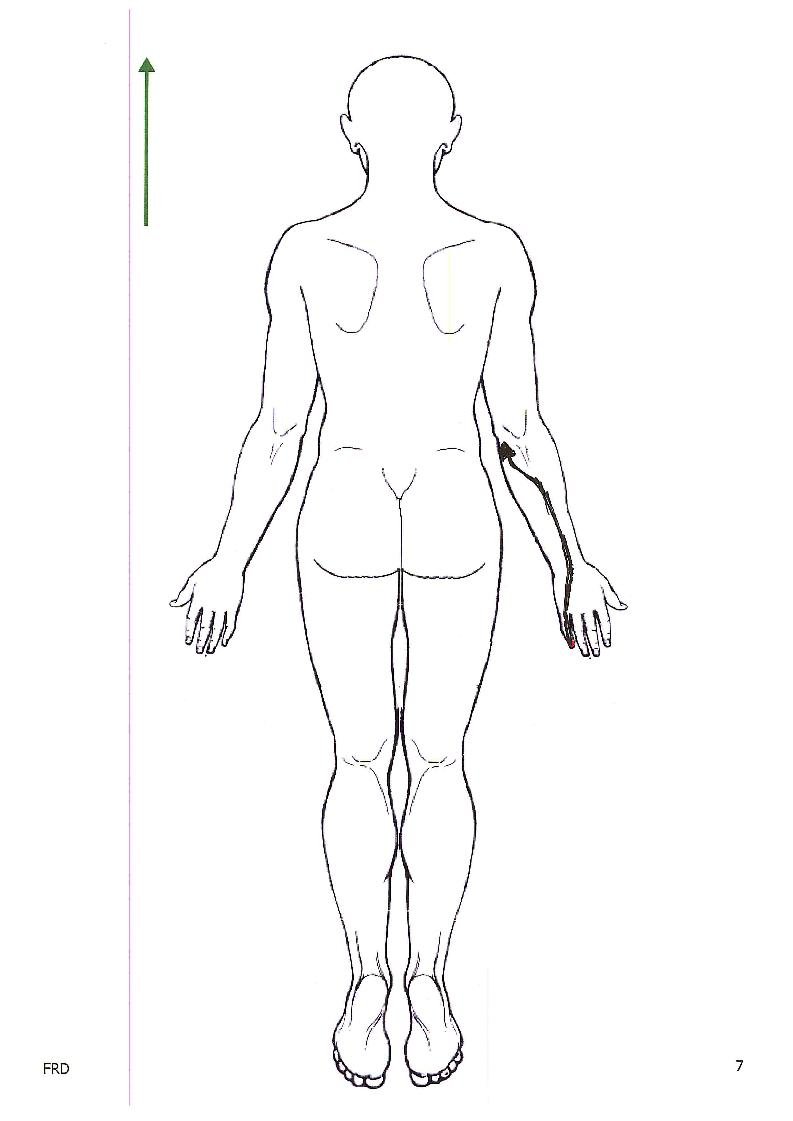

Supplement: S1 Raw Data — (ZIP) [file pone.0124808.s006.zip › Drawings - Imagined stimulation/finger_back/Subject_17_finger_back.jpg]

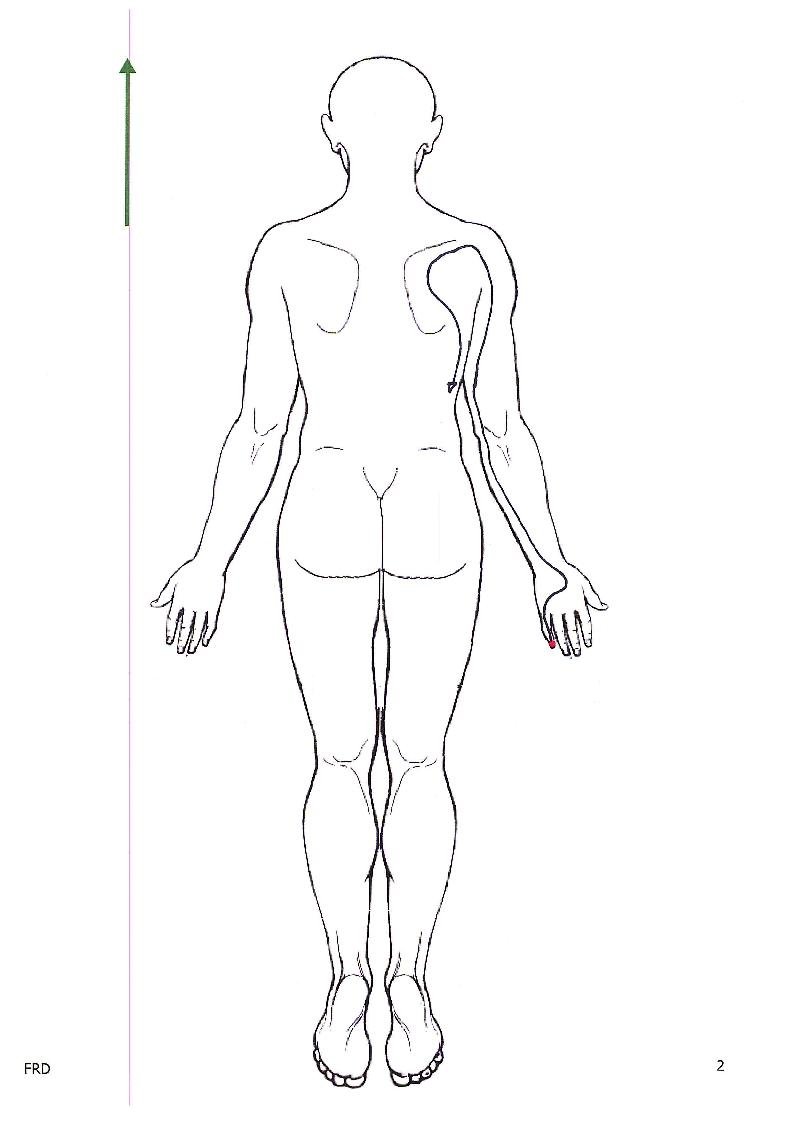

Supplement: S1 Raw Data — (ZIP) [file pone.0124808.s006.zip › Drawings - Imagined stimulation/finger_back/Subject_9_finger_back.jpg]

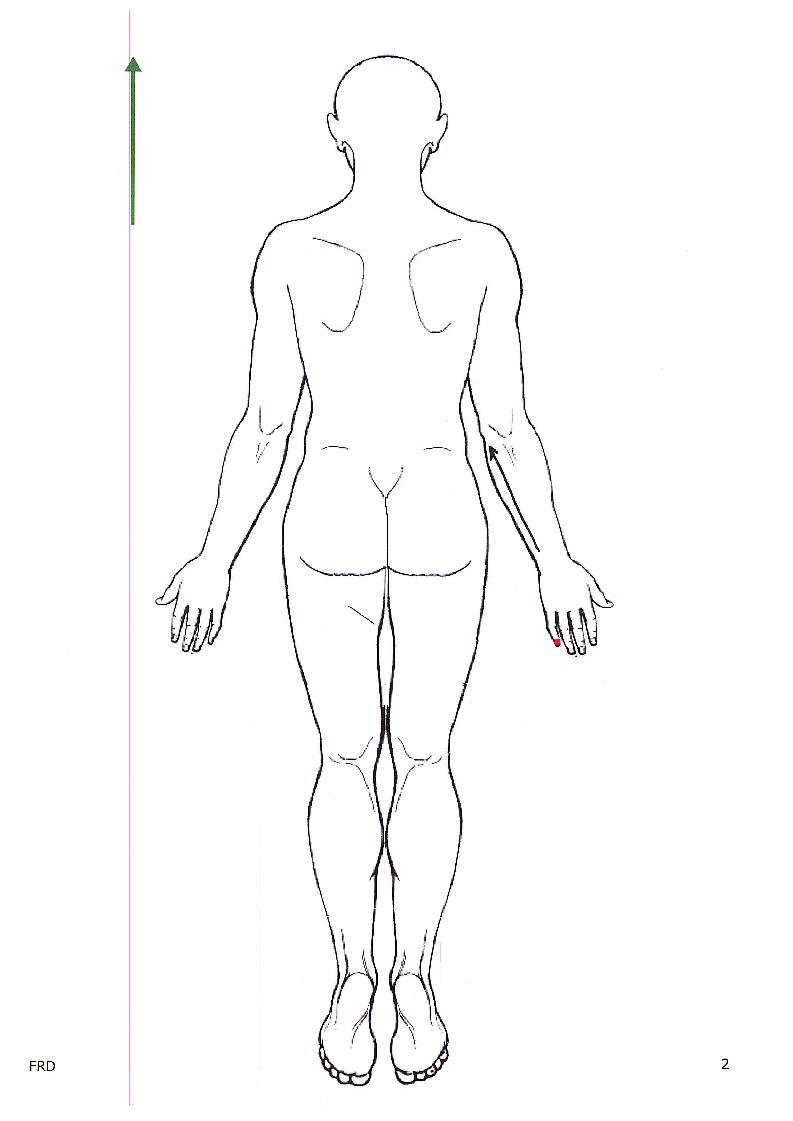

Supplement: S1 Raw Data — (ZIP) [file pone.0124808.s006.zip › Drawings - Imagined stimulation/finger_back/Subject_6_finger_back.jpg]

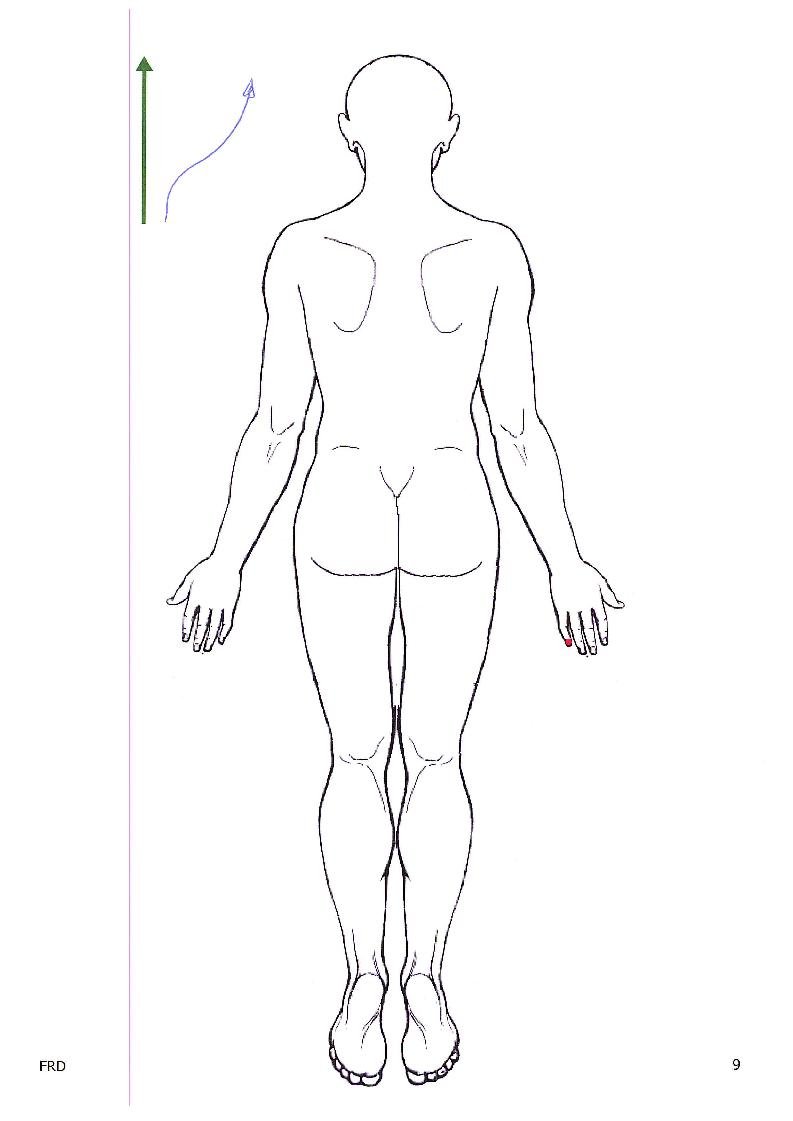

Supplement: S1 Raw Data — (ZIP) [file pone.0124808.s006.zip › Drawings - Imagined stimulation/finger_back/Subject_21_finger_back.jpg]

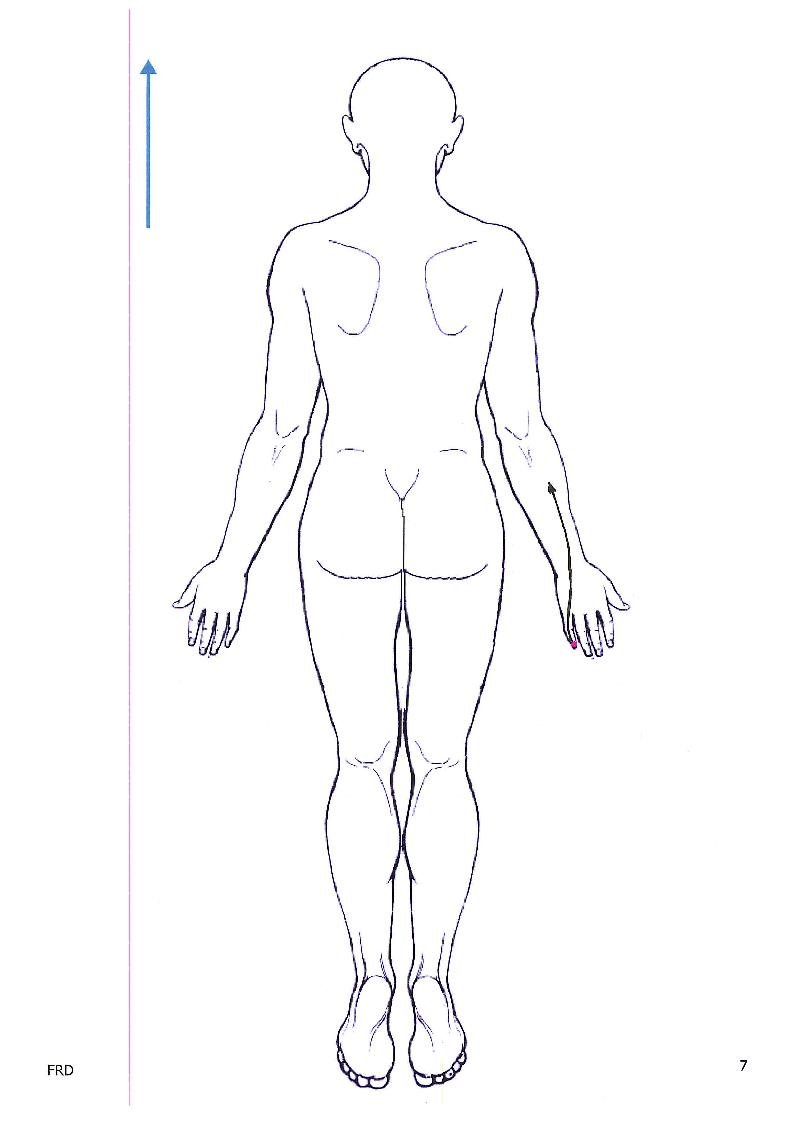

Supplement: S1 Raw Data — (ZIP) [file pone.0124808.s006.zip › Drawings - Imagined stimulation/finger_back/Subject_27_finger_back.jpg]

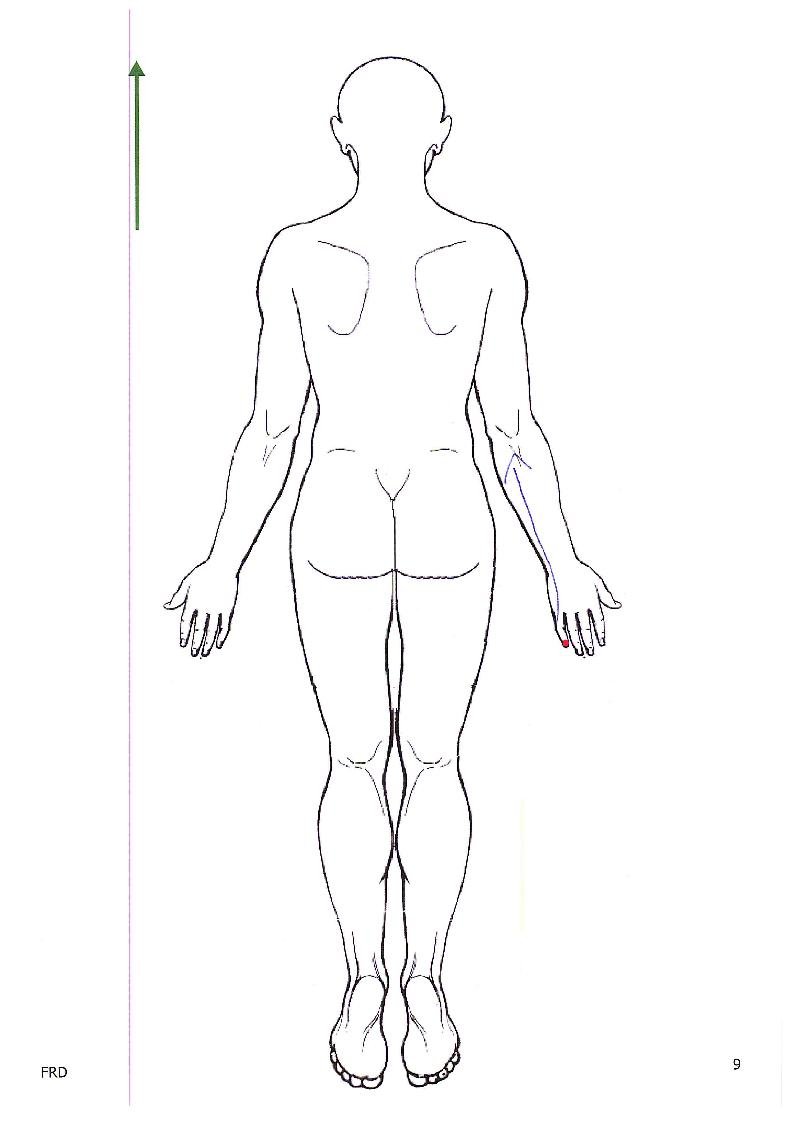

Supplement: S1 Raw Data — (ZIP) [file pone.0124808.s006.zip › Drawings - Imagined stimulation/finger_back/Subject_31_finger_back.jpg]

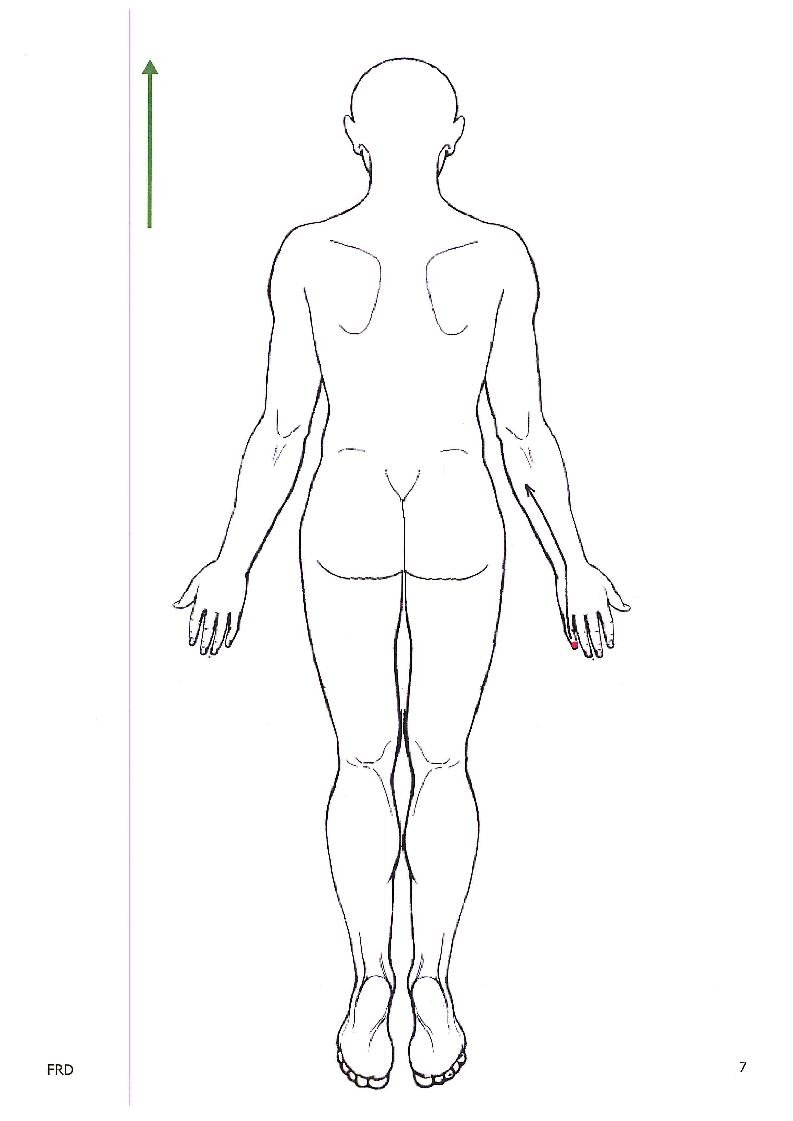

Supplement: S1 Raw Data — (ZIP) [file pone.0124808.s006.zip › Drawings - Imagined stimulation/finger_back/Subject_37_finger_back.jpg]

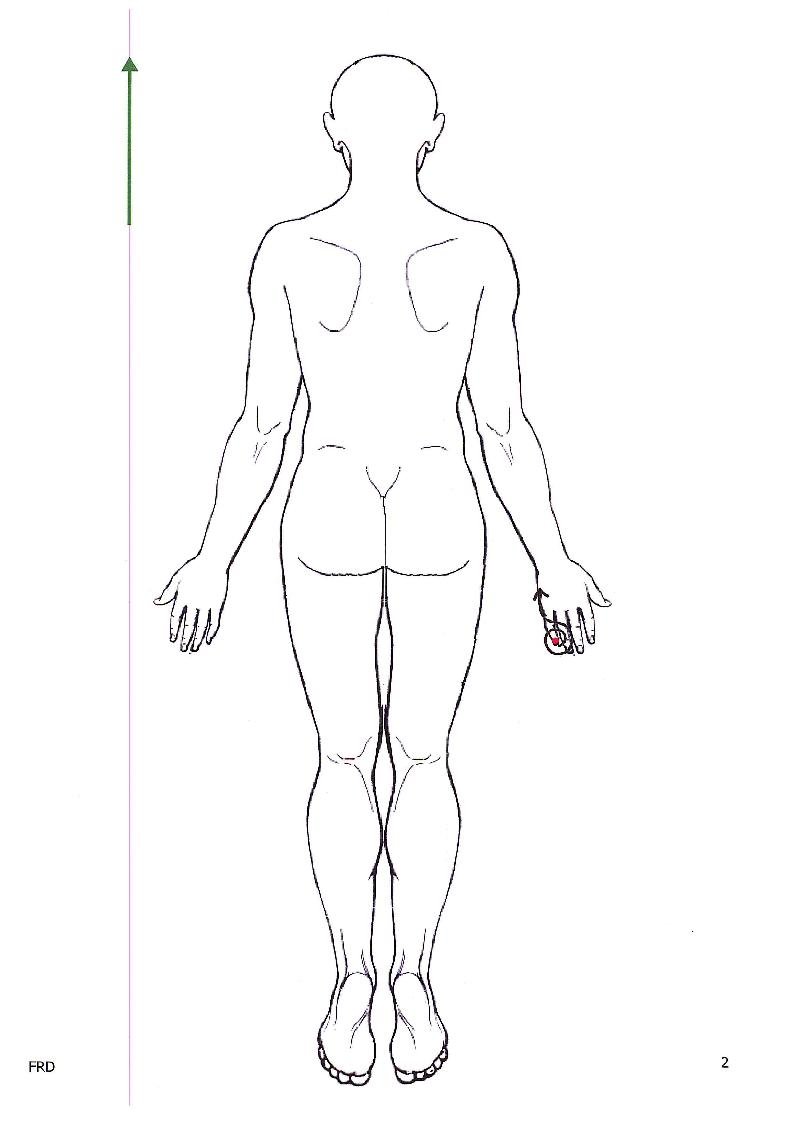

Supplement: S1 Raw Data — (ZIP) [file pone.0124808.s006.zip › Drawings - Imagined stimulation/finger_back/Subject_41_finger_back.jpg]

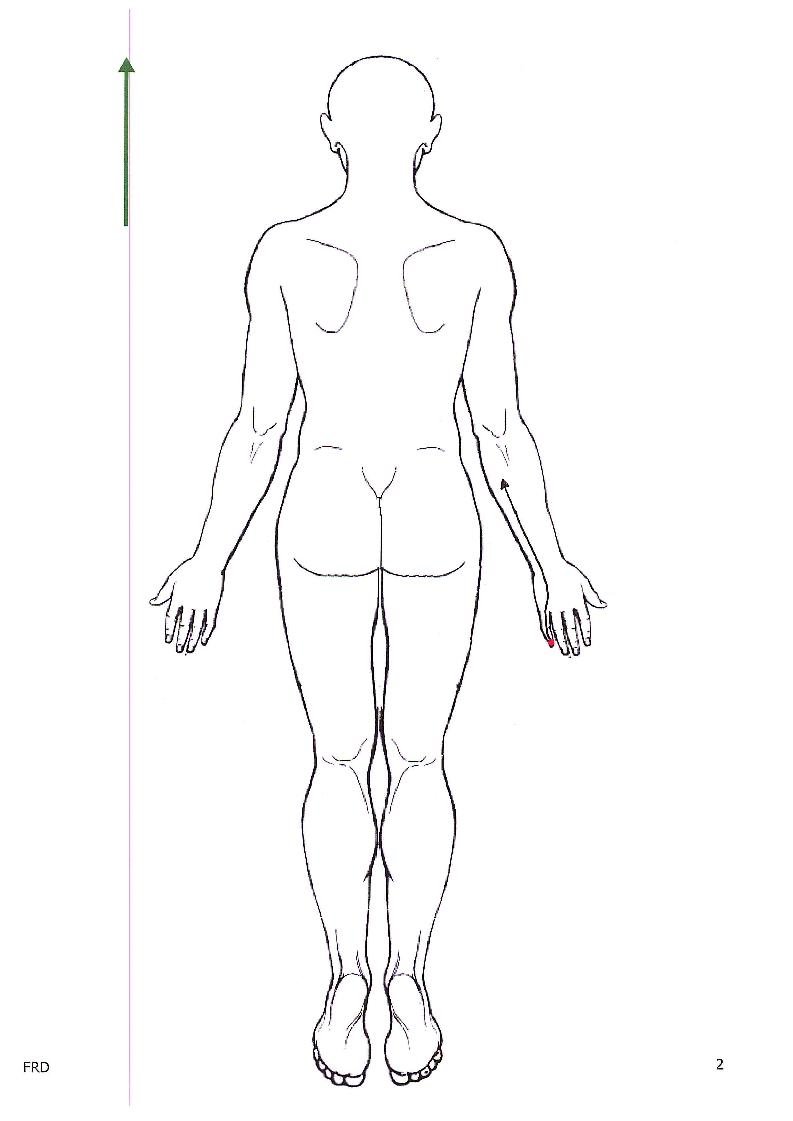

Supplement: S1 Raw Data — (ZIP) [file pone.0124808.s006.zip › Drawings - Imagined stimulation/finger_back/Subject_47_finger_back.jpg]

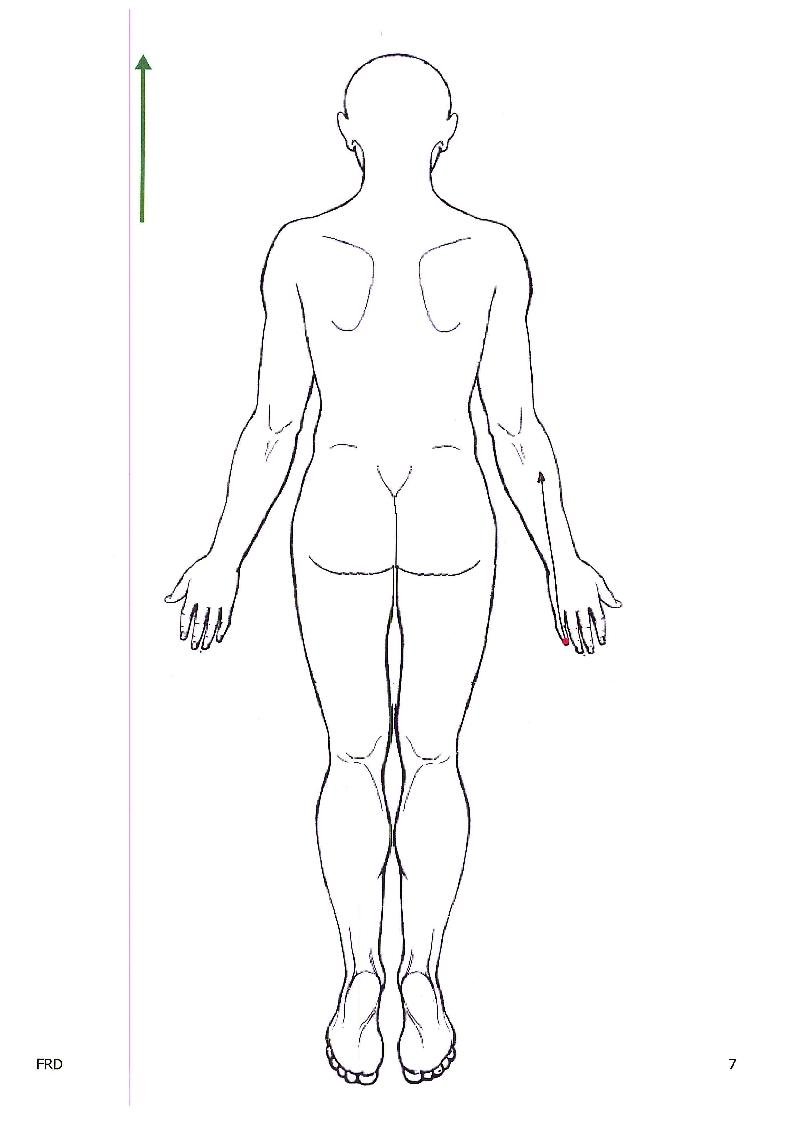

Supplement: S1 Raw Data — (ZIP) [file pone.0124808.s006.zip › Drawings - Imagined stimulation/finger_back/Subject_51_finger_back.jpg]

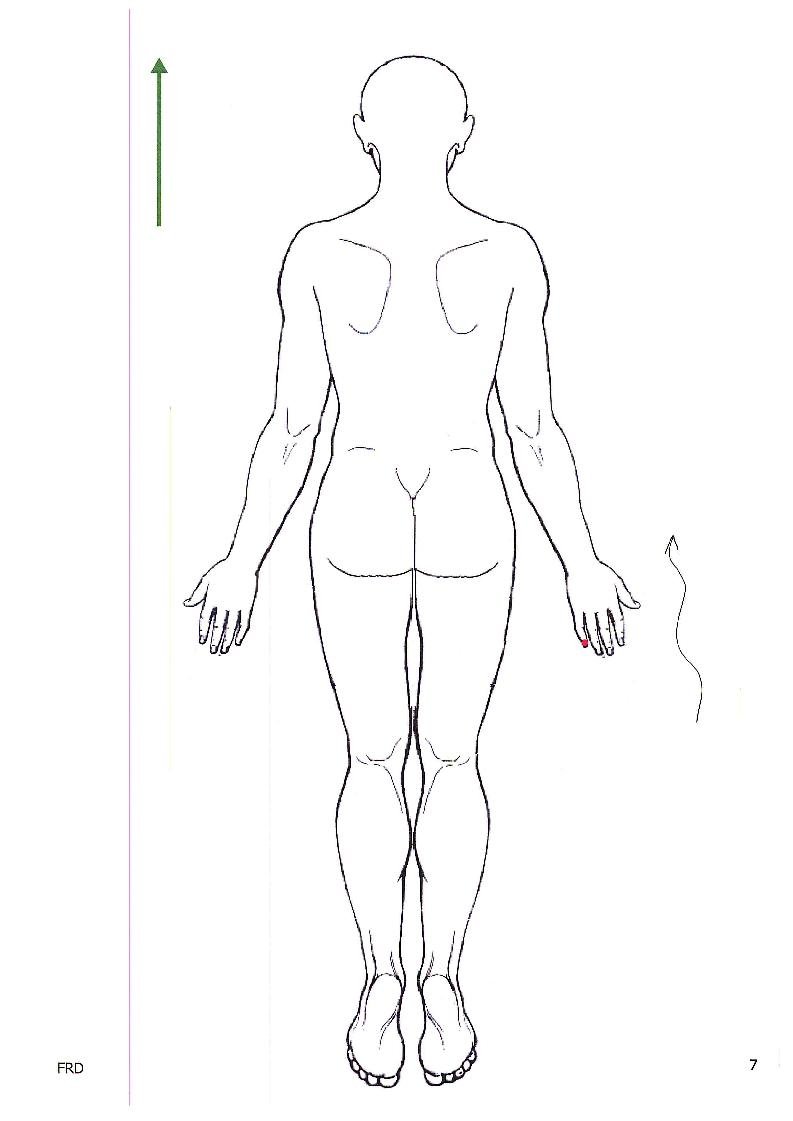

Supplement: S1 Raw Data — (ZIP) [file pone.0124808.s006.zip › Drawings - Imagined stimulation/finger_back/Subject_10_finger_back.jpg]

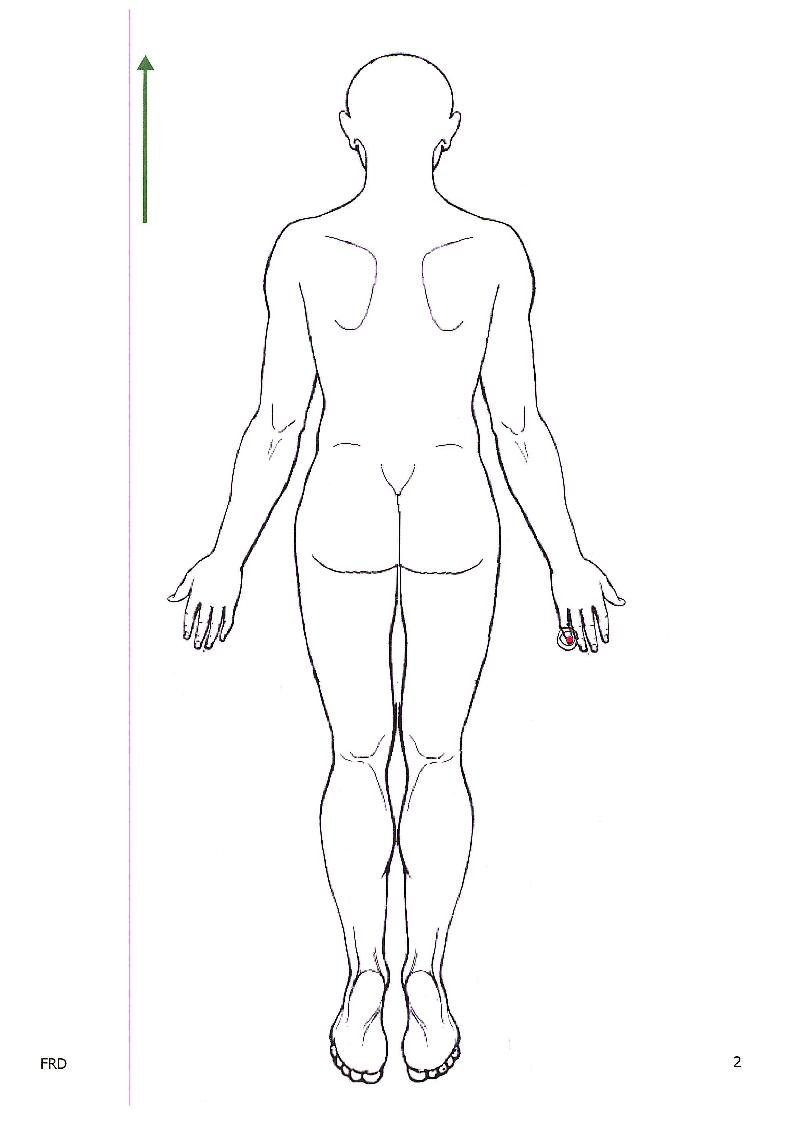

Supplement: S1 Raw Data — (ZIP) [file pone.0124808.s006.zip › Drawings - Imagined stimulation/finger_back/Subject_19_finger_back.jpg]

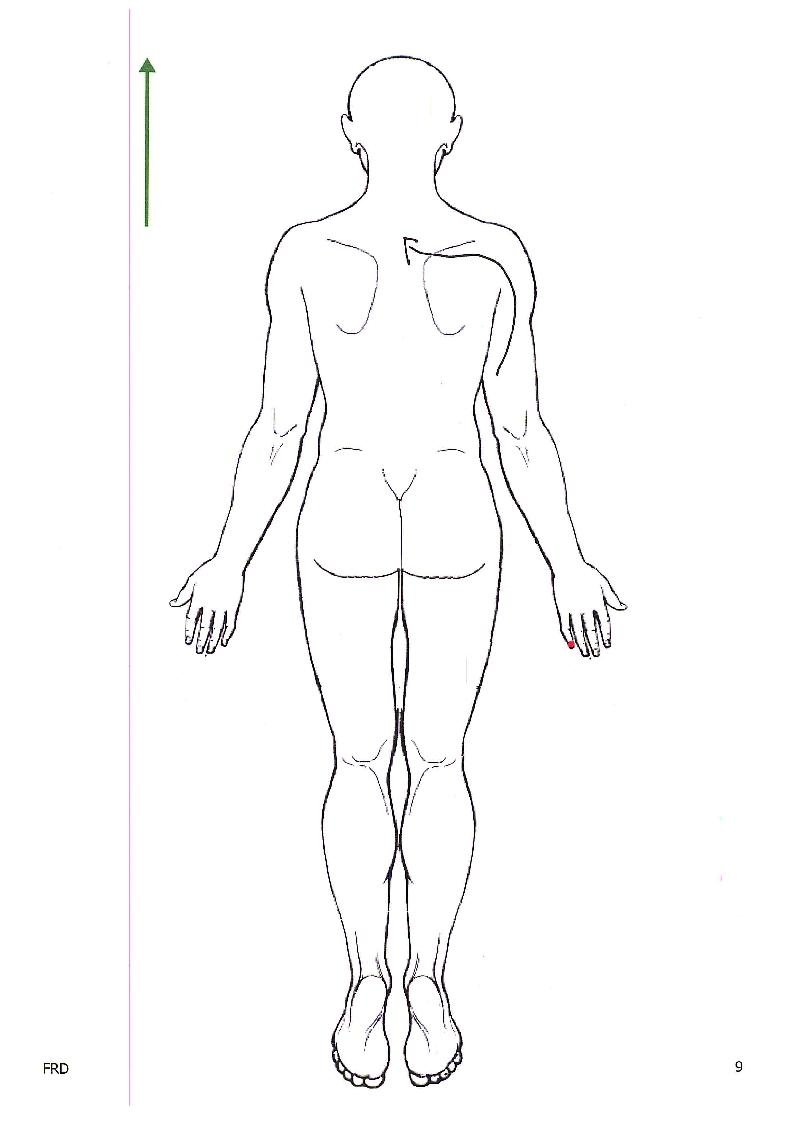

Supplement: S1 Raw Data — (ZIP) [file pone.0124808.s006.zip › Drawings - Imagined stimulation/finger_back/Subject_2_finger_back.jpg]

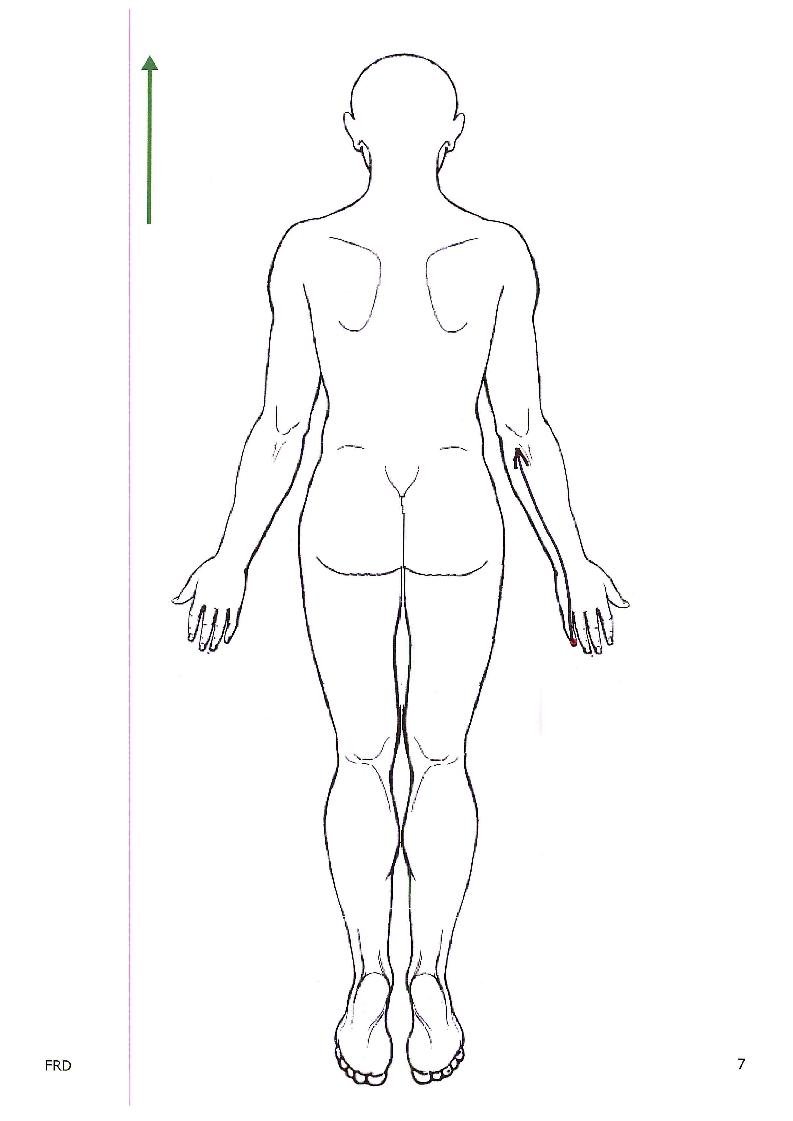

Supplement: S1 Raw Data — (ZIP) [file pone.0124808.s006.zip › Drawings - Imagined stimulation/finger_back/Subject_8_finger_back.jpg]

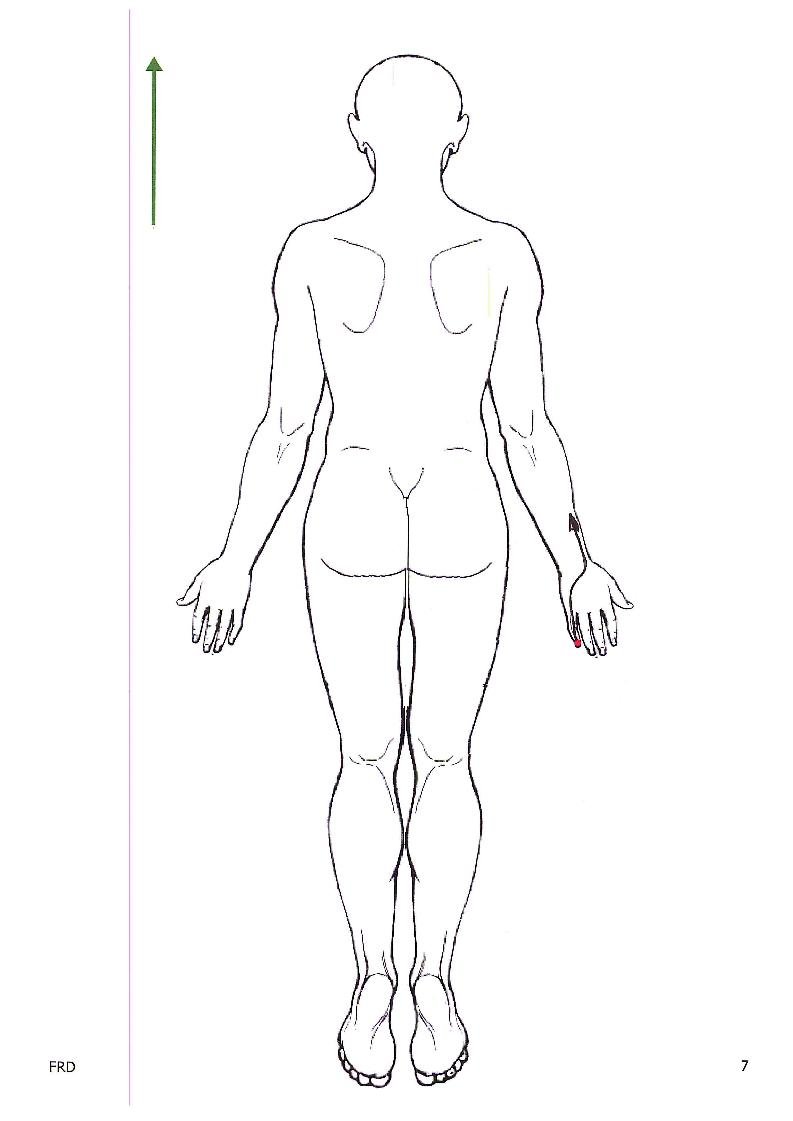

Supplement: S1 Raw Data — (ZIP) [file pone.0124808.s006.zip › Drawings - Imagined stimulation/finger_back/Subject_20_finger_back.jpg]

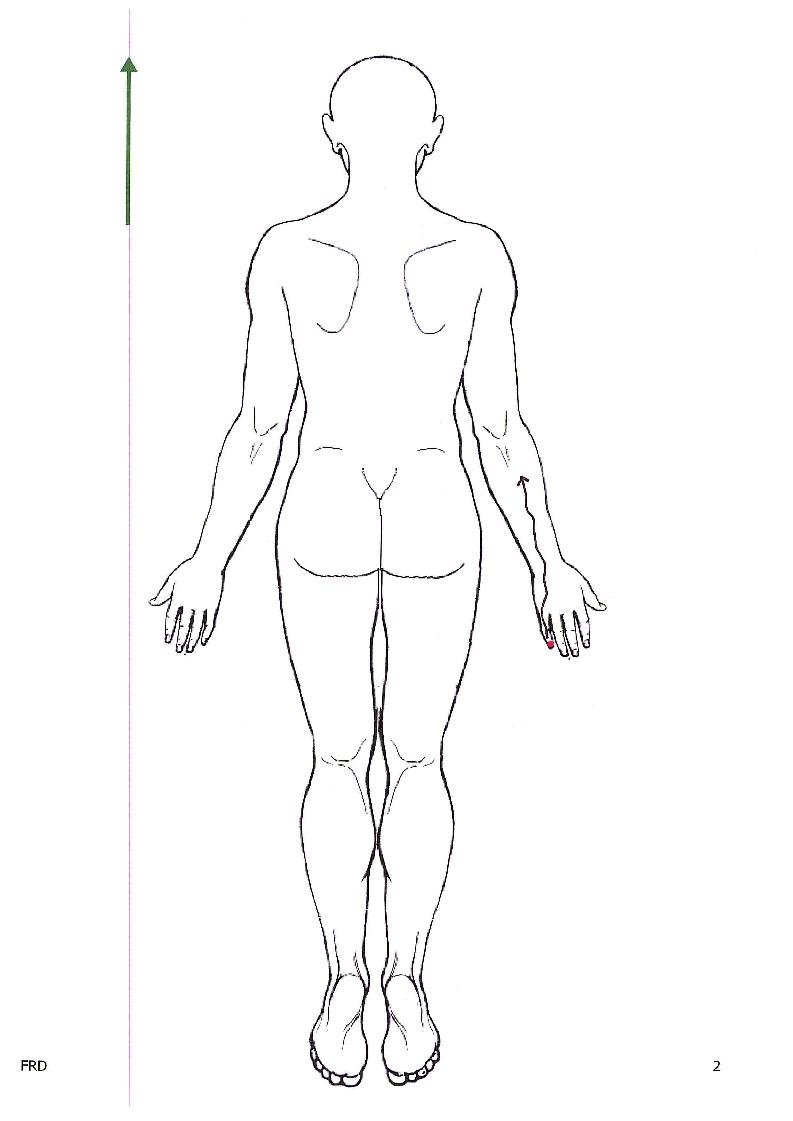

Supplement: S1 Raw Data — (ZIP) [file pone.0124808.s006.zip › Drawings - Imagined stimulation/finger_back/Subject_29_finger_back.jpg]

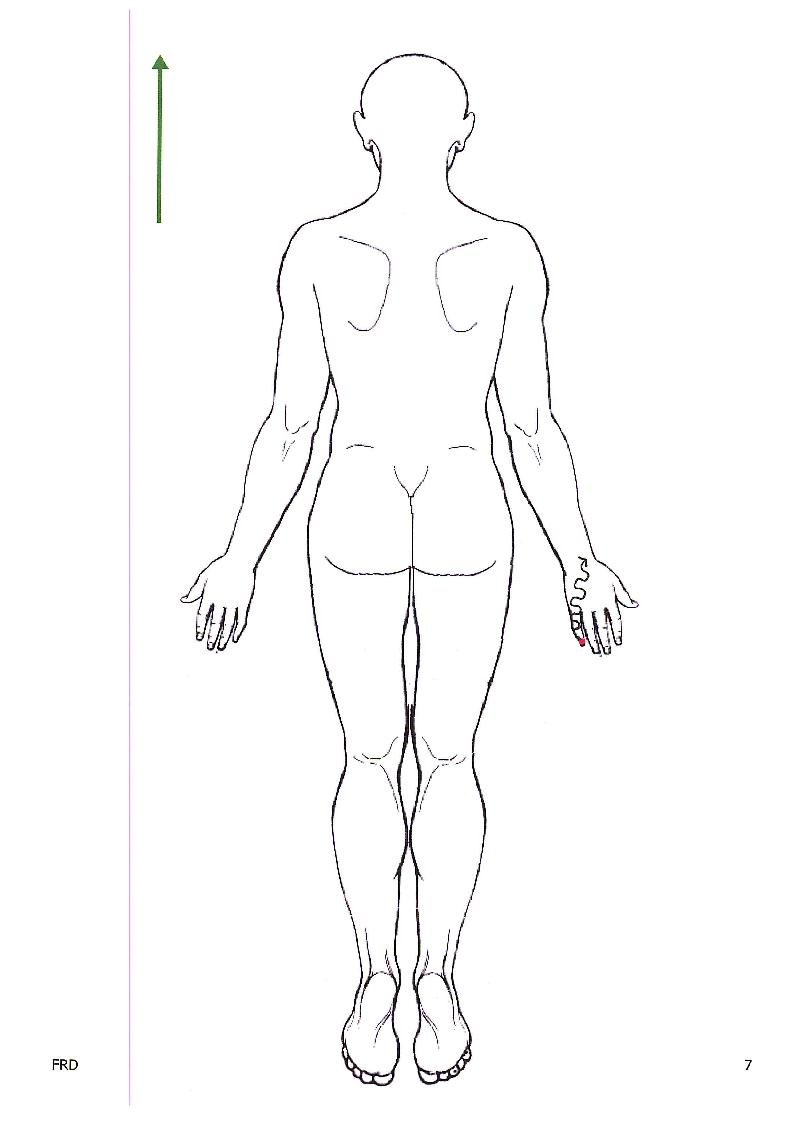

Supplement: S1 Raw Data — (ZIP) [file pone.0124808.s006.zip › Drawings - Imagined stimulation/finger_back/Subject_33_finger_back.jpg]

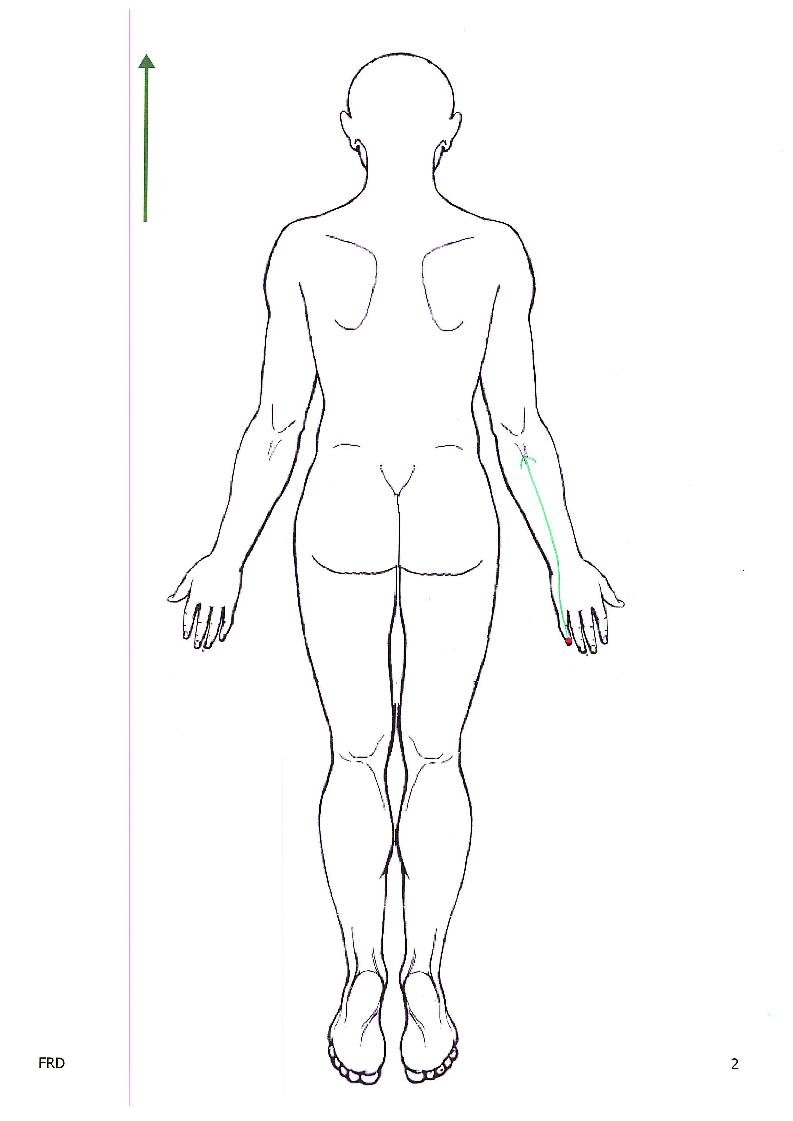

Supplement: S1 Raw Data — (ZIP) [file pone.0124808.s006.zip › Drawings - Imagined stimulation/finger_back/Subject_30_finger_back.jpg]

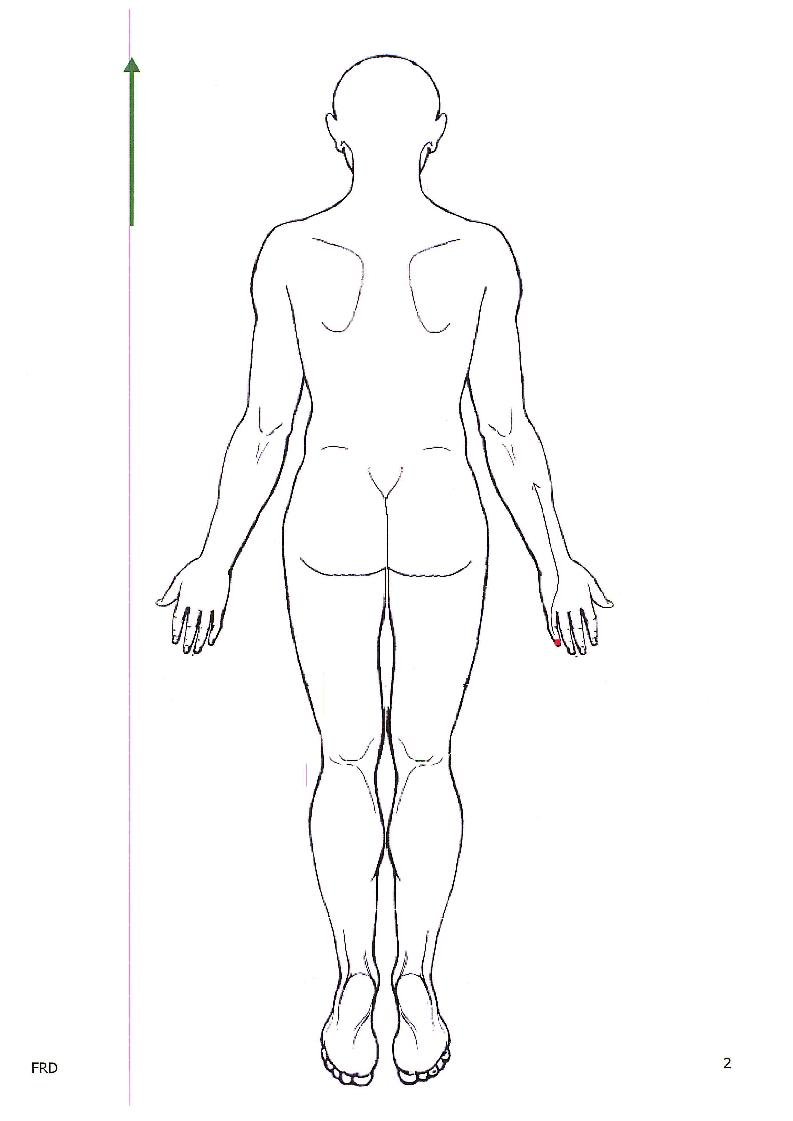

Supplement: S1 Raw Data — (ZIP) [file pone.0124808.s006.zip › Drawings - Imagined stimulation/finger_back/Subject_39_finger_back.jpg]

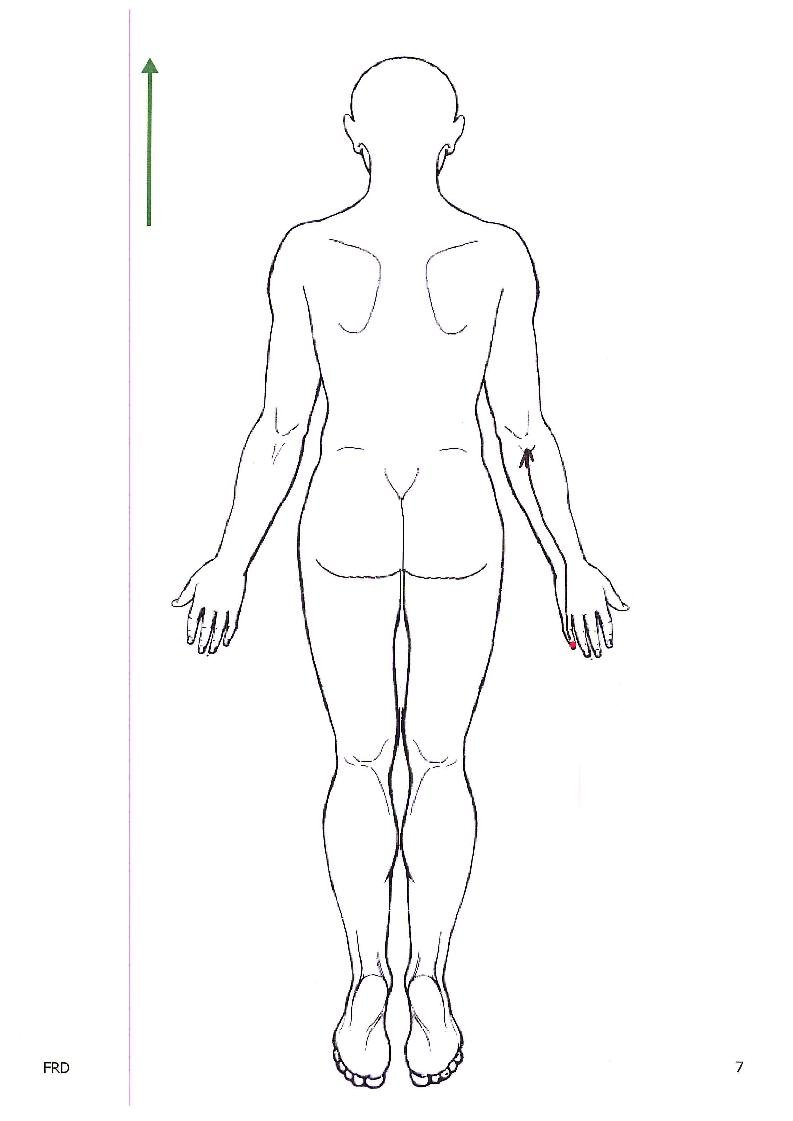

Supplement: S1 Raw Data — (ZIP) [file pone.0124808.s006.zip › Drawings - Imagined stimulation/finger_back/Subject_43_finger_back.jpg]

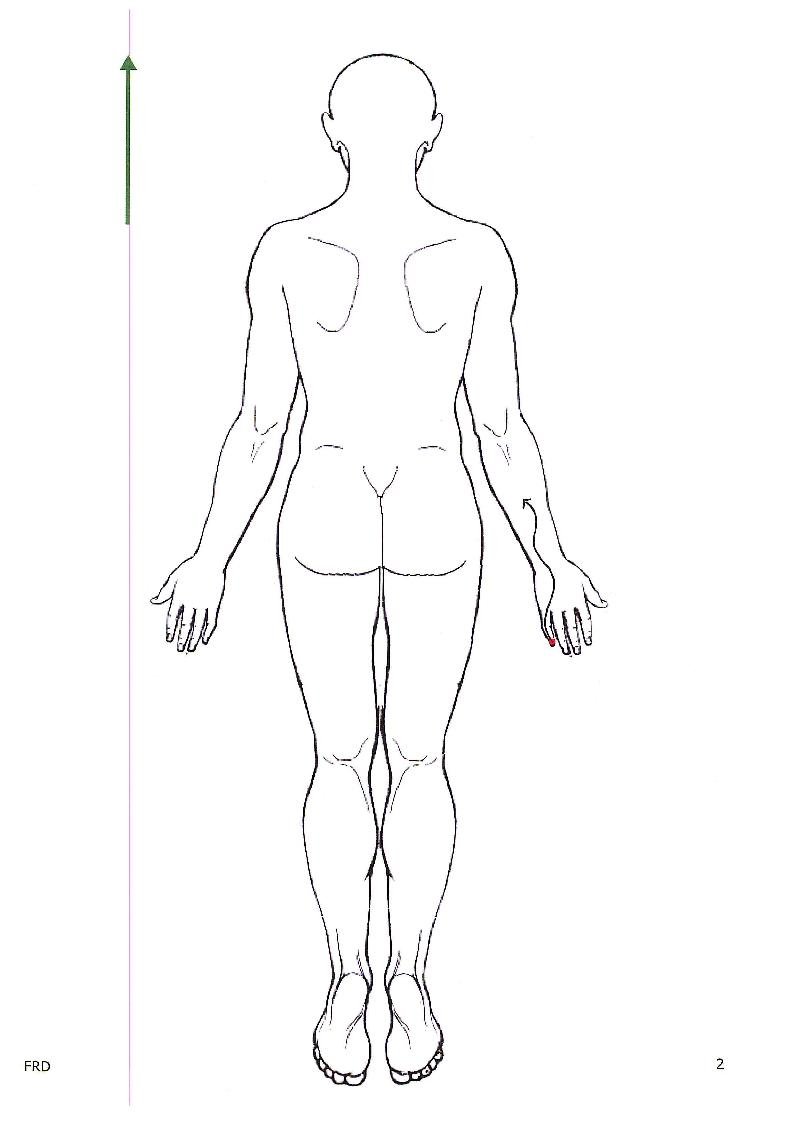

Supplement: S1 Raw Data — (ZIP) [file pone.0124808.s006.zip › Drawings - Imagined stimulation/finger_back/Subject_40_finger_back.jpg]

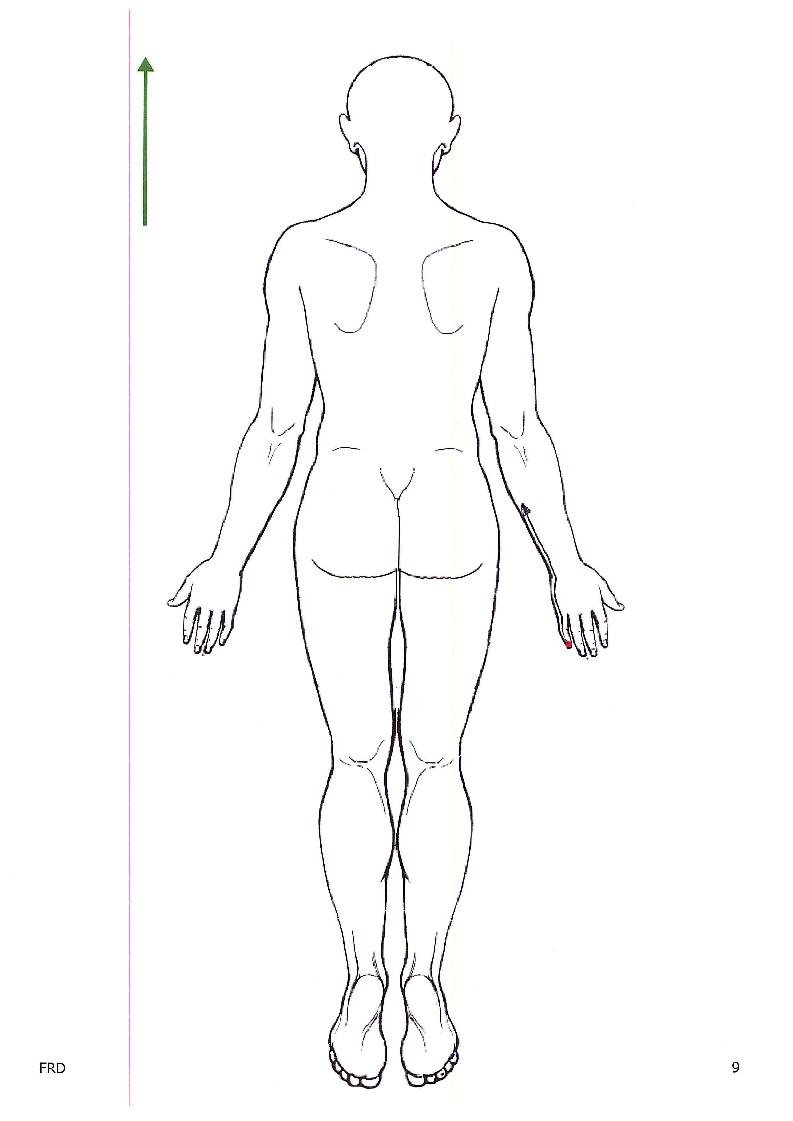

Supplement: S1 Raw Data — (ZIP) [file pone.0124808.s006.zip › Drawings - Imagined stimulation/finger_back/Subject_49_finger_back.jpg]

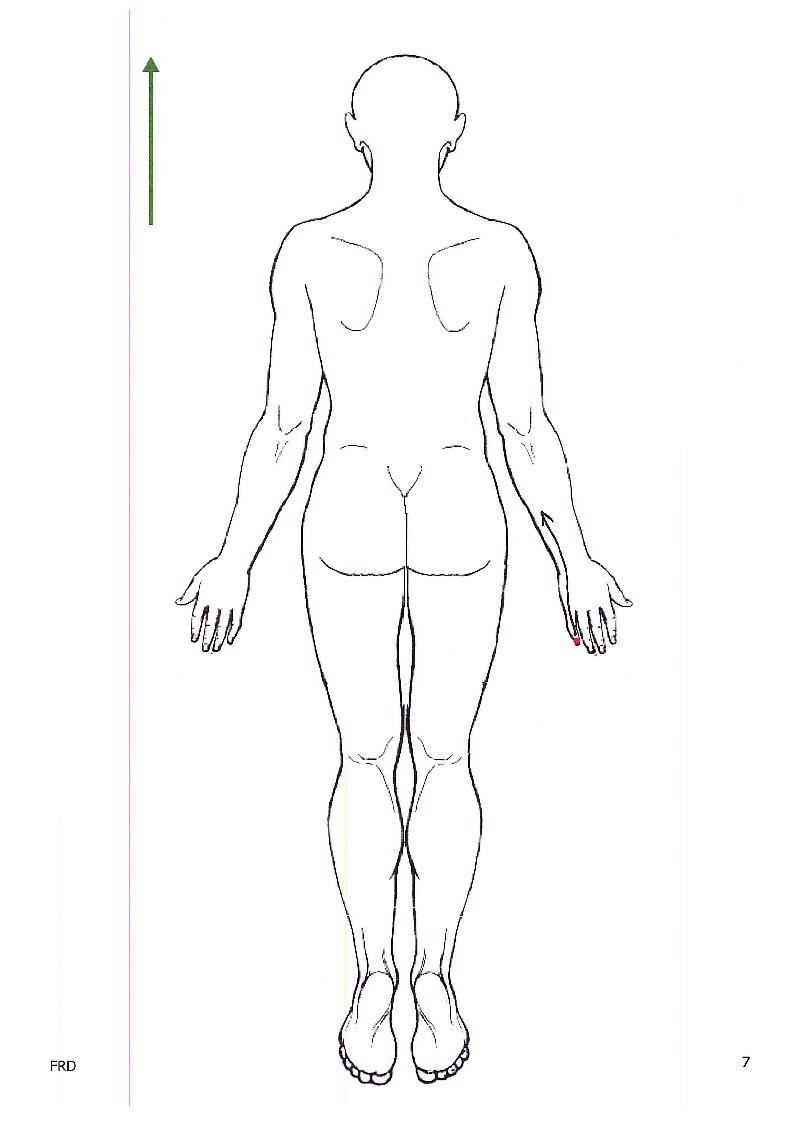

Supplement: S1 Raw Data — (ZIP) [file pone.0124808.s006.zip › Drawings - Imagined stimulation/finger_back/Subject_53_finger_back.jpg]

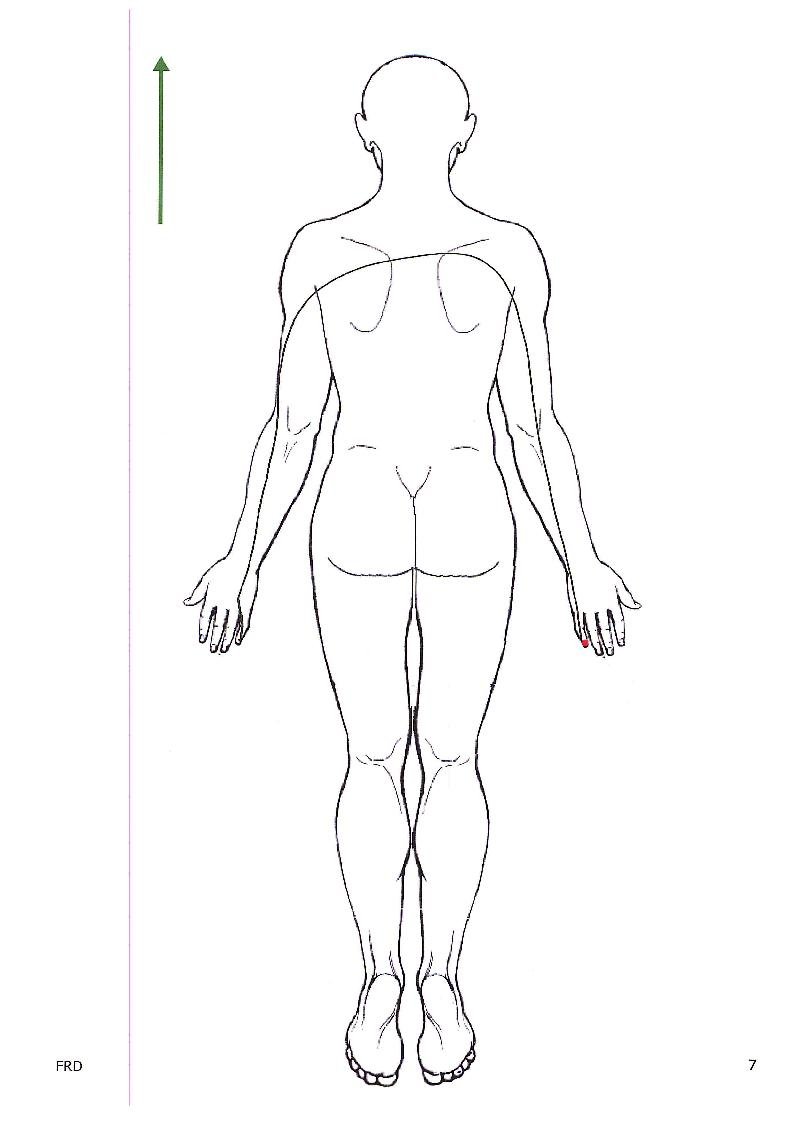

Supplement: S1 Raw Data — (ZIP) [file pone.0124808.s006.zip › Drawings - Imagined stimulation/finger_back/Subject_50_finger_back.jpg]

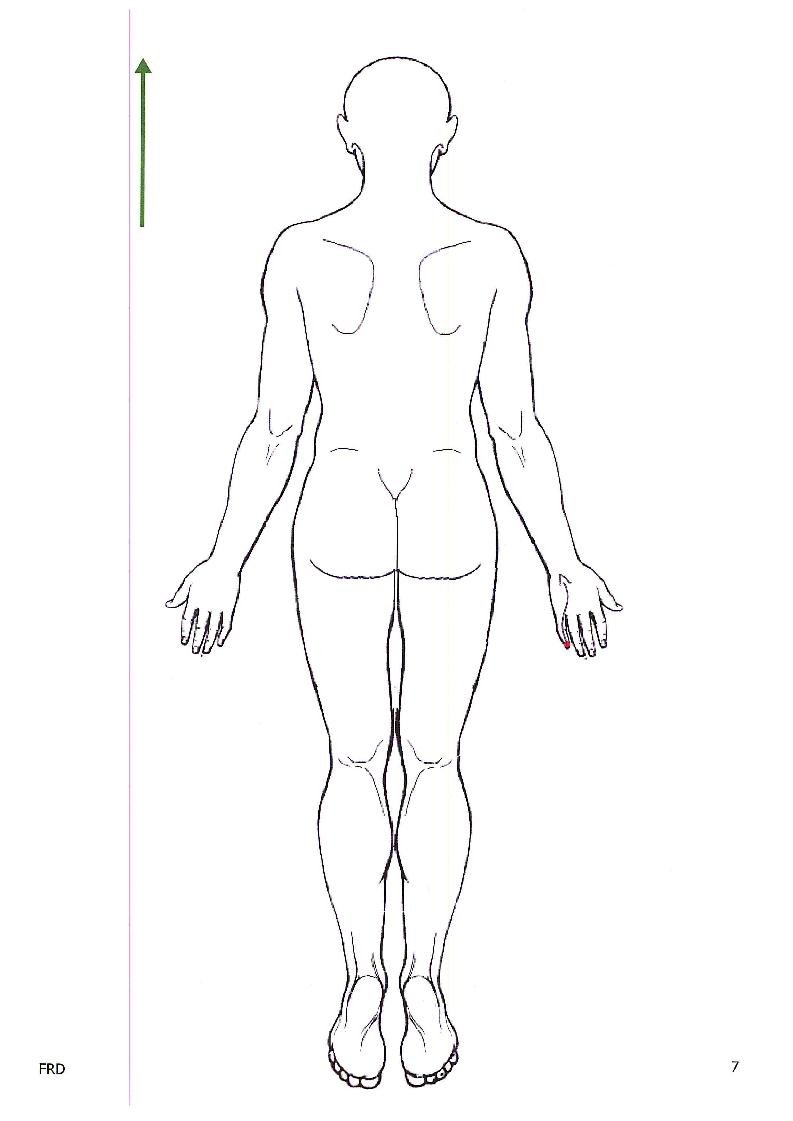

Supplement: S1 Raw Data — (ZIP) [file pone.0124808.s006.zip › Drawings - Imagined stimulation/finger_back/Subject_1_finger_back.jpg]

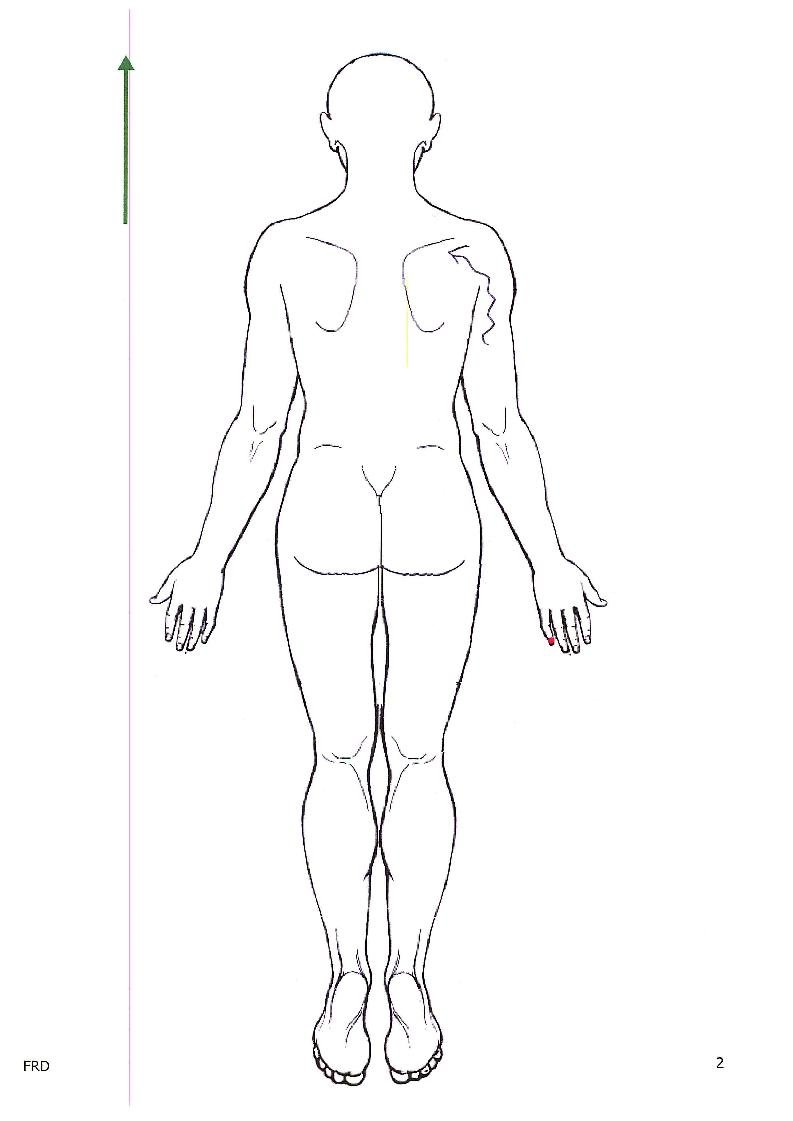

Supplement: S1 Raw Data — (ZIP) [file pone.0124808.s006.zip › Drawings - Imagined stimulation/finger_back/Subject_12_finger_back.jpg]

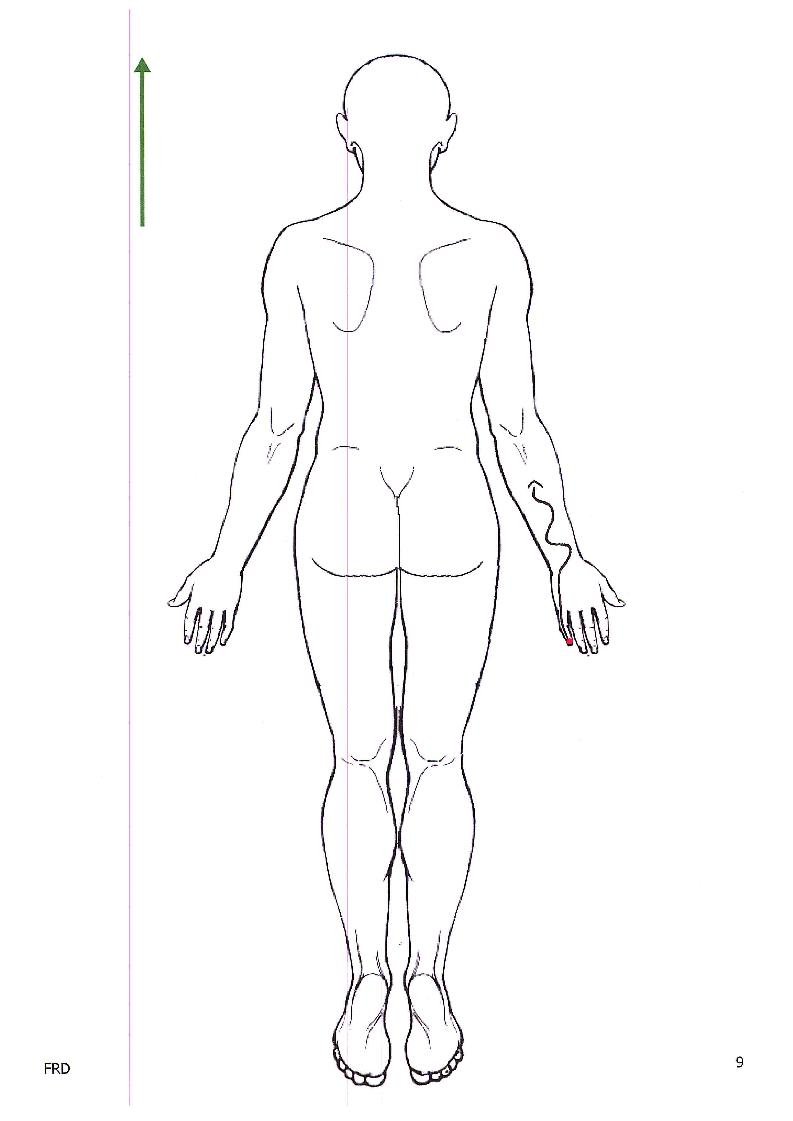

Supplement: S1 Raw Data — (ZIP) [file pone.0124808.s006.zip › Drawings - Imagined stimulation/finger_back/Subject_22_finger_back.jpg]

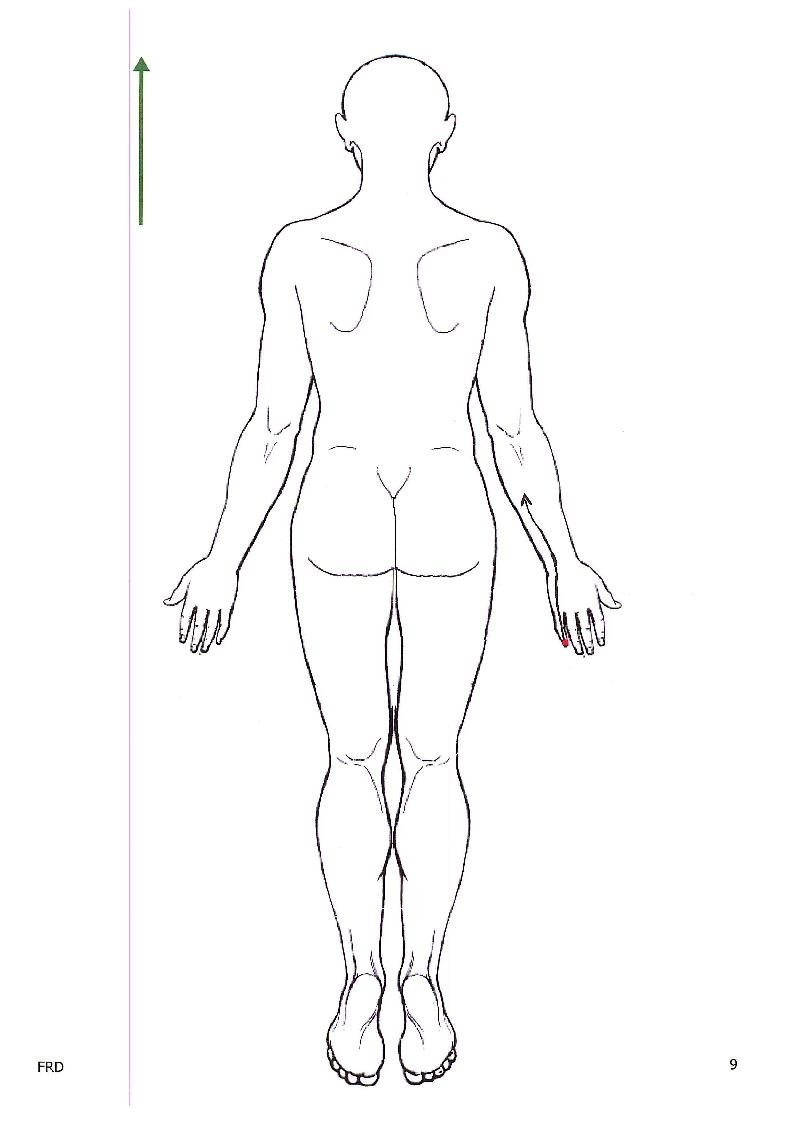

Supplement: S1 Raw Data — (ZIP) [file pone.0124808.s006.zip › Drawings - Imagined stimulation/finger_back/Subject_32_finger_back.jpg]

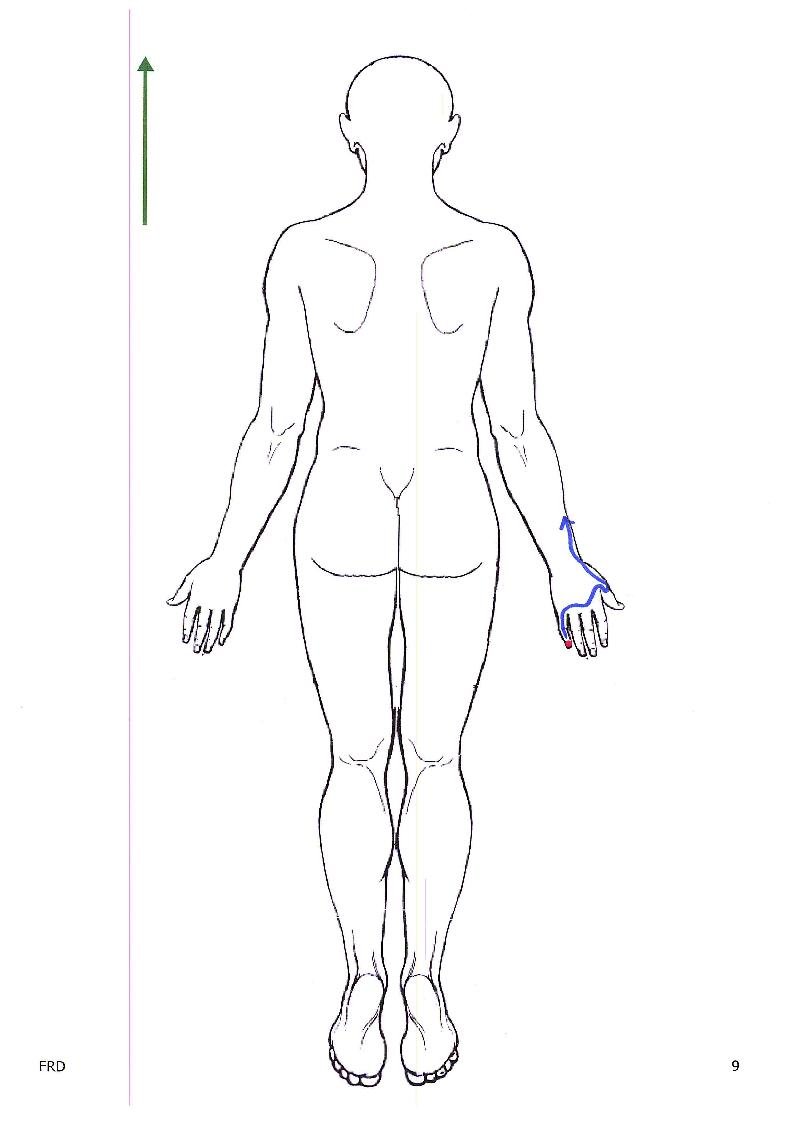

Supplement: S1 Raw Data — (ZIP) [file pone.0124808.s006.zip › Drawings - Imagined stimulation/finger_back/Subject_42_finger_back.jpg]

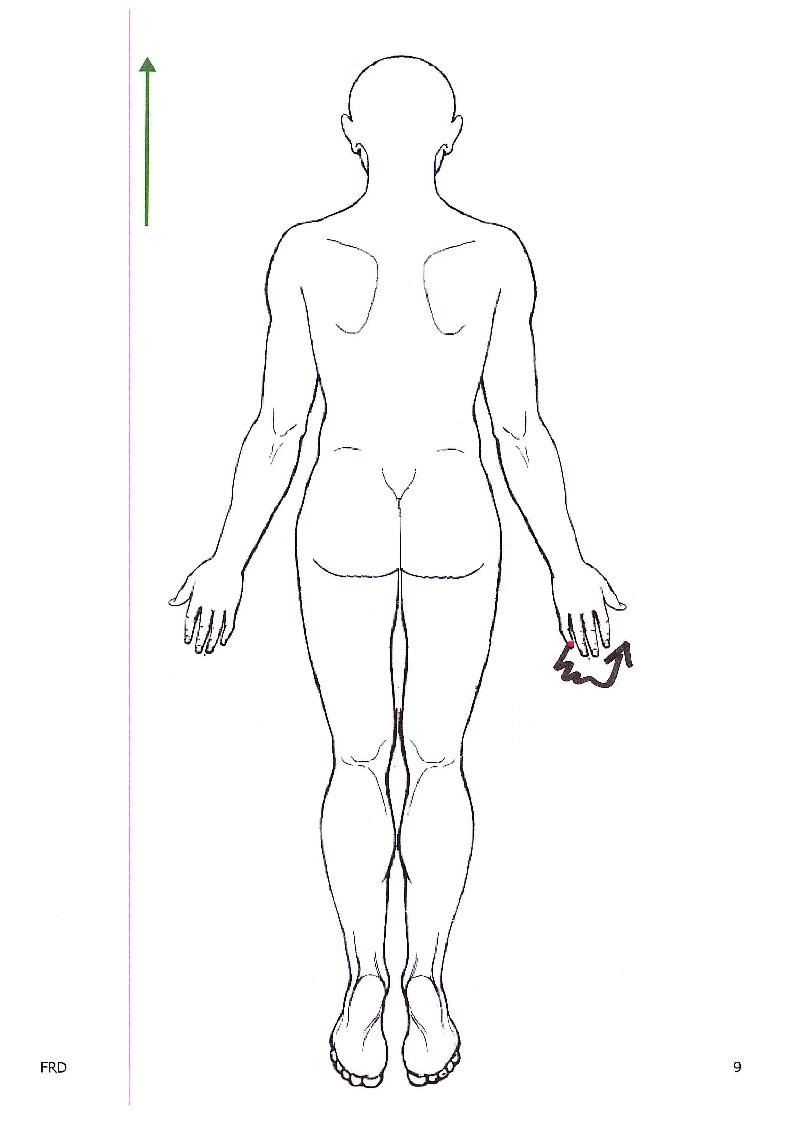

Supplement: S1 Raw Data — (ZIP) [file pone.0124808.s006.zip › Drawings - Imagined stimulation/finger_back/Subject_55_finger_back.jpg]

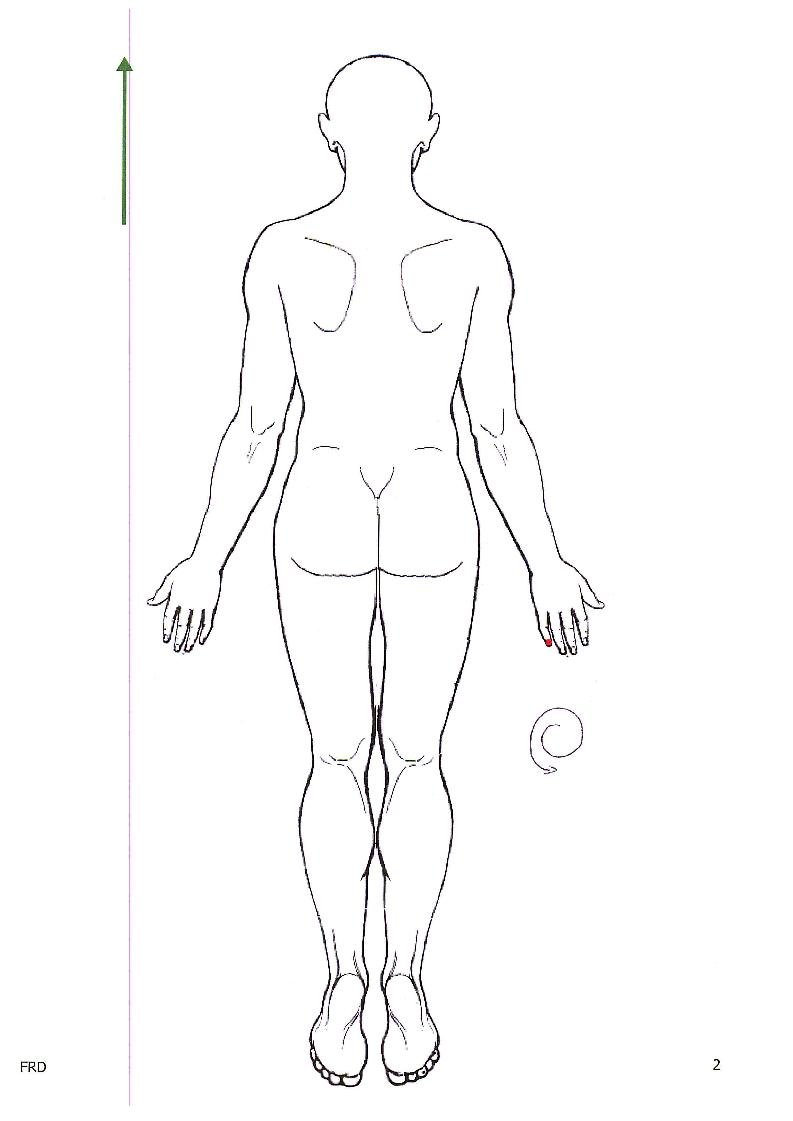

Supplement: S1 Raw Data — (ZIP) [file pone.0124808.s006.zip › Drawings - Imagined stimulation/finger_back/Subject_52_finger_back.jpg]

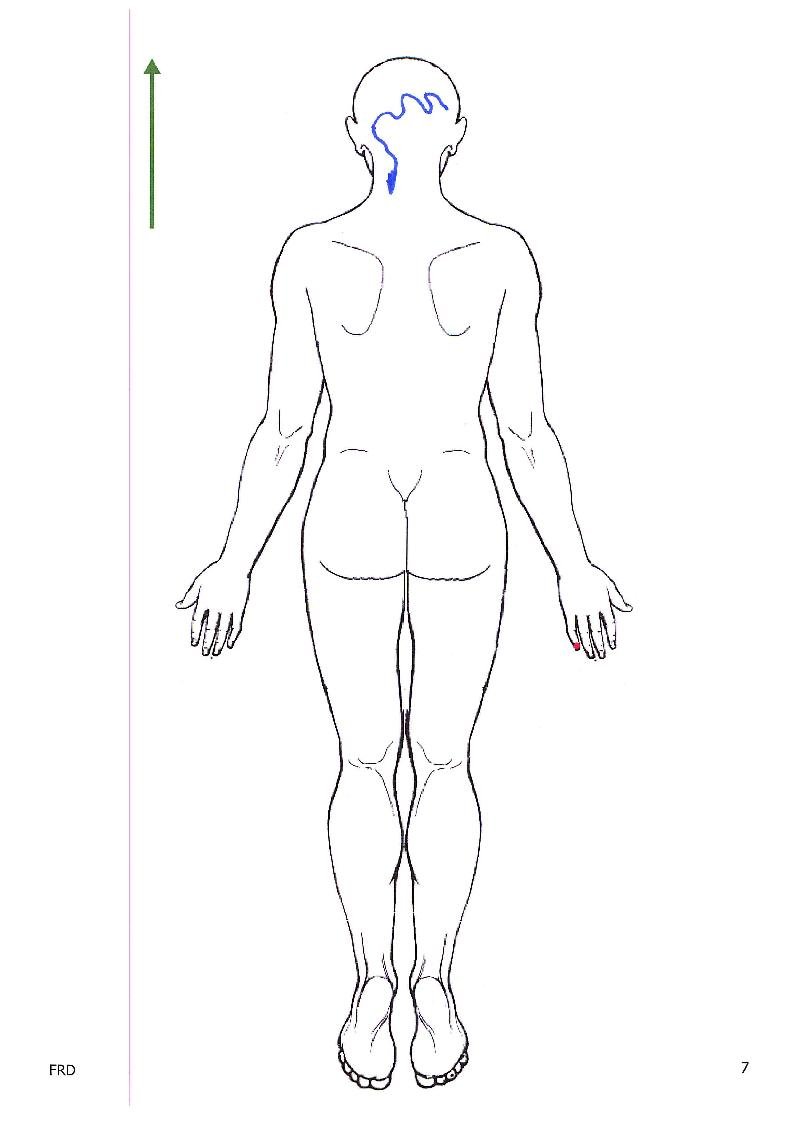

Supplement: S1 Raw Data — (ZIP) [file pone.0124808.s006.zip › Drawings - Imagined stimulation/finger_back/Subject_14_finger_back.jpg]

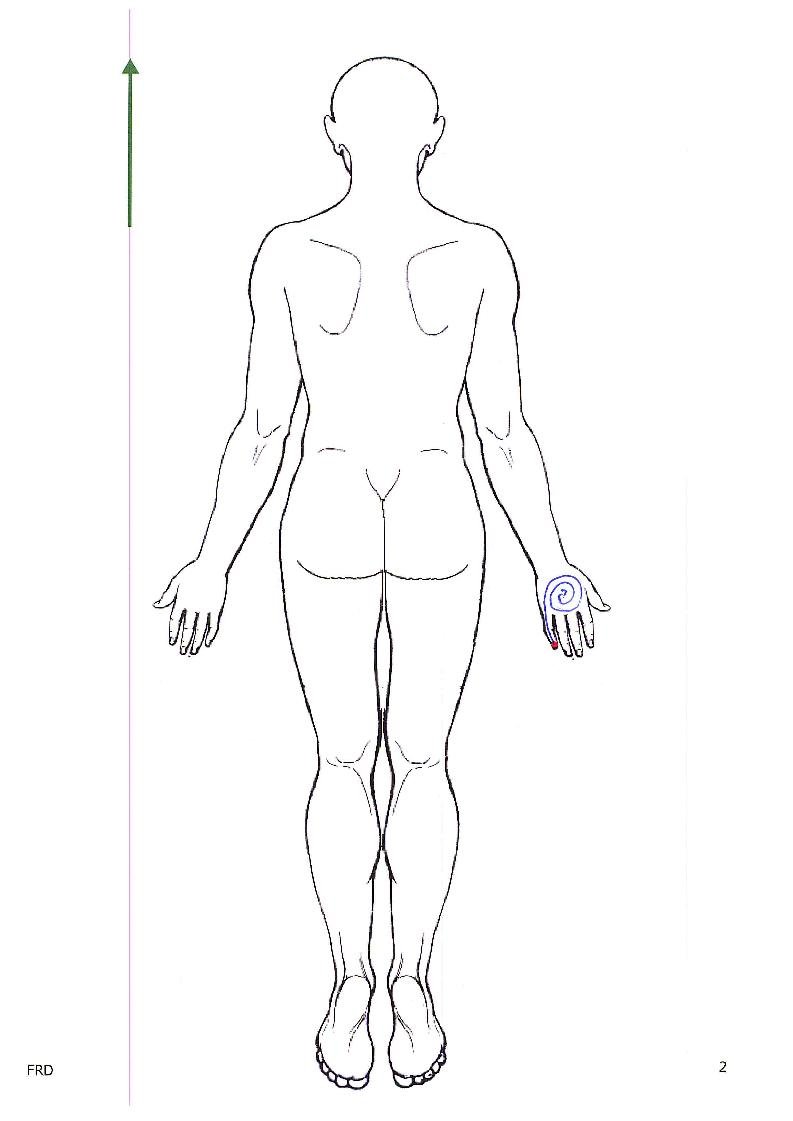

Supplement: S1 Raw Data — (ZIP) [file pone.0124808.s006.zip › Drawings - Imagined stimulation/finger_back/Subject_3_finger_back.jpg]

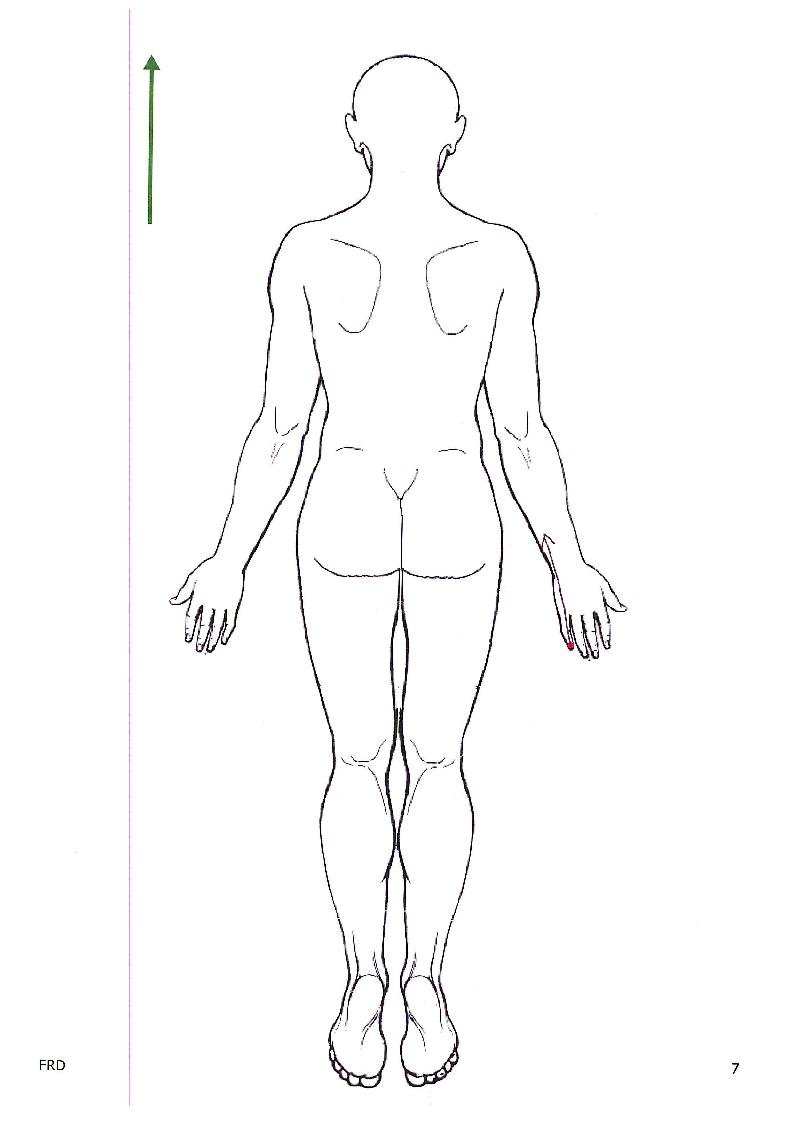

Supplement: S1 Raw Data — (ZIP) [file pone.0124808.s006.zip › Drawings - Imagined stimulation/finger_back/Subject_24_finger_back.jpg]

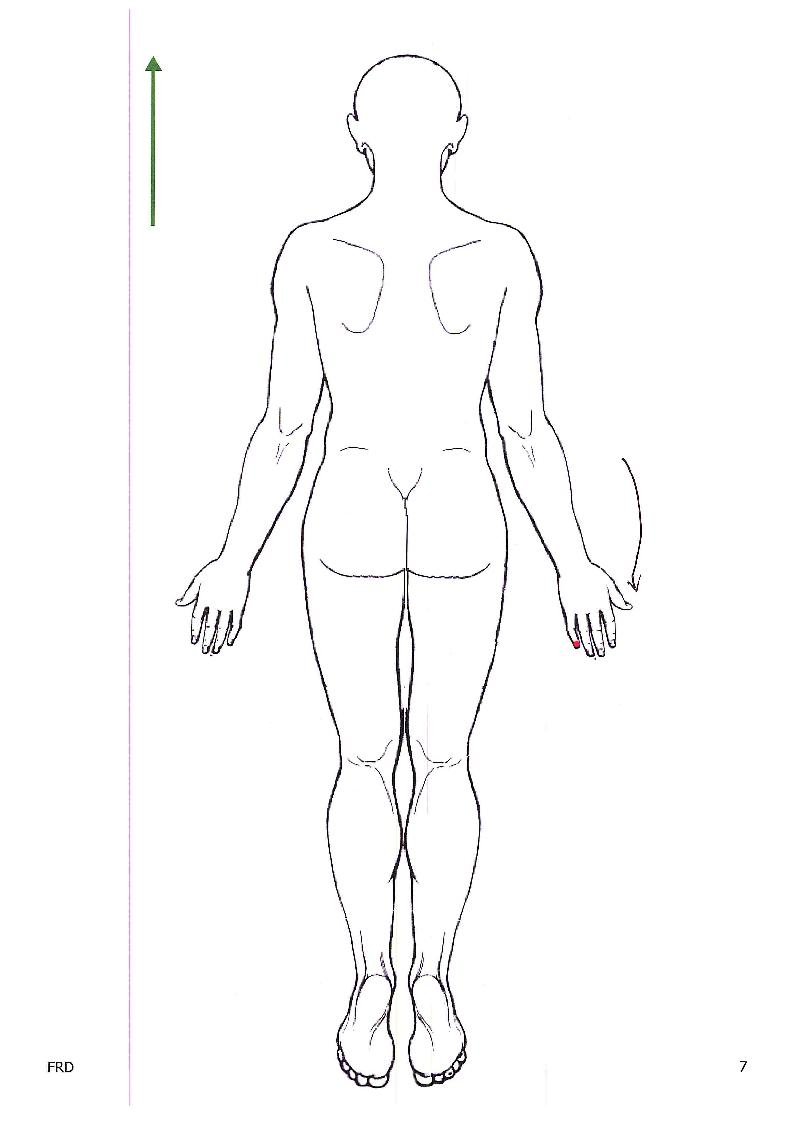

Supplement: S1 Raw Data — (ZIP) [file pone.0124808.s006.zip › Drawings - Imagined stimulation/finger_back/Subject_34_finger_back.jpg]

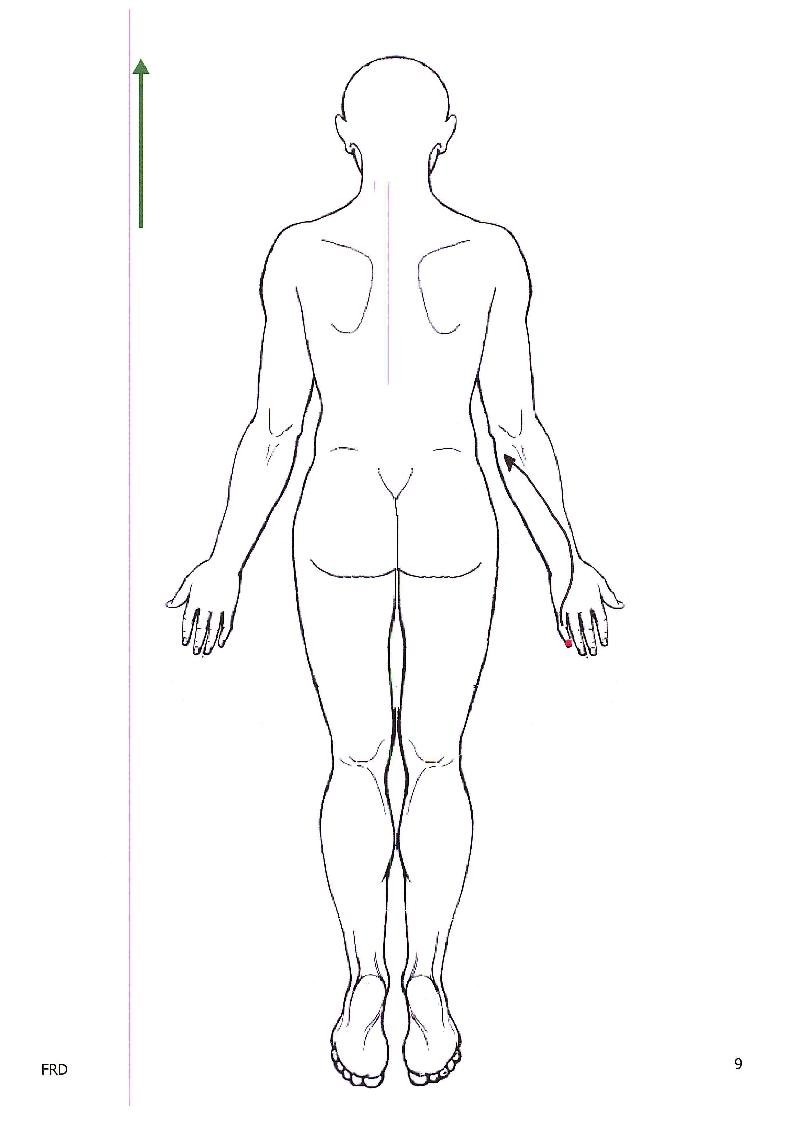

Supplement: S1 Raw Data — (ZIP) [file pone.0124808.s006.zip › Drawings - Imagined stimulation/finger_back/Subject_44_finger_back.jpg]

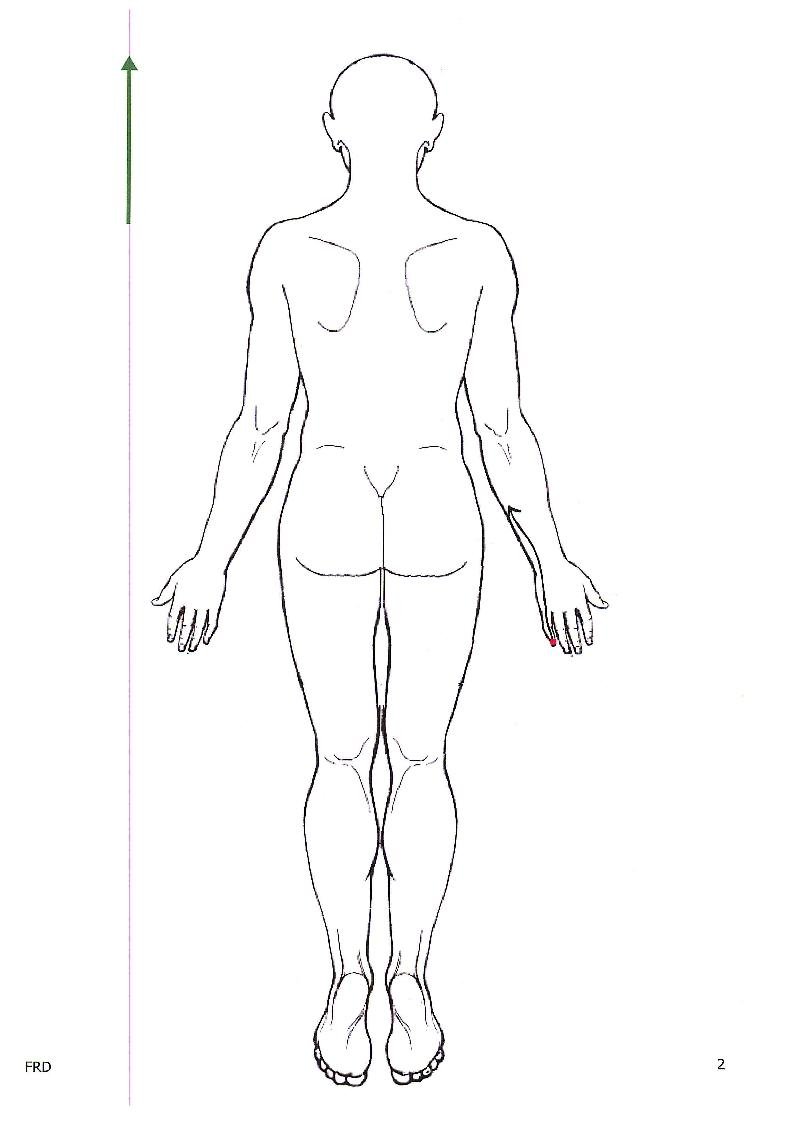

Supplement: S1 Raw Data — (ZIP) [file pone.0124808.s006.zip › Drawings - Imagined stimulation/finger_back/Subject_54_finger_back.jpg]

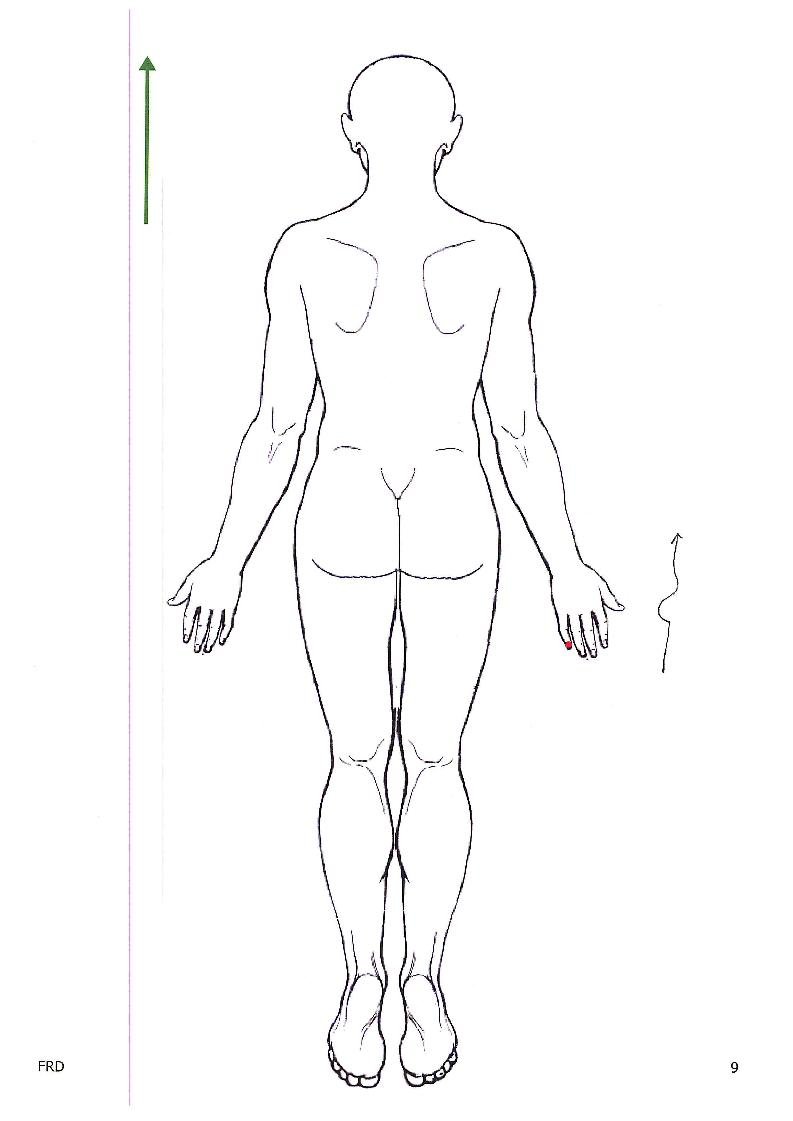

Supplement: S1 Raw Data — (ZIP) [file pone.0124808.s006.zip › Drawings - Imagined stimulation/finger_back/Subject_16_finger_back.jpg]

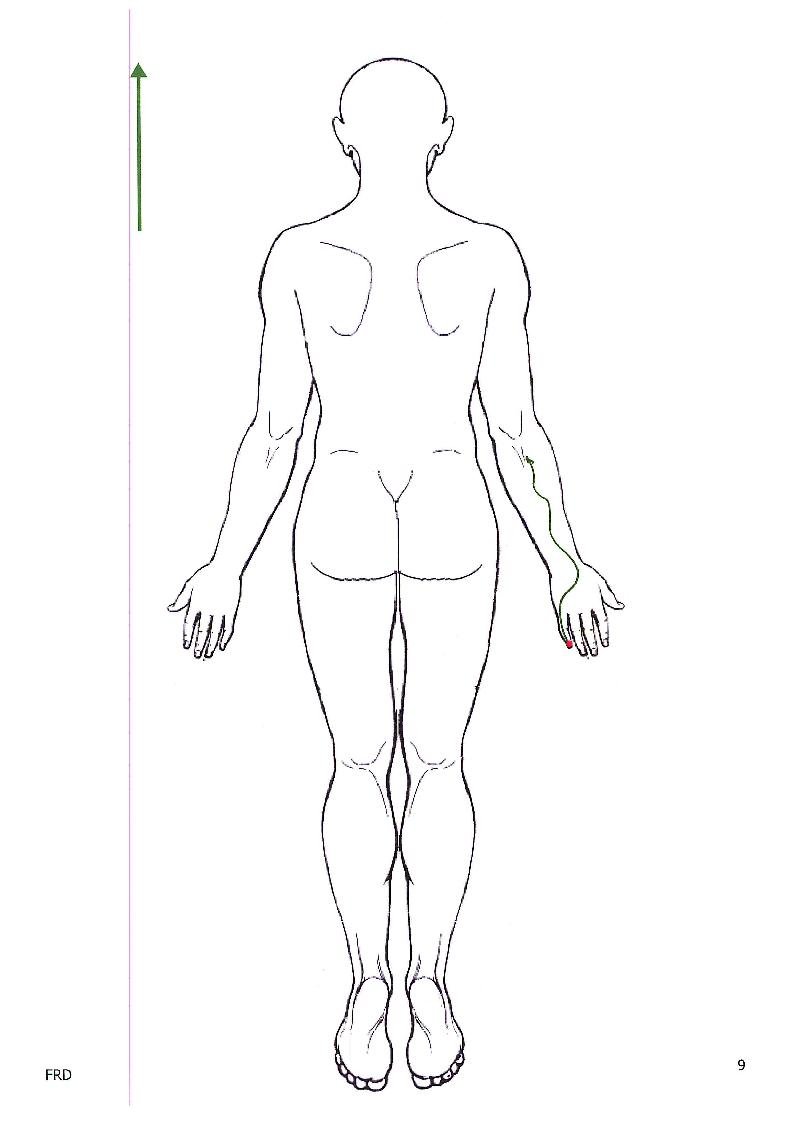

Supplement: S1 Raw Data — (ZIP) [file pone.0124808.s006.zip › Drawings - Imagined stimulation/finger_back/Subject_13_finger_back.jpg]

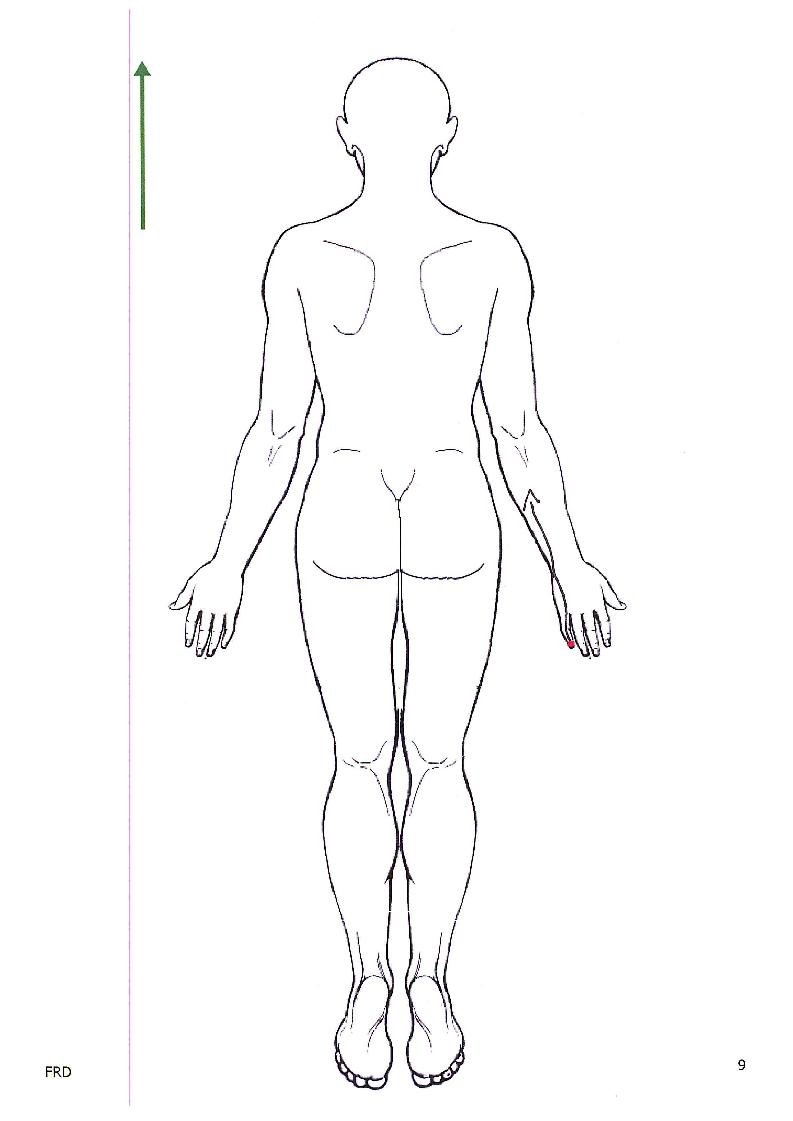

Supplement: S1 Raw Data — (ZIP) [file pone.0124808.s006.zip › Drawings - Imagined stimulation/finger_back/Subject_5_finger_back.jpg]

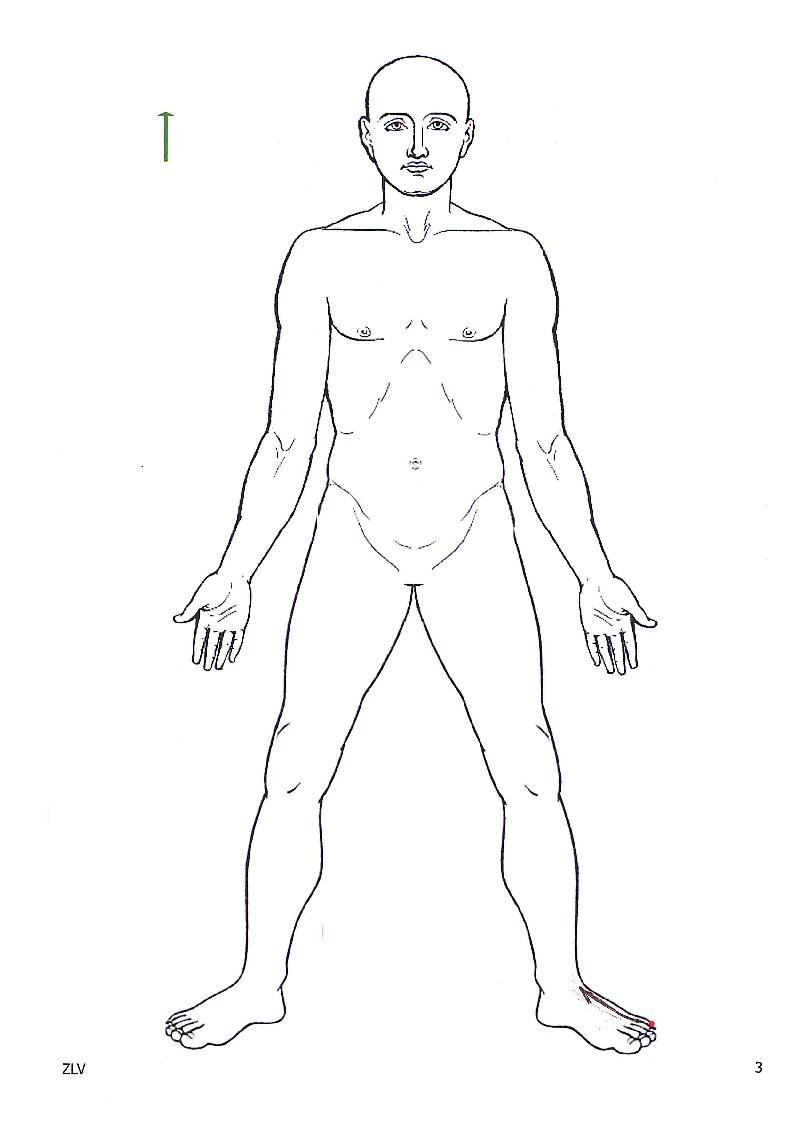

Supplement: S1 Raw Data — (ZIP) [file pone.0124808.s006.zip › Drawings - Imagined stimulation/toe_front/Subject_18_toe_front.jpg]

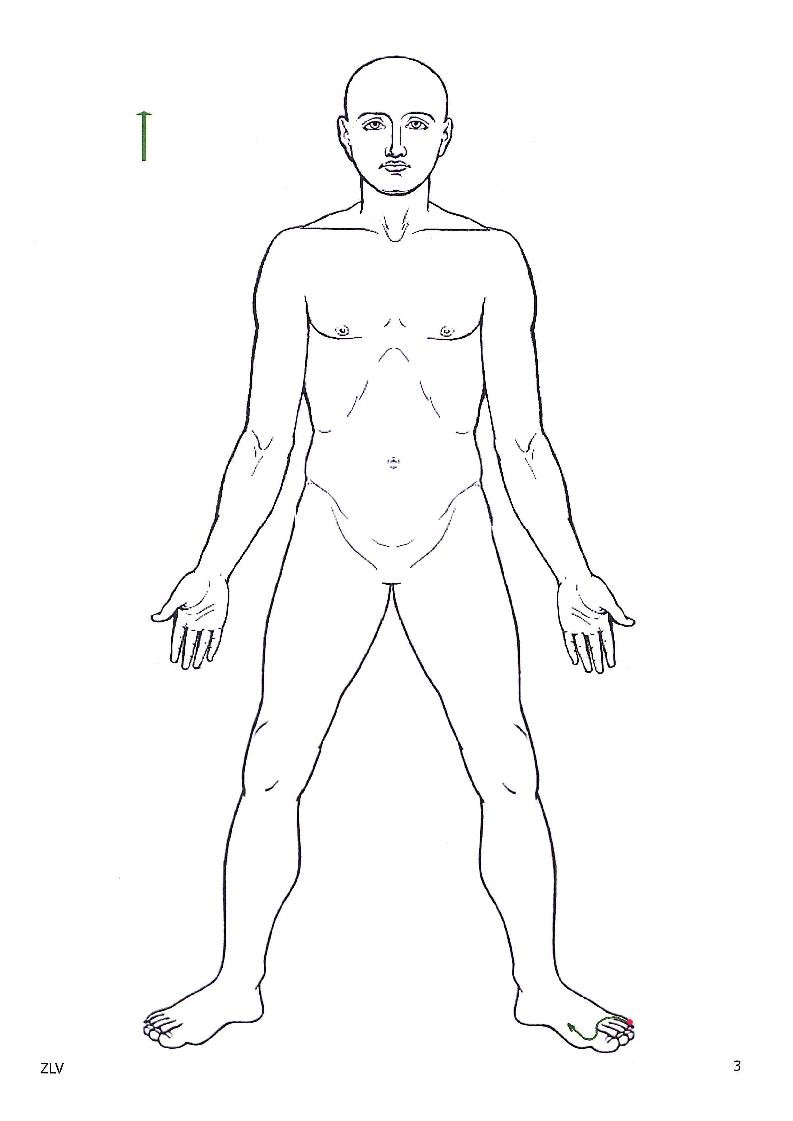

Supplement: S1 Raw Data — (ZIP) [file pone.0124808.s006.zip › Drawings - Imagined stimulation/toe_front/Subject_13_toe_front.jpg]

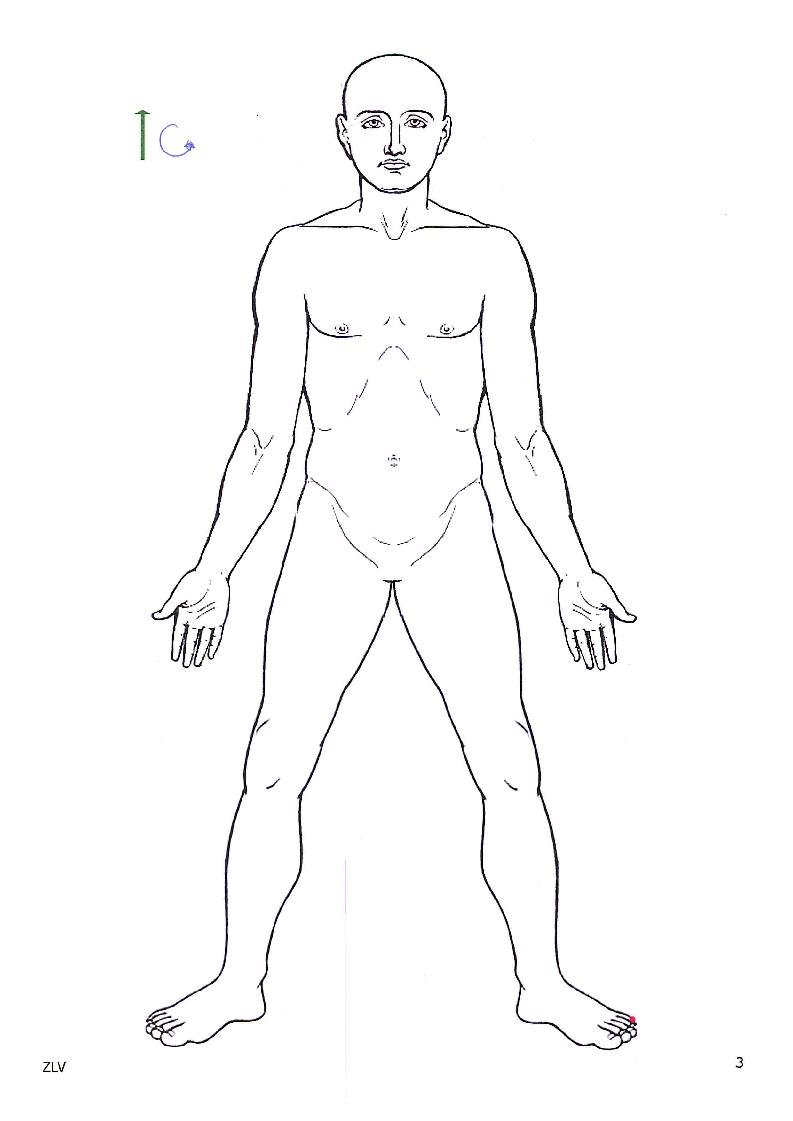

Supplement: S1 Raw Data — (ZIP) [file pone.0124808.s006.zip › Drawings - Imagined stimulation/toe_front/Subject_21_toe_front.jpg]

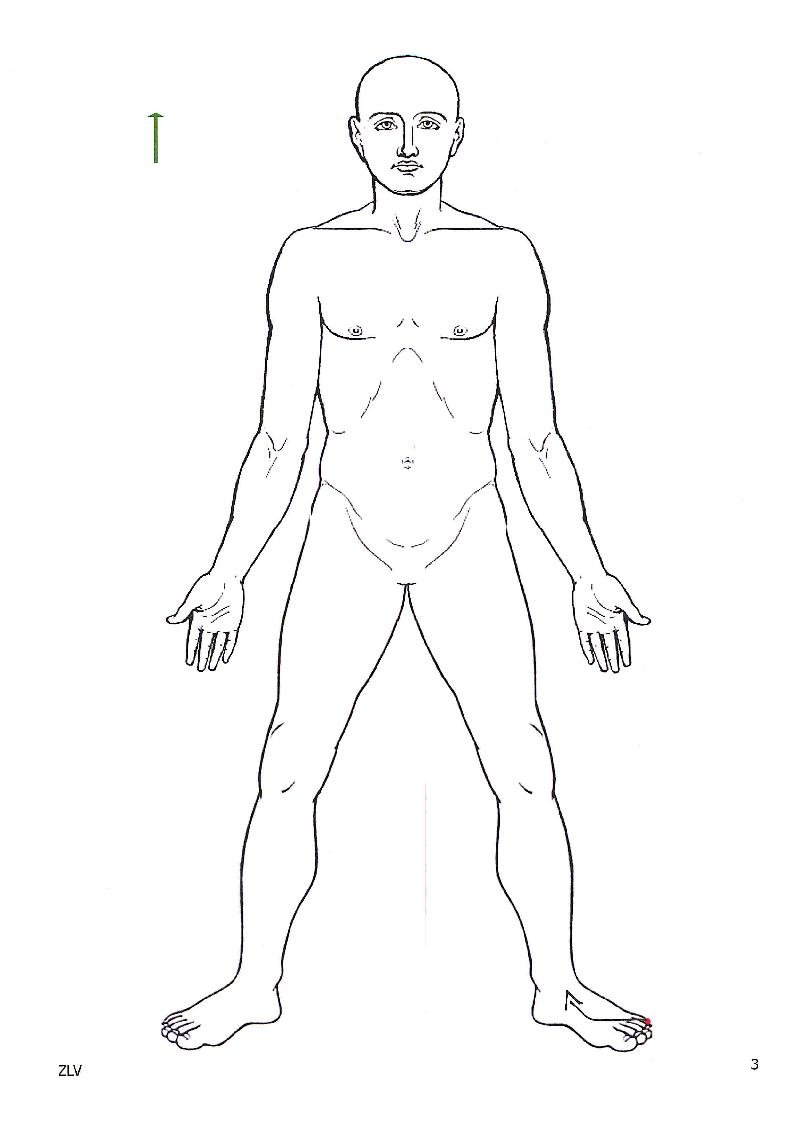

Supplement: S1 Raw Data — (ZIP) [file pone.0124808.s006.zip › Drawings - Imagined stimulation/toe_front/Subject_2_toe_front.jpg]

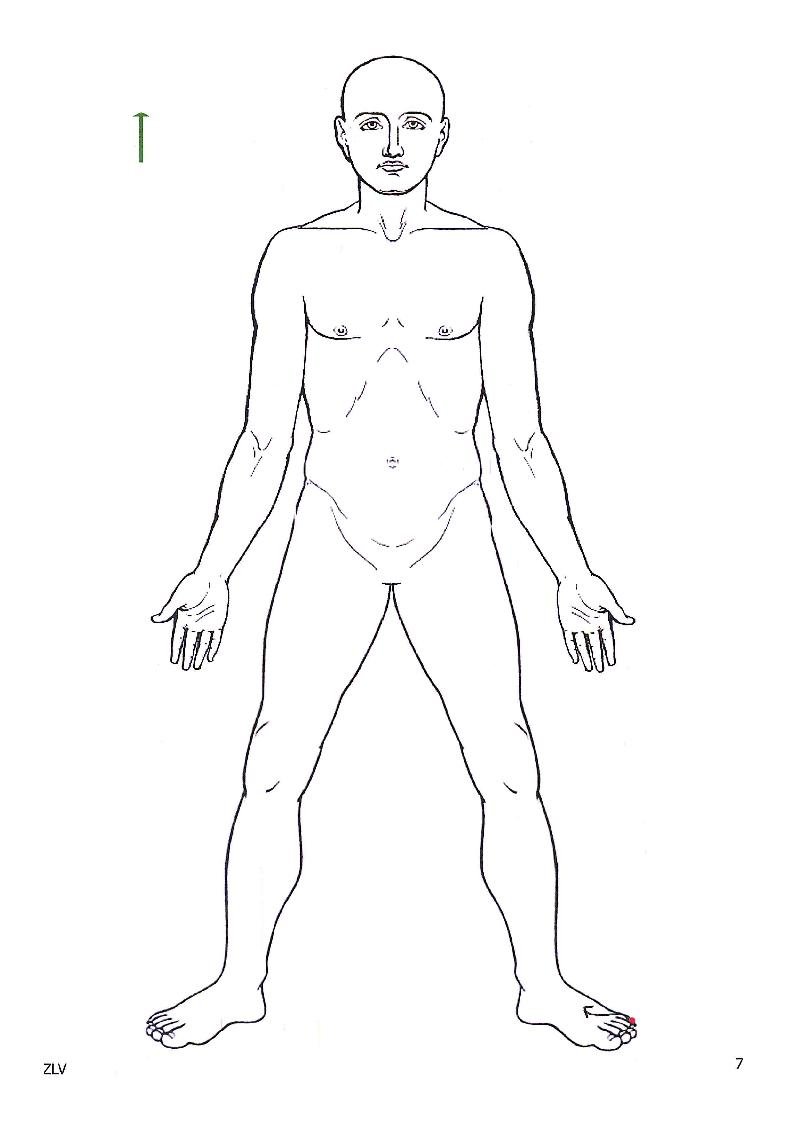

Supplement: S1 Raw Data — (ZIP) [file pone.0124808.s006.zip › Drawings - Imagined stimulation/toe_front/Subject_29_toe_front.jpg]

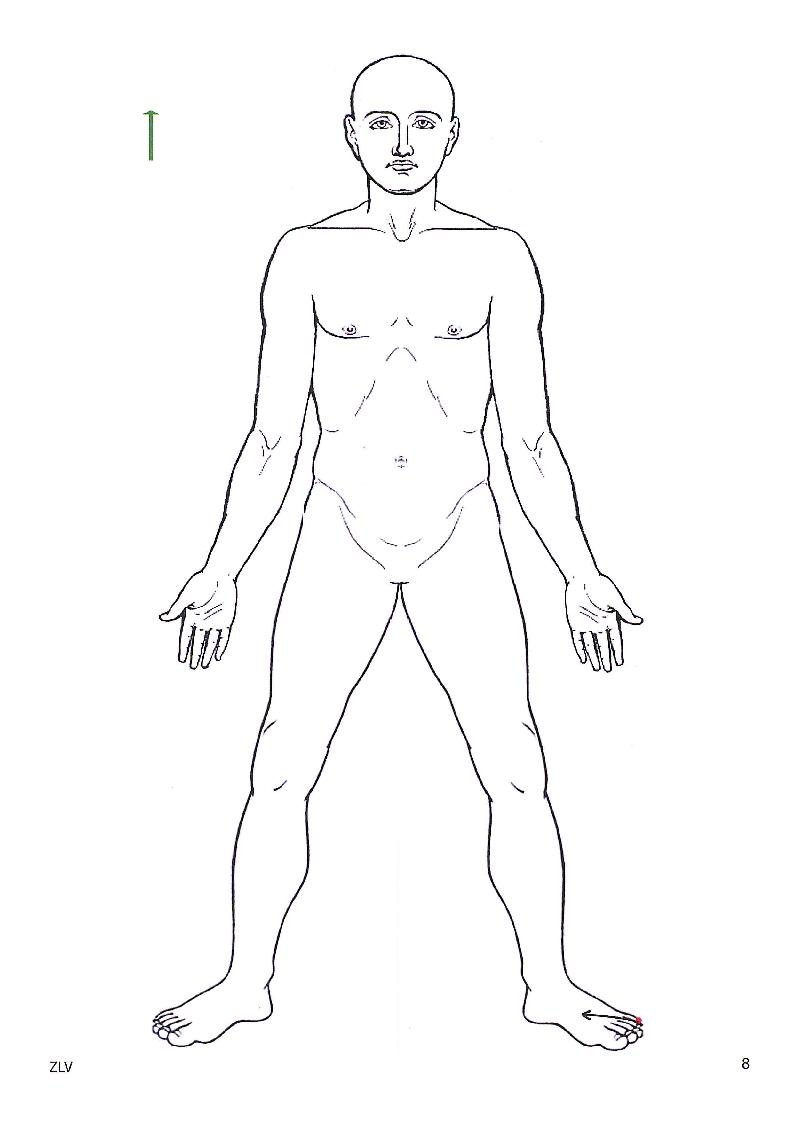

Supplement: S1 Raw Data — (ZIP) [file pone.0124808.s006.zip › Drawings - Imagined stimulation/toe_front/Subject_37_toe_front.jpg]

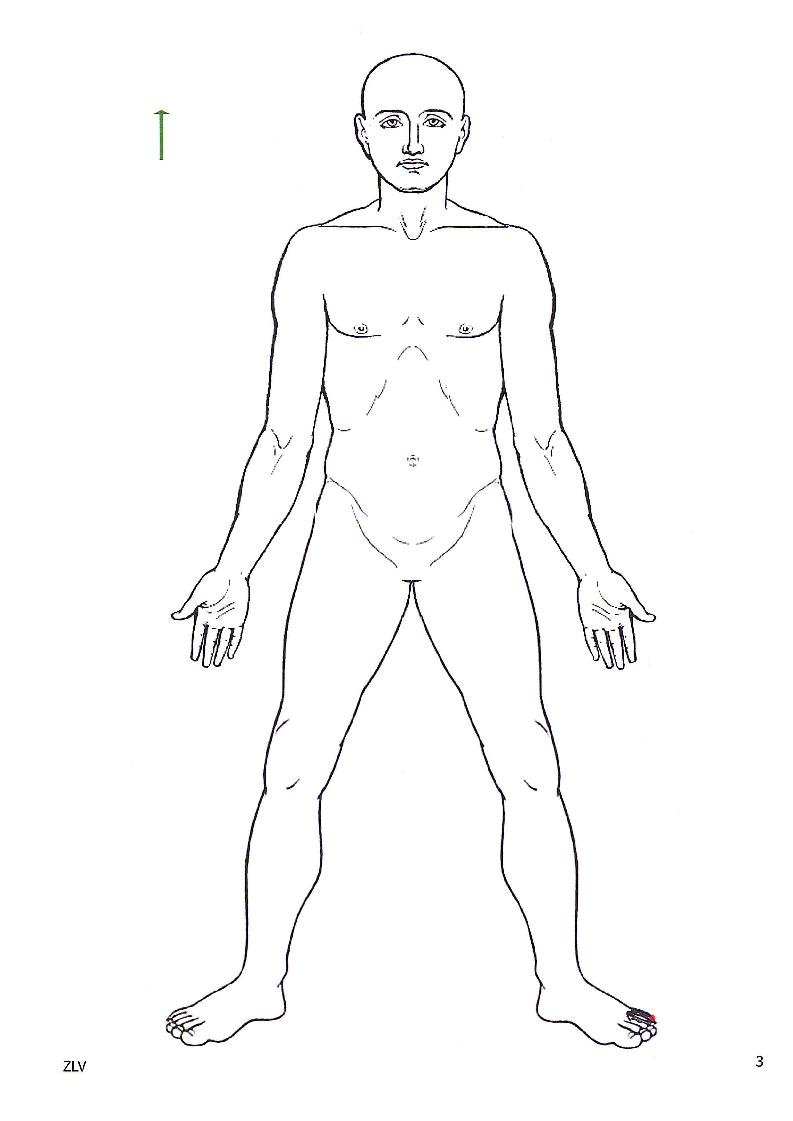

Supplement: S1 Raw Data — (ZIP) [file pone.0124808.s006.zip › Drawings - Imagined stimulation/toe_front/Subject_45_toe_front.jpg]

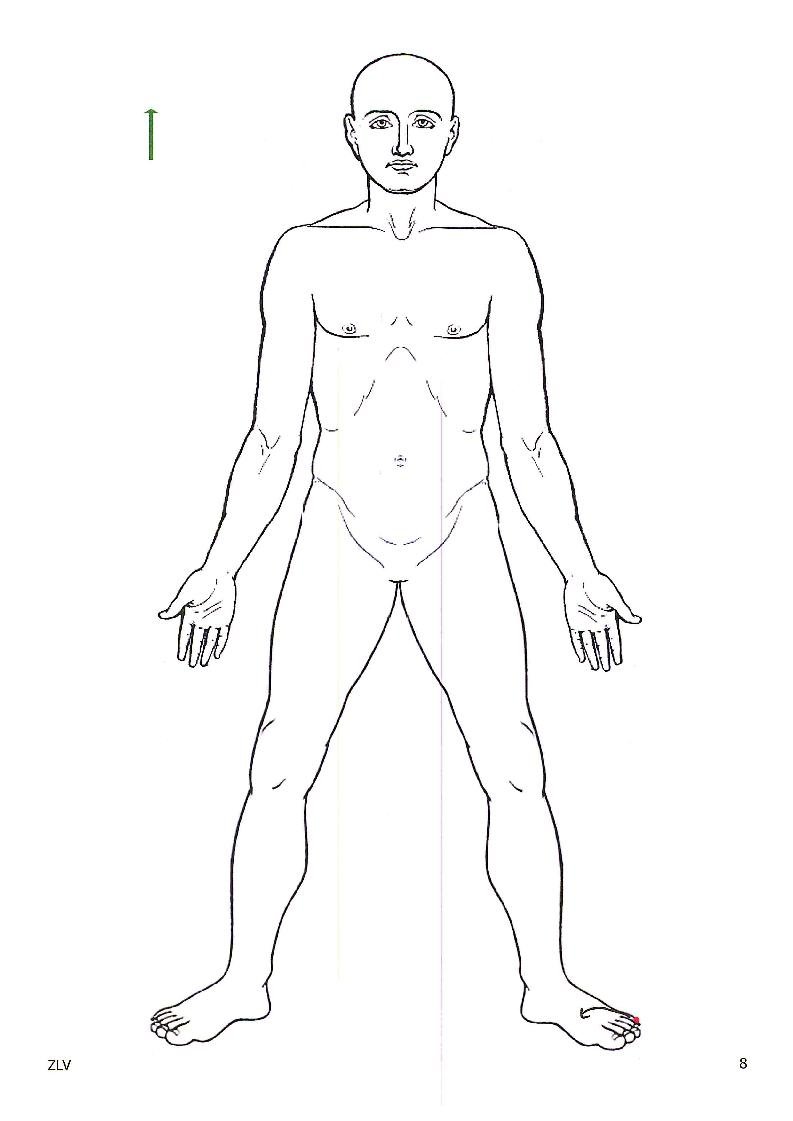

Supplement: S1 Raw Data — (ZIP) [file pone.0124808.s006.zip › Drawings - Imagined stimulation/toe_front/Subject_53_toe_front.jpg]

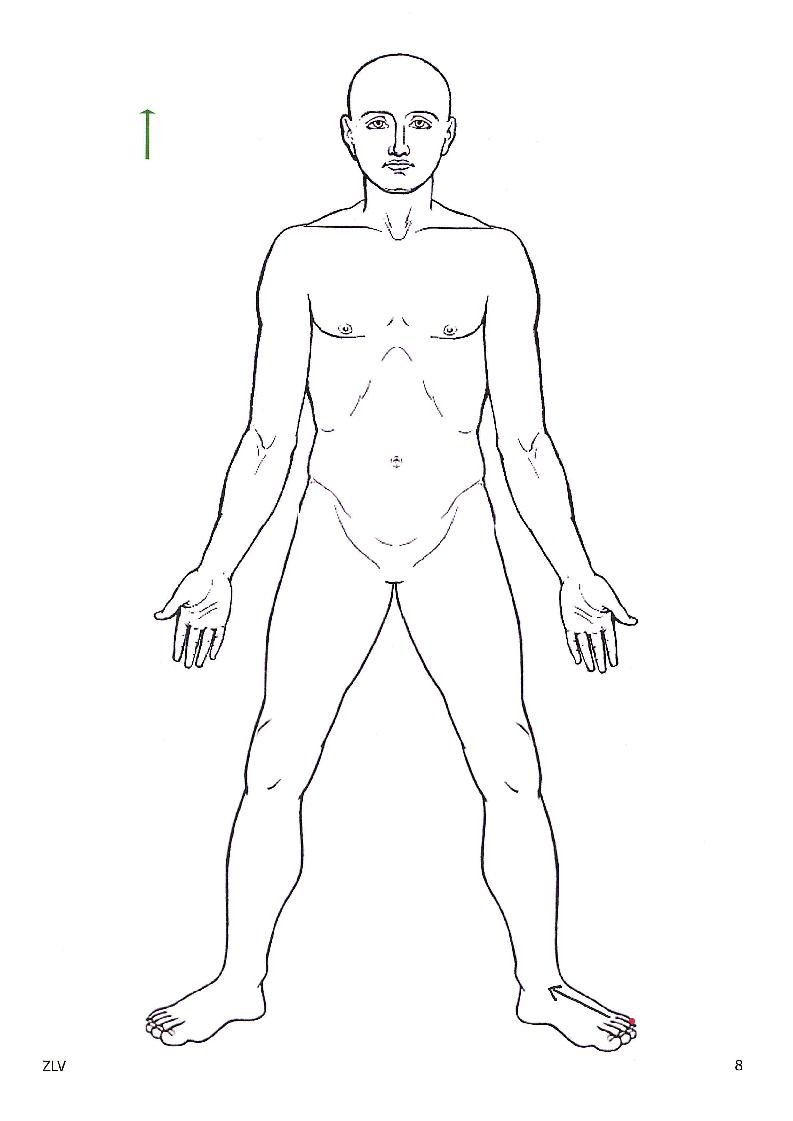

Supplement: S1 Raw Data — (ZIP) [file pone.0124808.s006.zip › Drawings - Imagined stimulation/toe_front/Subject_11_toe_front.jpg]

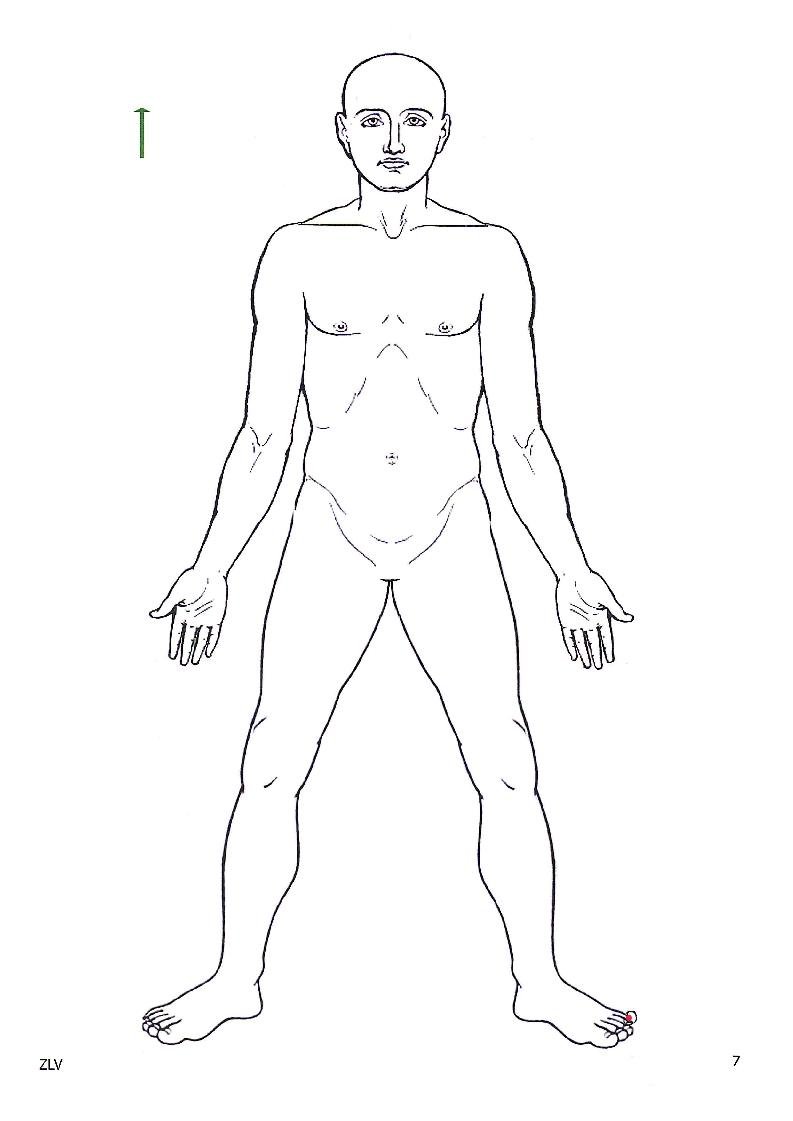

Supplement: S1 Raw Data — (ZIP) [file pone.0124808.s006.zip › Drawings - Imagined stimulation/toe_front/Subject_19_toe_front.jpg]

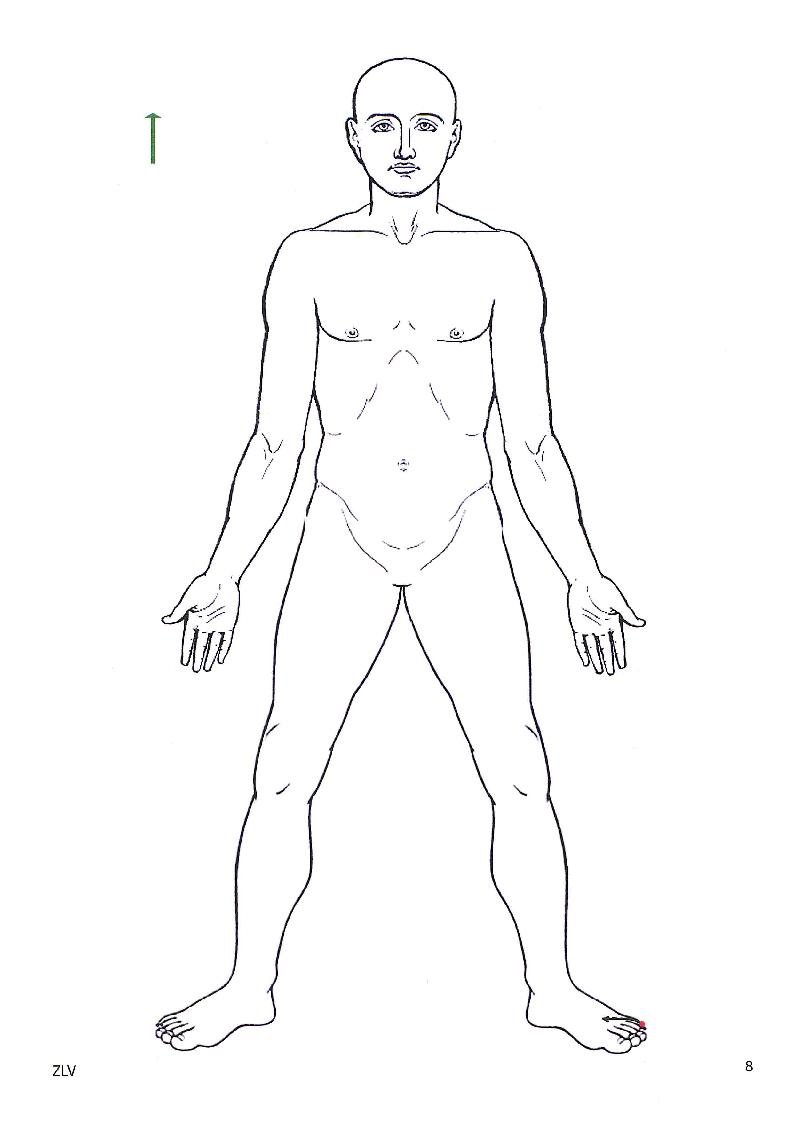

Supplement: S1 Raw Data — (ZIP) [file pone.0124808.s006.zip › Drawings - Imagined stimulation/toe_front/Subject_27_toe_front.jpg]

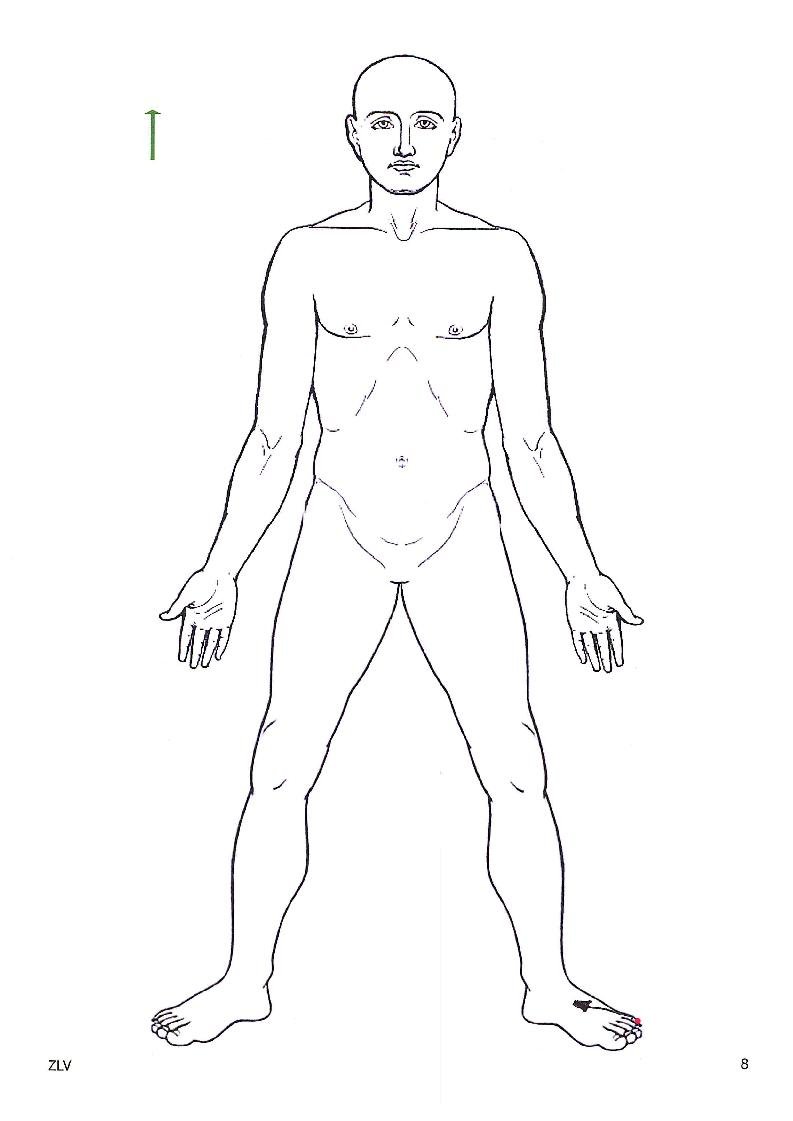

Supplement: S1 Raw Data — (ZIP) [file pone.0124808.s006.zip › Drawings - Imagined stimulation/toe_front/Subject_43_toe_front.jpg]

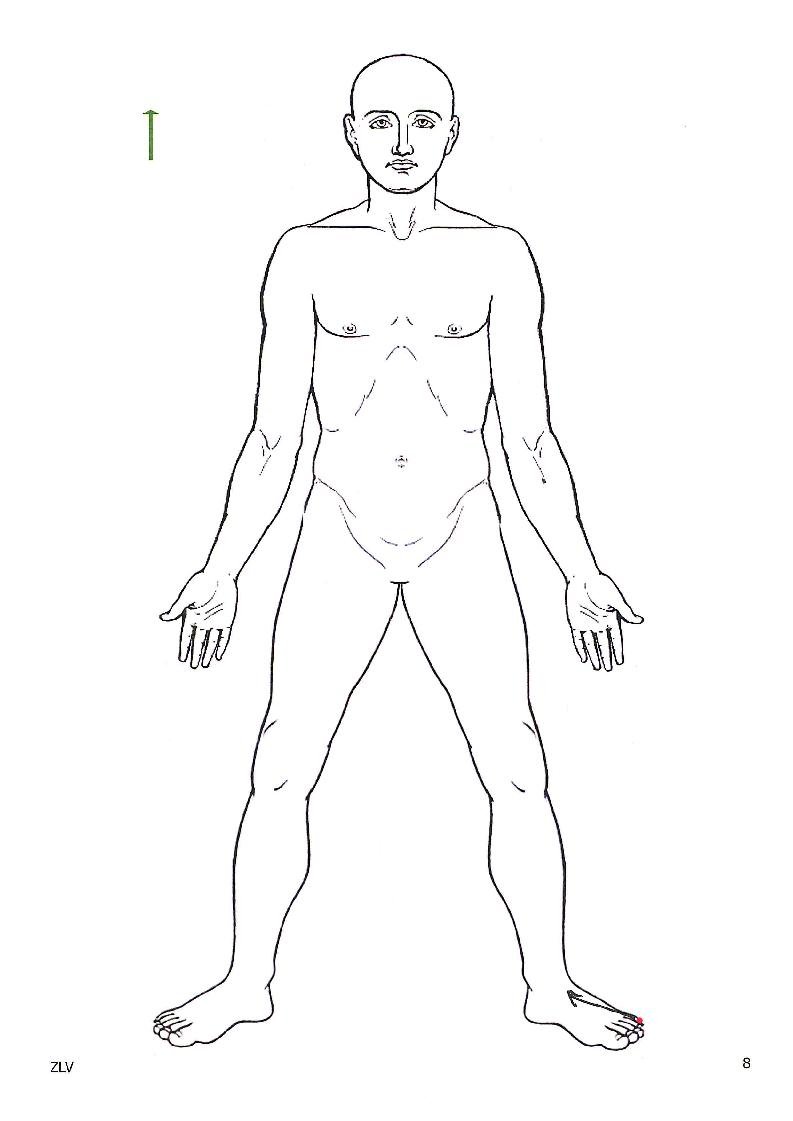

Supplement: S1 Raw Data — (ZIP) [file pone.0124808.s006.zip › Drawings - Imagined stimulation/toe_front/Subject_35_toe_front.jpg]

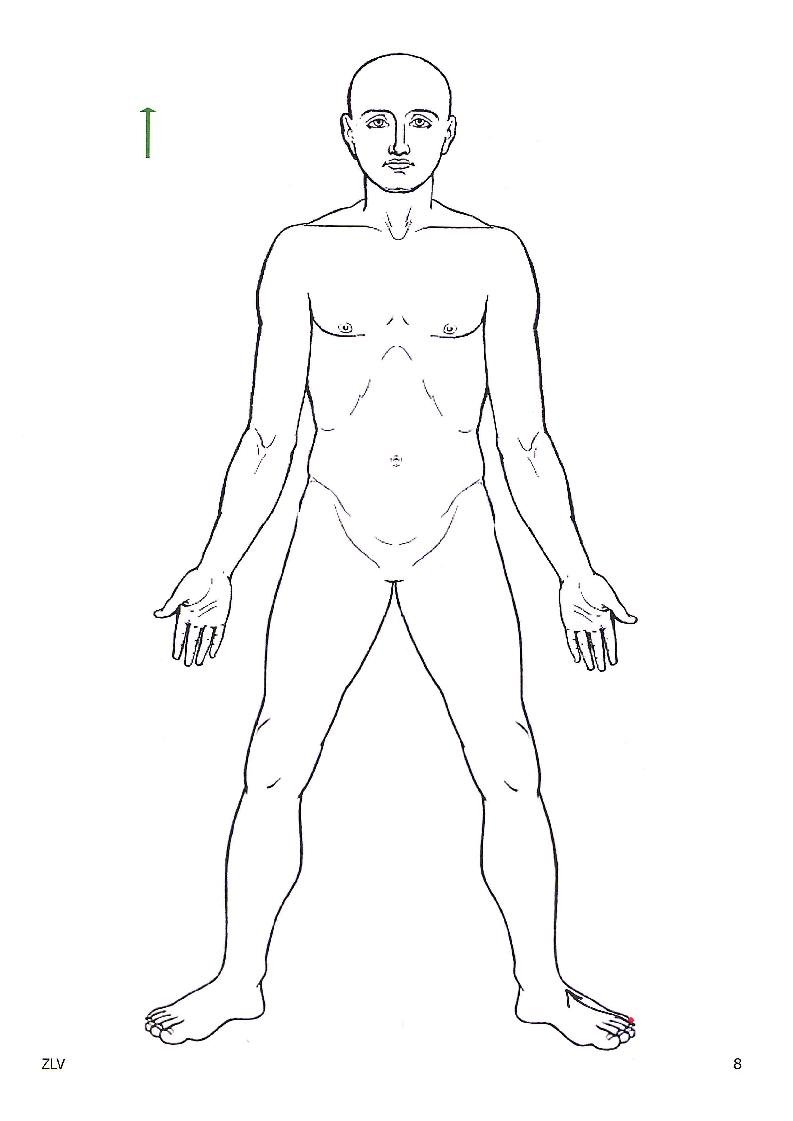

Supplement: S1 Raw Data — (ZIP) [file pone.0124808.s006.zip › Drawings - Imagined stimulation/toe_front/Subject_8_toe_front.jpg]

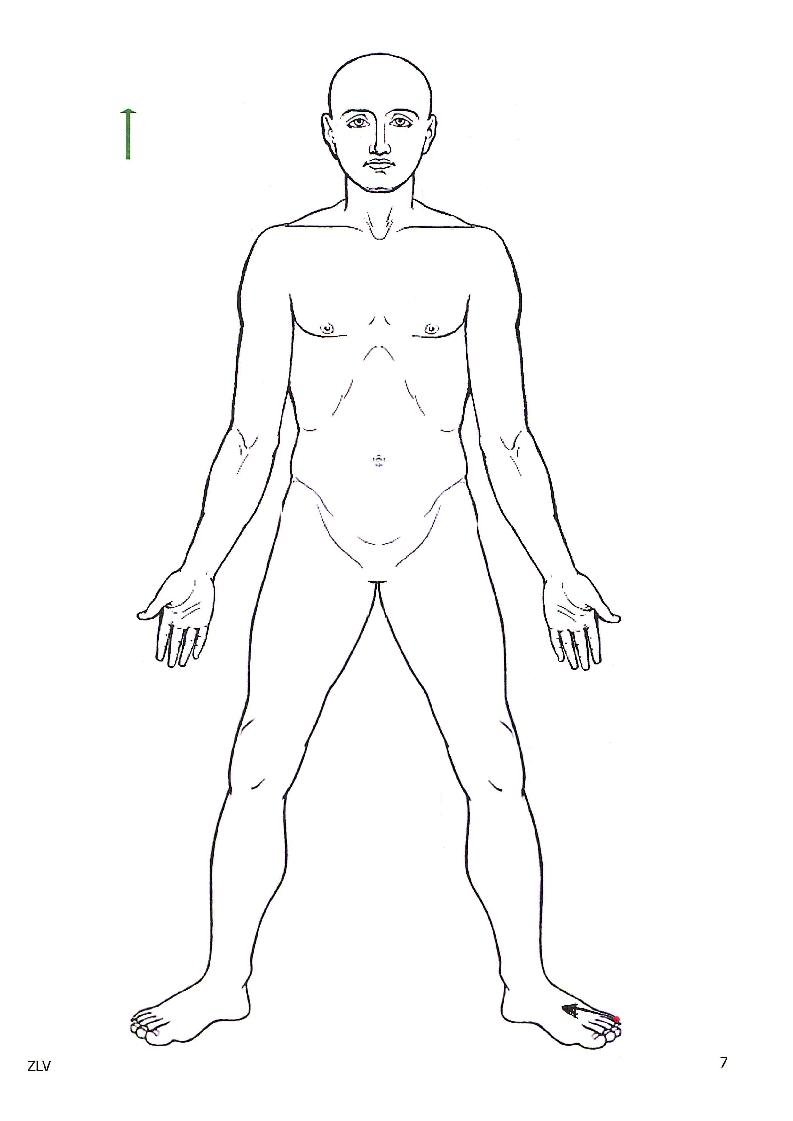

Supplement: S1 Raw Data — (ZIP) [file pone.0124808.s006.zip › Drawings - Imagined stimulation/toe_front/Subject_54_toe_front.jpg]

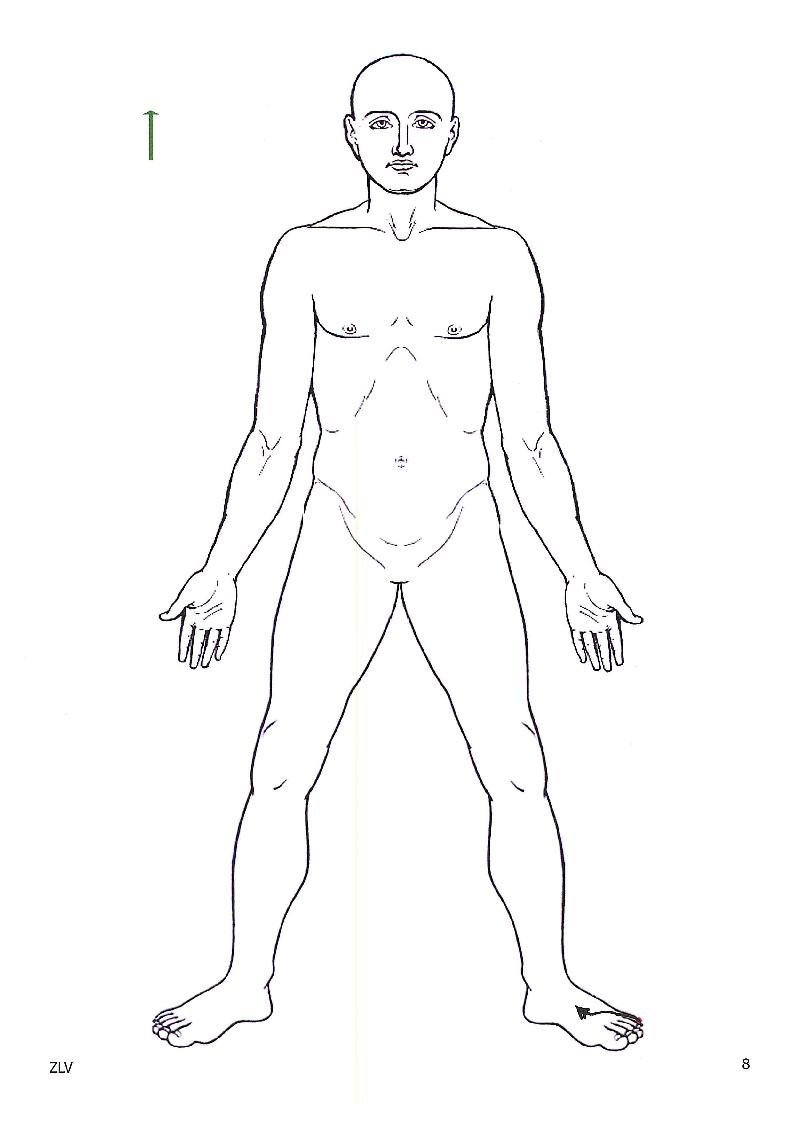

Supplement: S1 Raw Data — (ZIP) [file pone.0124808.s006.zip › Drawings - Imagined stimulation/toe_front/Subject_17_toe_front.jpg]

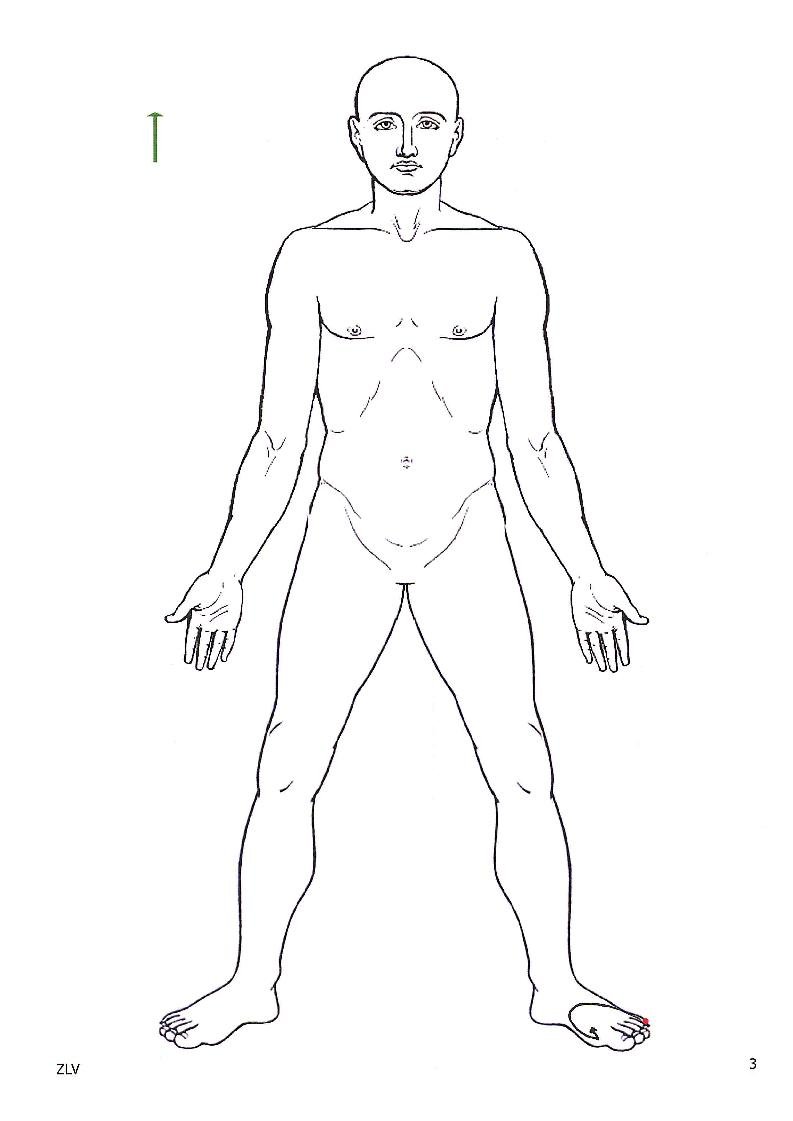

Supplement: S1 Raw Data — (ZIP) [file pone.0124808.s006.zip › Drawings - Imagined stimulation/toe_front/Subject_25_toe_front.jpg]

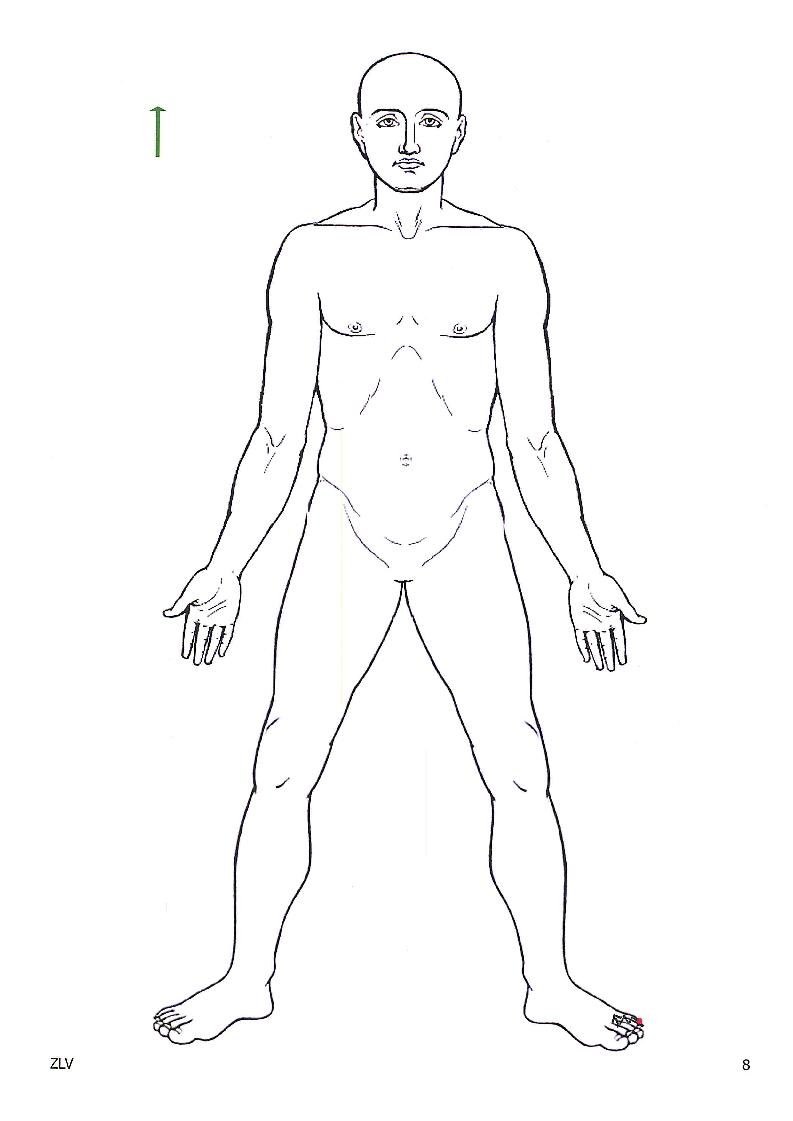

Supplement: S1 Raw Data — (ZIP) [file pone.0124808.s006.zip › Drawings - Imagined stimulation/toe_front/Subject_33_toe_front.jpg]

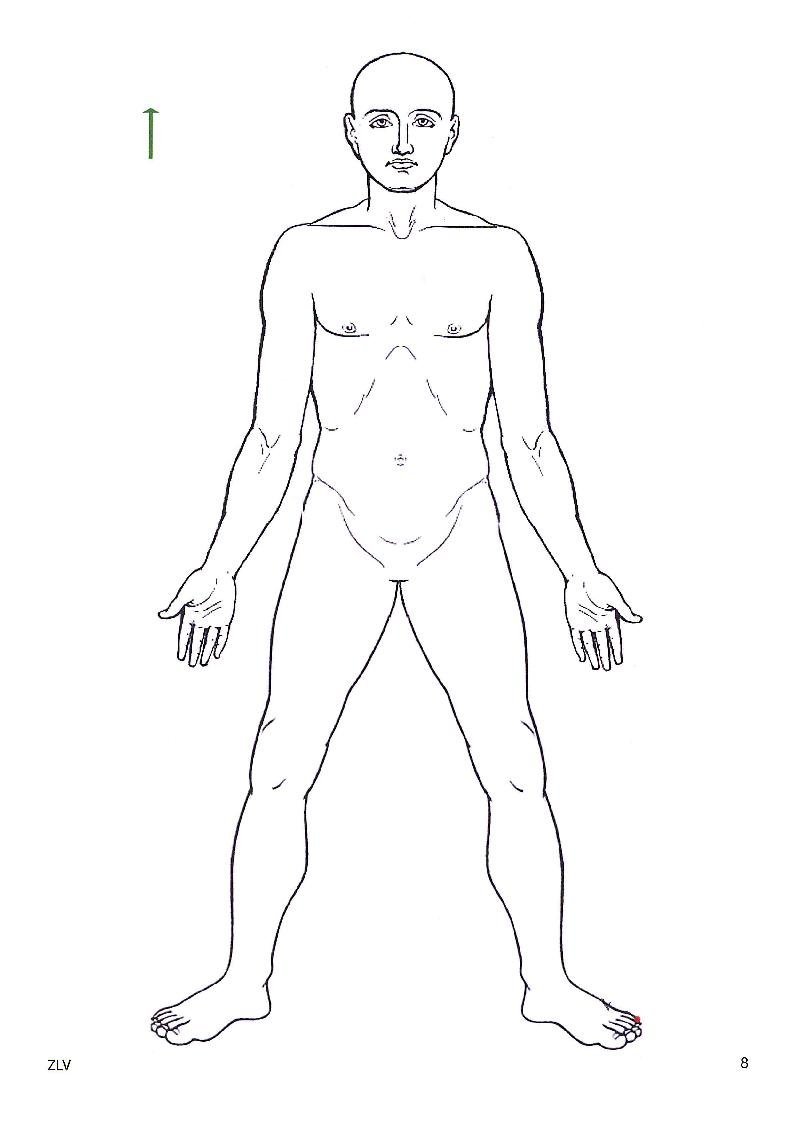

Supplement: S1 Raw Data — (ZIP) [file pone.0124808.s006.zip › Drawings - Imagined stimulation/toe_front/Subject_1_toe_front.jpg]

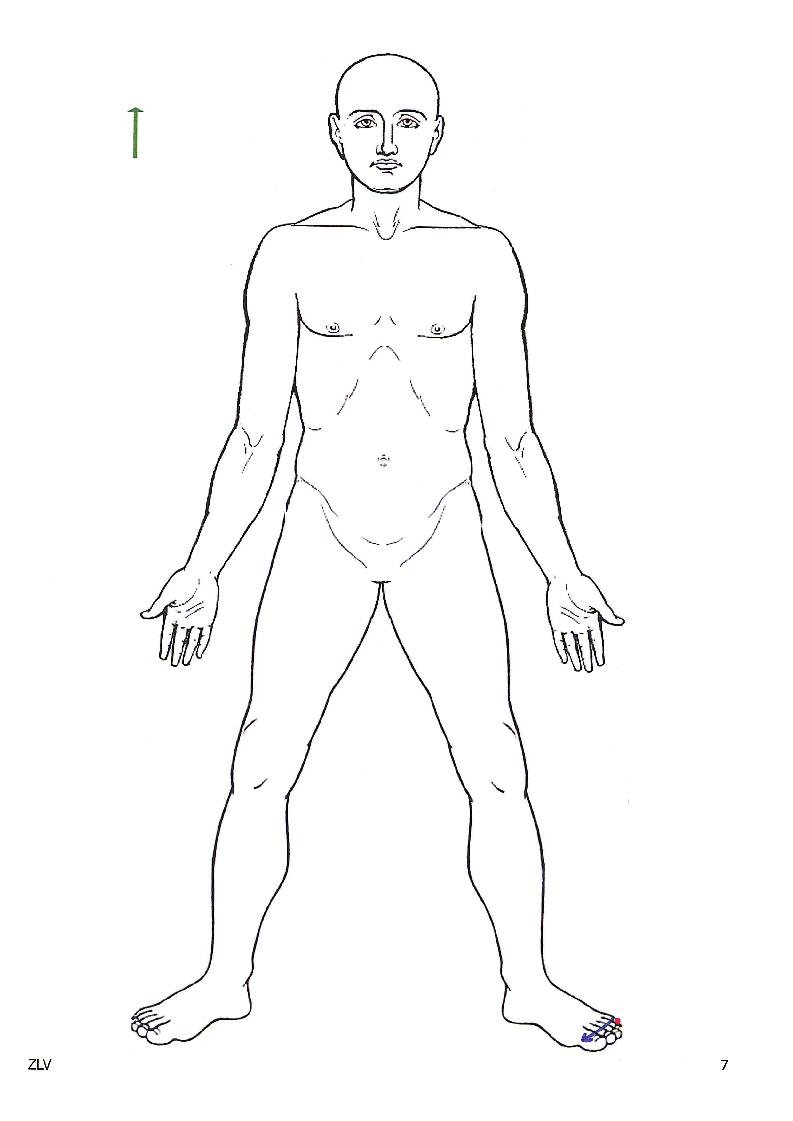

Supplement: S1 Raw Data — (ZIP) [file pone.0124808.s006.zip › Drawings - Imagined stimulation/toe_front/Subject_36_toe_front.jpg]

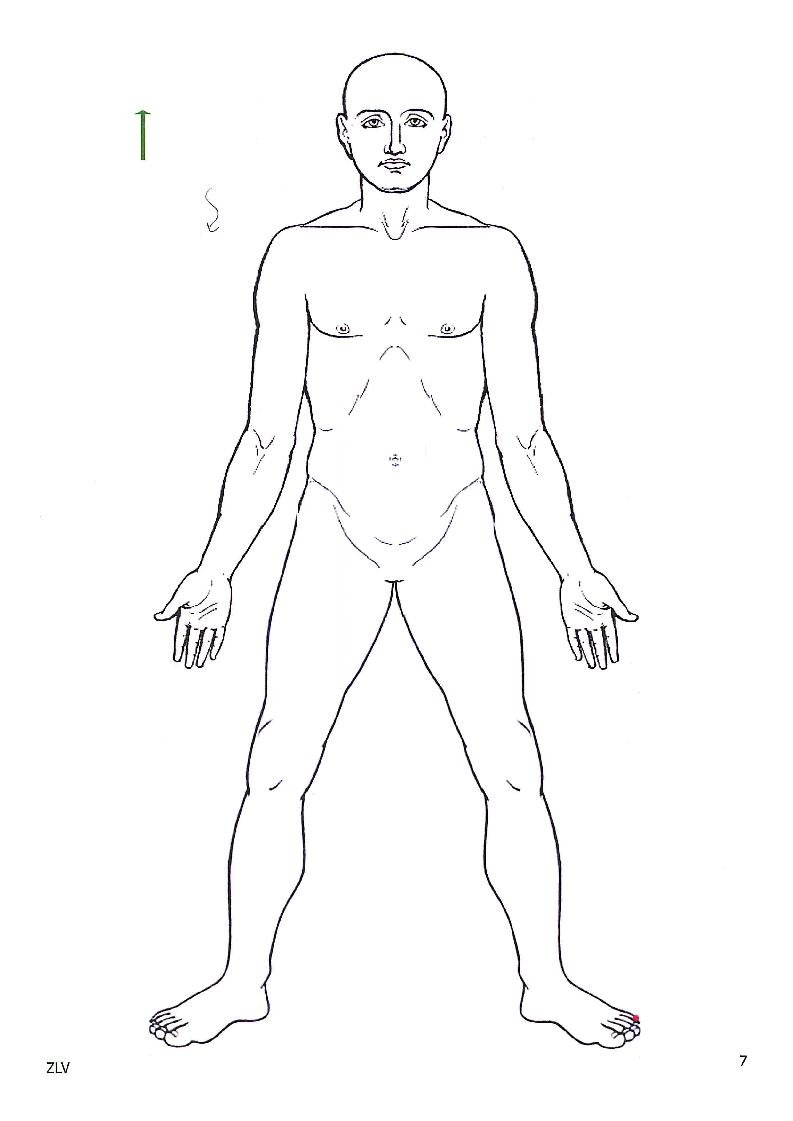

Supplement: S1 Raw Data — (ZIP) [file pone.0124808.s006.zip › Drawings - Imagined stimulation/toe_front/Subject_52_toe_front.jpg]

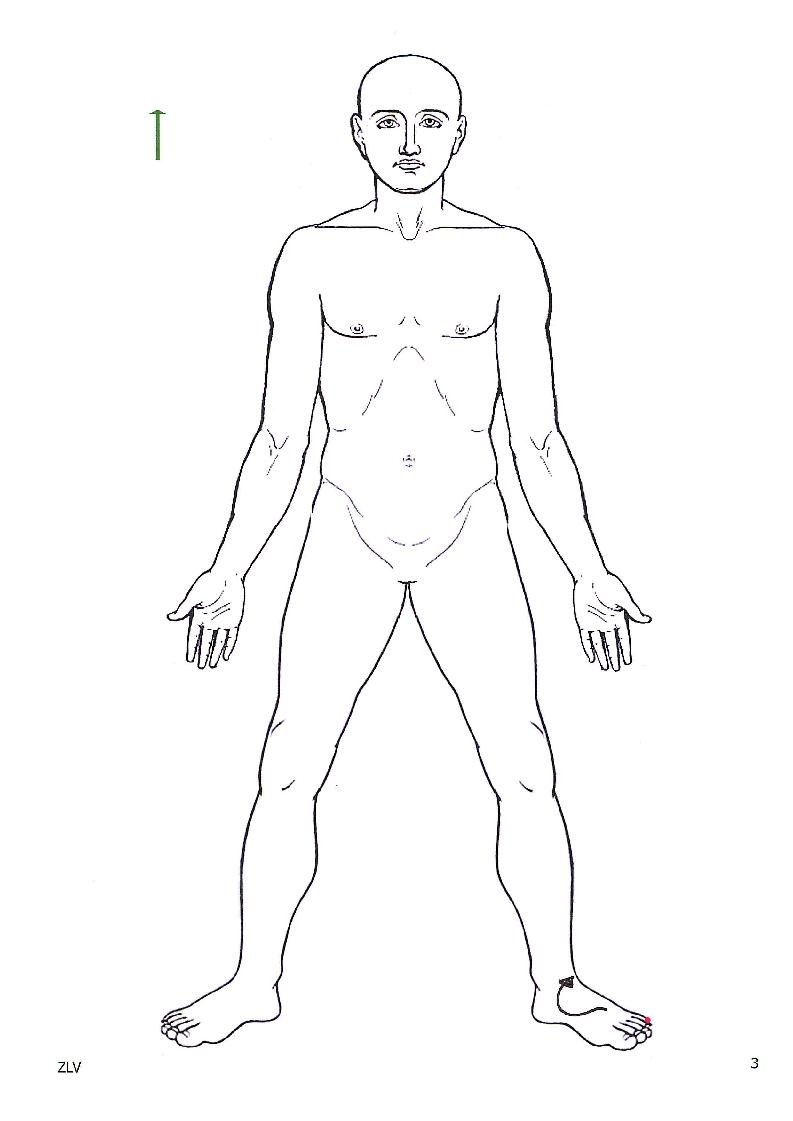

Supplement: S1 Raw Data — (ZIP) [file pone.0124808.s006.zip › Drawings - Imagined stimulation/toe_front/Subject_44_toe_front.jpg]

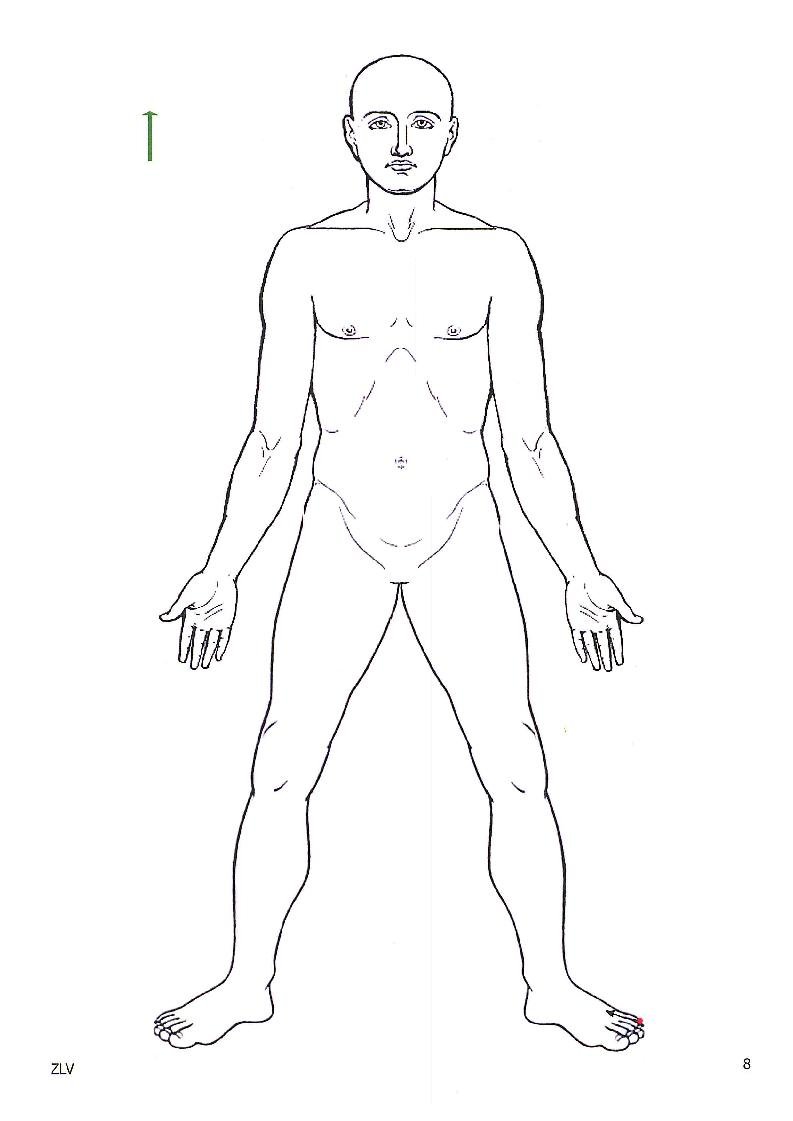

Supplement: S1 Raw Data — (ZIP) [file pone.0124808.s006.zip › Drawings - Imagined stimulation/toe_front/Subject_15_toe_front.jpg]

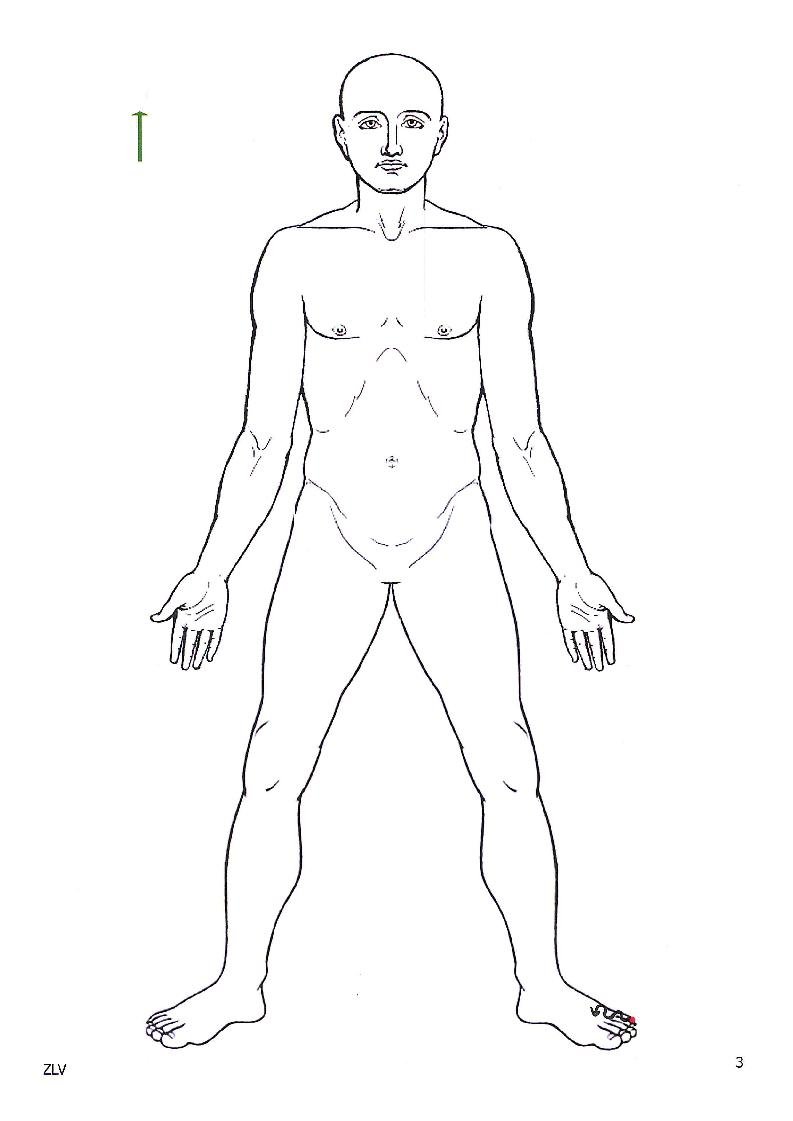

Supplement: S1 Raw Data — (ZIP) [file pone.0124808.s006.zip › Drawings - Imagined stimulation/toe_front/Subject_23_toe_front.jpg]

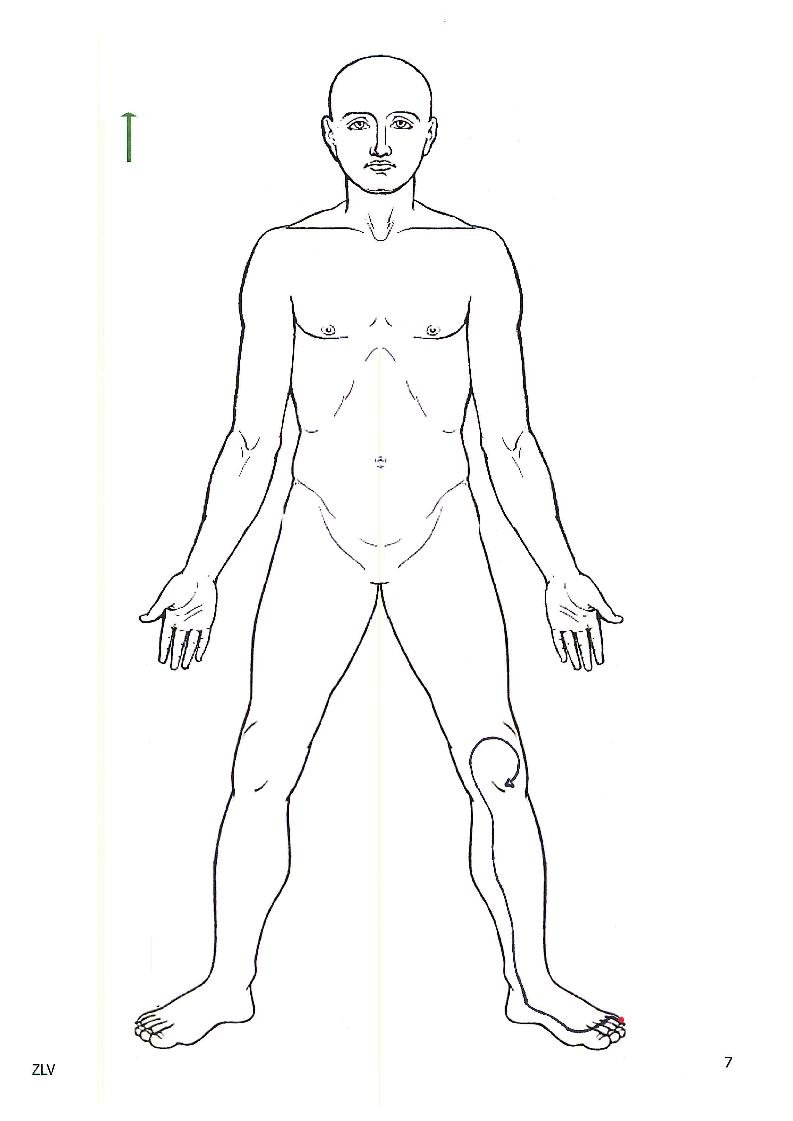

Supplement: S1 Raw Data — (ZIP) [file pone.0124808.s006.zip › Drawings - Imagined stimulation/toe_front/Subject_9_toe_front.jpg]

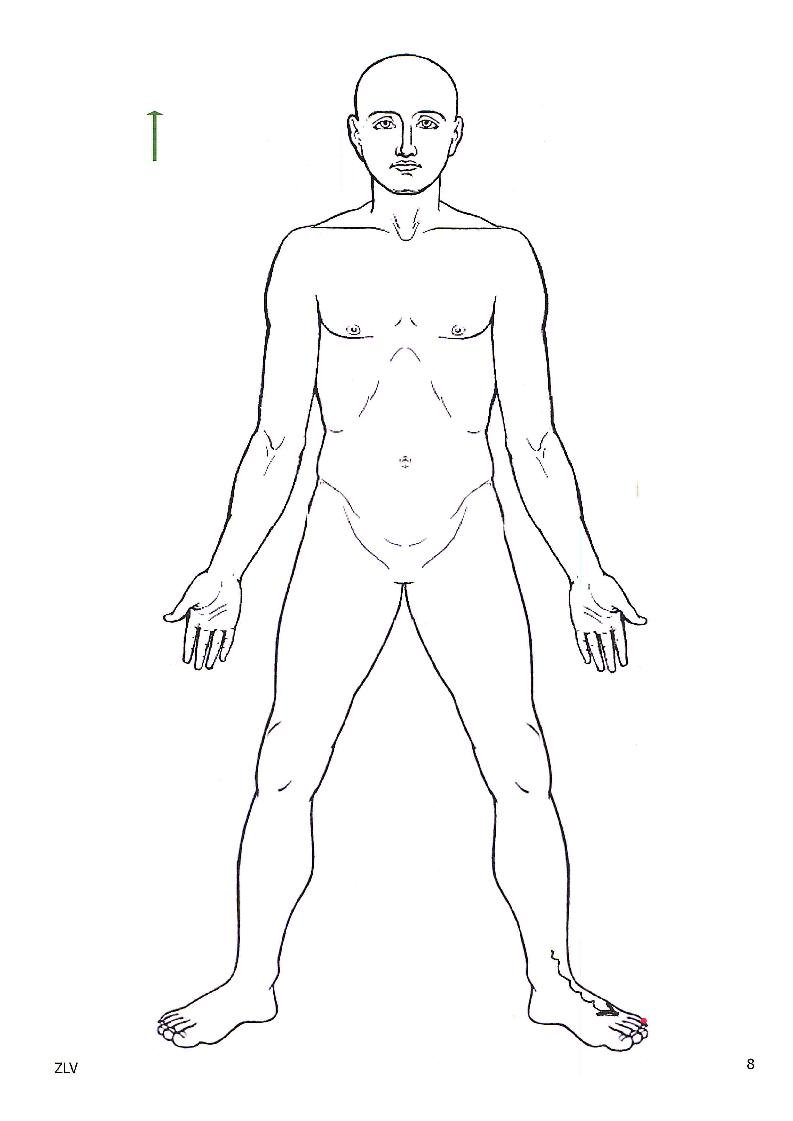

Supplement: S1 Raw Data — (ZIP) [file pone.0124808.s006.zip › Drawings - Imagined stimulation/toe_front/Subject_26_toe_front.jpg]

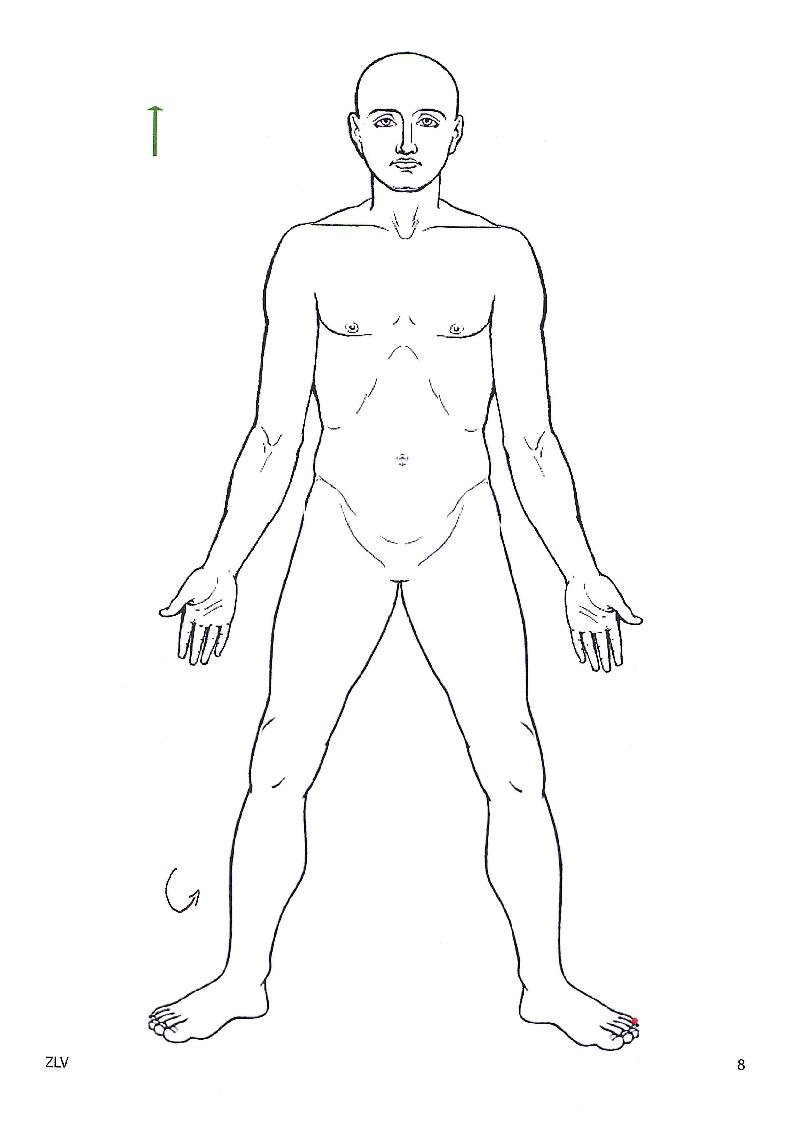

Supplement: S1 Raw Data — (ZIP) [file pone.0124808.s006.zip › Drawings - Imagined stimulation/toe_front/Subject_34_toe_front.jpg]

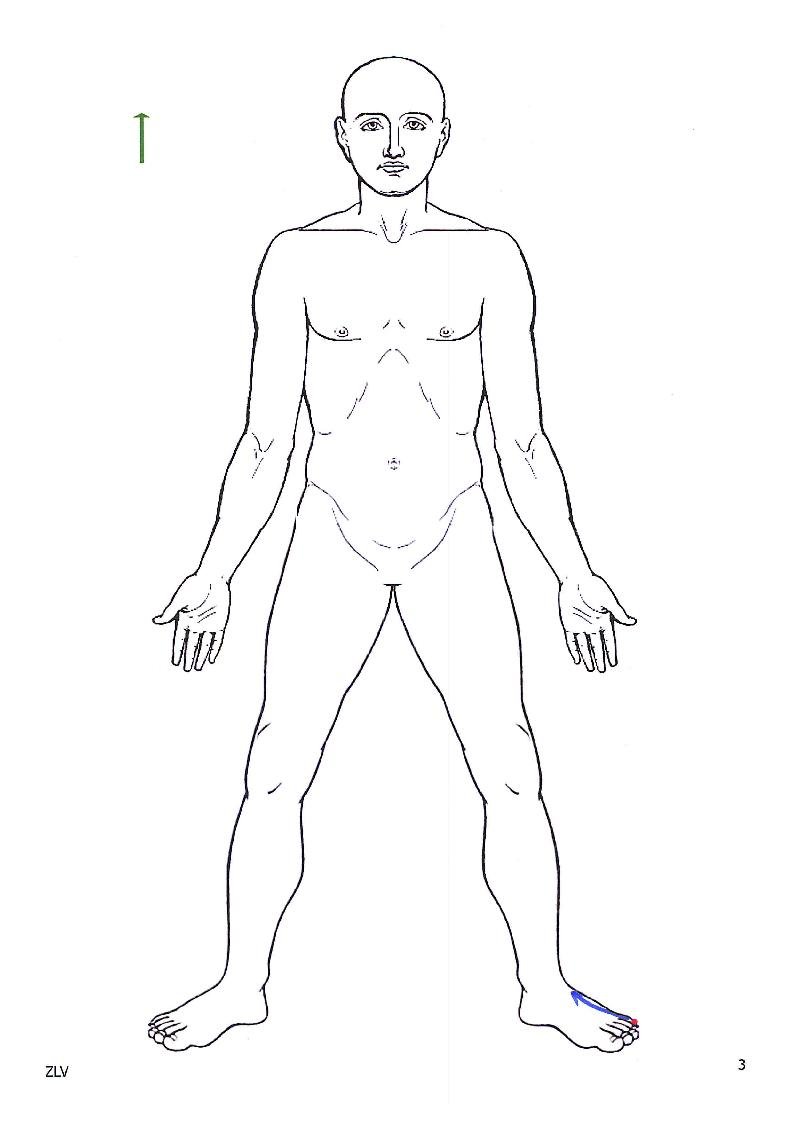

Supplement: S1 Raw Data — (ZIP) [file pone.0124808.s006.zip › Drawings - Imagined stimulation/toe_front/Subject_42_toe_front.jpg]

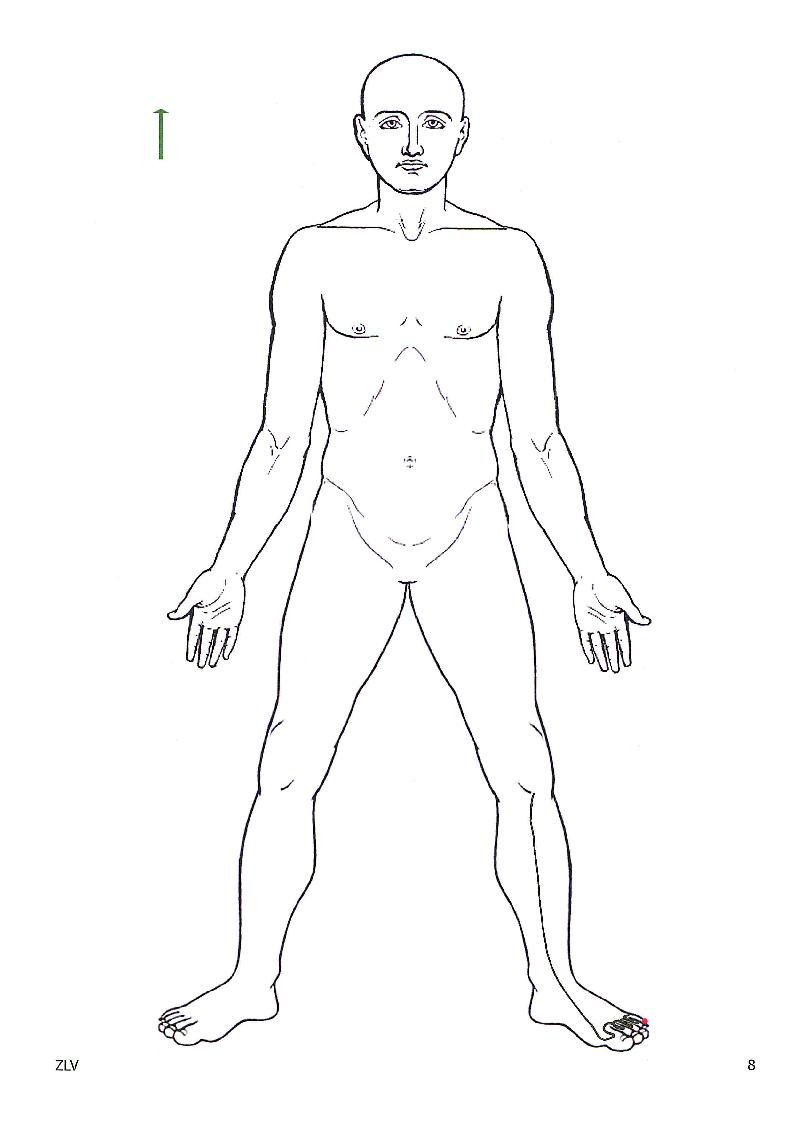

Supplement: S1 Raw Data — (ZIP) [file pone.0124808.s006.zip › Drawings - Imagined stimulation/toe_front/Subject_50_toe_front.jpg]

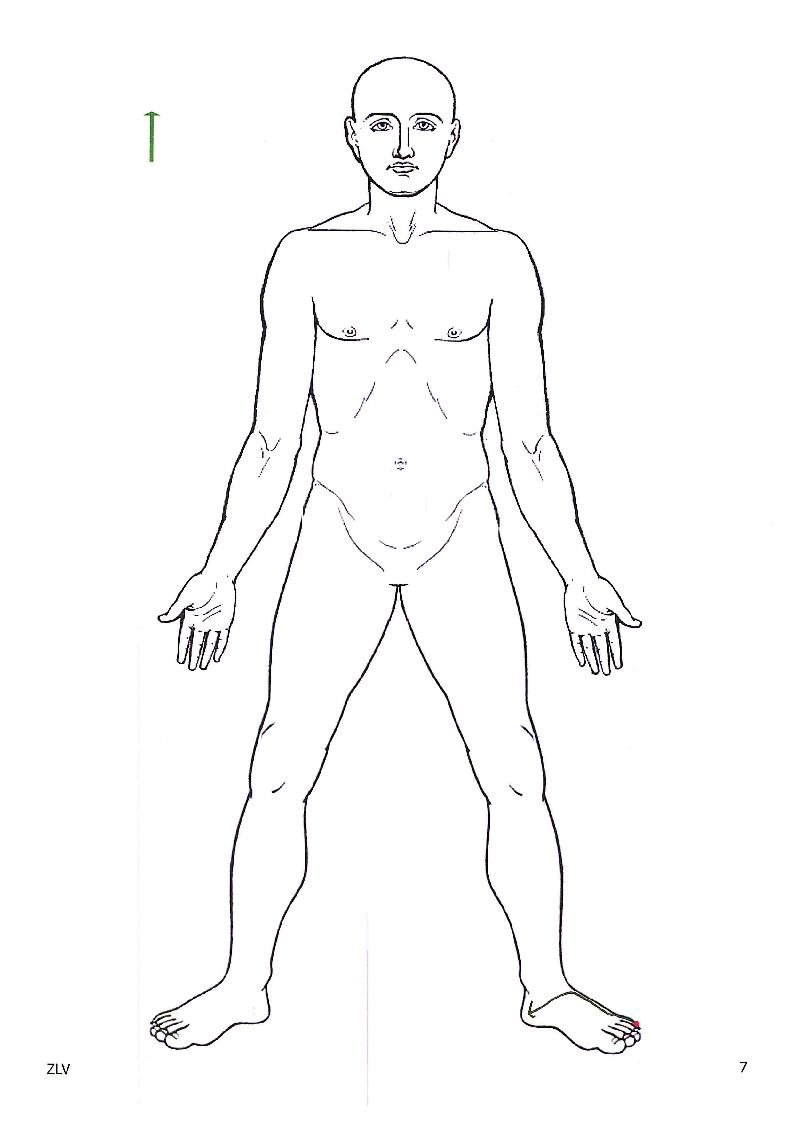

Supplement: S1 Raw Data — (ZIP) [file pone.0124808.s006.zip › Drawings - Imagined stimulation/toe_front/Subject_7_toe_front.jpg]

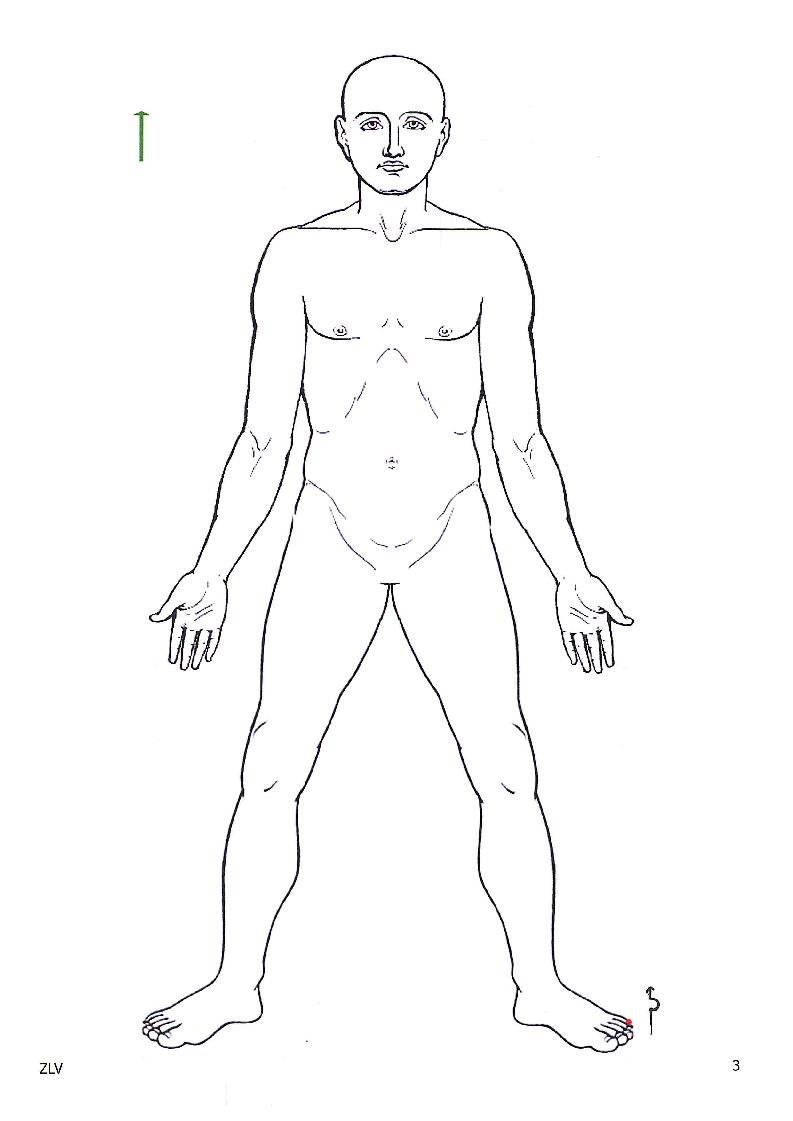

Supplement: S1 Raw Data — (ZIP) [file pone.0124808.s006.zip › Drawings - Imagined stimulation/toe_front/Subject_16_toe_front.jpg]

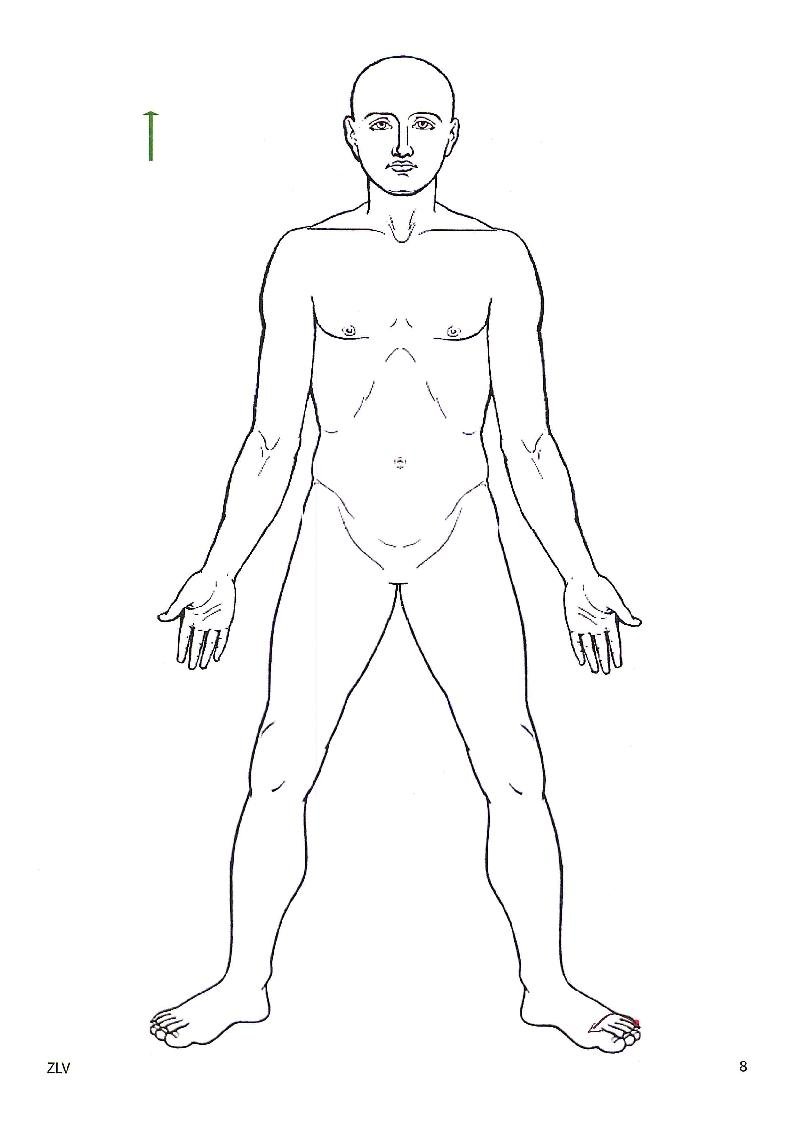

Supplement: S1 Raw Data — (ZIP) [file pone.0124808.s006.zip › Drawings - Imagined stimulation/toe_front/Subject_24_toe_front.jpg]

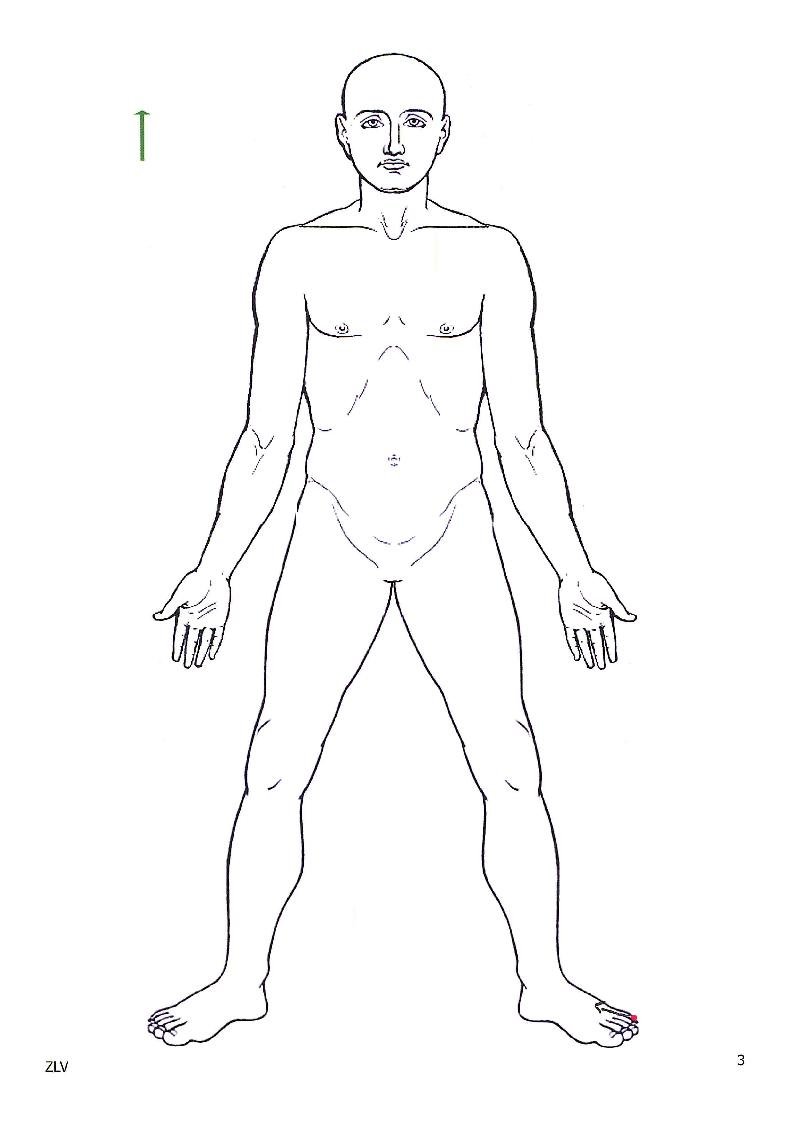

Supplement: S1 Raw Data — (ZIP) [file pone.0124808.s006.zip › Drawings - Imagined stimulation/toe_front/Subject_32_toe_front.jpg]

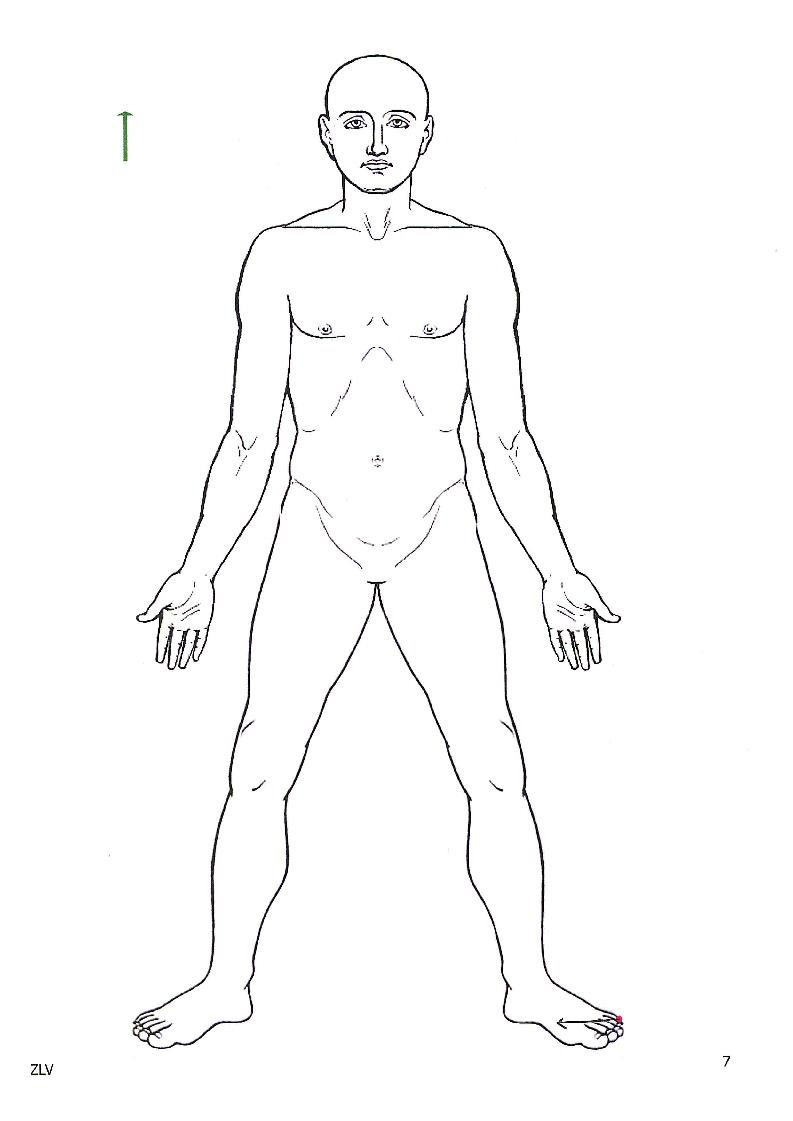

Supplement: S1 Raw Data — (ZIP) [file pone.0124808.s006.zip › Drawings - Imagined stimulation/toe_front/Subject_40_toe_front.jpg]

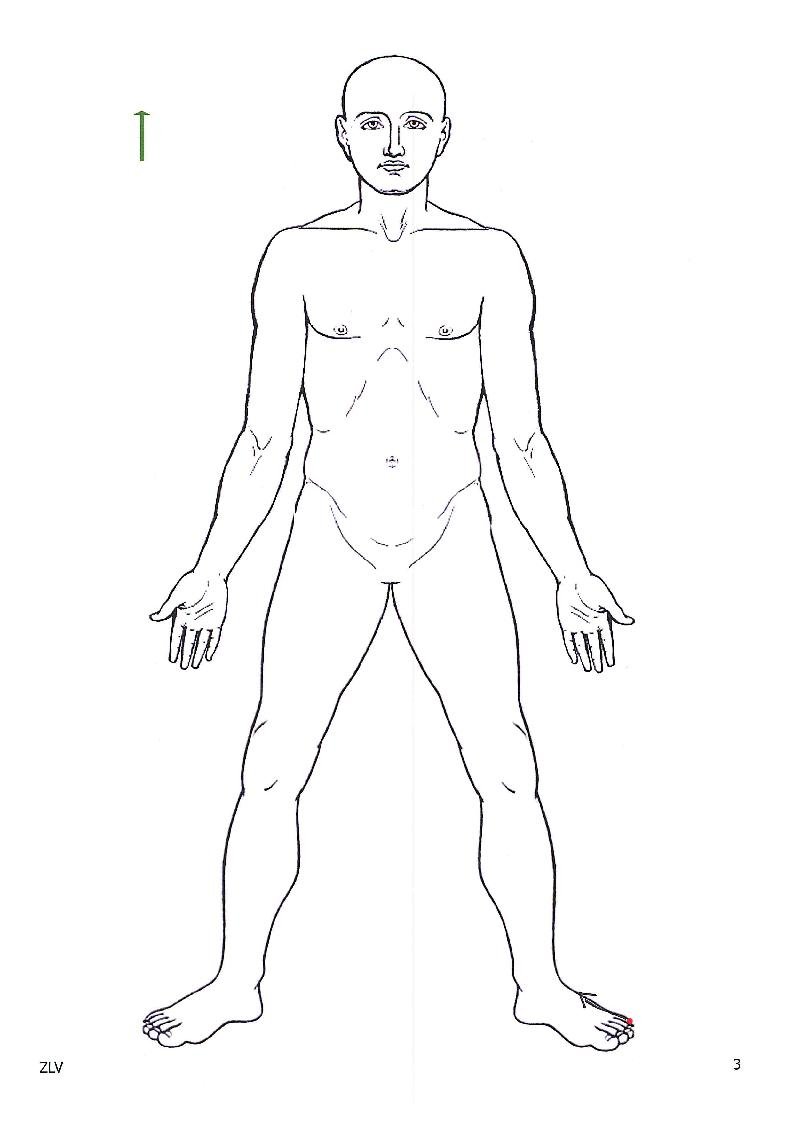

Supplement: S1 Raw Data — (ZIP) [file pone.0124808.s006.zip › Drawings - Imagined stimulation/toe_front/Subject_5_toe_front.jpg]

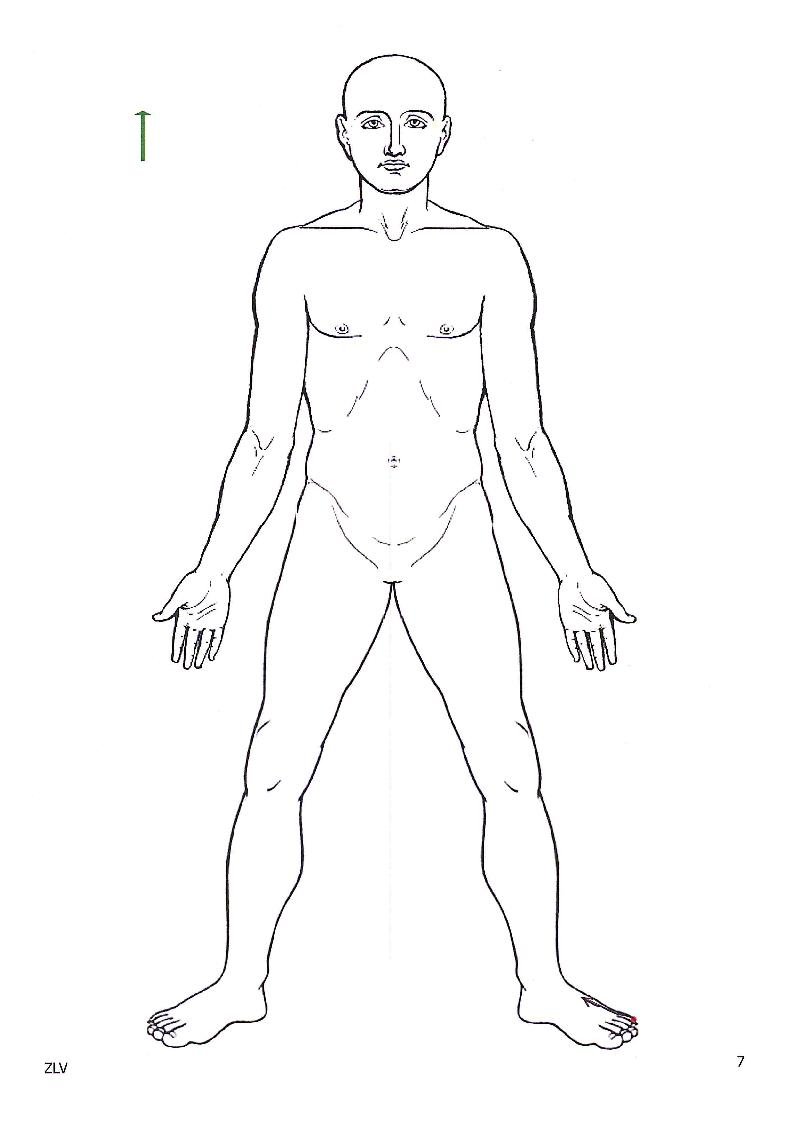

Supplement: S1 Raw Data — (ZIP) [file pone.0124808.s006.zip › Drawings - Imagined stimulation/toe_front/Subject_48_toe_front.jpg]

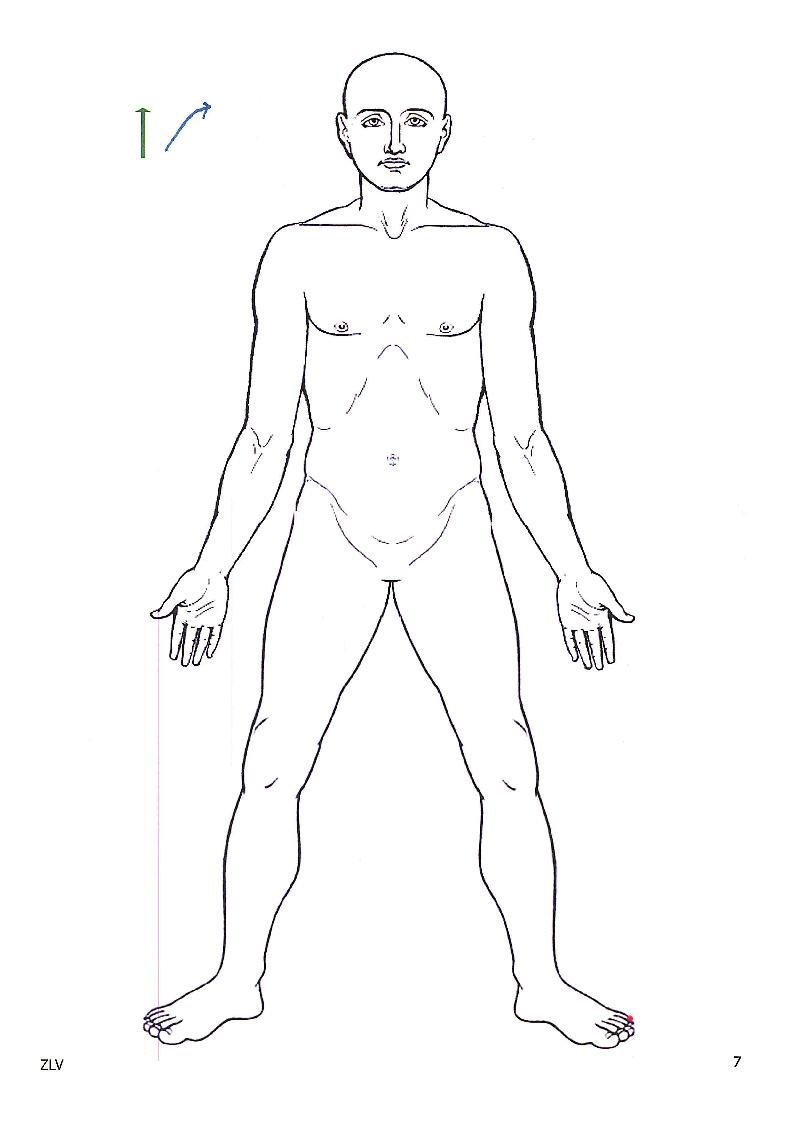

Supplement: S1 Raw Data — (ZIP) [file pone.0124808.s006.zip › Drawings - Imagined stimulation/toe_front/Subject_56_toe_front.jpg]

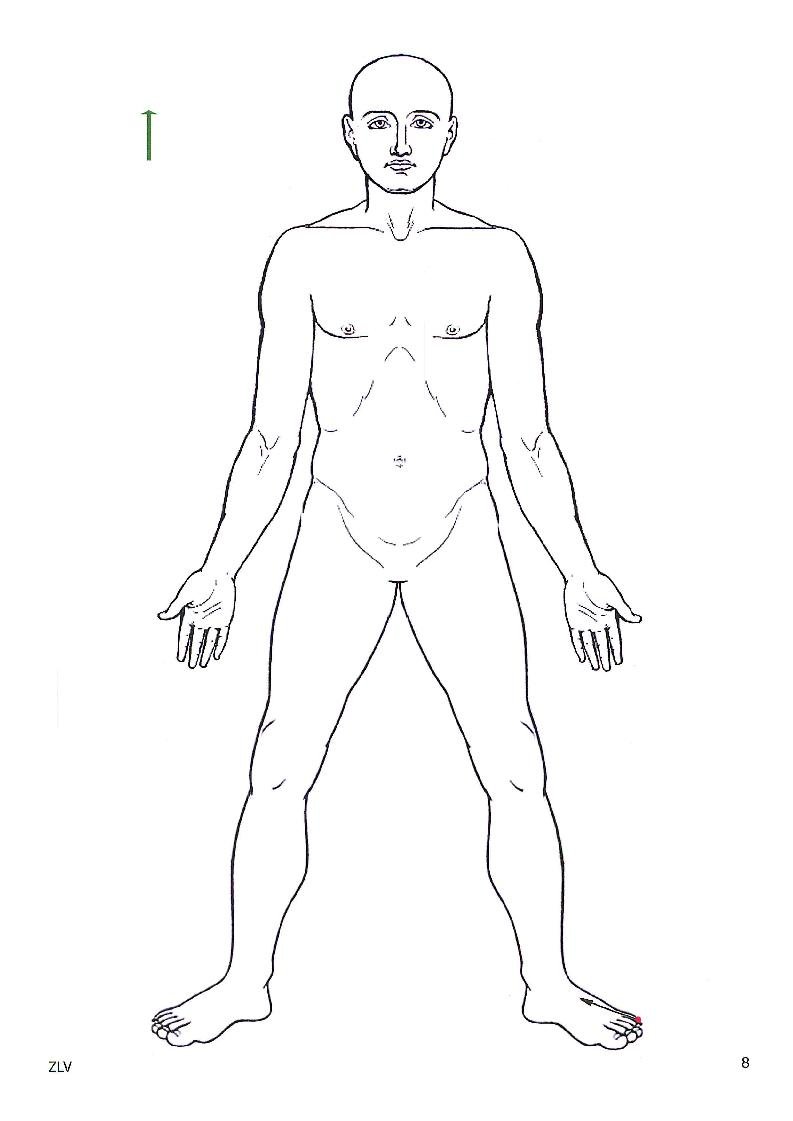

Supplement: S1 Raw Data — (ZIP) [file pone.0124808.s006.zip › Drawings - Imagined stimulation/toe_front/Subject_51_toe_front.jpg]

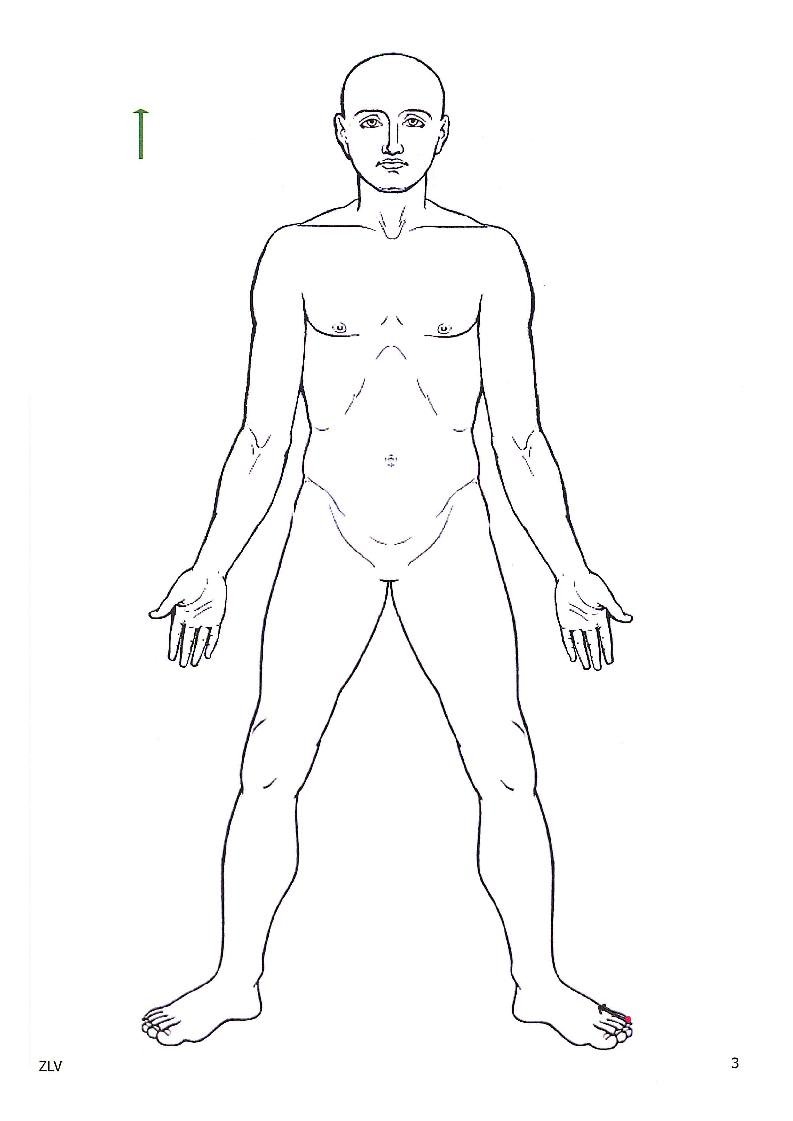

Supplement: S1 Raw Data — (ZIP) [file pone.0124808.s006.zip › Drawings - Imagined stimulation/toe_front/Subject_22_toe_front.jpg]
