# Supplementary figures and images for: Placebo-Induced Somatic Sensations: A Multi-Modal Study of Three Different Placebo Interventions (part 3 of 3)
Source: PLoS One. 2015 Apr 22;10(4):e0124808. doi: 10.1371/journal.pone.0124808 (PMC4406515; doi:10.1371/journal.pone.0124808)

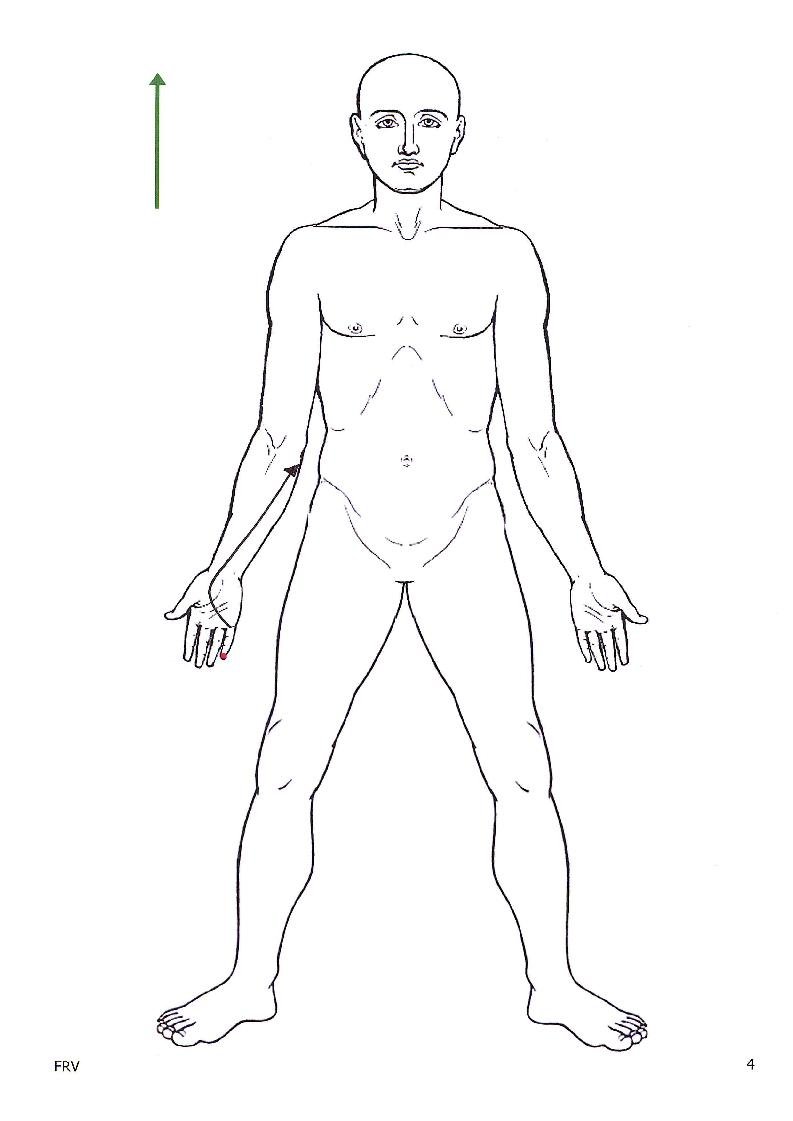

Supplement: S1 Raw Data — (ZIP) [file pone.0124808.s006.zip › Drawings - Imagined stimulation/finger_front/Subject_44_finger_front.jpg]

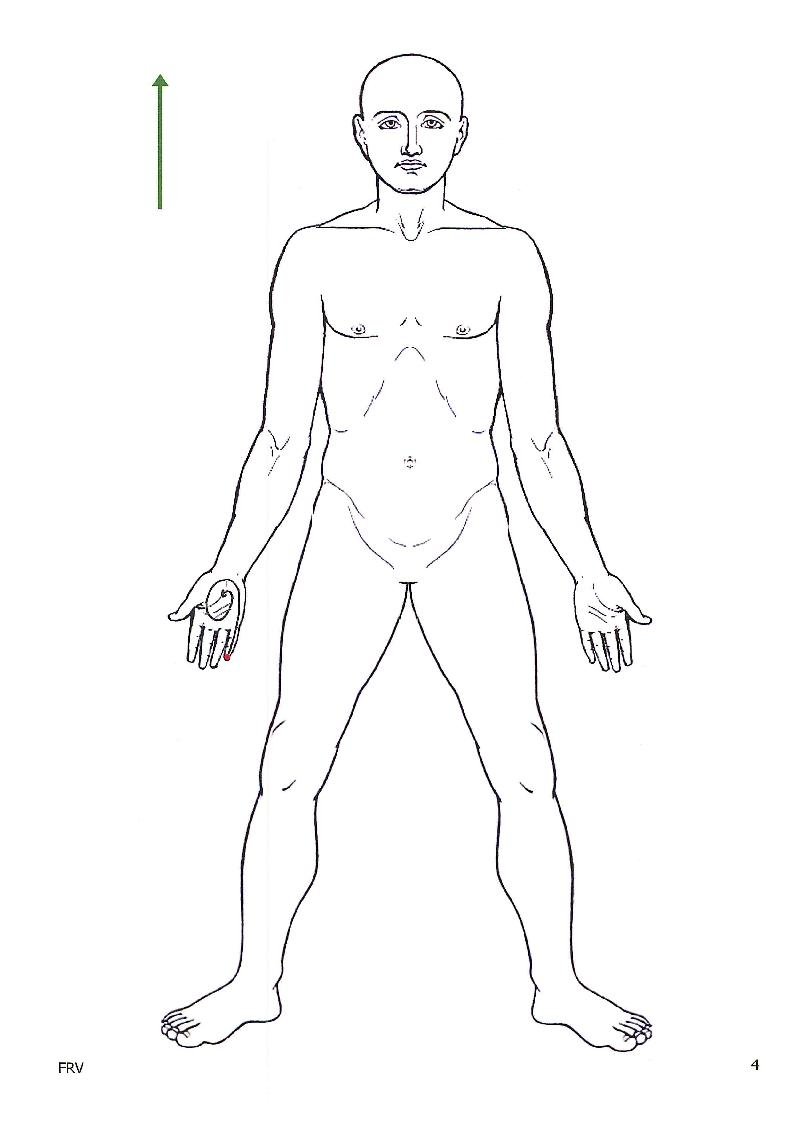

Supplement: S1 Raw Data — (ZIP) [file pone.0124808.s006.zip › Drawings - Imagined stimulation/finger_front/Subject_38_finger_front.jpg]

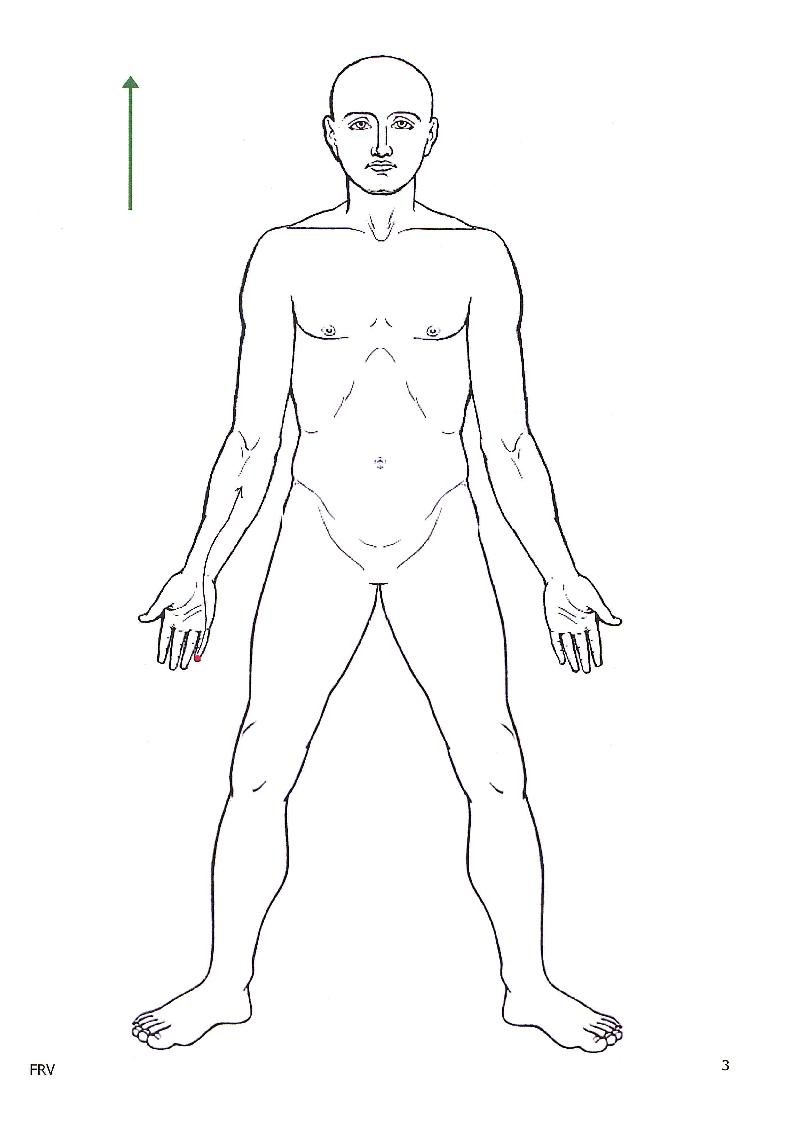

Supplement: S1 Raw Data — (ZIP) [file pone.0124808.s006.zip › Drawings - Imagined stimulation/finger_front/Subject_39_finger_front.jpg]

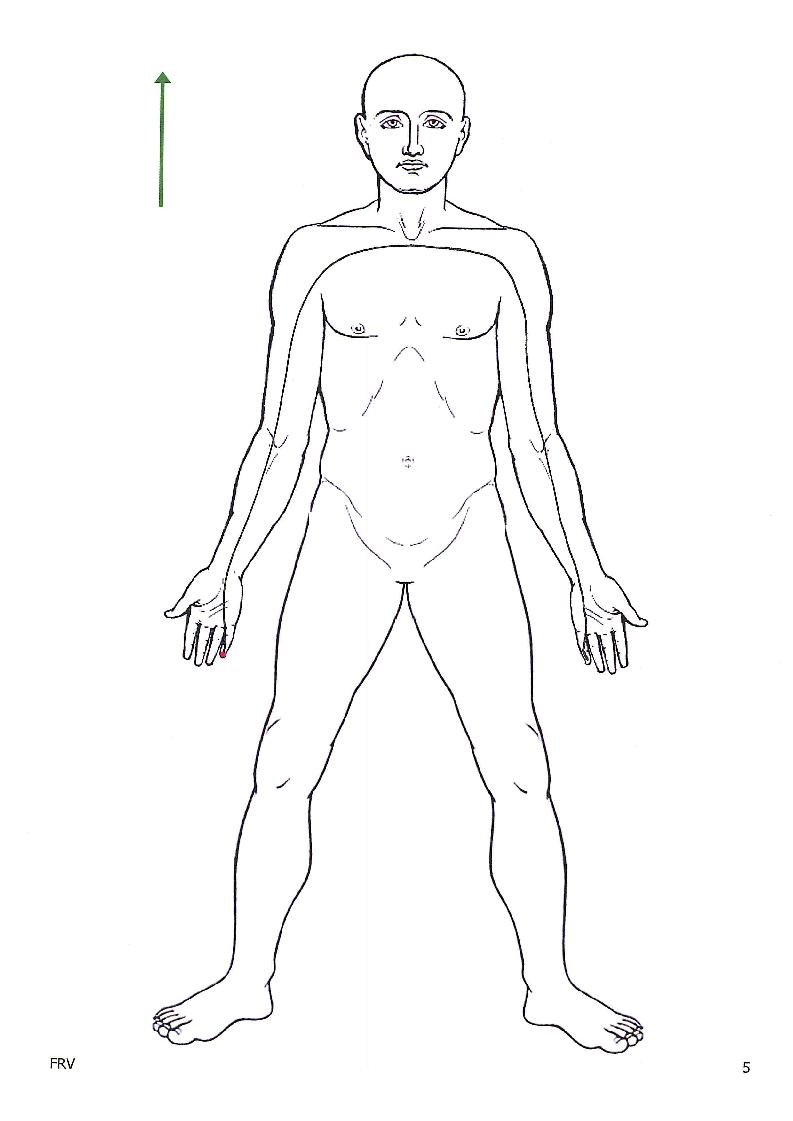

Supplement: S1 Raw Data — (ZIP) [file pone.0124808.s006.zip › Drawings - Imagined stimulation/finger_front/Subject_50_finger_front.jpg]

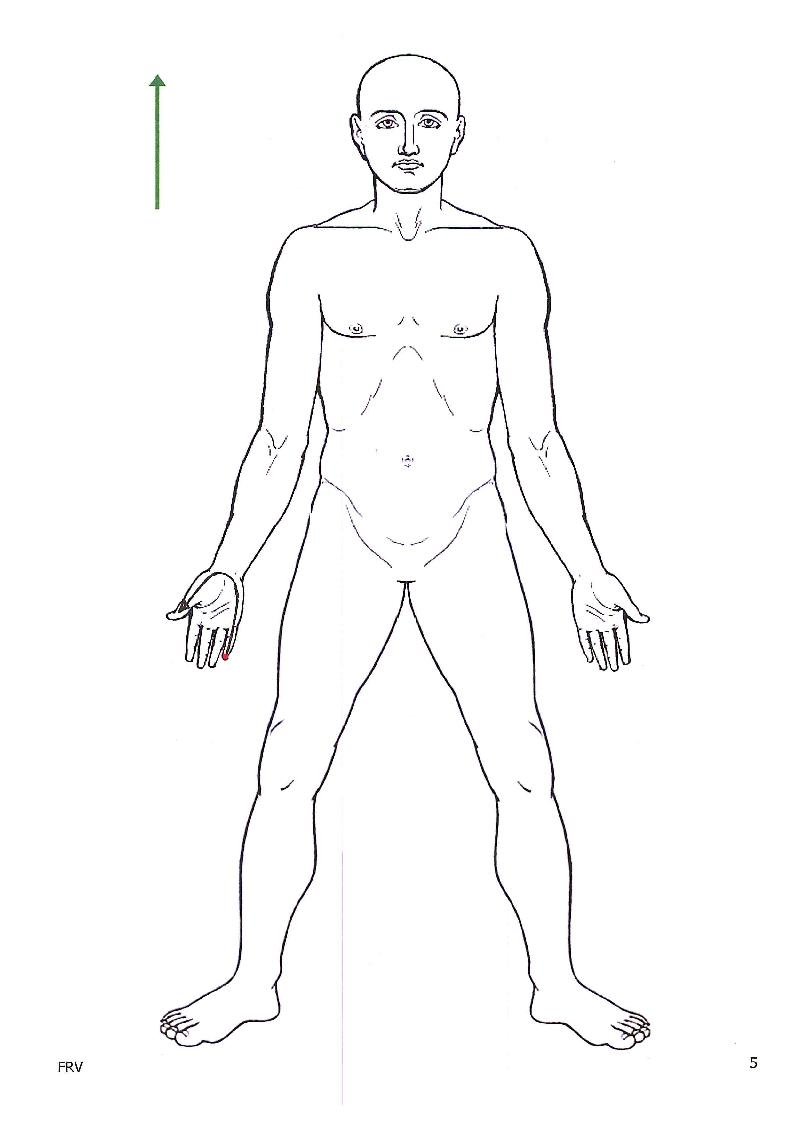

Supplement: S1 Raw Data — (ZIP) [file pone.0124808.s006.zip › Drawings - Imagined stimulation/finger_front/Subject_20_finger_front.jpg]
