# Supplementary figures and images for: Placebo-Induced Somatic Sensations: A Multi-Modal Study of Three Different Placebo Interventions (part 2 of 3)
Source: PLoS One. 2015 Apr 22;10(4):e0124808. doi: 10.1371/journal.pone.0124808 (PMC4406515; doi:10.1371/journal.pone.0124808)

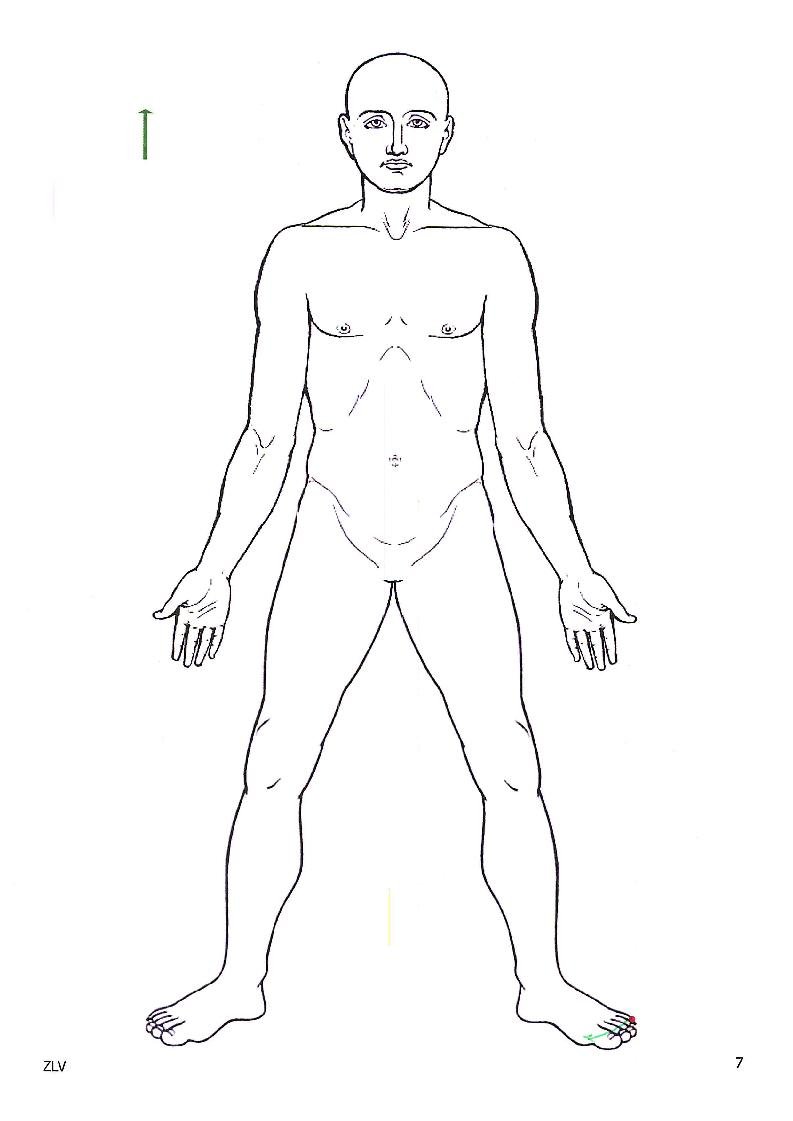

Supplement: S1 Raw Data — (ZIP) [file pone.0124808.s006.zip › Drawings - Imagined stimulation/toe_front/Subject_30_toe_front.jpg]

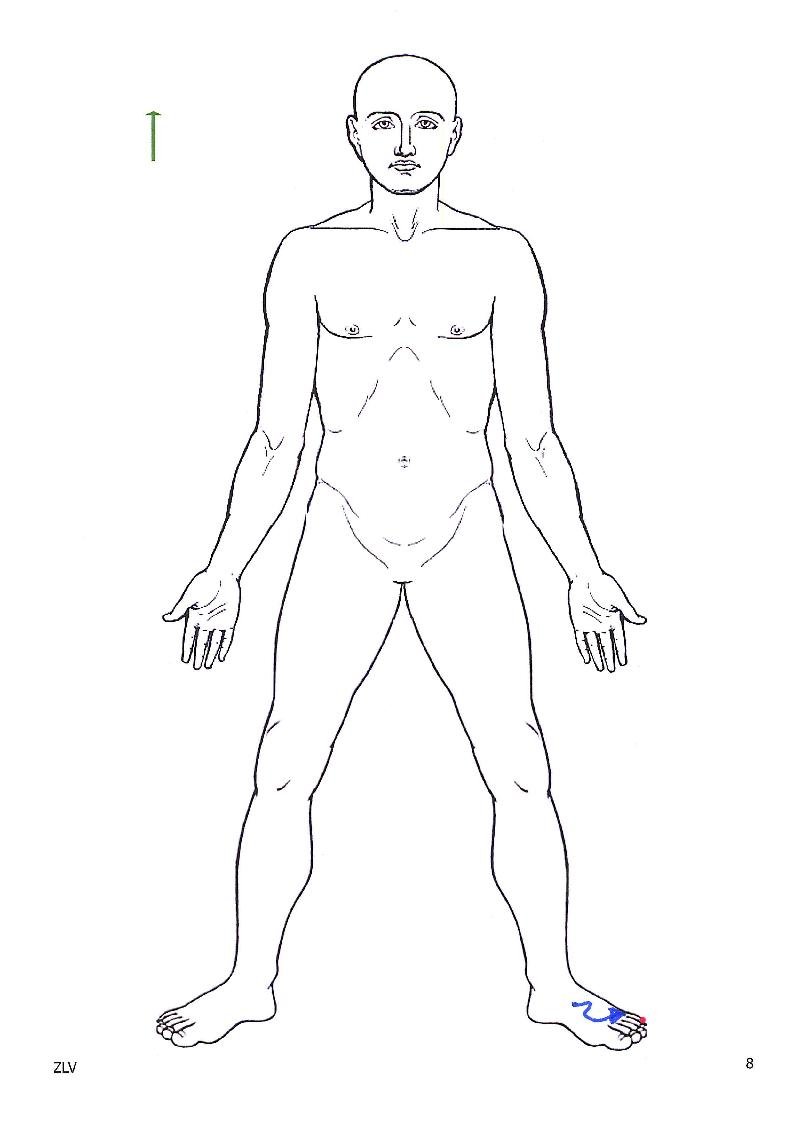

Supplement: S1 Raw Data — (ZIP) [file pone.0124808.s006.zip › Drawings - Imagined stimulation/toe_front/Subject_14_toe_front.jpg]

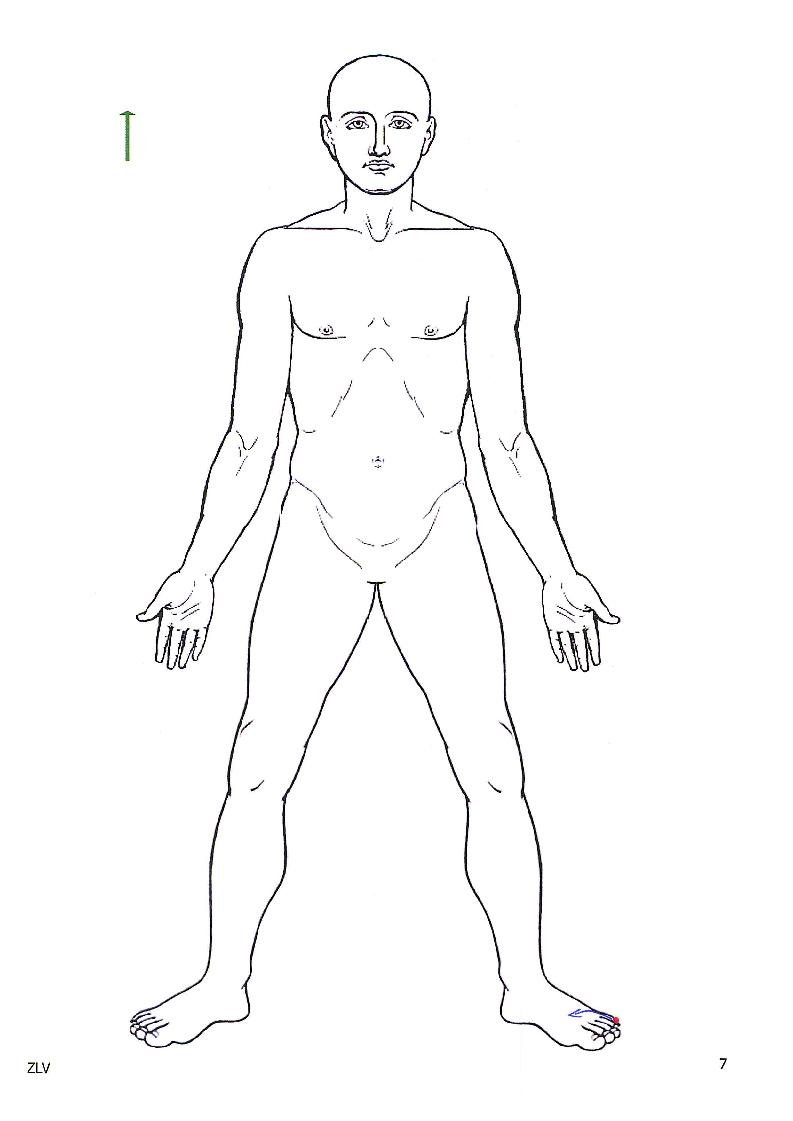

Supplement: S1 Raw Data — (ZIP) [file pone.0124808.s006.zip › Drawings - Imagined stimulation/toe_front/Subject_3_toe_front.jpg]

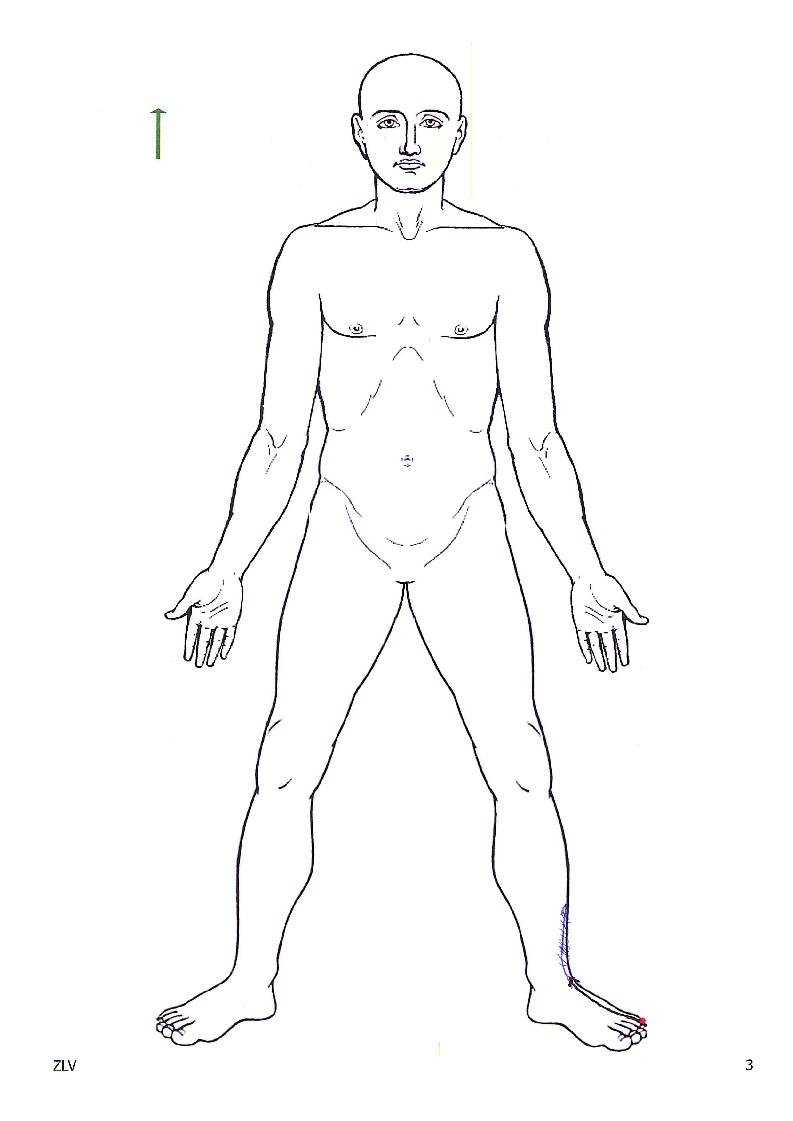

Supplement: S1 Raw Data — (ZIP) [file pone.0124808.s006.zip › Drawings - Imagined stimulation/toe_front/Subject_38_toe_front.jpg]

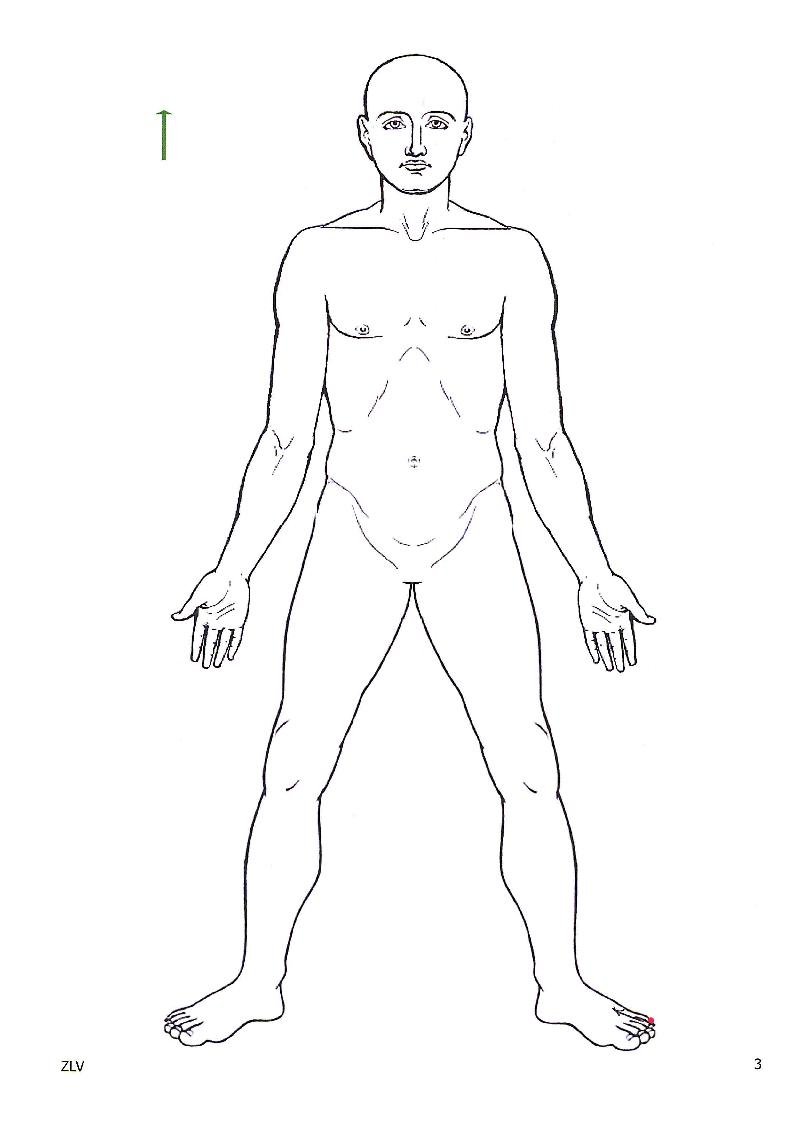

Supplement: S1 Raw Data — (ZIP) [file pone.0124808.s006.zip › Drawings - Imagined stimulation/toe_front/Subject_46_toe_front.jpg]

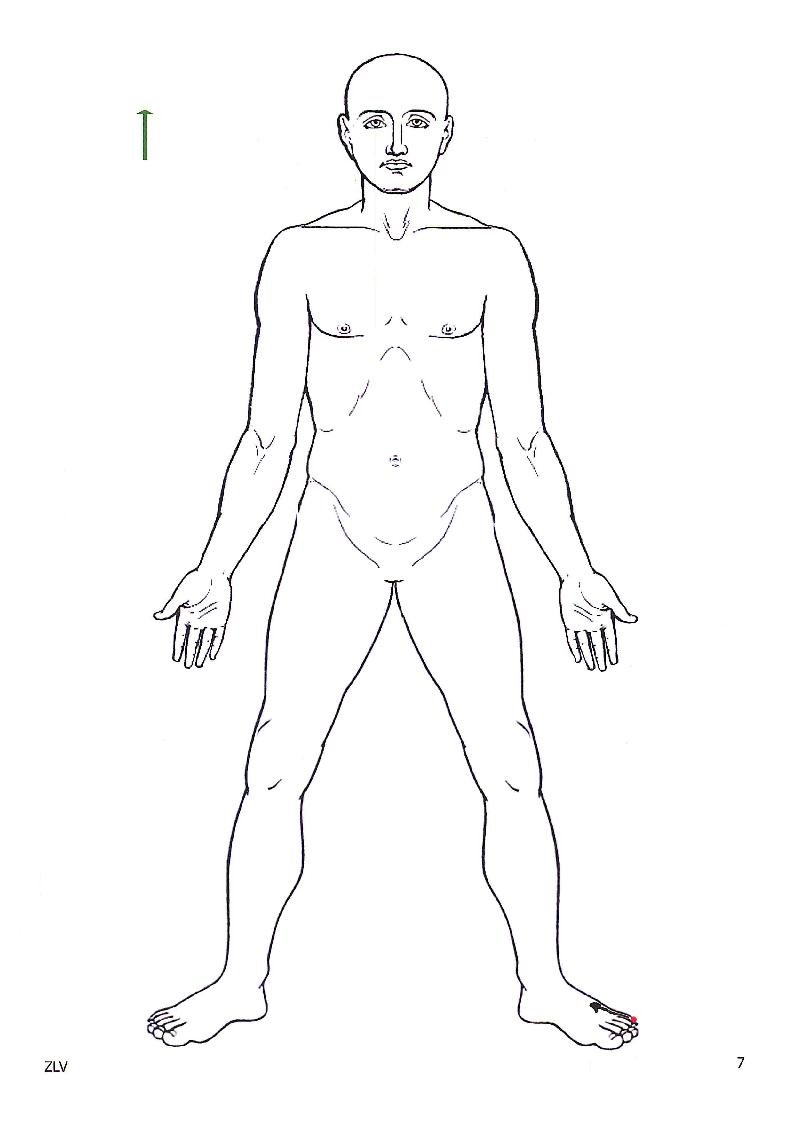

Supplement: S1 Raw Data — (ZIP) [file pone.0124808.s006.zip › Drawings - Imagined stimulation/toe_front/Subject_41_toe_front.jpg]

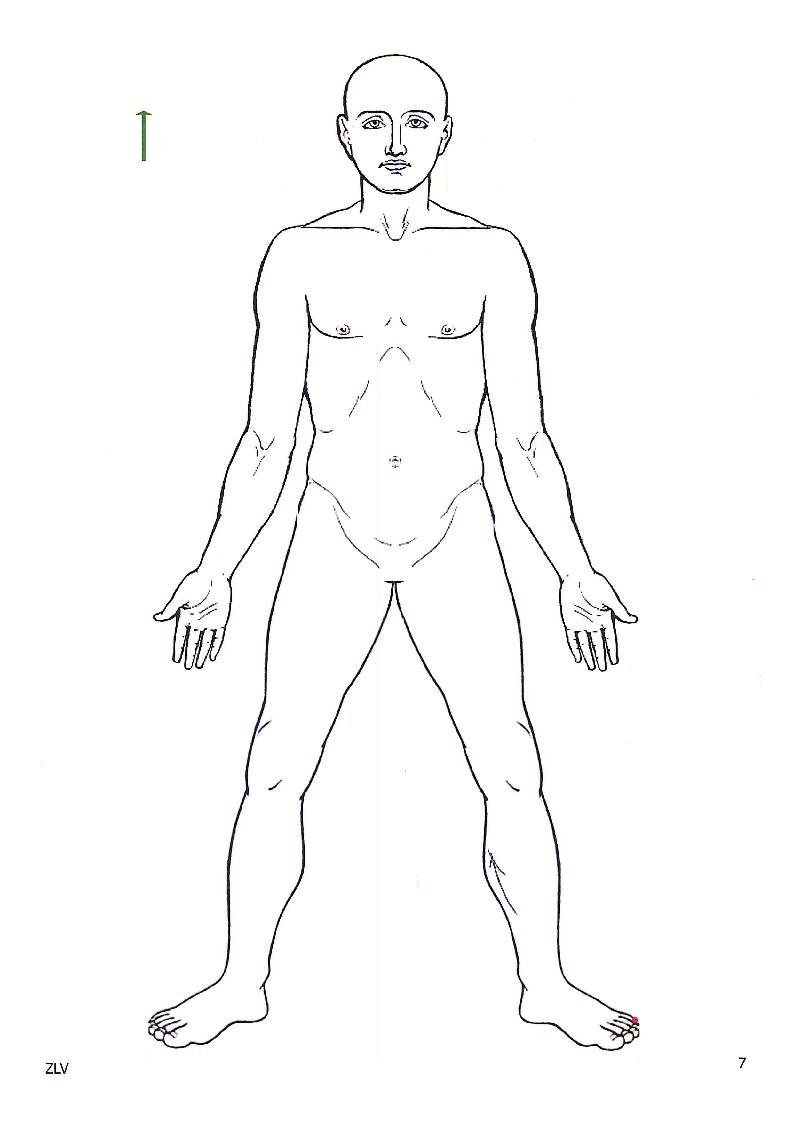

Supplement: S1 Raw Data — (ZIP) [file pone.0124808.s006.zip › Drawings - Imagined stimulation/toe_front/Subject_12_toe_front.jpg]

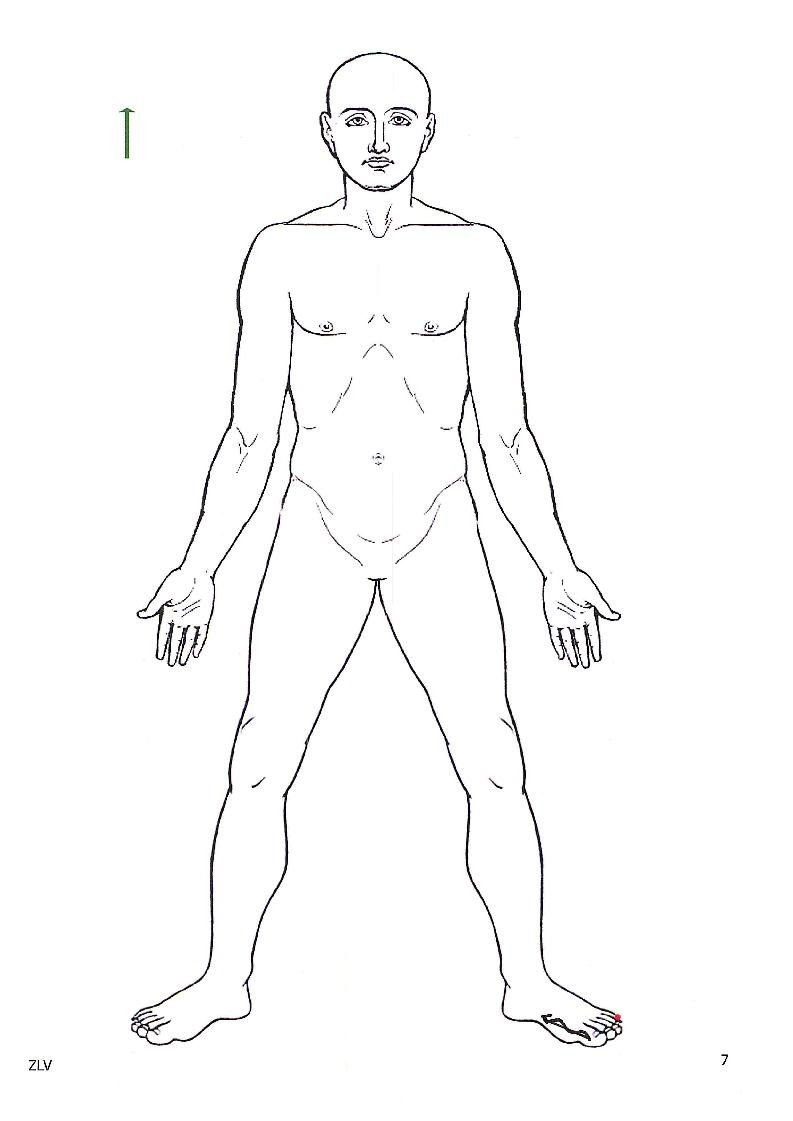

Supplement: S1 Raw Data — (ZIP) [file pone.0124808.s006.zip › Drawings - Imagined stimulation/toe_front/Subject_6_toe_front.jpg]

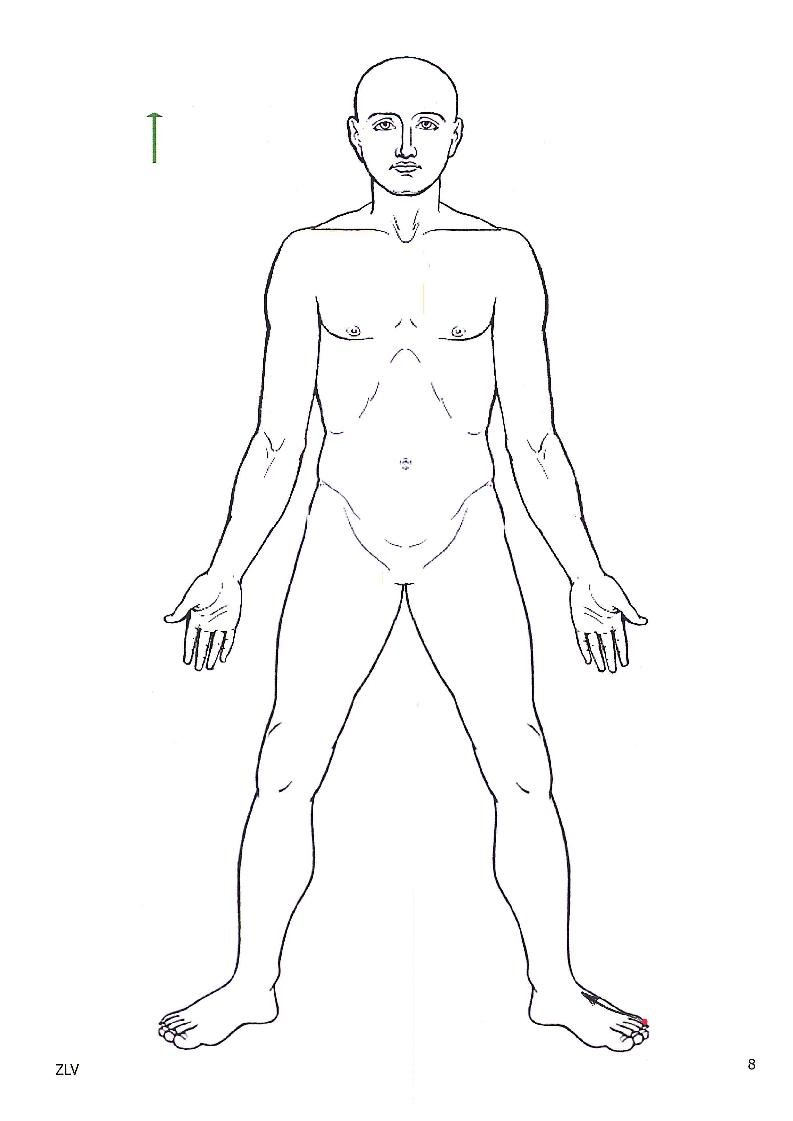

Supplement: S1 Raw Data — (ZIP) [file pone.0124808.s006.zip › Drawings - Imagined stimulation/toe_front/Subject_20_toe_front.jpg]

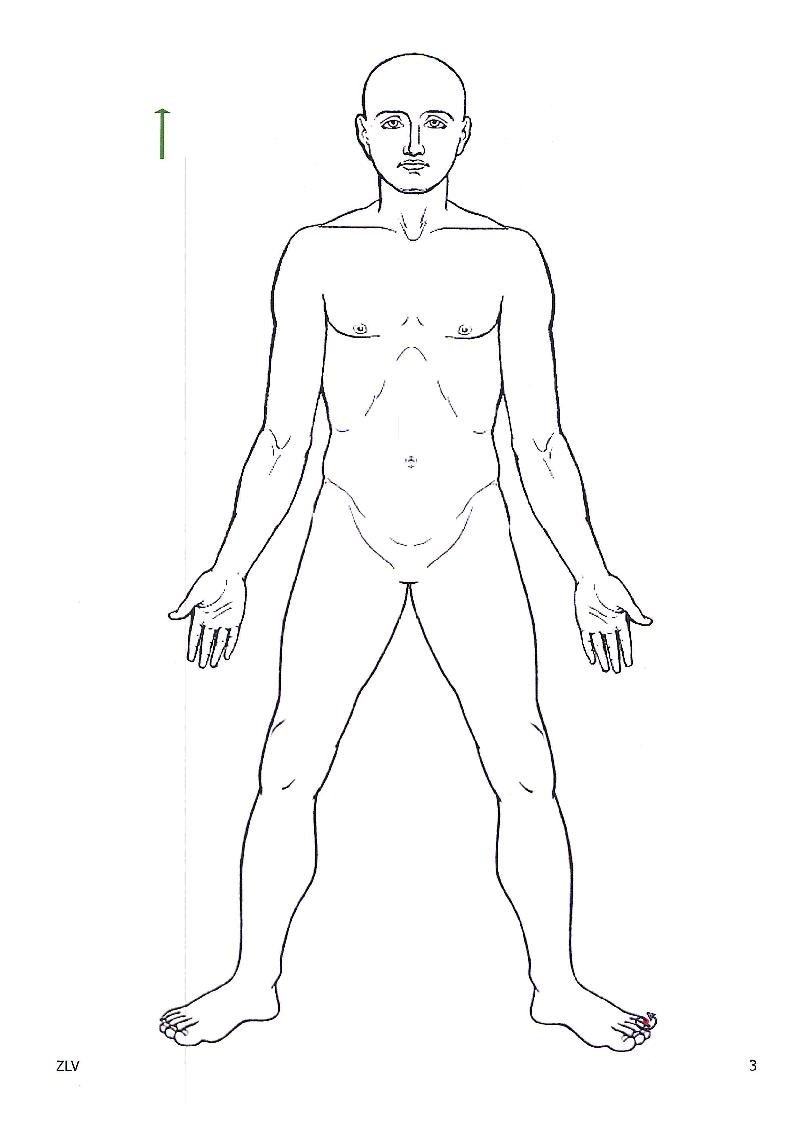

Supplement: S1 Raw Data — (ZIP) [file pone.0124808.s006.zip › Drawings - Imagined stimulation/toe_front/Subject_49_toe_front.jpg]

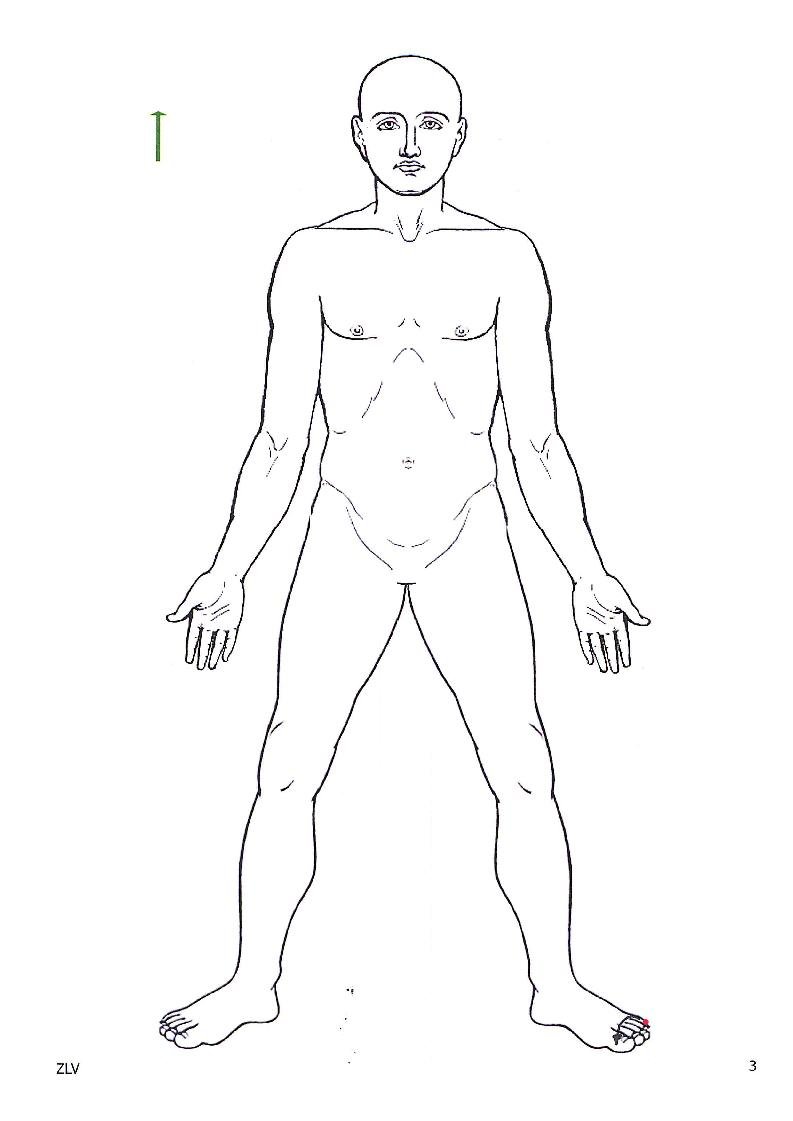

Supplement: S1 Raw Data — (ZIP) [file pone.0124808.s006.zip › Drawings - Imagined stimulation/toe_front/Subject_28_toe_front.jpg]

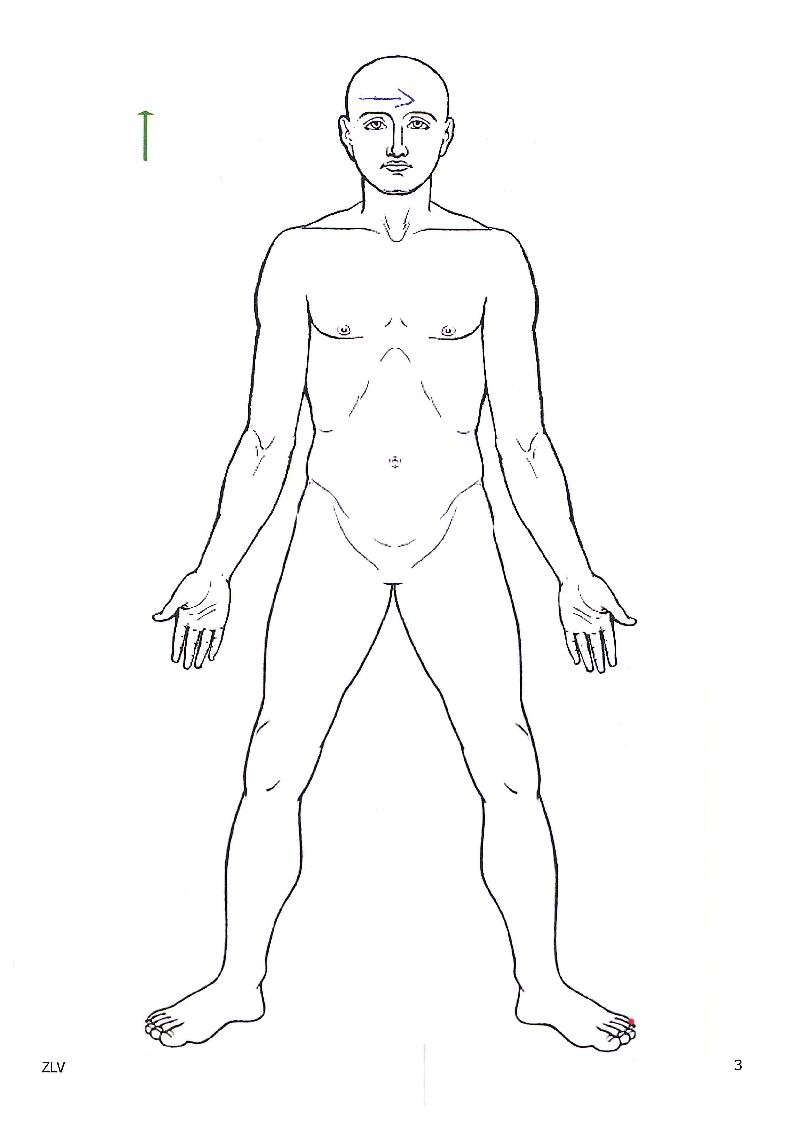

Supplement: S1 Raw Data — (ZIP) [file pone.0124808.s006.zip › Drawings - Imagined stimulation/toe_front/Subject_31_toe_front.jpg]

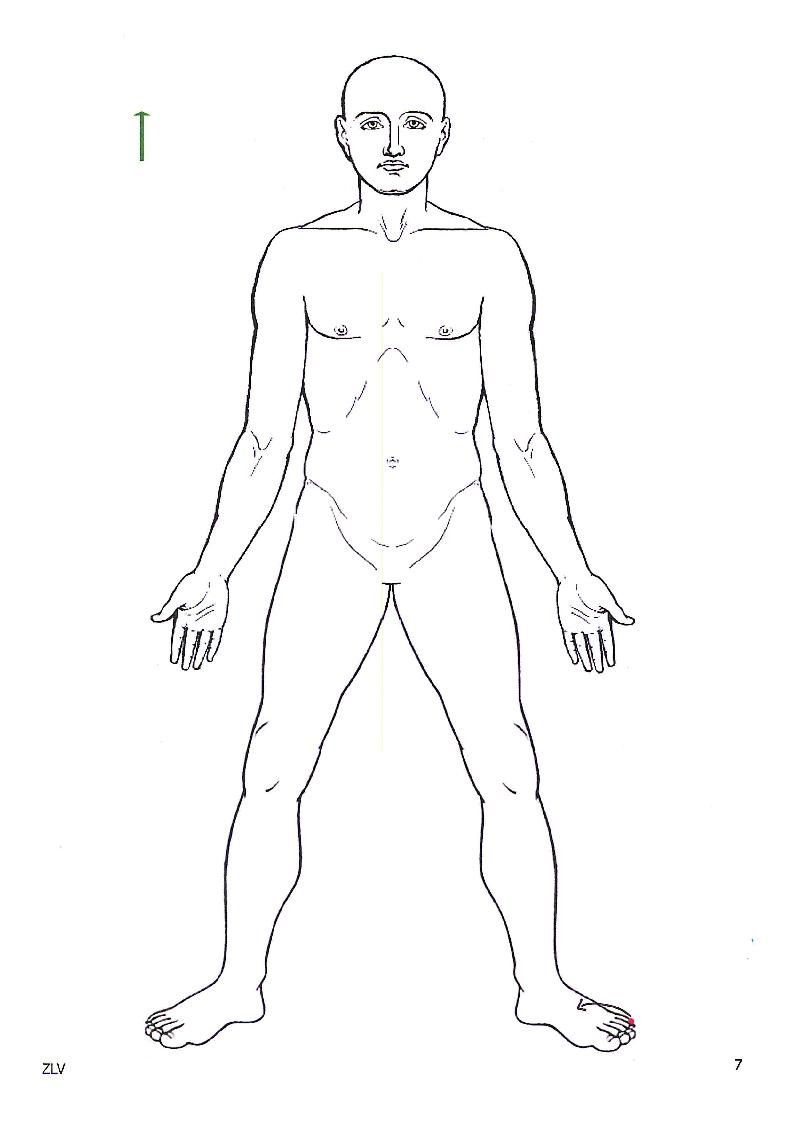

Supplement: S1 Raw Data — (ZIP) [file pone.0124808.s006.zip › Drawings - Imagined stimulation/toe_front/Subject_4_toe_front.jpg]

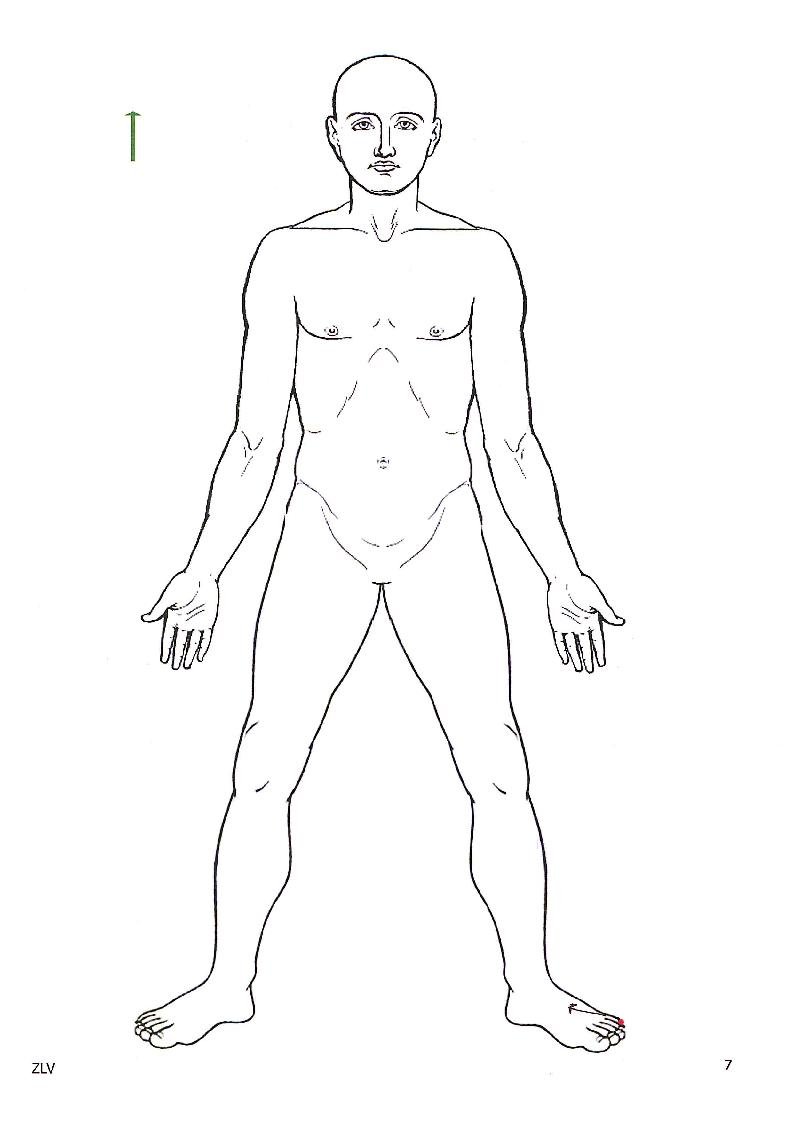

Supplement: S1 Raw Data — (ZIP) [file pone.0124808.s006.zip › Drawings - Imagined stimulation/toe_front/Subject_39_toe_front.jpg]

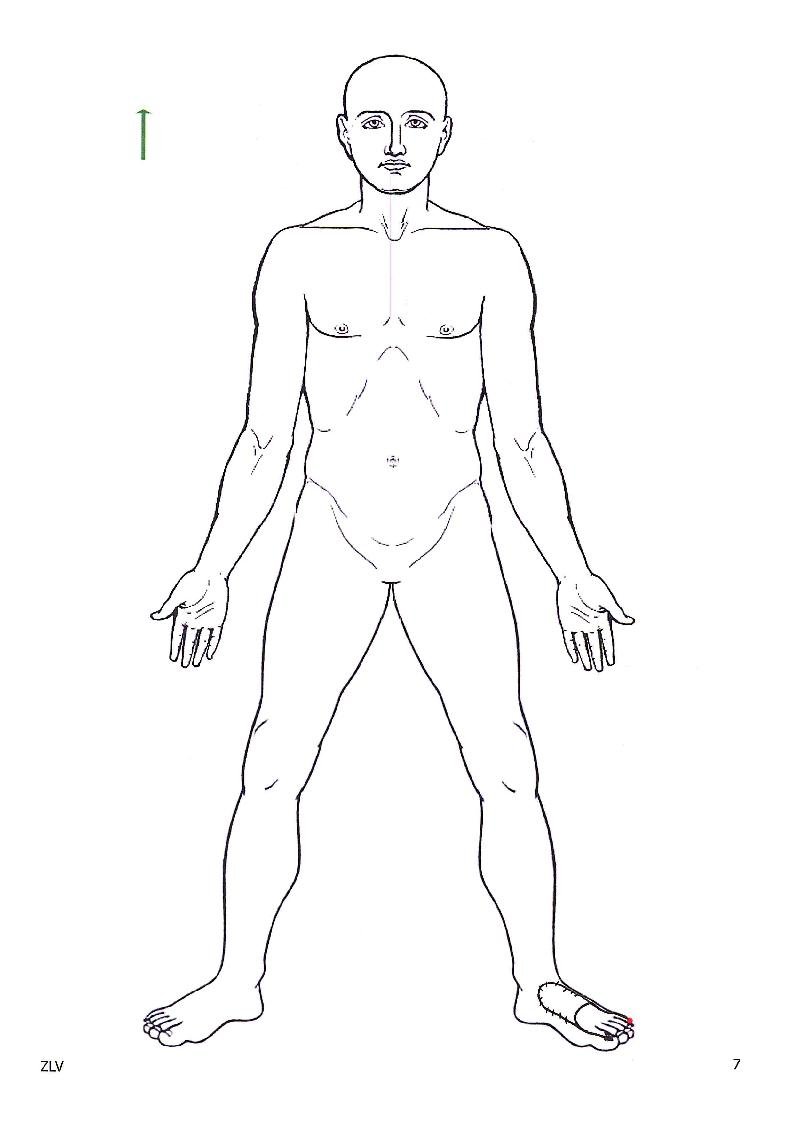

Supplement: S1 Raw Data — (ZIP) [file pone.0124808.s006.zip › Drawings - Imagined stimulation/toe_front/Subject_47_toe_front.jpg]

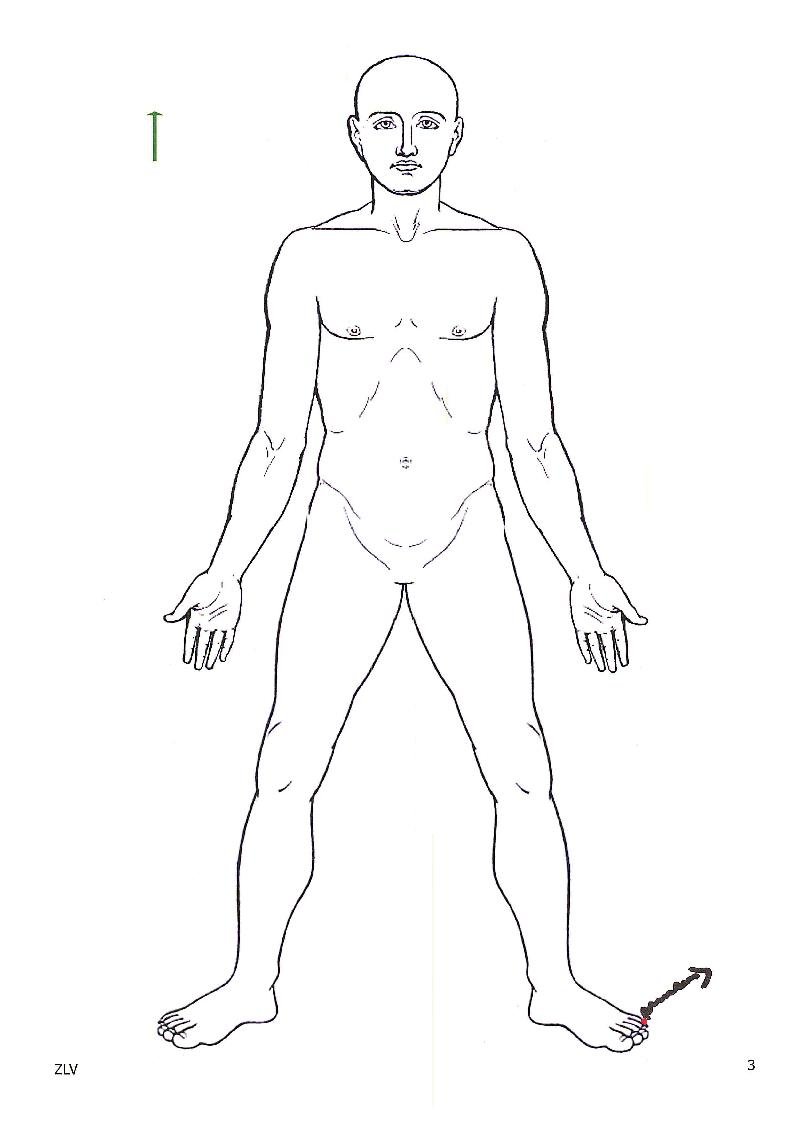

Supplement: S1 Raw Data — (ZIP) [file pone.0124808.s006.zip › Drawings - Imagined stimulation/toe_front/Subject_55_toe_front.jpg]

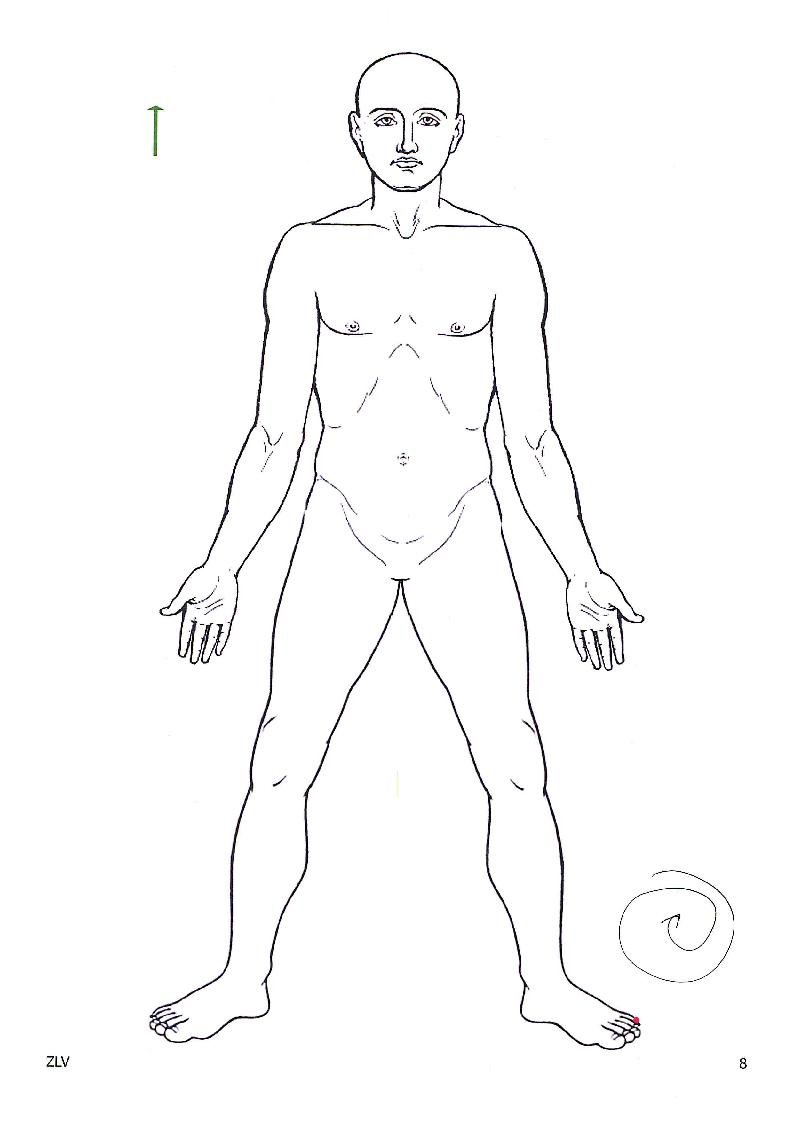

Supplement: S1 Raw Data — (ZIP) [file pone.0124808.s006.zip › Drawings - Imagined stimulation/toe_front/Subject_10_toe_front.jpg]

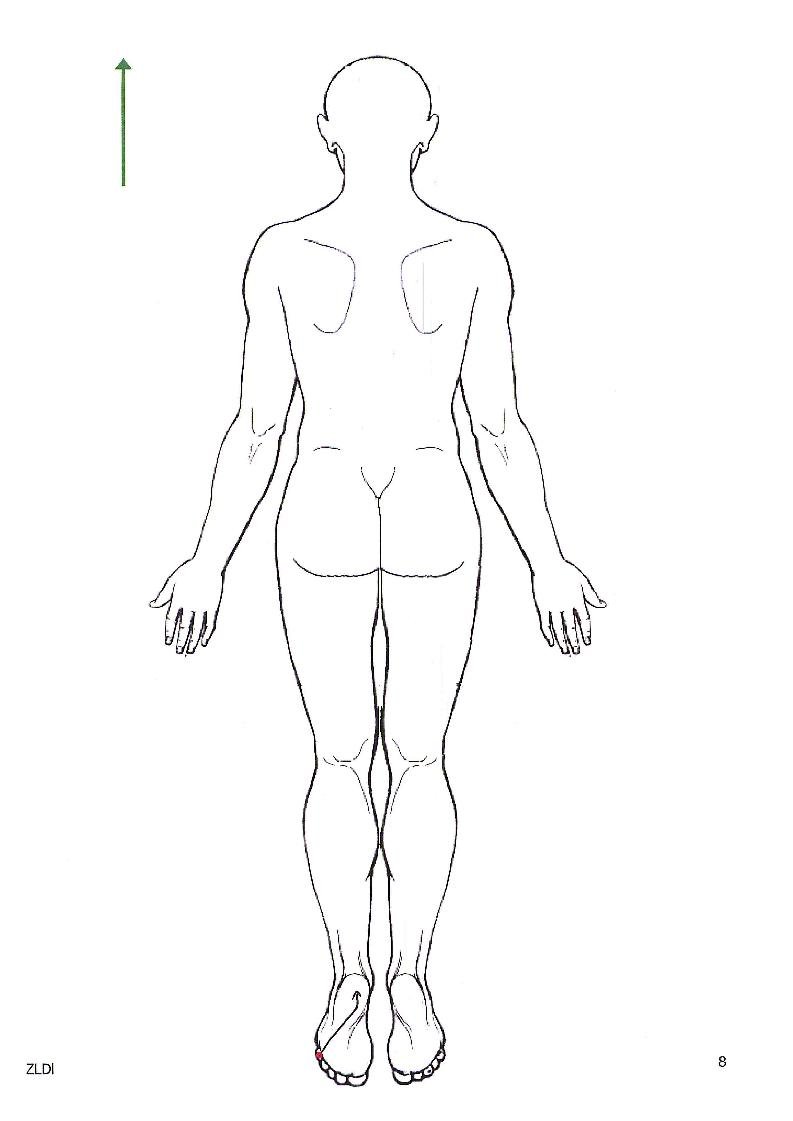

Supplement: S1 Raw Data — (ZIP) [file pone.0124808.s006.zip › Drawings - Imagined stimulation/toe_back/Subject_40_toe_back.jpg]

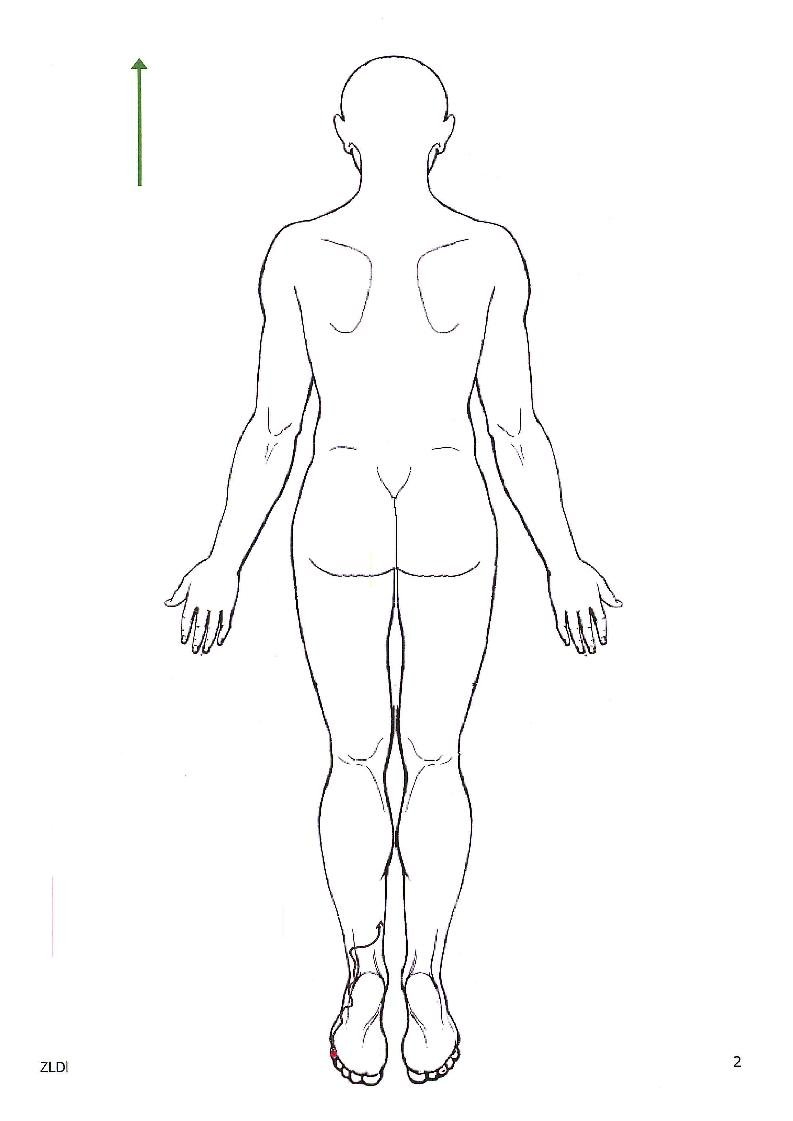

Supplement: S1 Raw Data — (ZIP) [file pone.0124808.s006.zip › Drawings - Imagined stimulation/toe_back/Subject_45_toe_back.jpg]

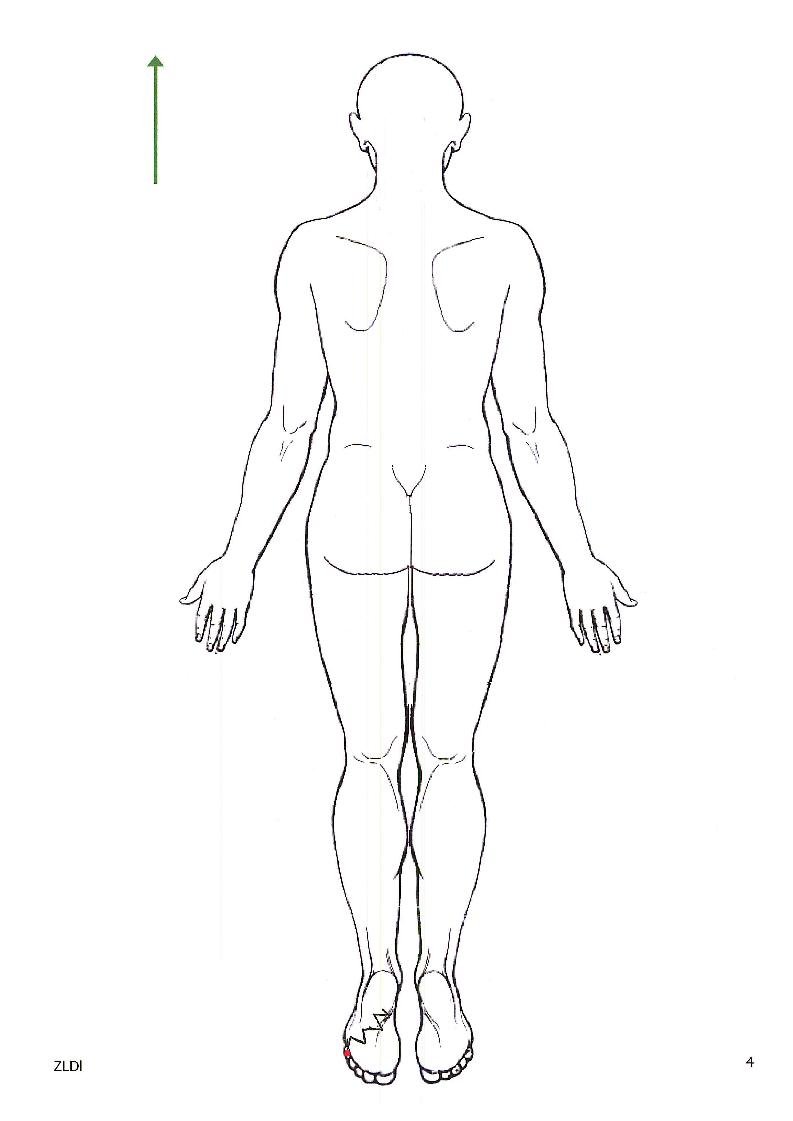

Supplement: S1 Raw Data — (ZIP) [file pone.0124808.s006.zip › Drawings - Imagined stimulation/toe_back/Subject_33_toe_back.jpg]

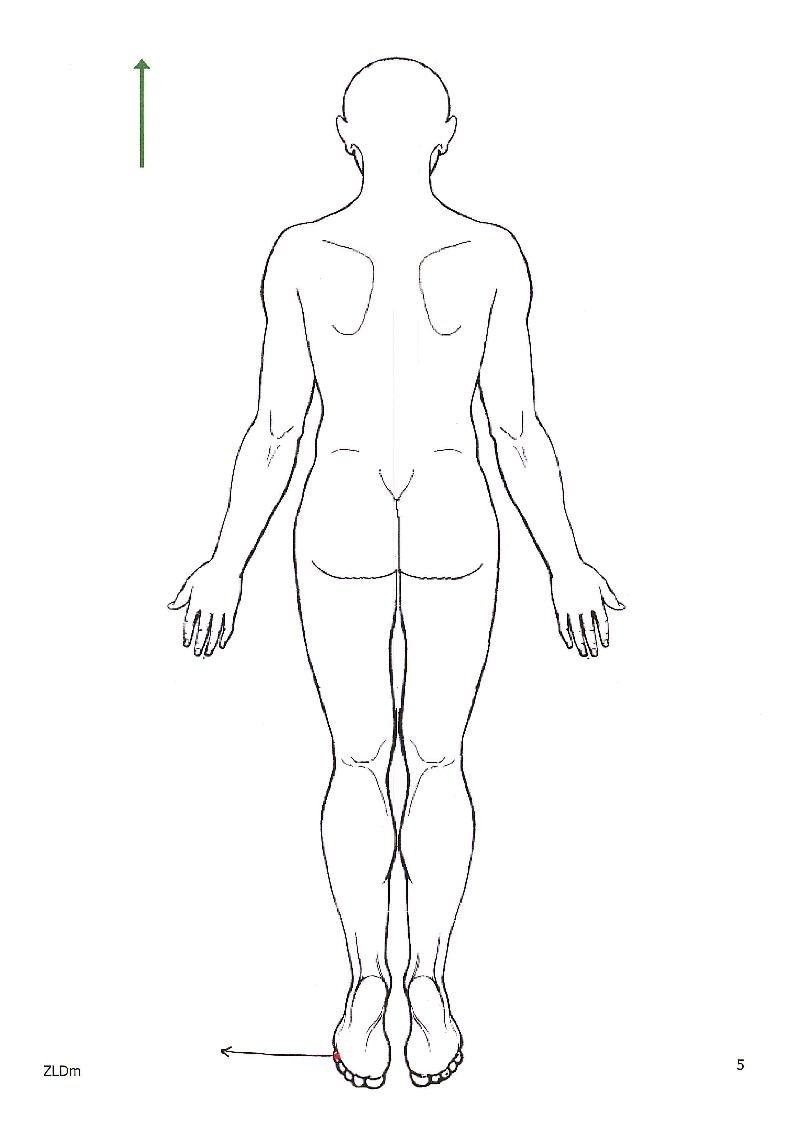

Supplement: S1 Raw Data — (ZIP) [file pone.0124808.s006.zip › Drawings - Imagined stimulation/toe_back/Subject_4_toe_back.jpg]

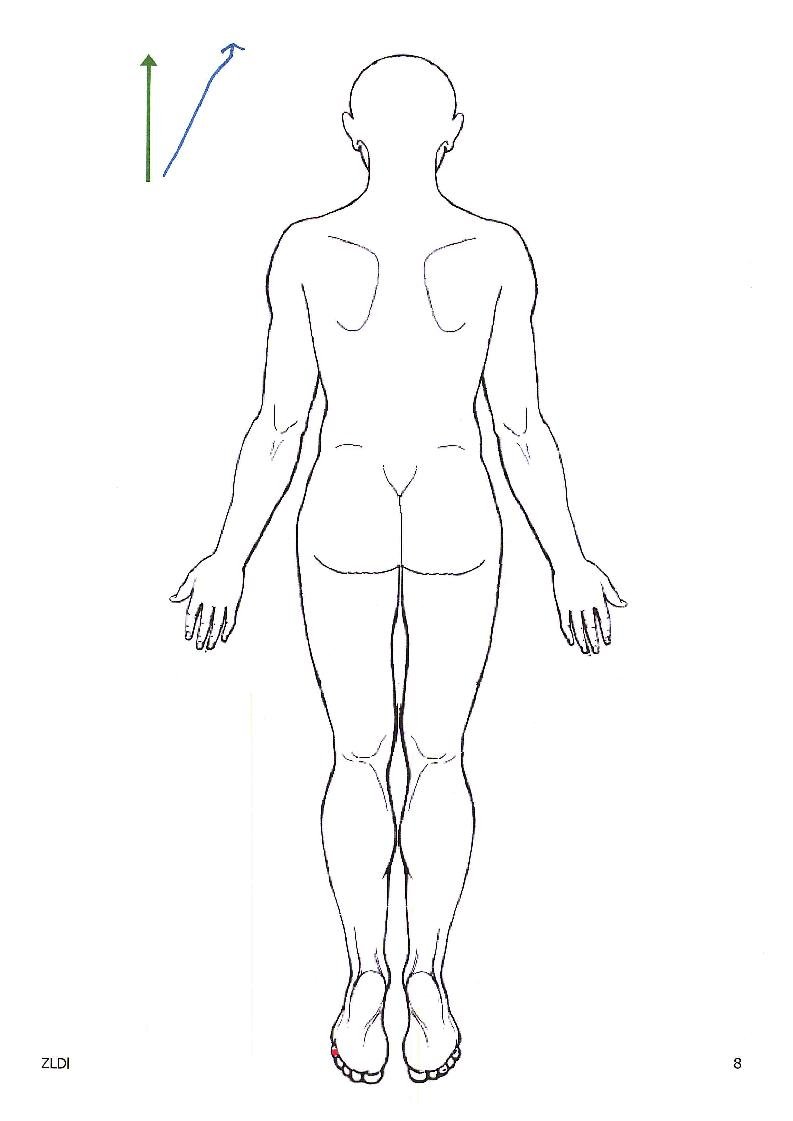

Supplement: S1 Raw Data — (ZIP) [file pone.0124808.s006.zip › Drawings - Imagined stimulation/toe_back/Subject_56_toe_back.jpg]

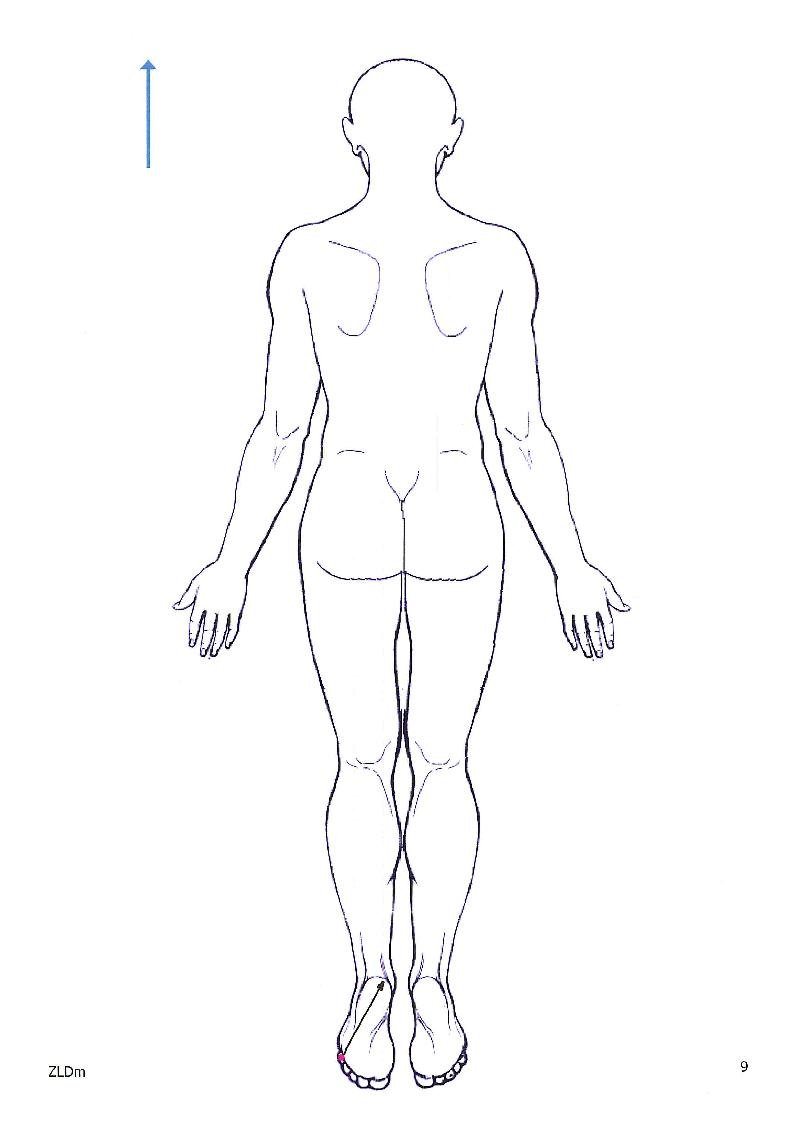

Supplement: S1 Raw Data — (ZIP) [file pone.0124808.s006.zip › Drawings - Imagined stimulation/toe_back/Subject_27_toe_back.jpg]

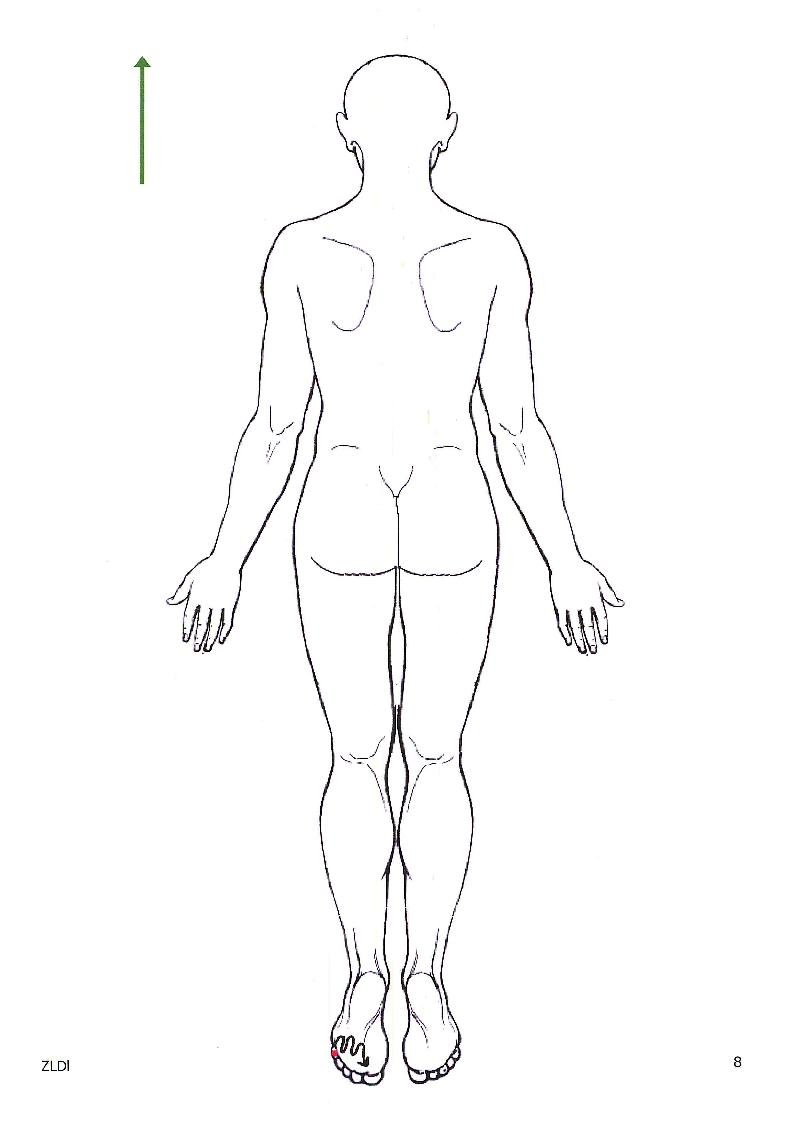

Supplement: S1 Raw Data — (ZIP) [file pone.0124808.s006.zip › Drawings - Imagined stimulation/toe_back/Subject_41_toe_back.jpg]

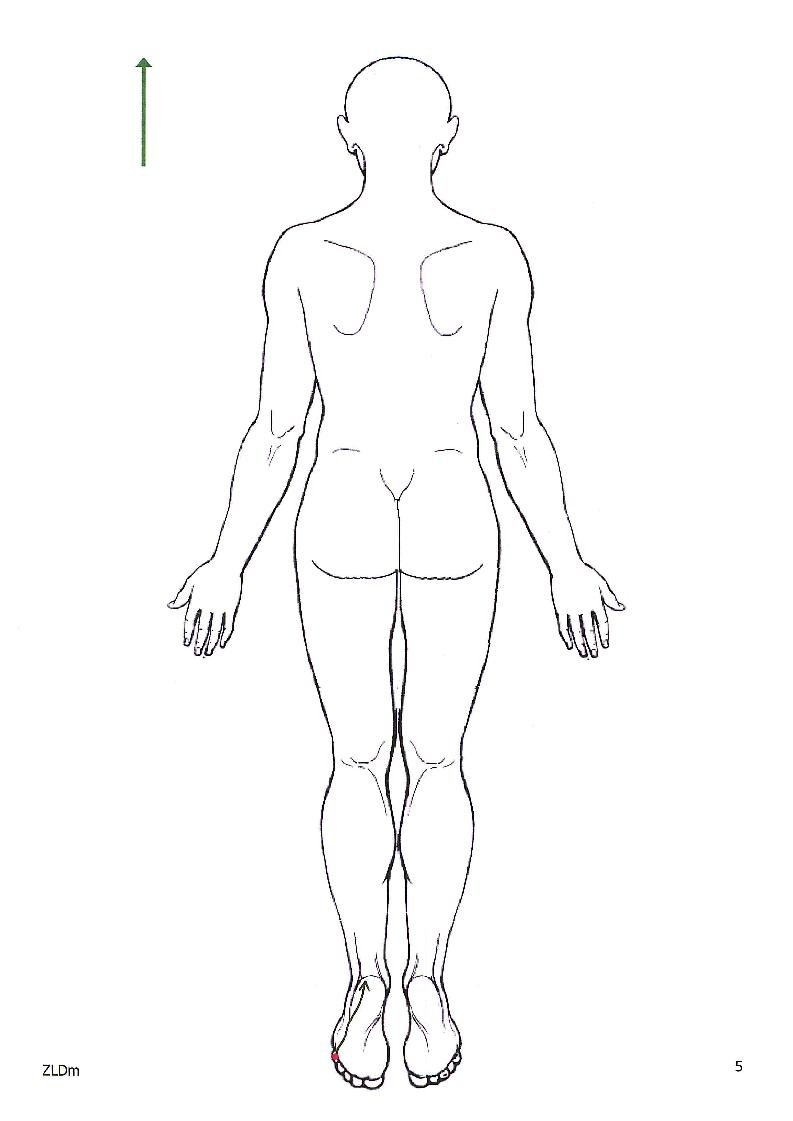

Supplement: S1 Raw Data — (ZIP) [file pone.0124808.s006.zip › Drawings - Imagined stimulation/toe_back/Subject_7_toe_back.jpg]

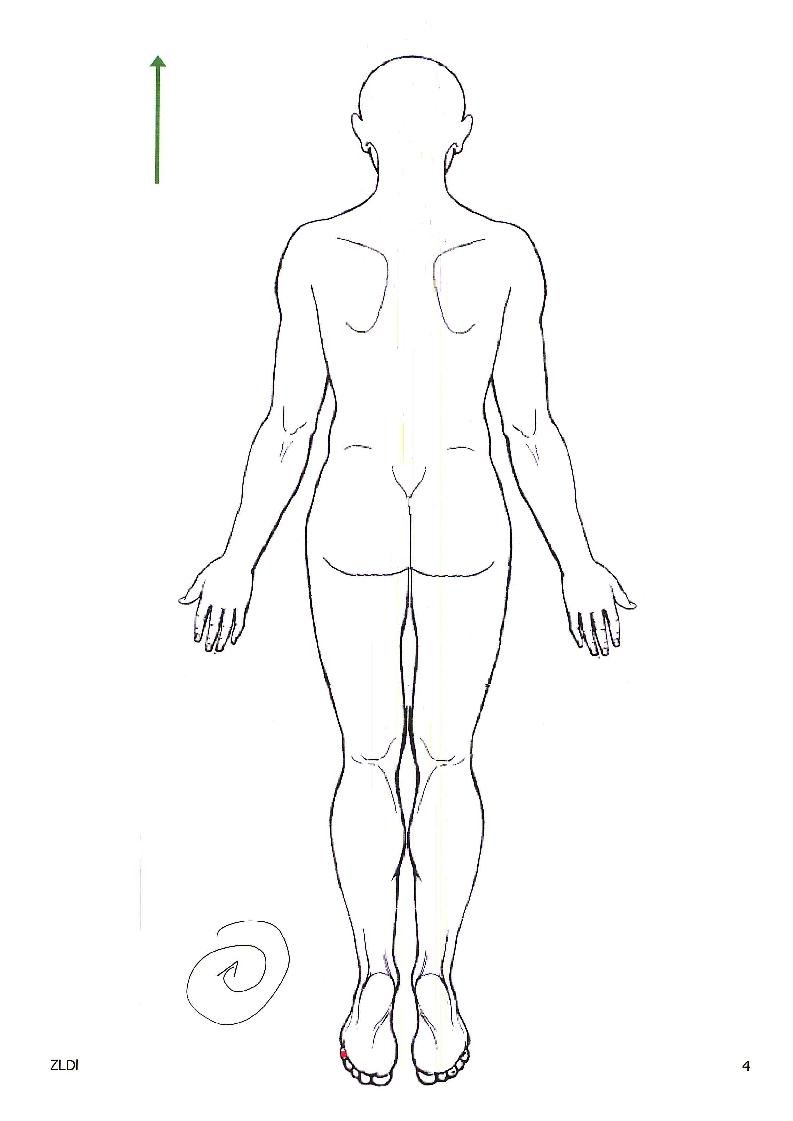

Supplement: S1 Raw Data — (ZIP) [file pone.0124808.s006.zip › Drawings - Imagined stimulation/toe_back/Subject_10_toe_back.jpg]

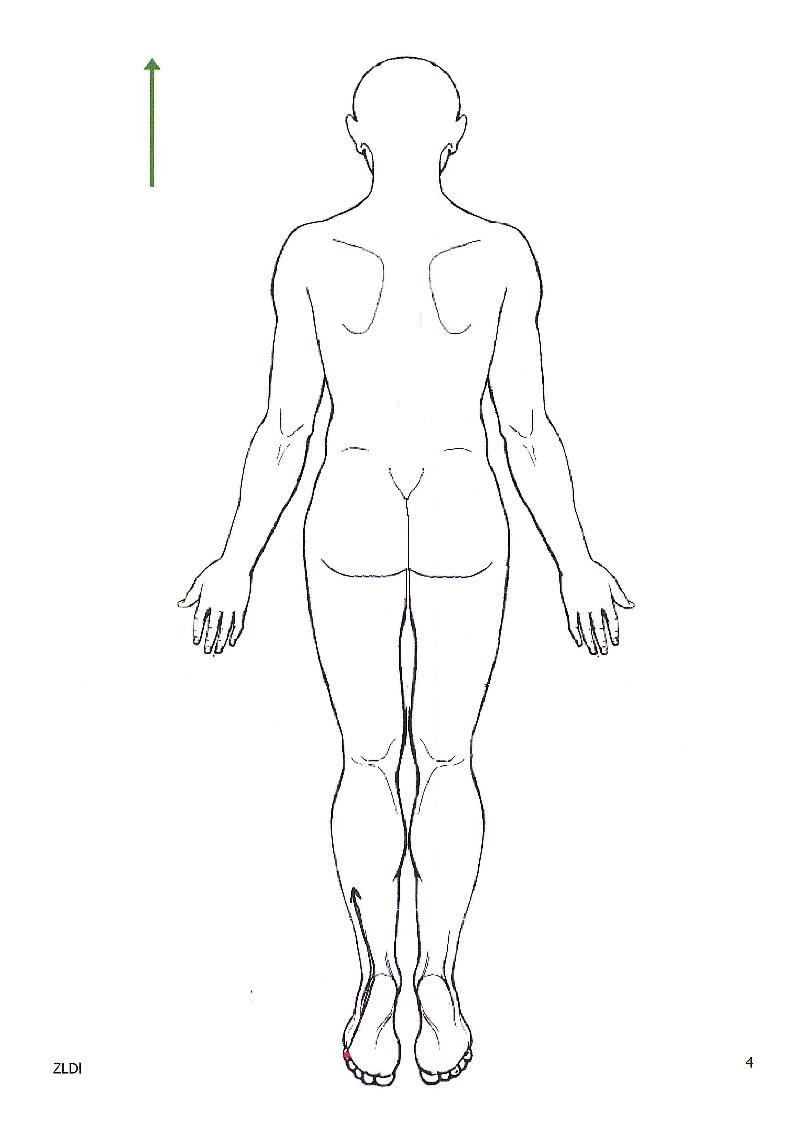

Supplement: S1 Raw Data — (ZIP) [file pone.0124808.s006.zip › Drawings - Imagined stimulation/toe_back/Subject_43_toe_back.jpg]

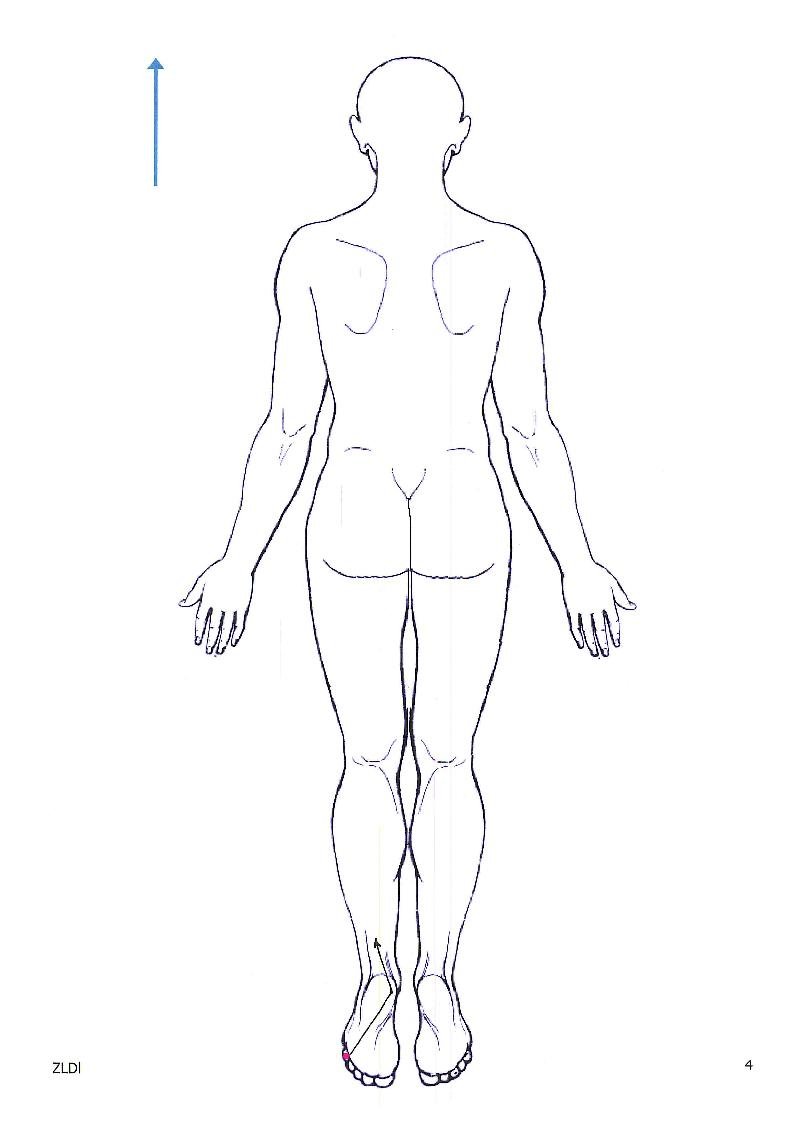

Supplement: S1 Raw Data — (ZIP) [file pone.0124808.s006.zip › Drawings - Imagined stimulation/toe_back/Subject_15_toe_back.jpg]

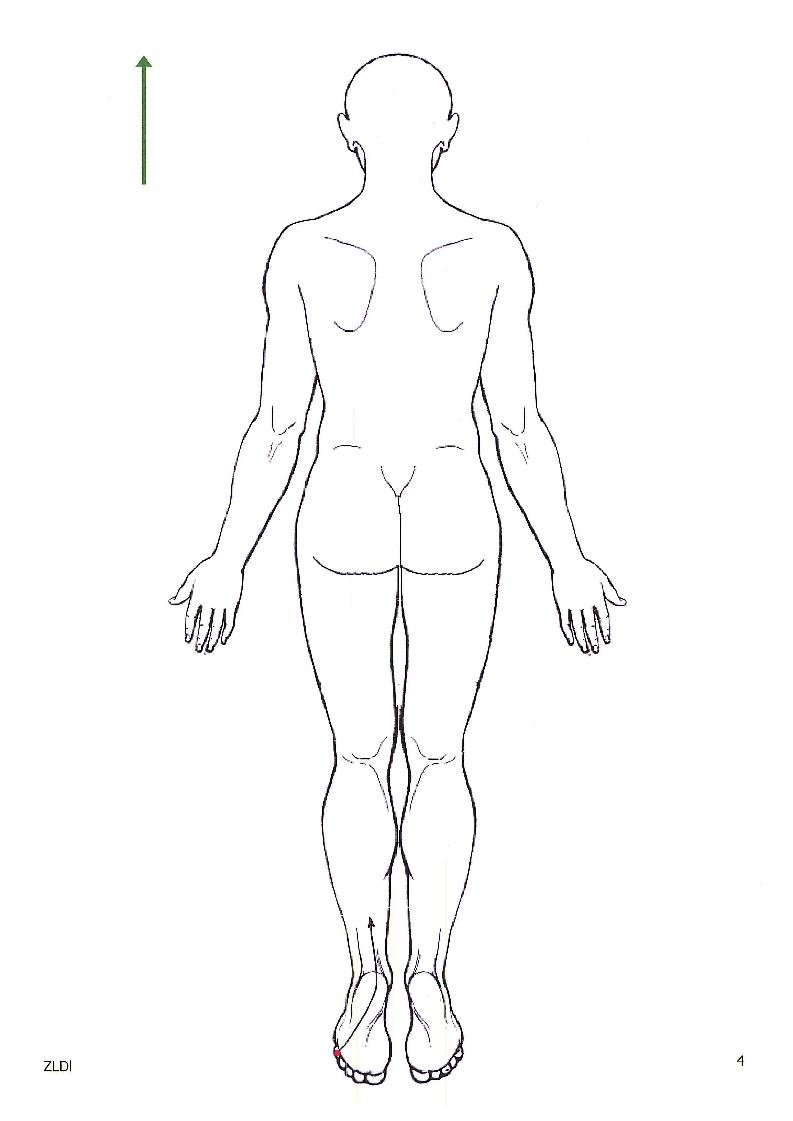

Supplement: S1 Raw Data — (ZIP) [file pone.0124808.s006.zip › Drawings - Imagined stimulation/toe_back/Subject_51_toe_back.jpg]

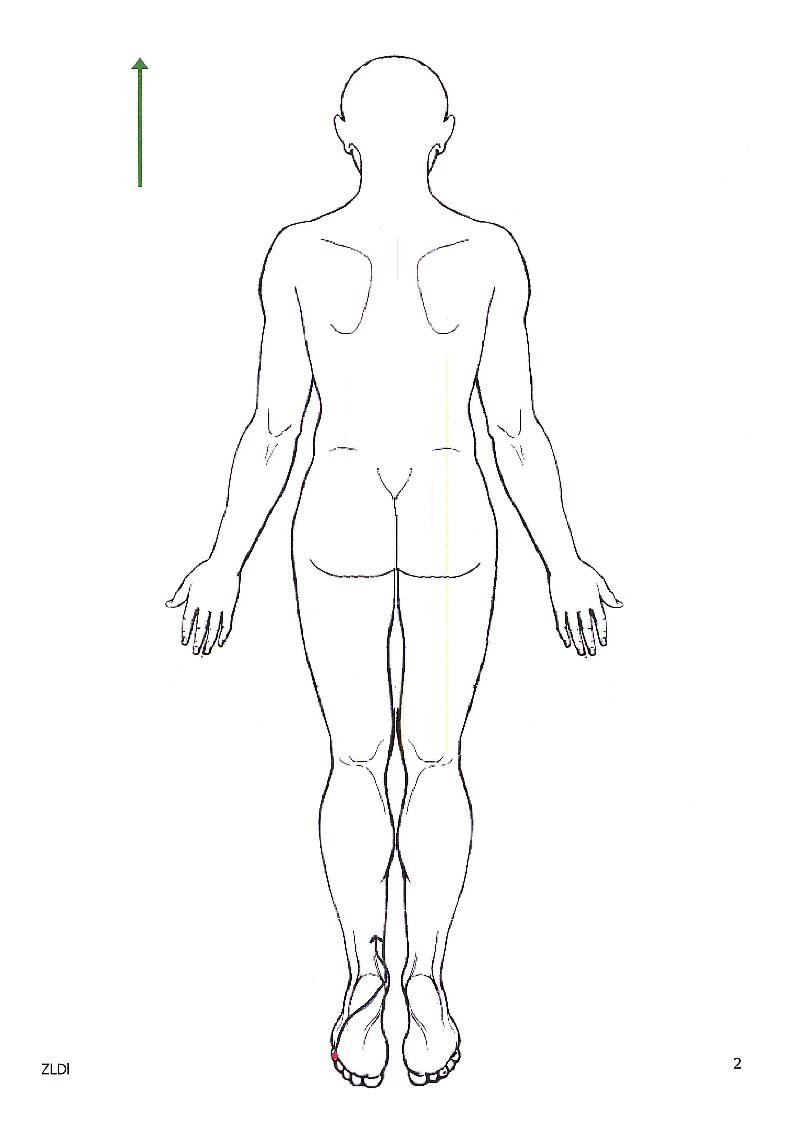

Supplement: S1 Raw Data — (ZIP) [file pone.0124808.s006.zip › Drawings - Imagined stimulation/toe_back/Subject_38_toe_back.jpg]

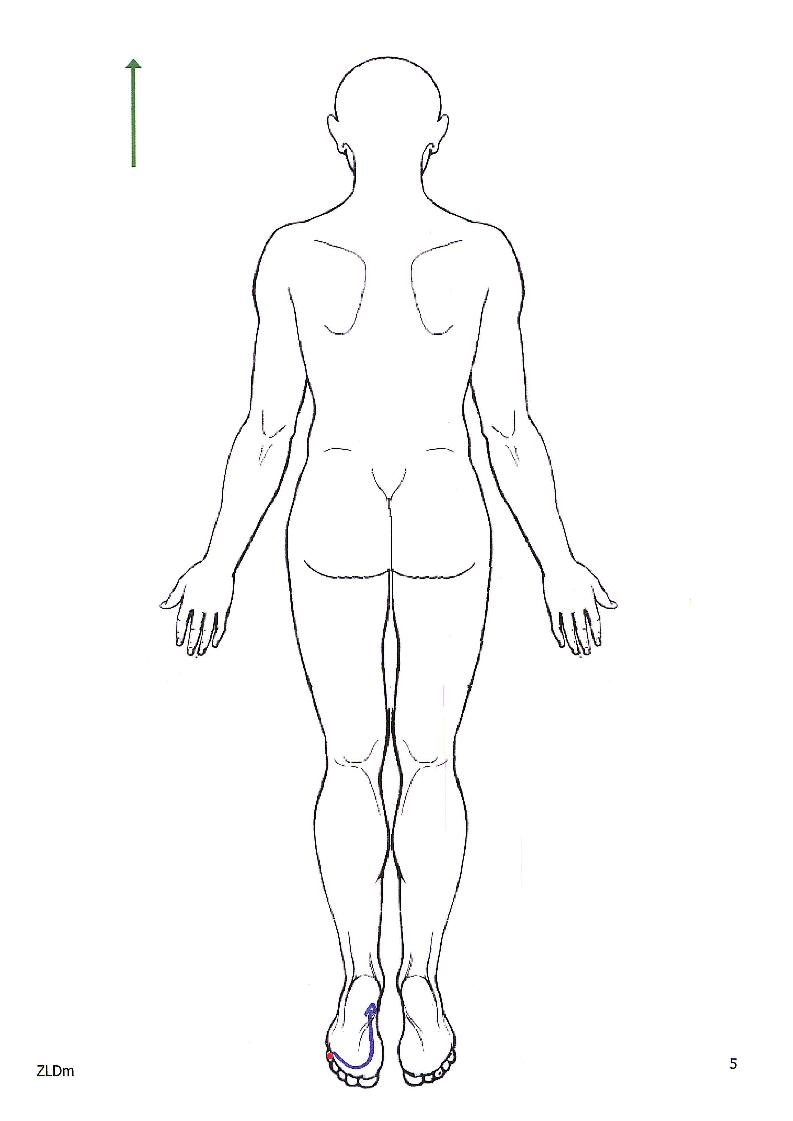

Supplement: S1 Raw Data — (ZIP) [file pone.0124808.s006.zip › Drawings - Imagined stimulation/toe_back/Subject_36_toe_back.jpg]

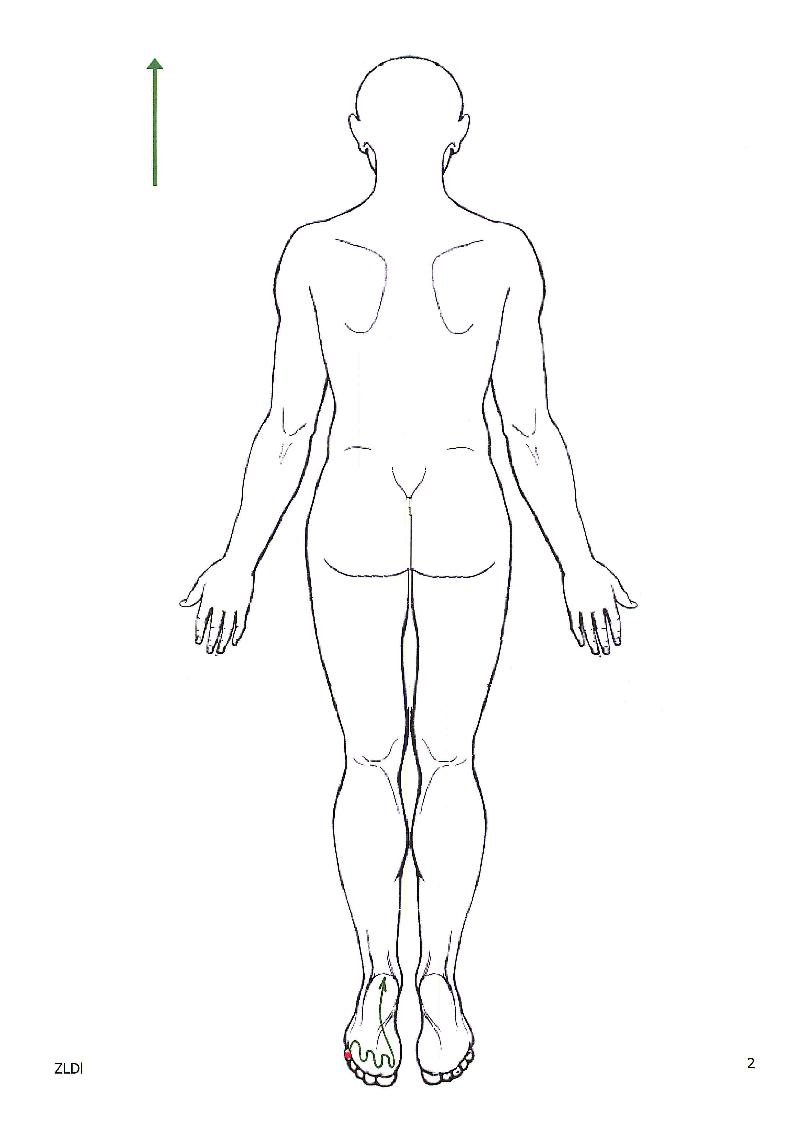

Supplement: S1 Raw Data — (ZIP) [file pone.0124808.s006.zip › Drawings - Imagined stimulation/toe_back/Subject_13_toe_back.jpg]

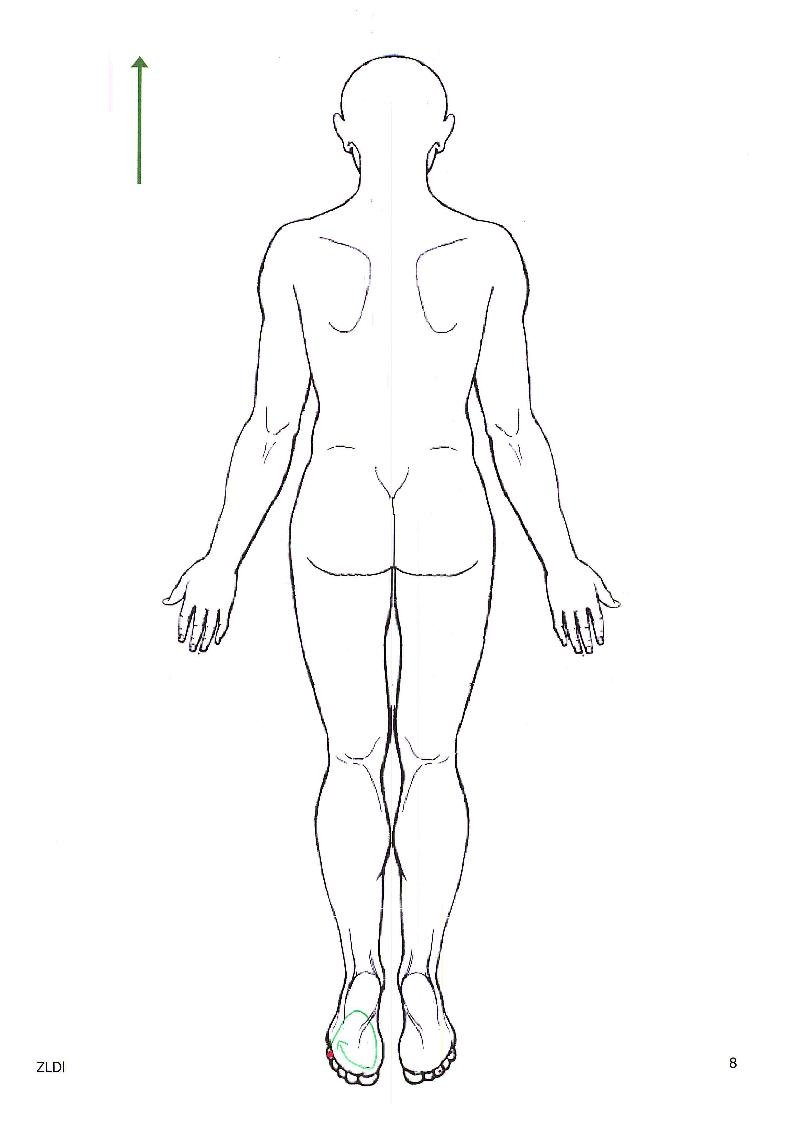

Supplement: S1 Raw Data — (ZIP) [file pone.0124808.s006.zip › Drawings - Imagined stimulation/toe_back/Subject_30_toe_back.jpg]

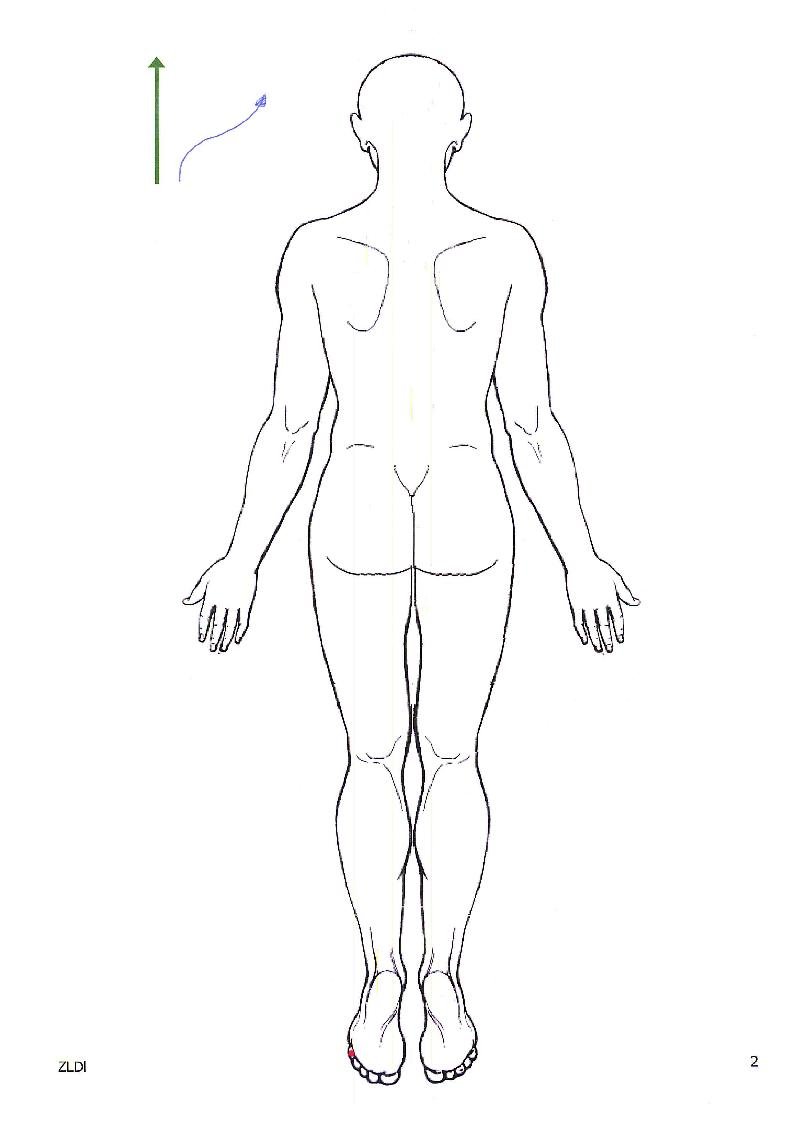

Supplement: S1 Raw Data — (ZIP) [file pone.0124808.s006.zip › Drawings - Imagined stimulation/toe_back/Subject_21_toe_back.jpg]

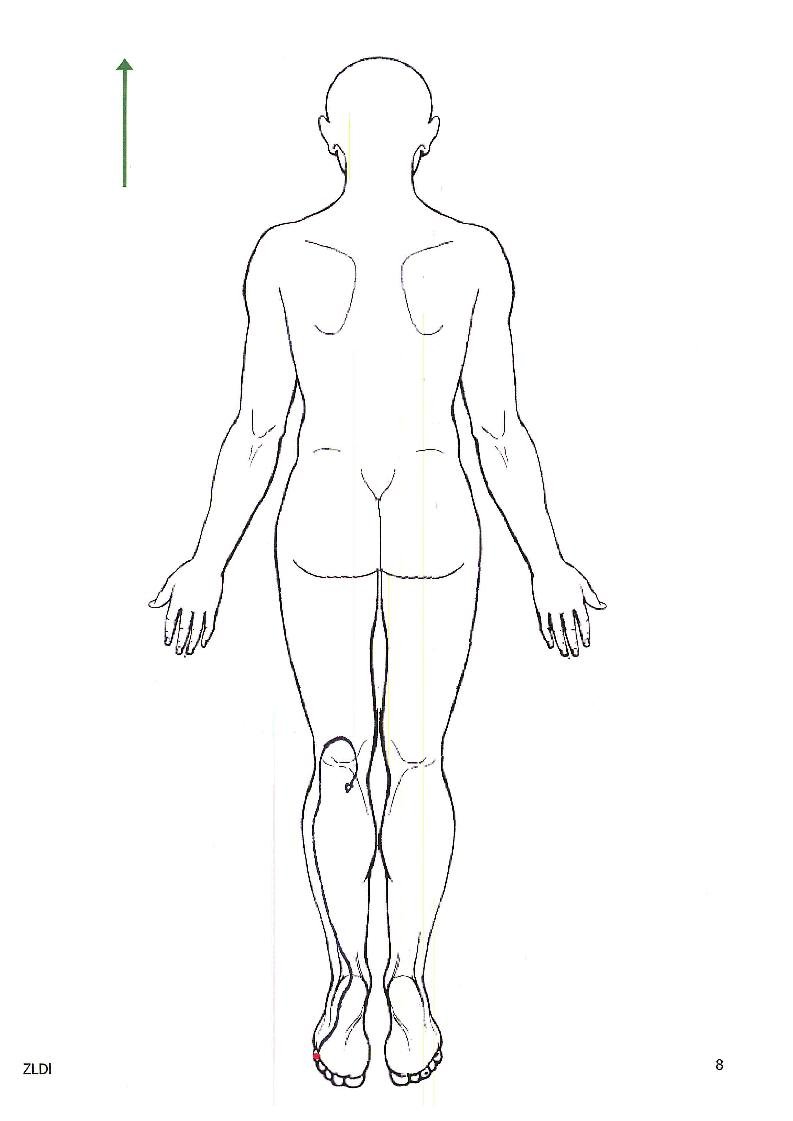

Supplement: S1 Raw Data — (ZIP) [file pone.0124808.s006.zip › Drawings - Imagined stimulation/toe_back/Subject_9_toe_back.jpg]

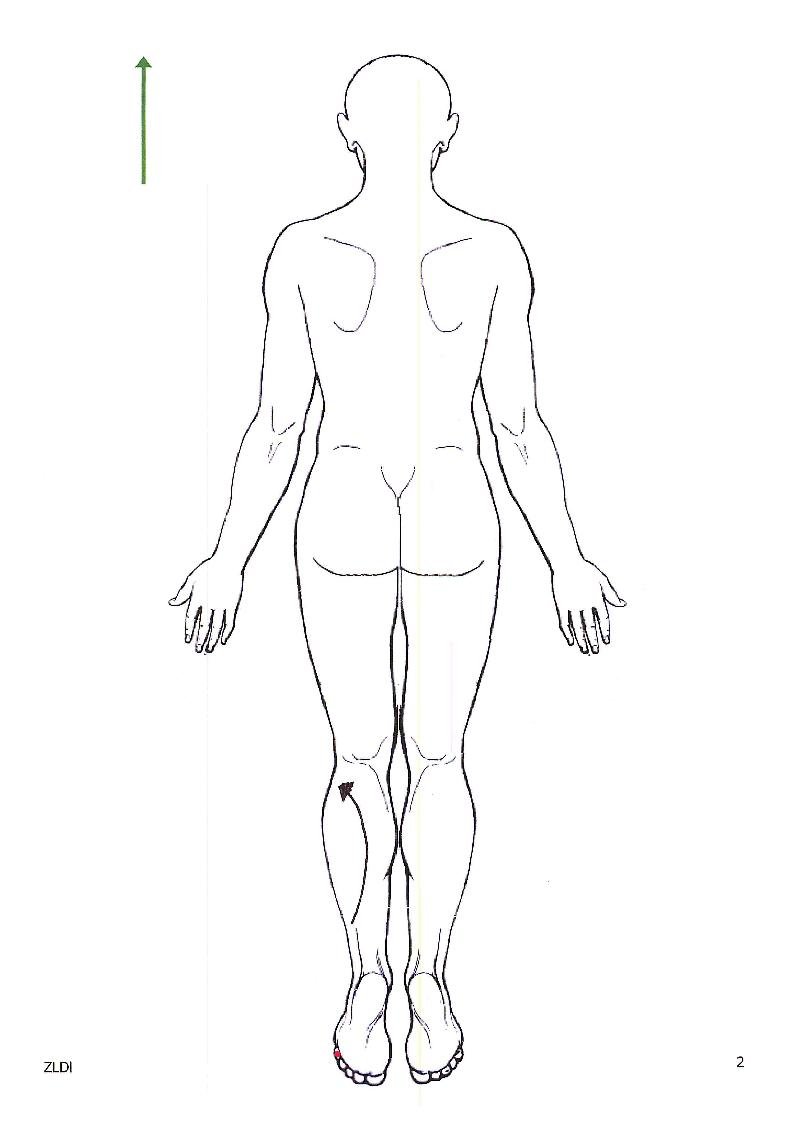

Supplement: S1 Raw Data — (ZIP) [file pone.0124808.s006.zip › Drawings - Imagined stimulation/toe_back/Subject_44_toe_back.jpg]

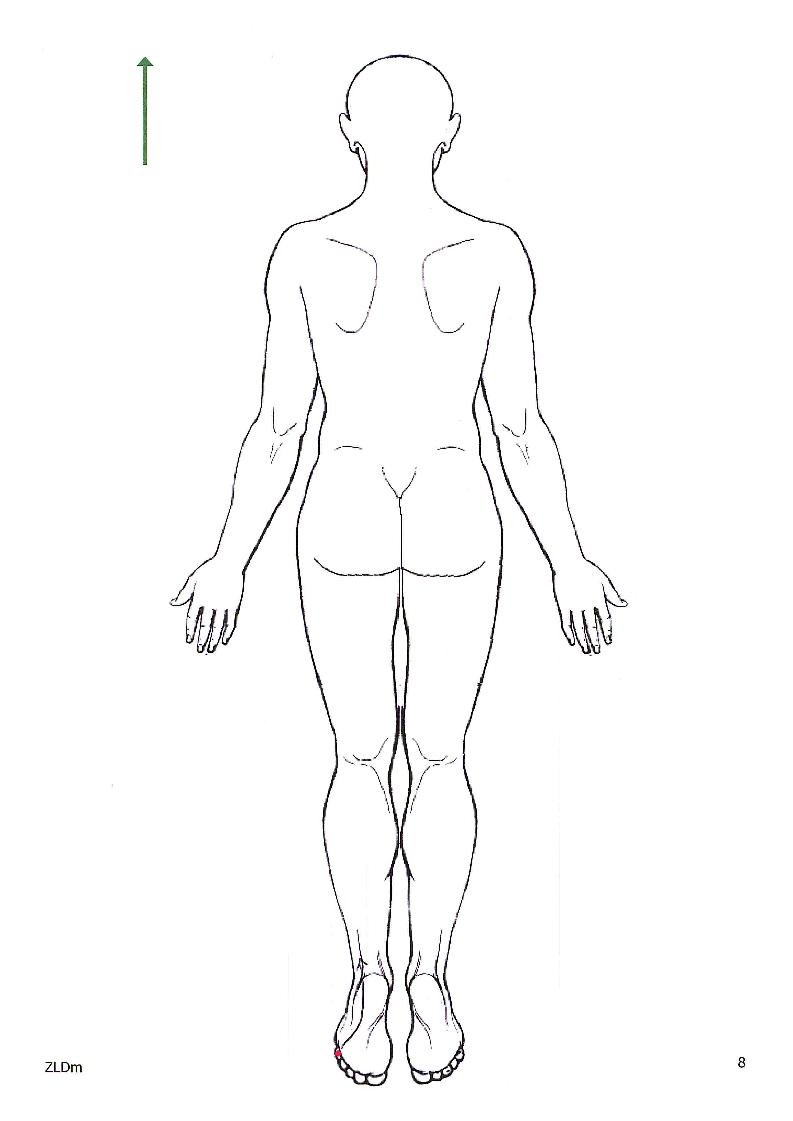

Supplement: S1 Raw Data — (ZIP) [file pone.0124808.s006.zip › Drawings - Imagined stimulation/toe_back/Subject_46_toe_back.jpg]

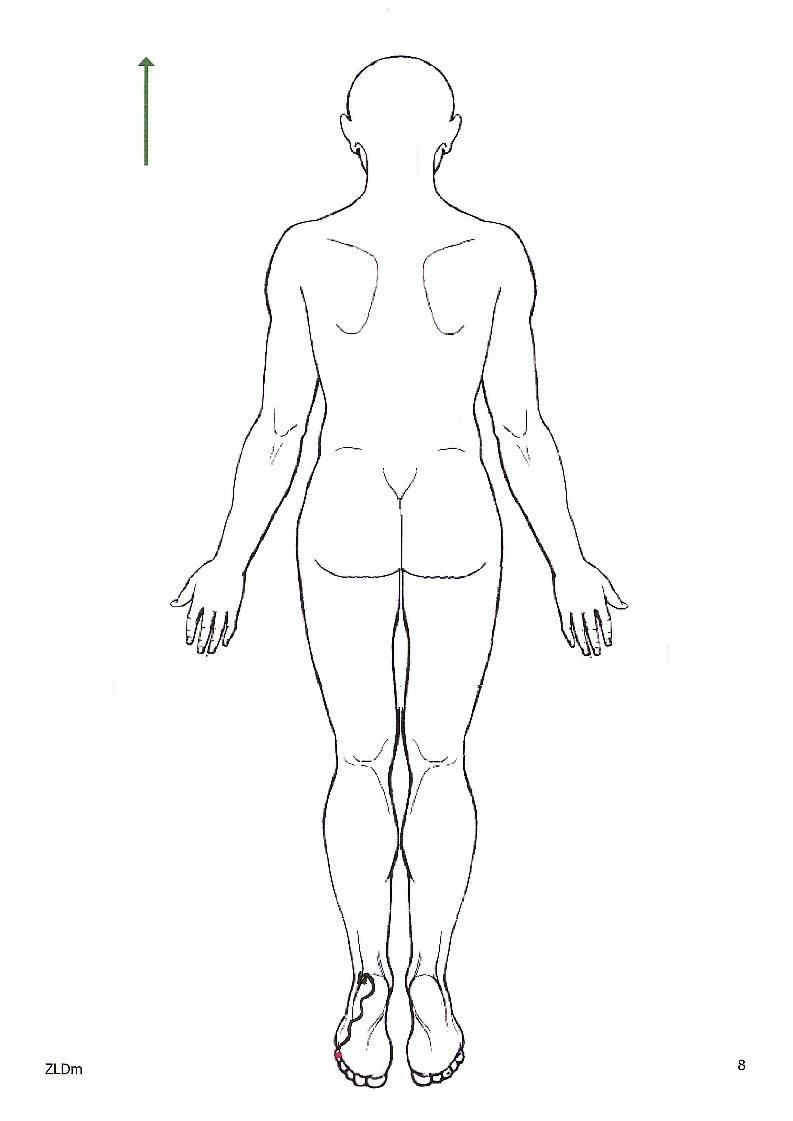

Supplement: S1 Raw Data — (ZIP) [file pone.0124808.s006.zip › Drawings - Imagined stimulation/toe_back/Subject_23_toe_back.jpg]

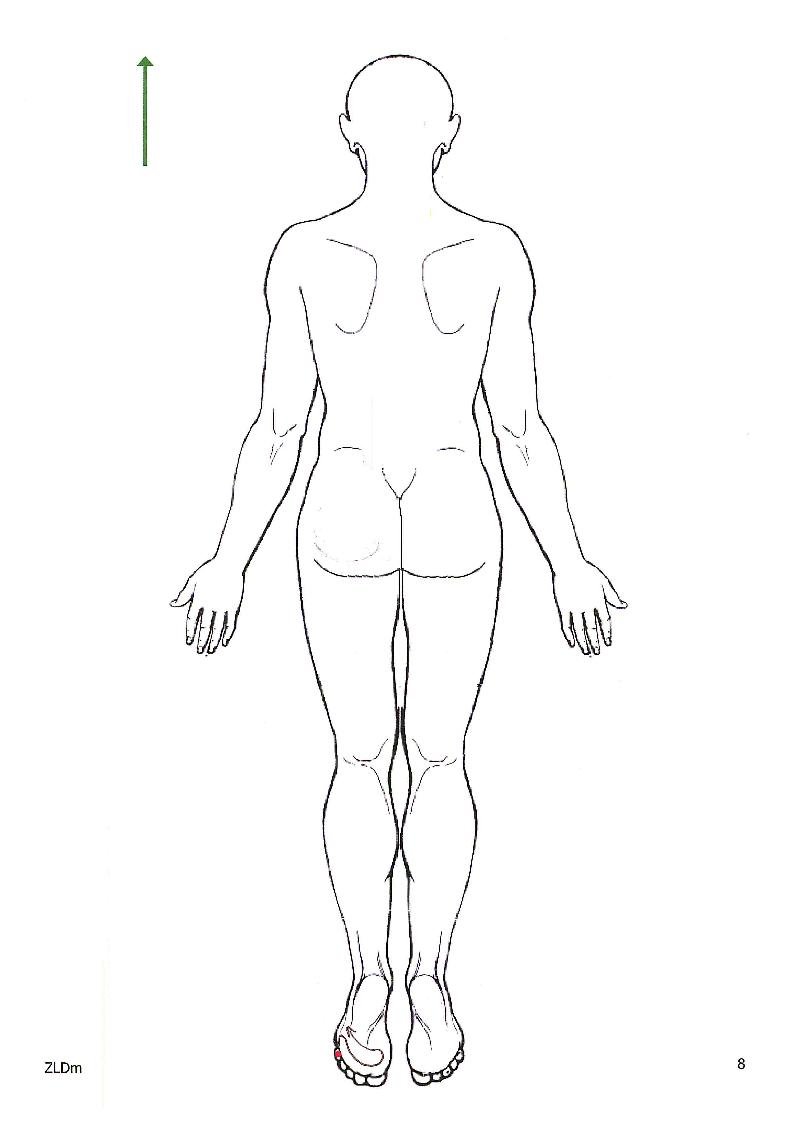

Supplement: S1 Raw Data — (ZIP) [file pone.0124808.s006.zip › Drawings - Imagined stimulation/toe_back/Subject_18_toe_back.jpg]

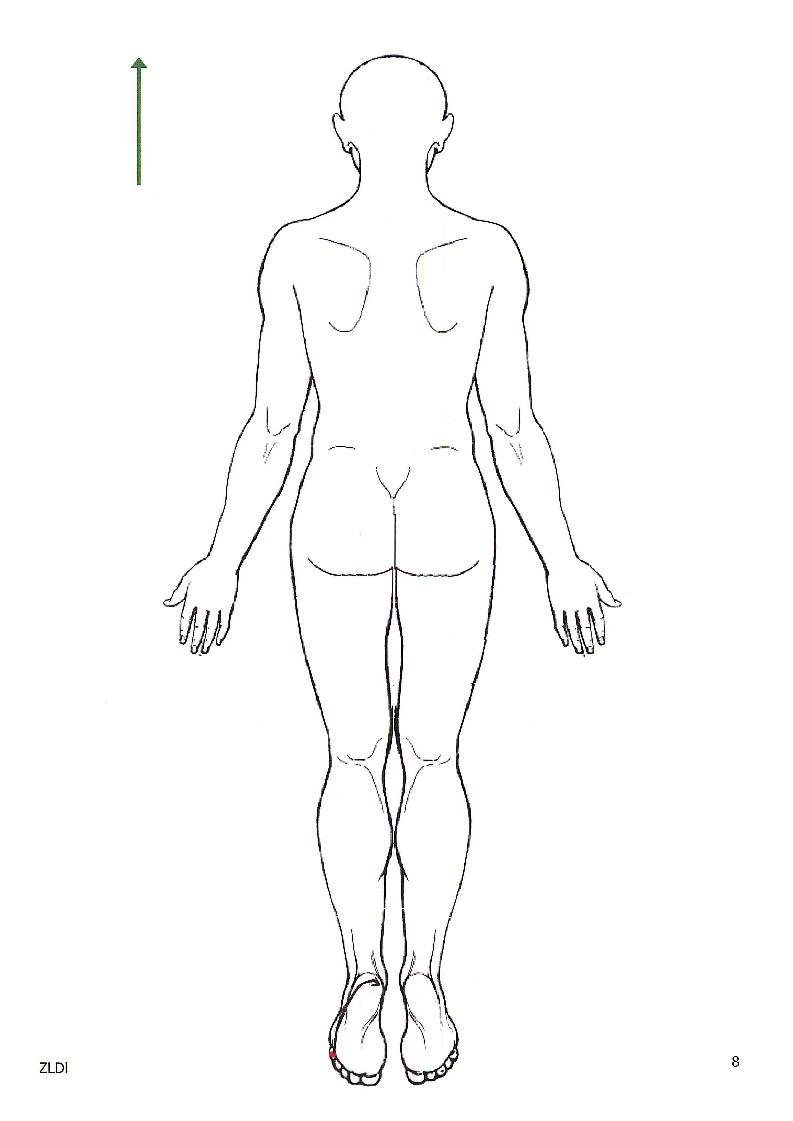

Supplement: S1 Raw Data — (ZIP) [file pone.0124808.s006.zip › Drawings - Imagined stimulation/toe_back/Subject_48_toe_back.jpg]

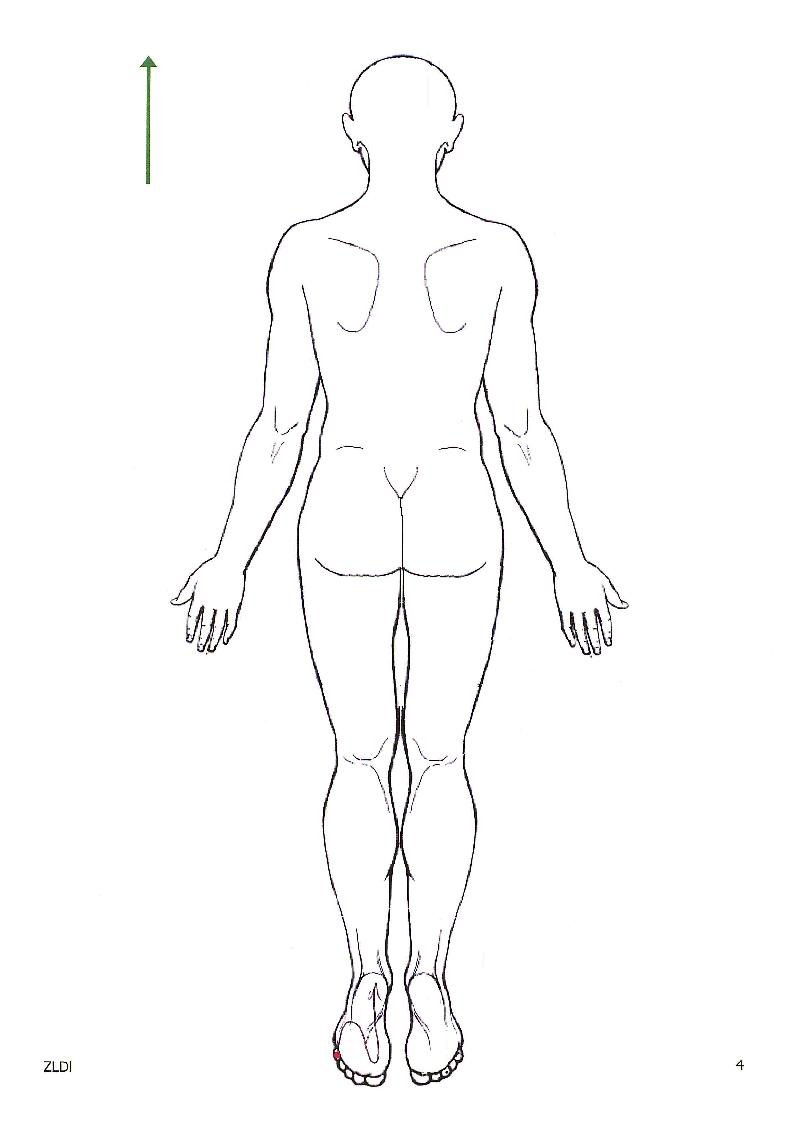

Supplement: S1 Raw Data — (ZIP) [file pone.0124808.s006.zip › Drawings - Imagined stimulation/toe_back/Subject_24_toe_back.jpg]

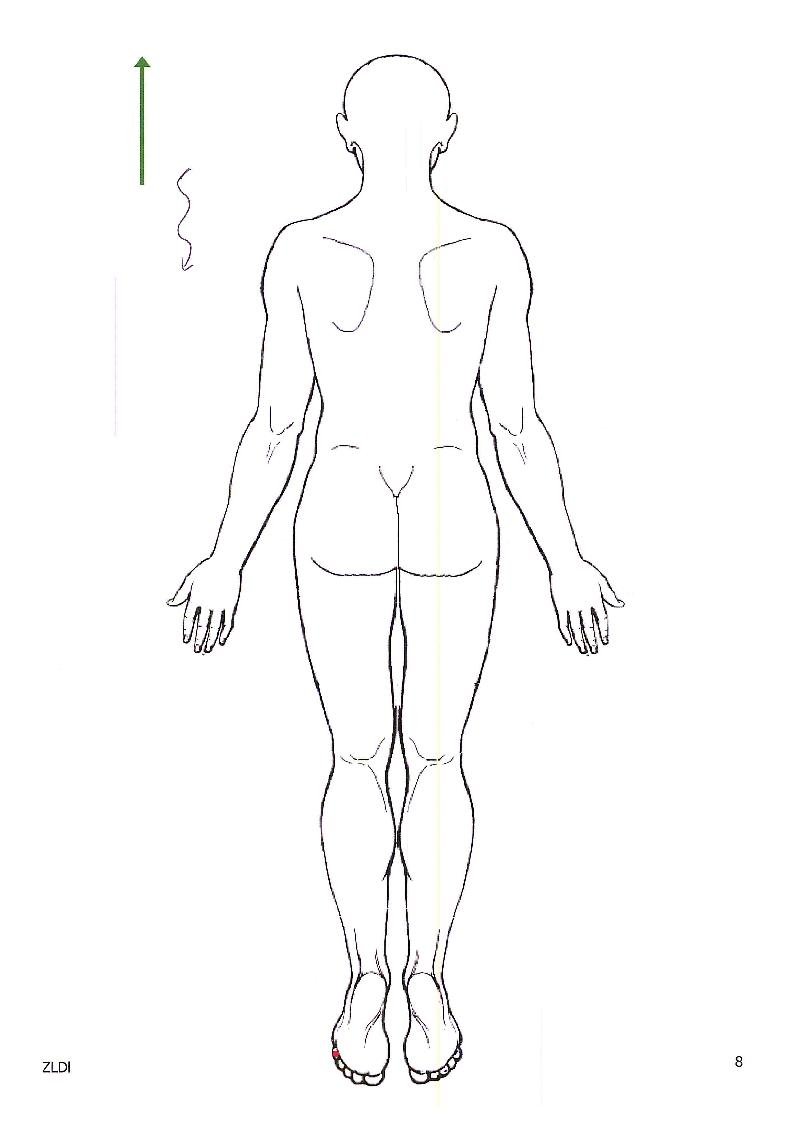

Supplement: S1 Raw Data — (ZIP) [file pone.0124808.s006.zip › Drawings - Imagined stimulation/toe_back/Subject_52_toe_back.jpg]

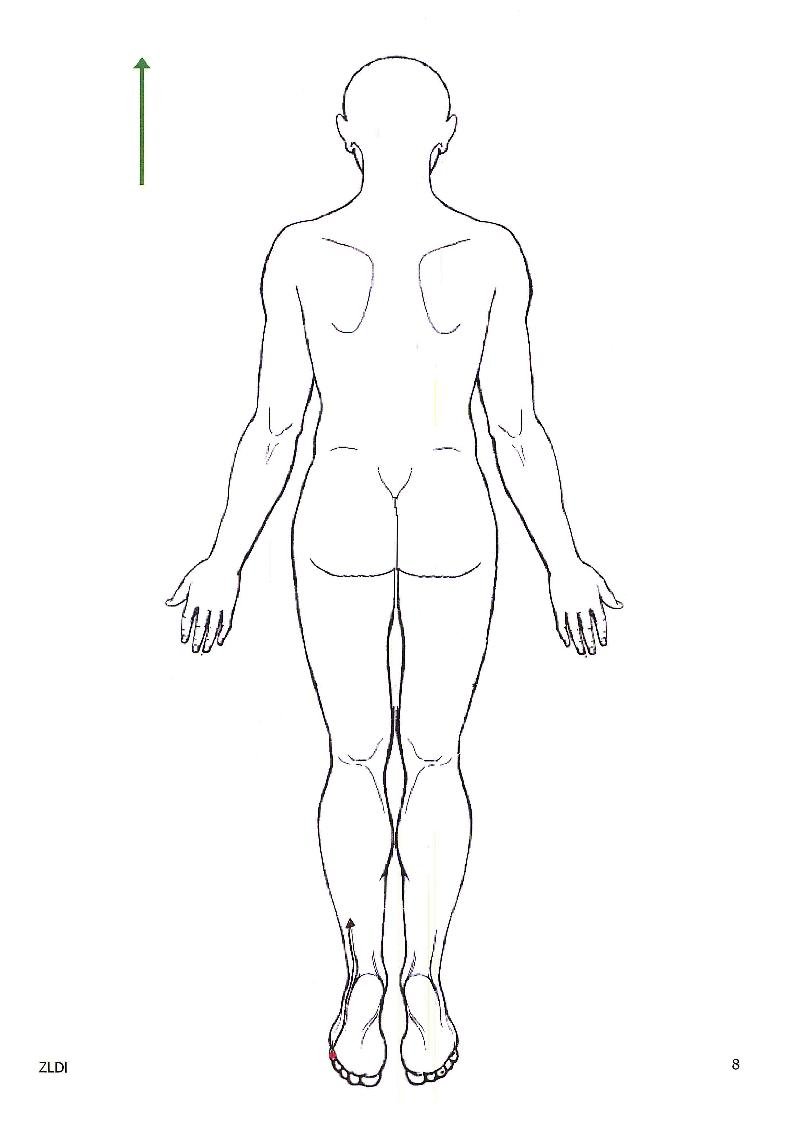

Supplement: S1 Raw Data — (ZIP) [file pone.0124808.s006.zip › Drawings - Imagined stimulation/toe_back/Subject_47_toe_back.jpg]

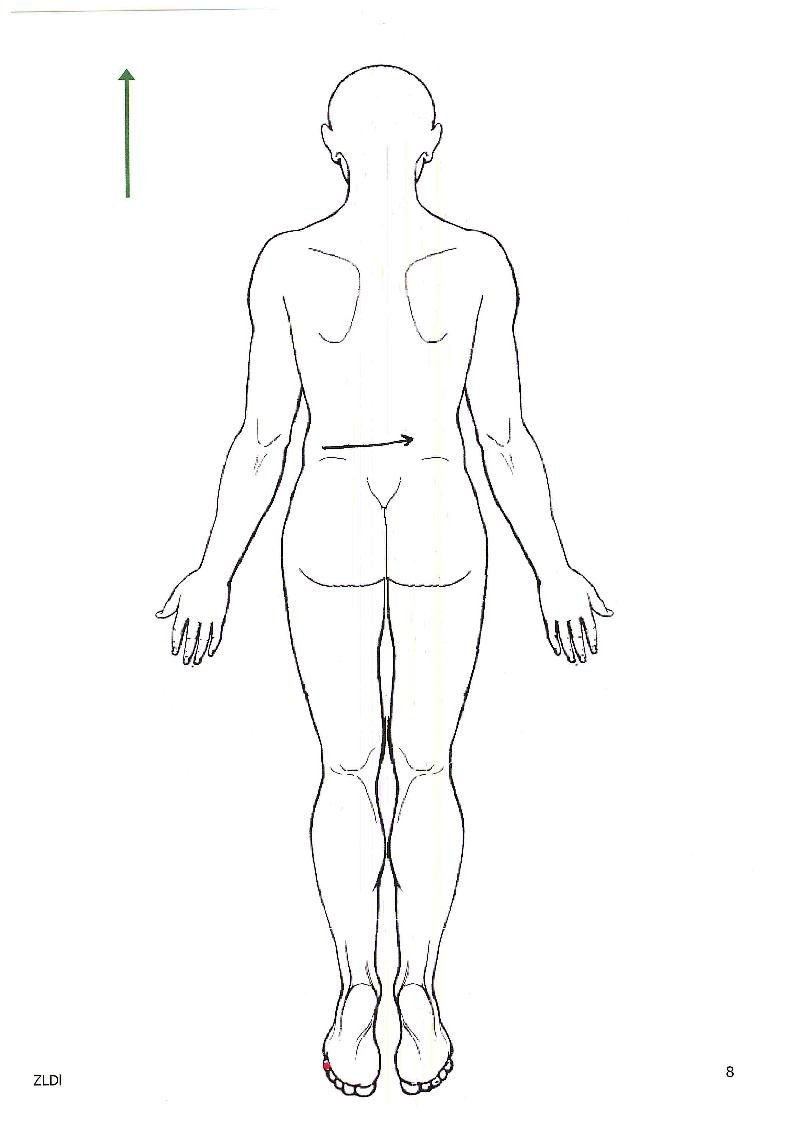

Supplement: S1 Raw Data — (ZIP) [file pone.0124808.s006.zip › Drawings - Imagined stimulation/toe_back/Subject_6_toe_back.jpg]

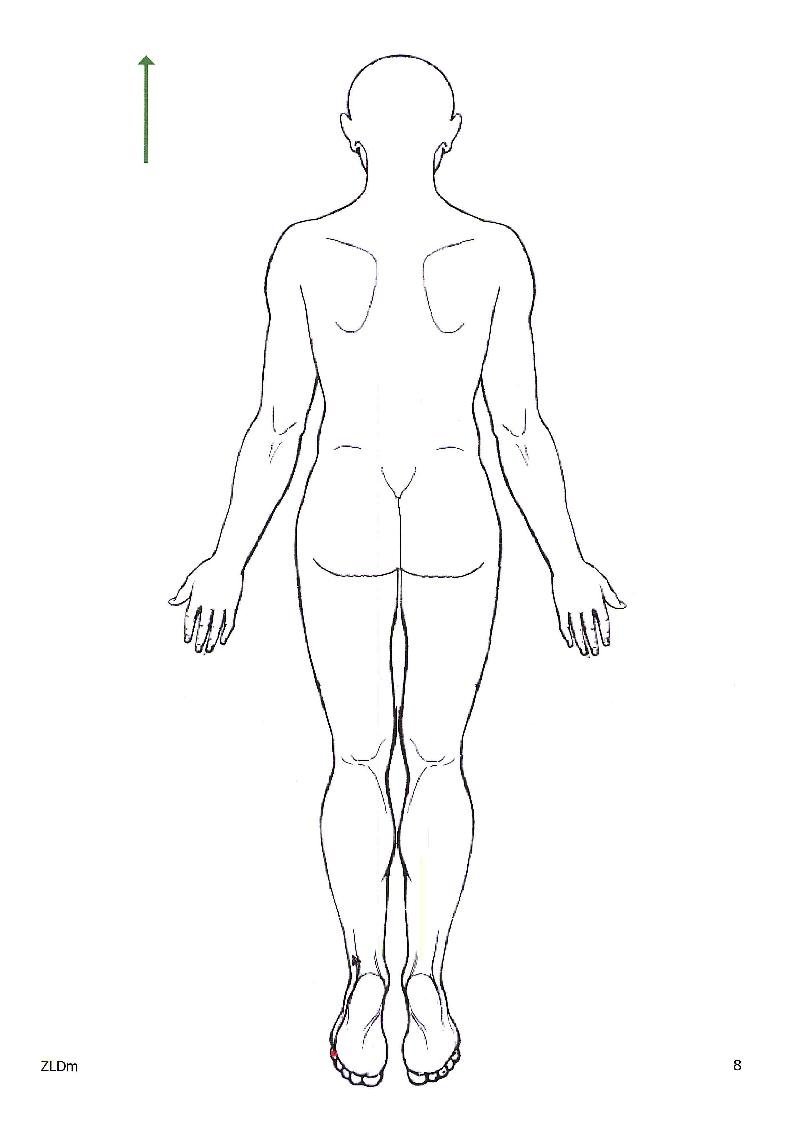

Supplement: S1 Raw Data — (ZIP) [file pone.0124808.s006.zip › Drawings - Imagined stimulation/toe_back/Subject_49_toe_back.jpg]

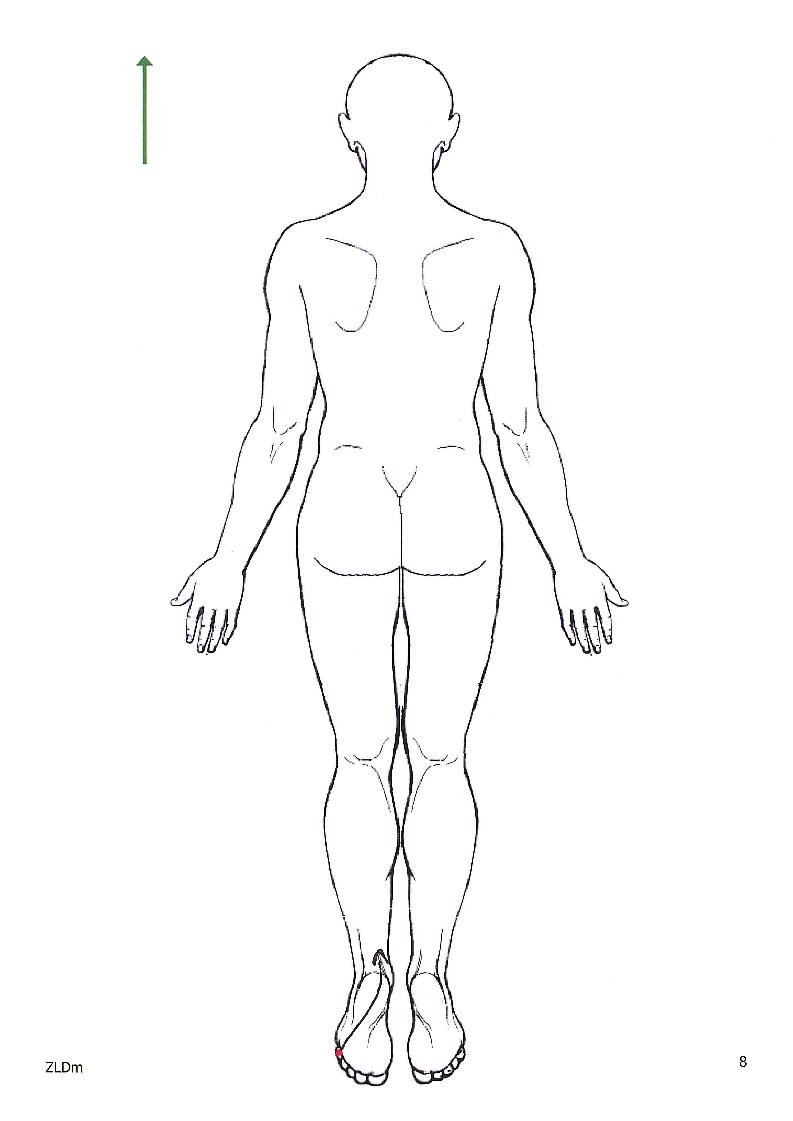

Supplement: S1 Raw Data — (ZIP) [file pone.0124808.s006.zip › Drawings - Imagined stimulation/toe_back/Subject_2_toe_back.jpg]

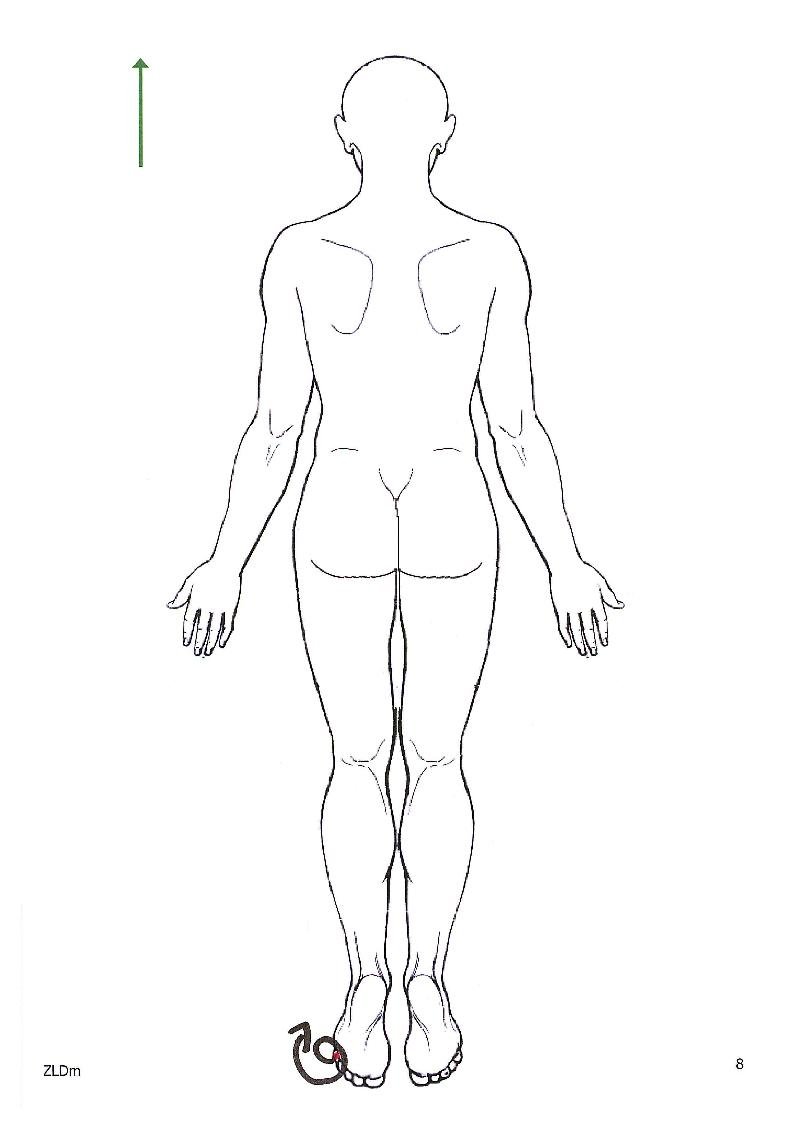

Supplement: S1 Raw Data — (ZIP) [file pone.0124808.s006.zip › Drawings - Imagined stimulation/toe_back/Subject_55_toe_back.jpg]

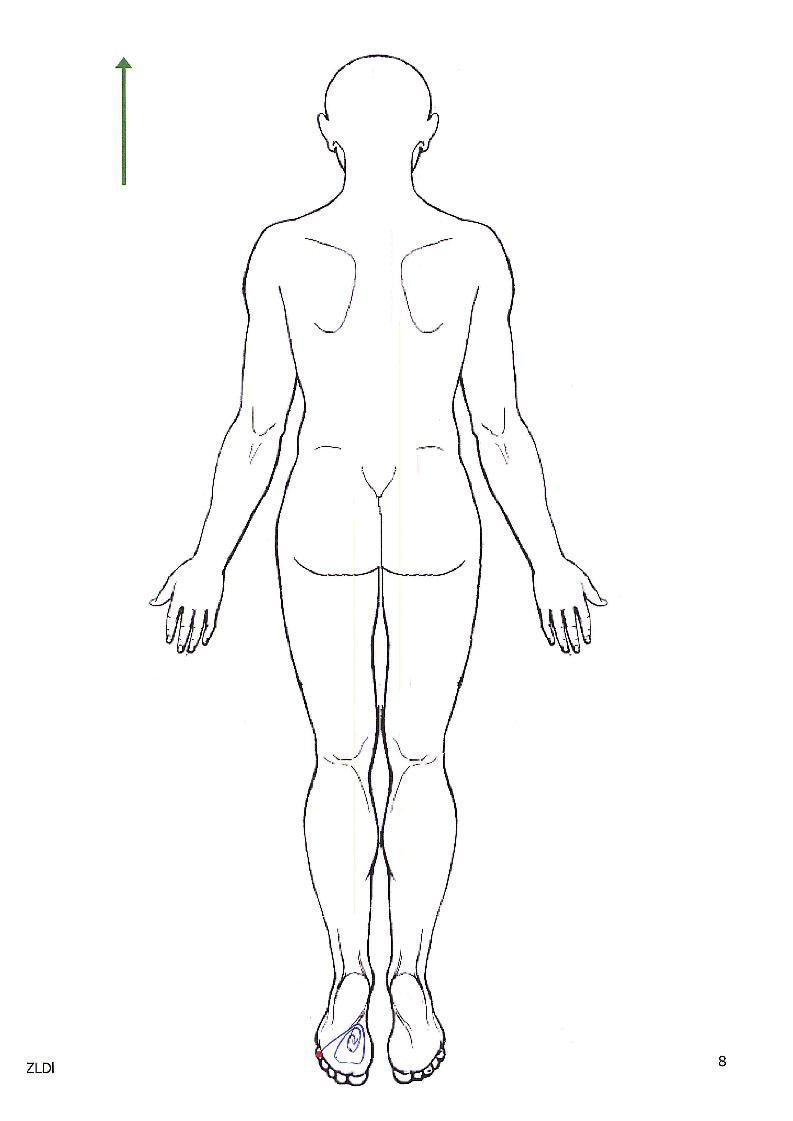

Supplement: S1 Raw Data — (ZIP) [file pone.0124808.s006.zip › Drawings - Imagined stimulation/toe_back/Subject_3_toe_back.jpg]

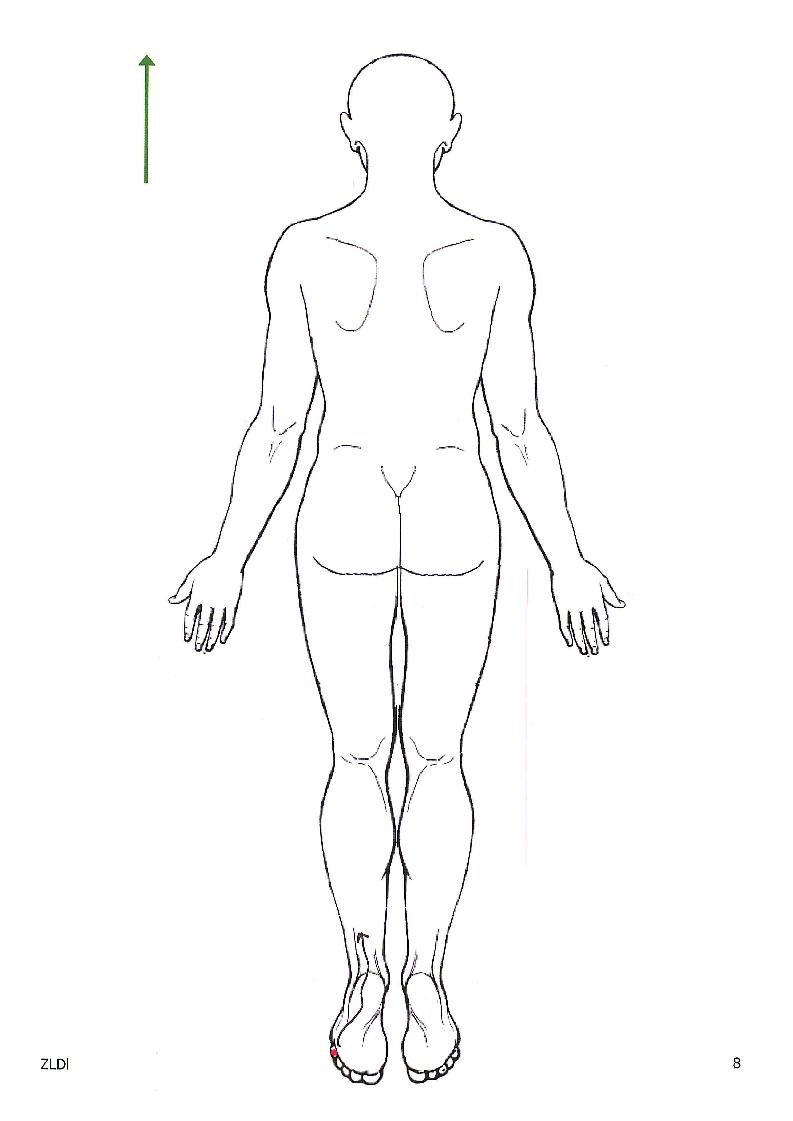

Supplement: S1 Raw Data — (ZIP) [file pone.0124808.s006.zip › Drawings - Imagined stimulation/toe_back/Subject_29_toe_back.jpg]

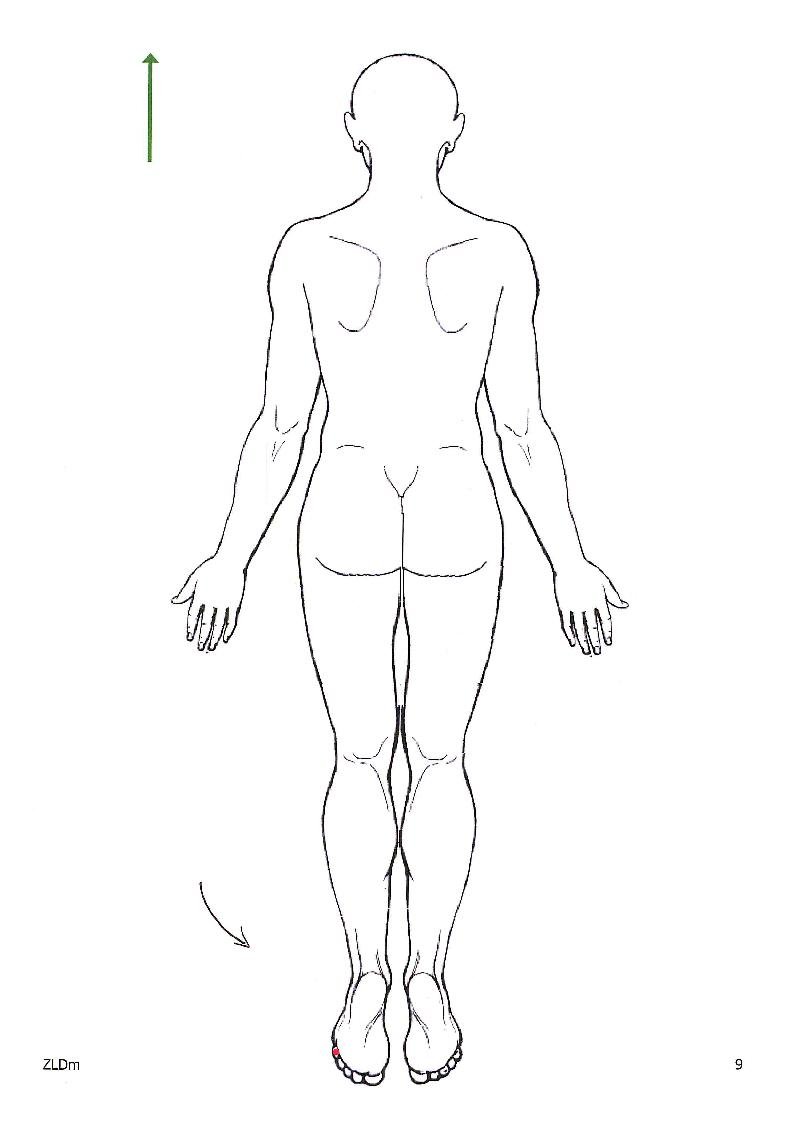

Supplement: S1 Raw Data — (ZIP) [file pone.0124808.s006.zip › Drawings - Imagined stimulation/toe_back/Subject_34_toe_back.jpg]

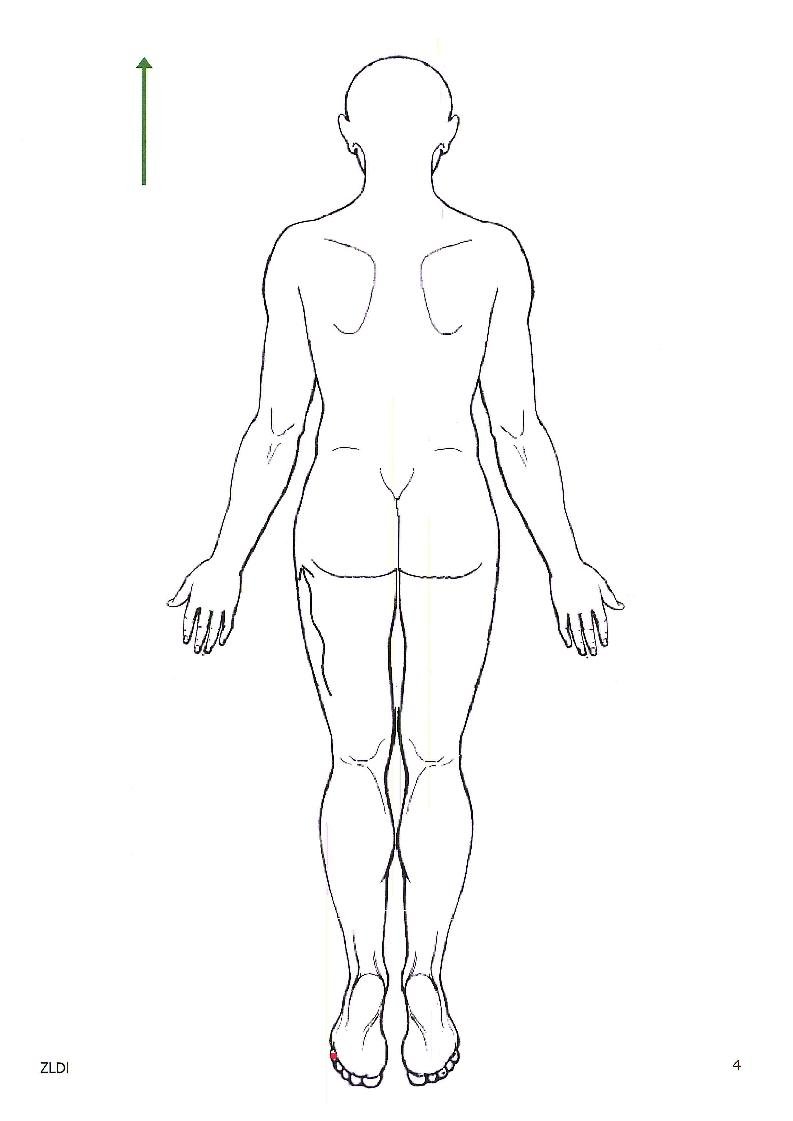

Supplement: S1 Raw Data — (ZIP) [file pone.0124808.s006.zip › Drawings - Imagined stimulation/toe_back/Subject_11_toe_back.jpg]

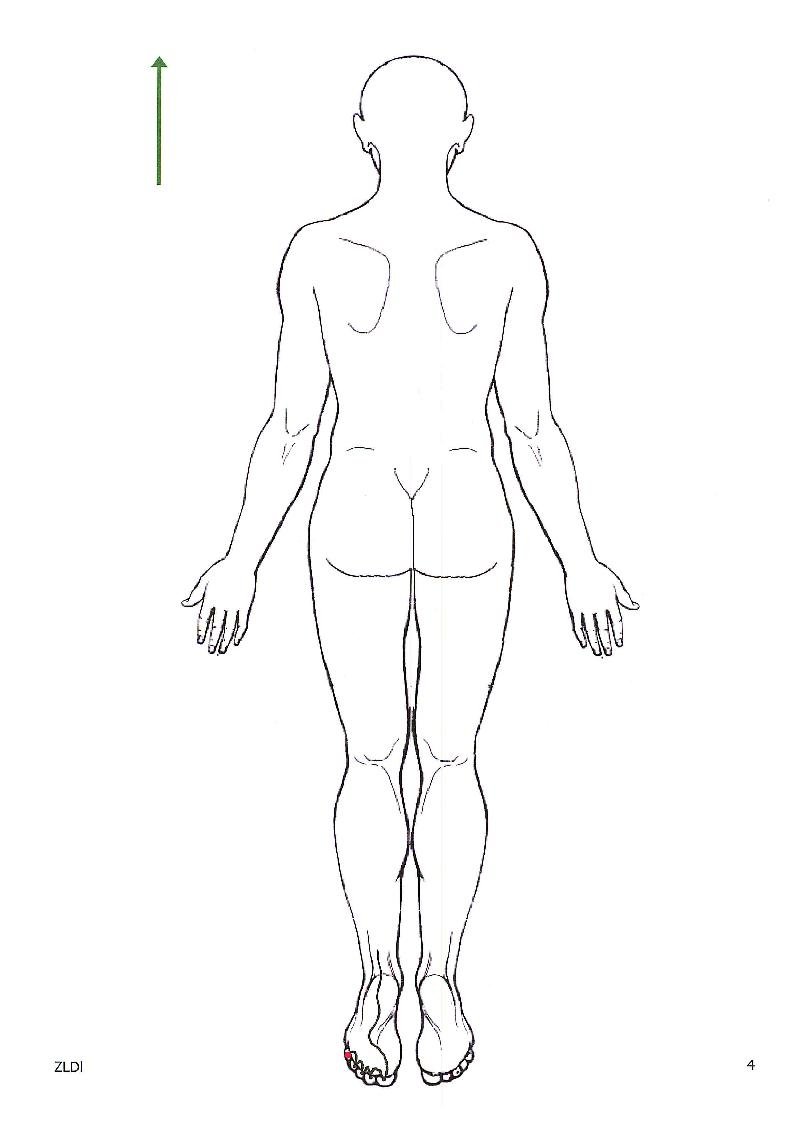

Supplement: S1 Raw Data — (ZIP) [file pone.0124808.s006.zip › Drawings - Imagined stimulation/toe_back/Subject_50_toe_back.jpg]

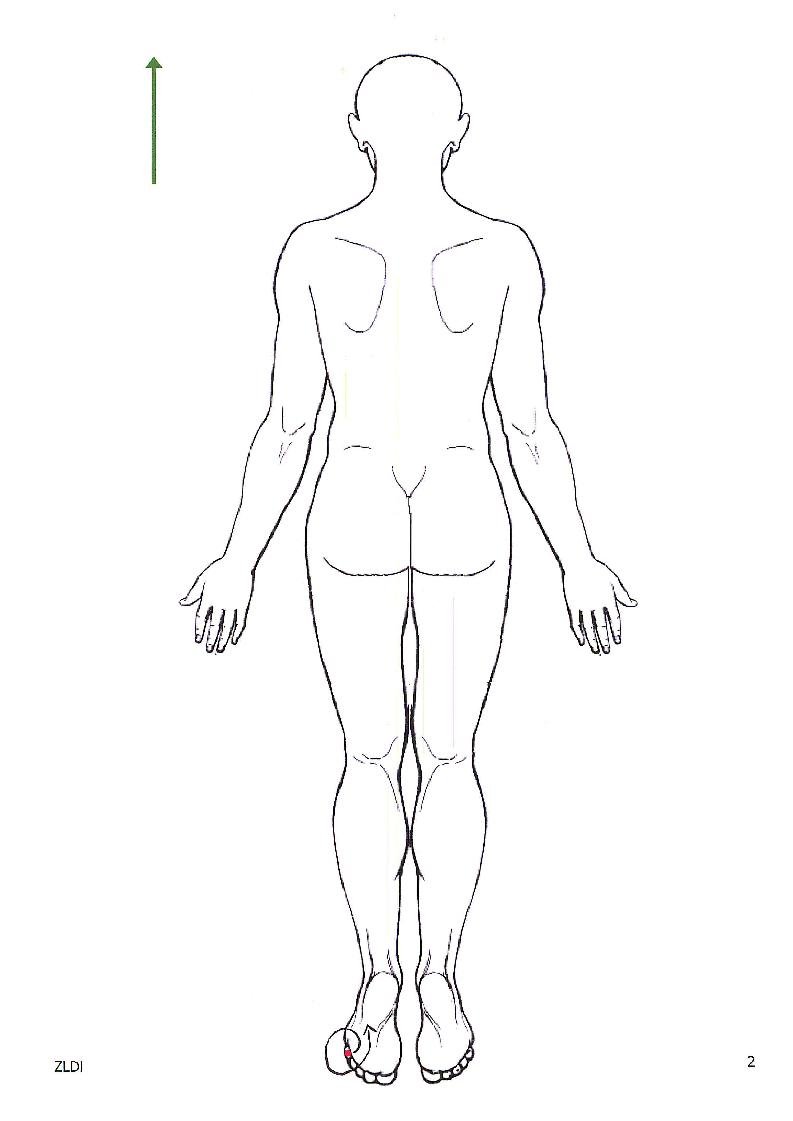

Supplement: S1 Raw Data — (ZIP) [file pone.0124808.s006.zip › Drawings - Imagined stimulation/toe_back/Subject_32_toe_back.jpg]

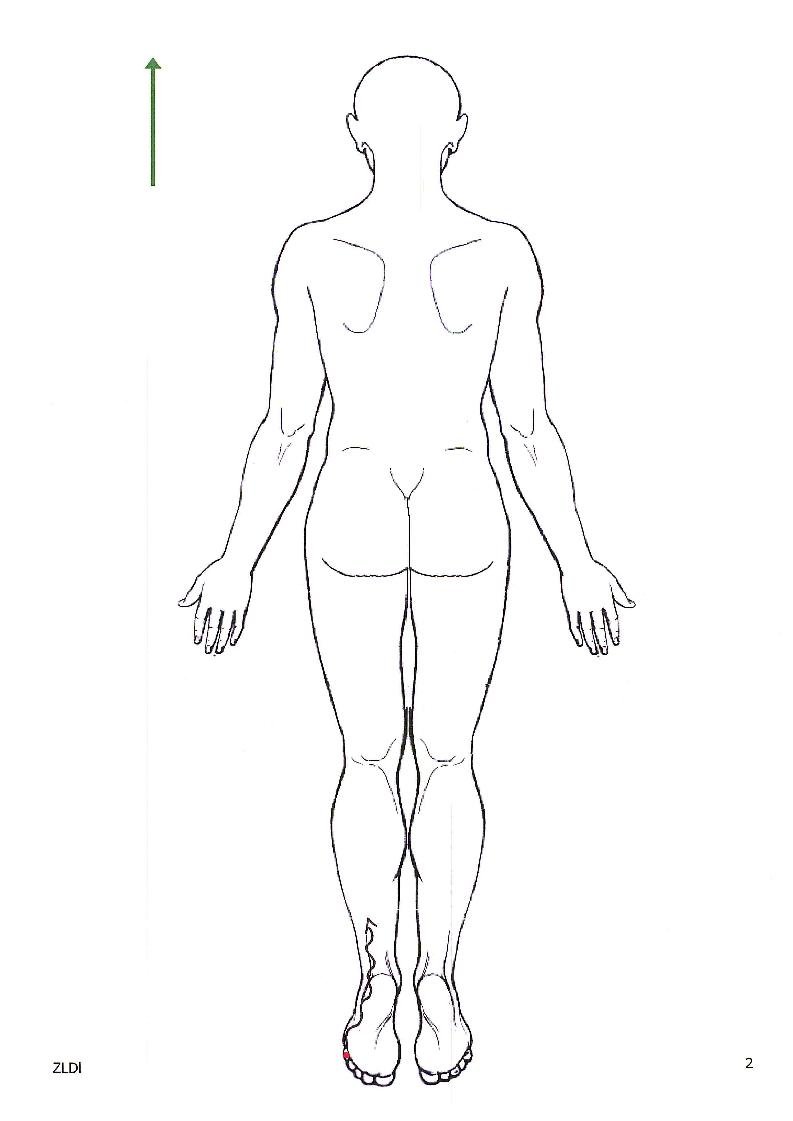

Supplement: S1 Raw Data — (ZIP) [file pone.0124808.s006.zip › Drawings - Imagined stimulation/toe_back/Subject_5_toe_back.jpg]

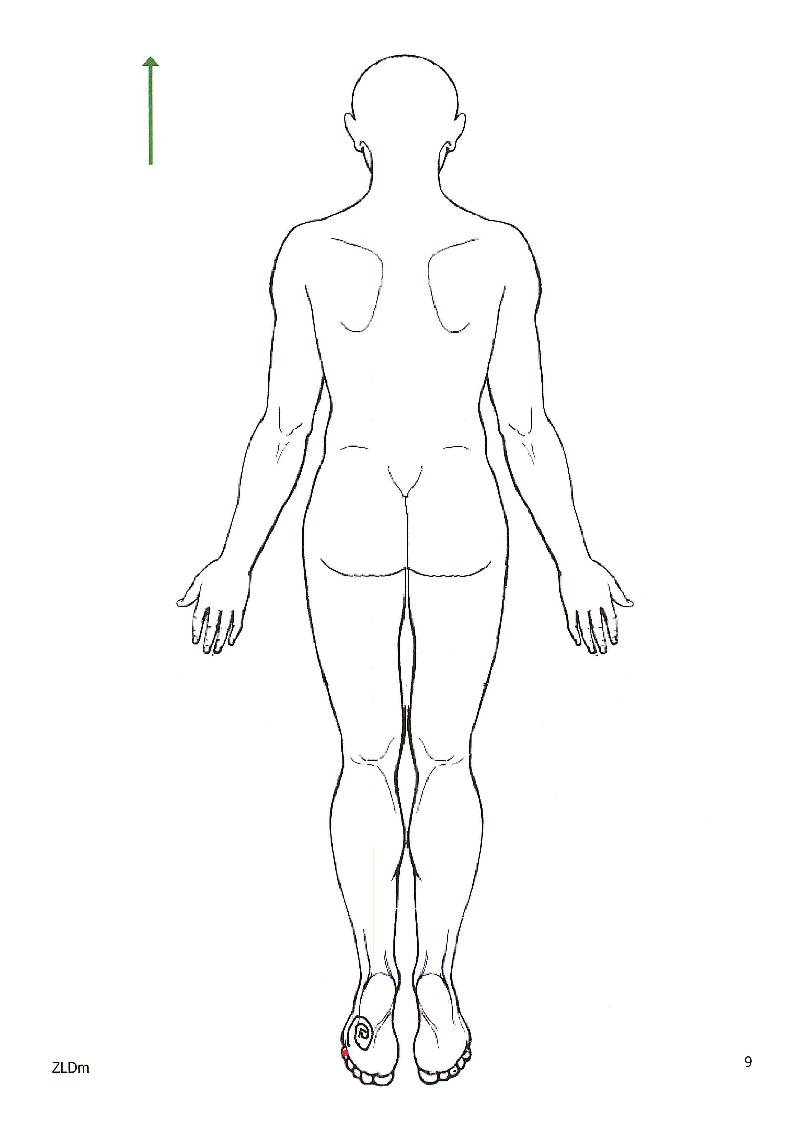

Supplement: S1 Raw Data — (ZIP) [file pone.0124808.s006.zip › Drawings - Imagined stimulation/toe_back/Subject_26_toe_back.jpg]

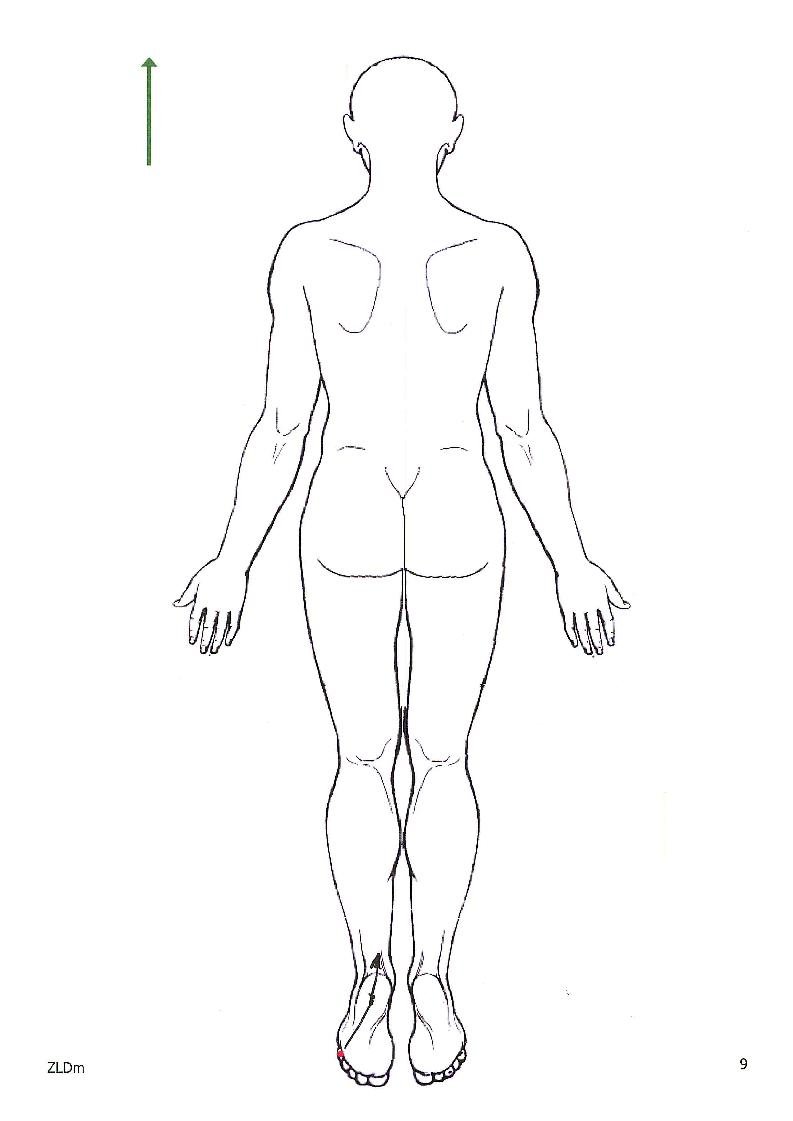

Supplement: S1 Raw Data — (ZIP) [file pone.0124808.s006.zip › Drawings - Imagined stimulation/toe_back/Subject_35_toe_back.jpg]

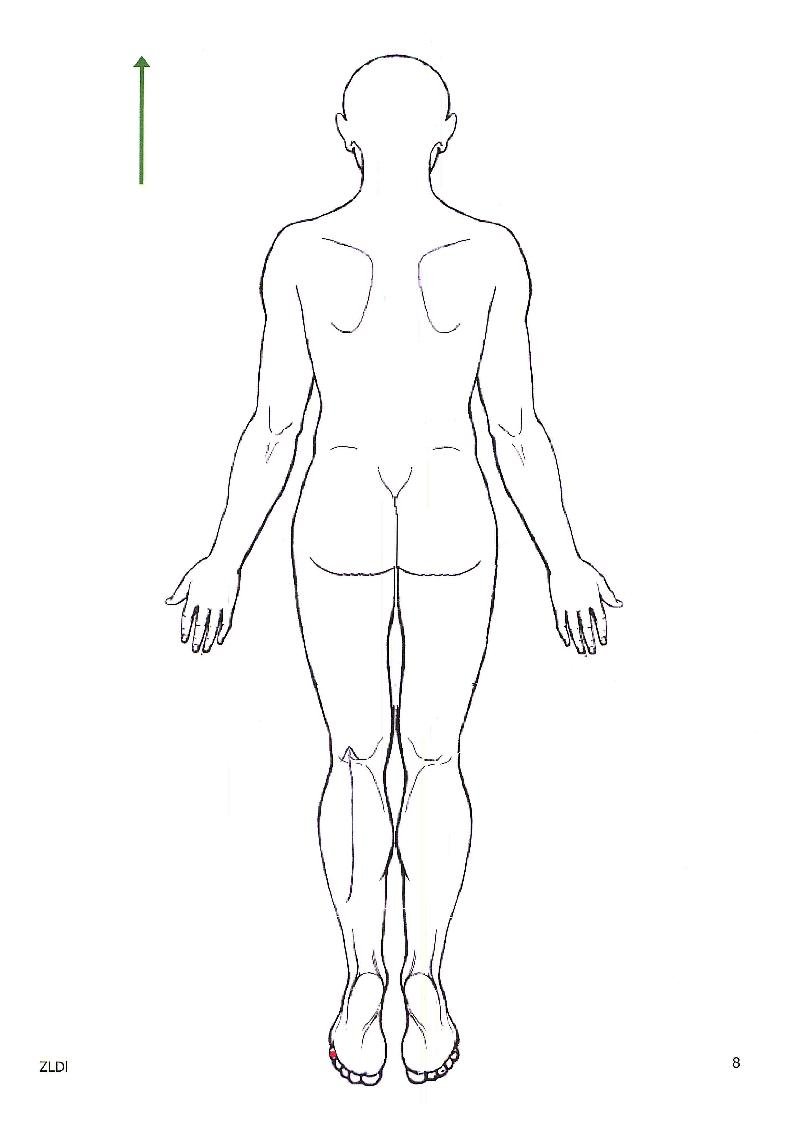

Supplement: S1 Raw Data — (ZIP) [file pone.0124808.s006.zip › Drawings - Imagined stimulation/toe_back/Subject_12_toe_back.jpg]

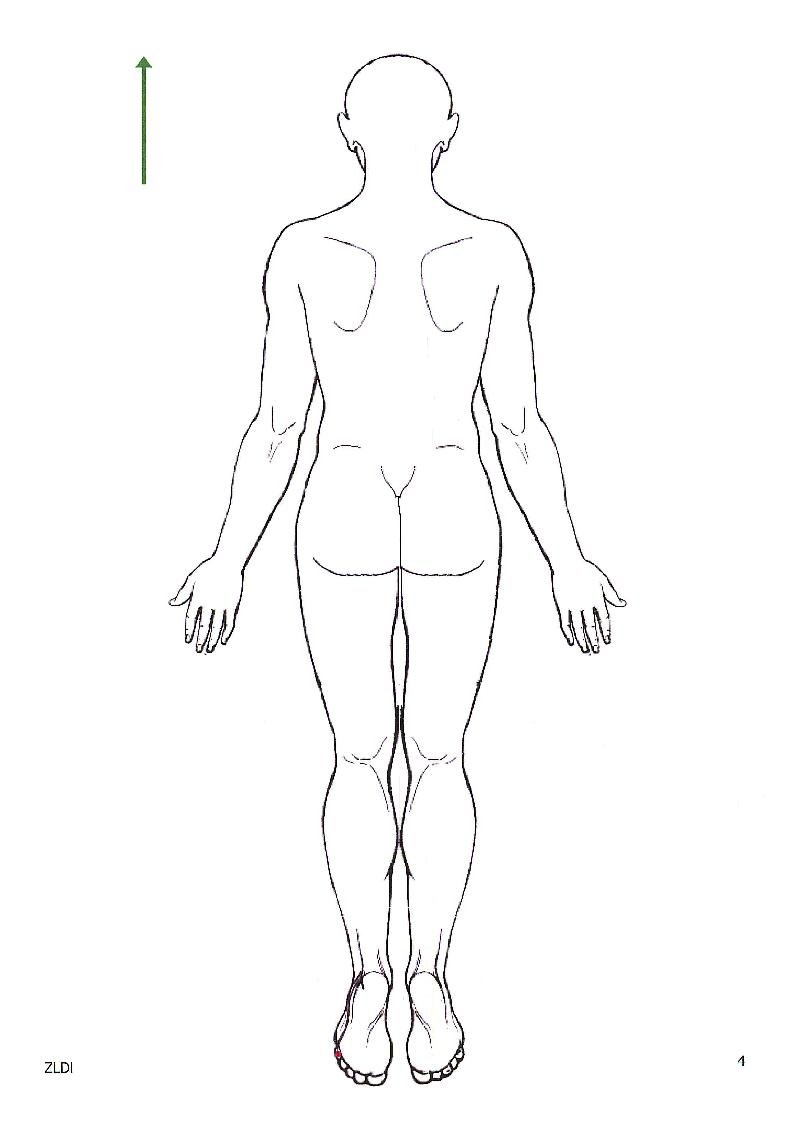

Supplement: S1 Raw Data — (ZIP) [file pone.0124808.s006.zip › Drawings - Imagined stimulation/toe_back/Subject_1_toe_back.jpg]

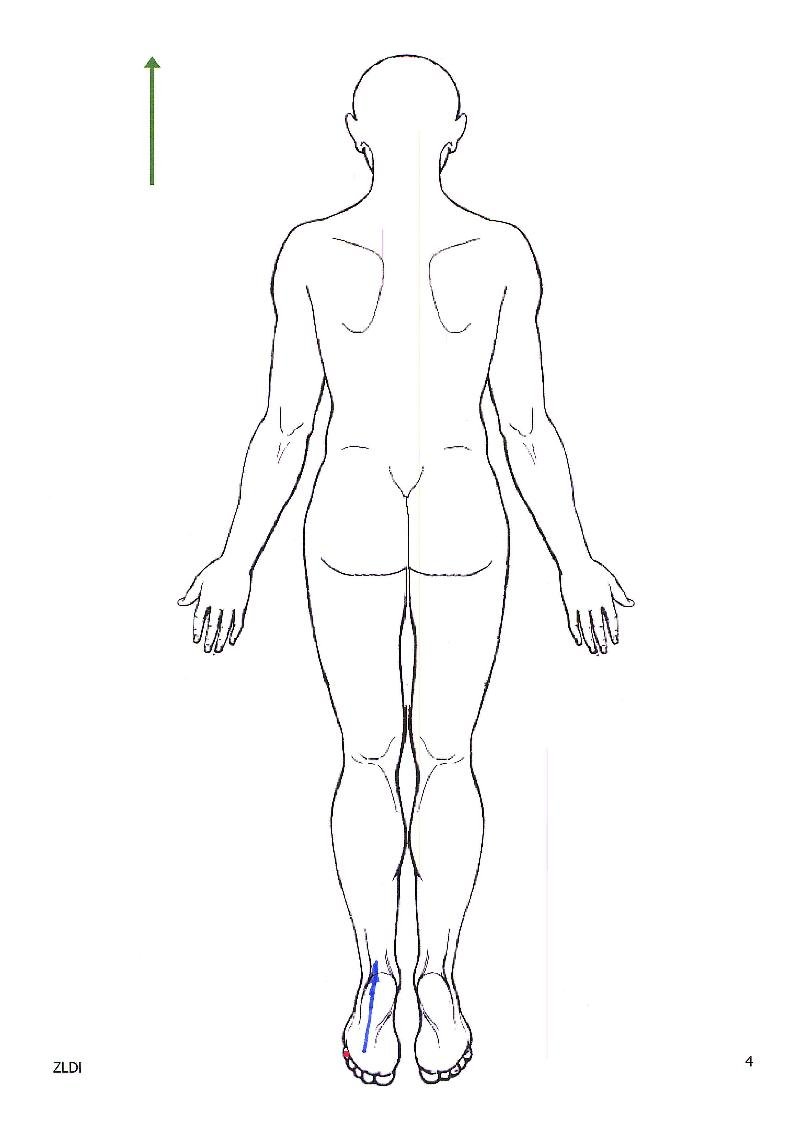

Supplement: S1 Raw Data — (ZIP) [file pone.0124808.s006.zip › Drawings - Imagined stimulation/toe_back/Subject_14_toe_back.jpg]

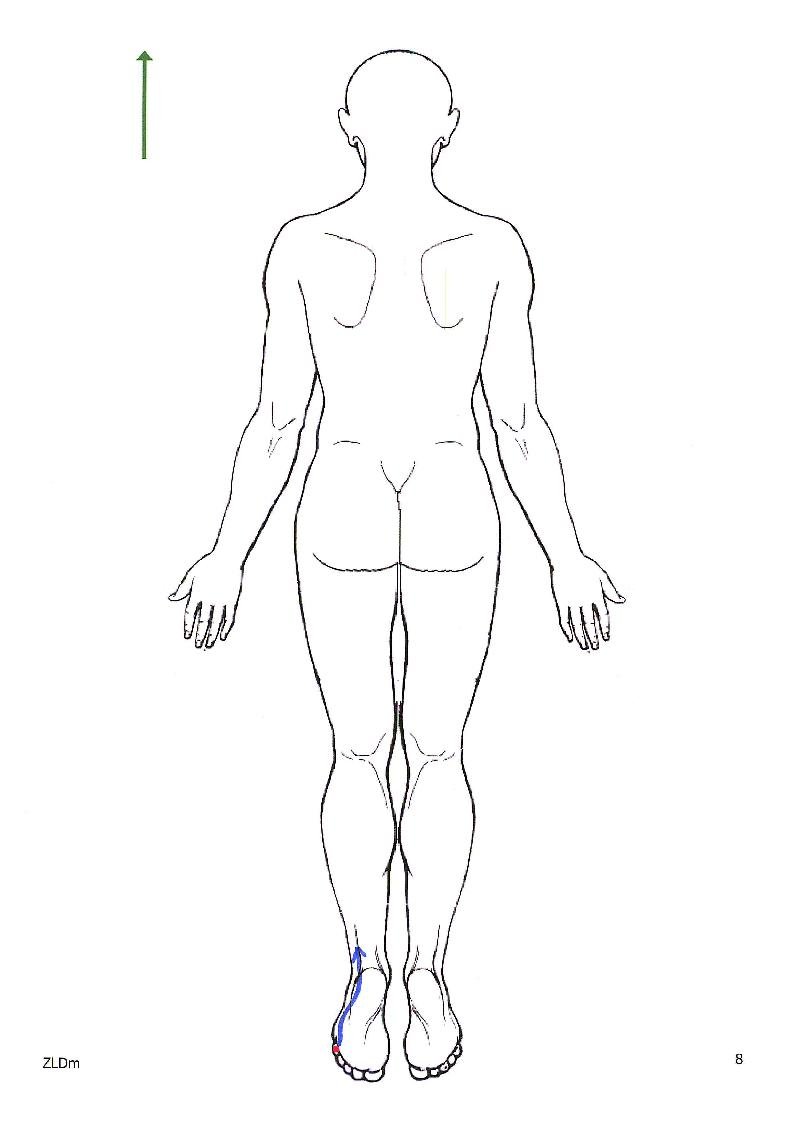

Supplement: S1 Raw Data — (ZIP) [file pone.0124808.s006.zip › Drawings - Imagined stimulation/toe_back/Subject_42_toe_back.jpg]

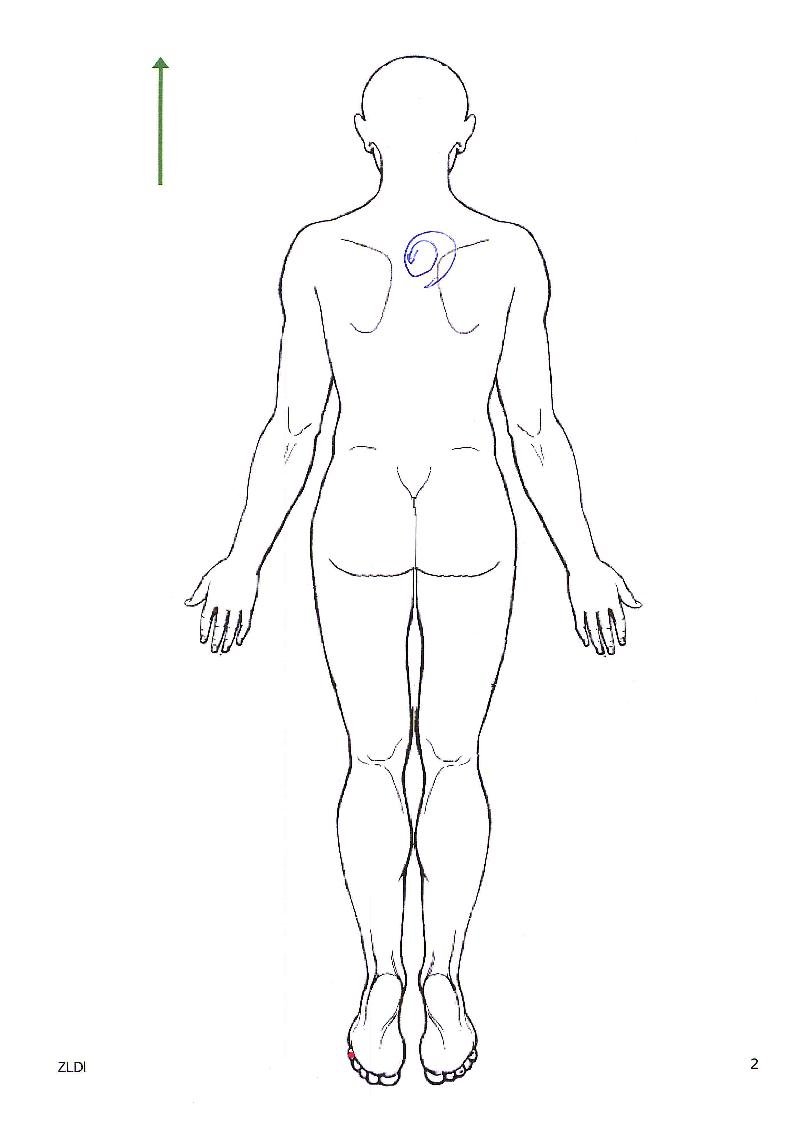

Supplement: S1 Raw Data — (ZIP) [file pone.0124808.s006.zip › Drawings - Imagined stimulation/toe_back/Subject_31_toe_back.jpg]

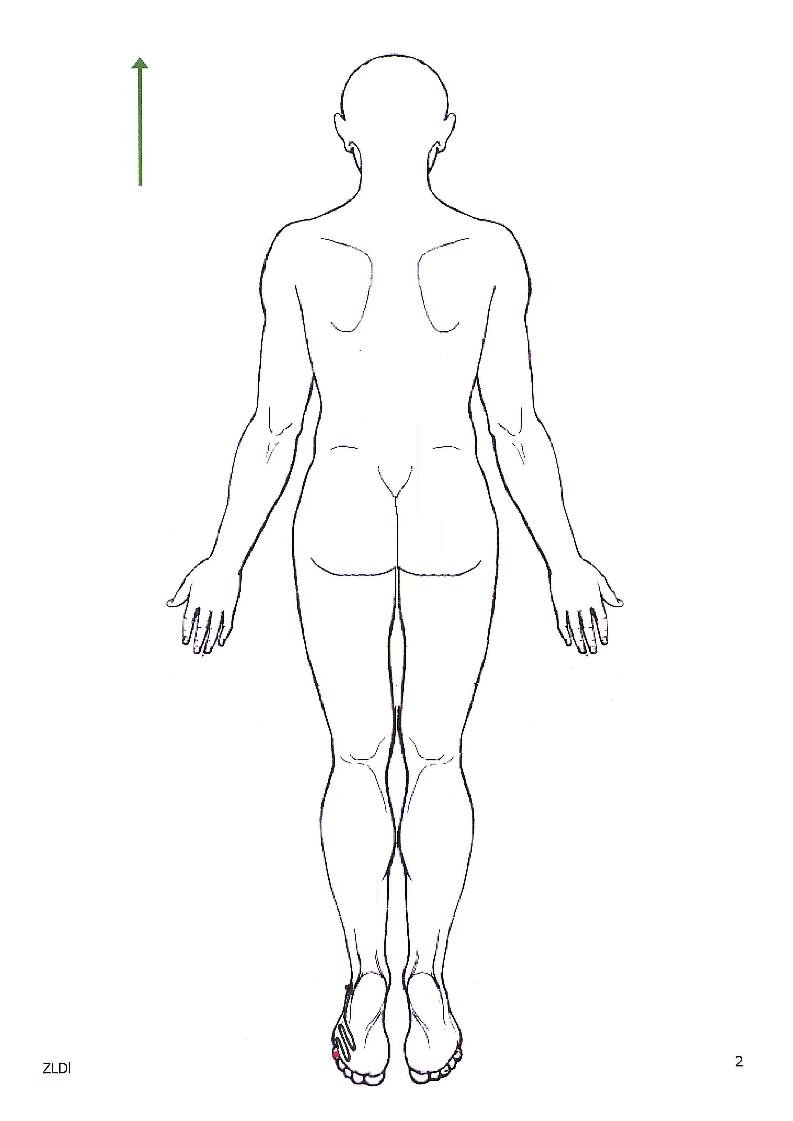

Supplement: S1 Raw Data — (ZIP) [file pone.0124808.s006.zip › Drawings - Imagined stimulation/toe_back/Subject_28_toe_back.jpg]

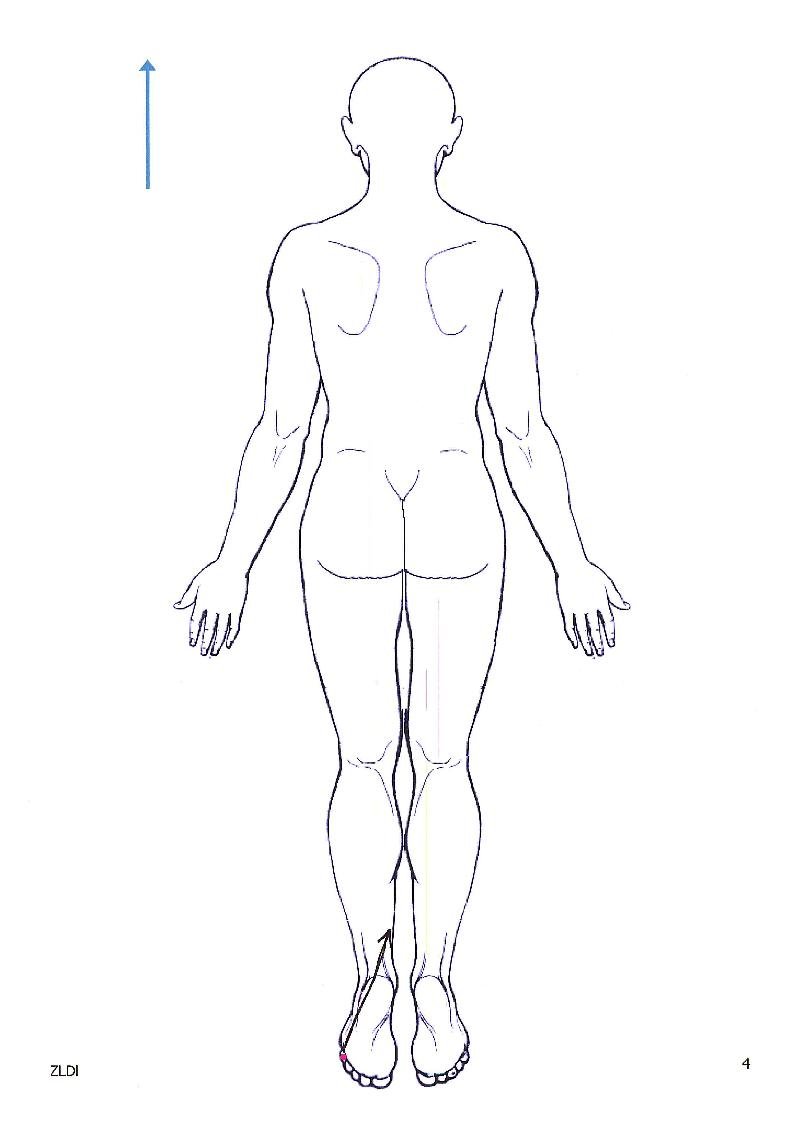

Supplement: S1 Raw Data — (ZIP) [file pone.0124808.s006.zip › Drawings - Imagined stimulation/toe_back/Subject_37_toe_back.jpg]

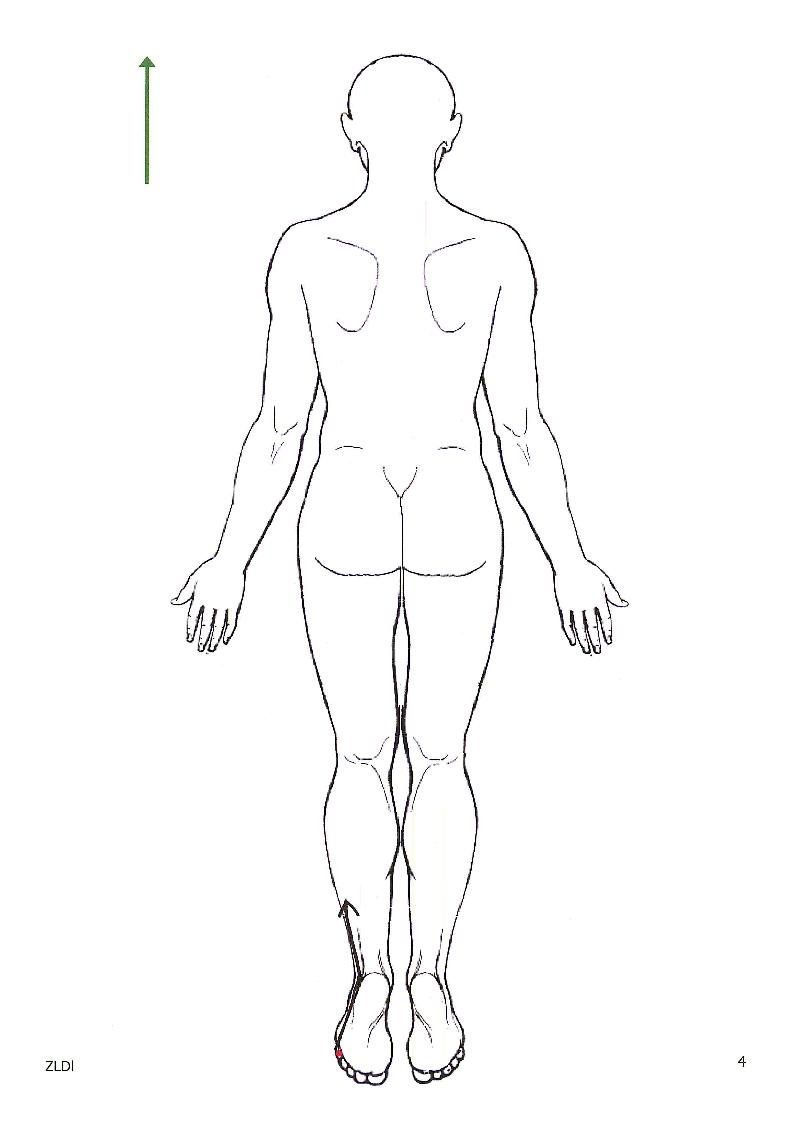

Supplement: S1 Raw Data — (ZIP) [file pone.0124808.s006.zip › Drawings - Imagined stimulation/toe_back/Subject_8_toe_back.jpg]

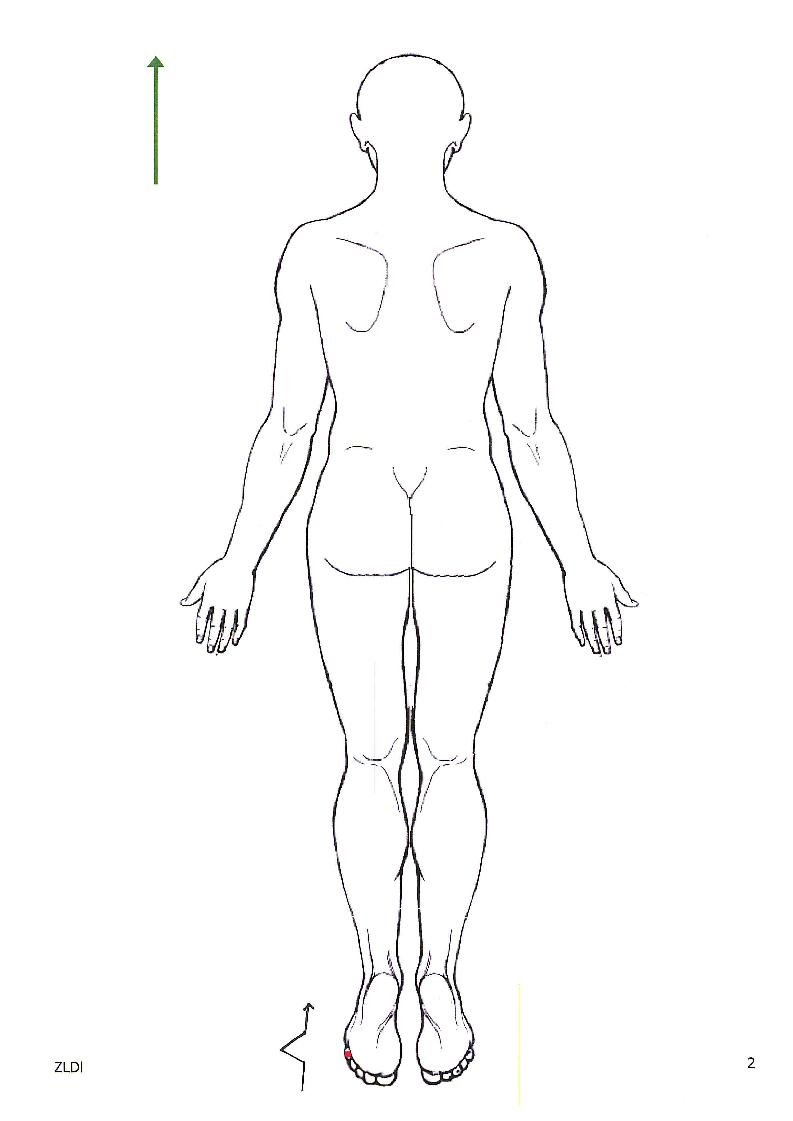

Supplement: S1 Raw Data — (ZIP) [file pone.0124808.s006.zip › Drawings - Imagined stimulation/toe_back/Subject_16_toe_back.jpg]

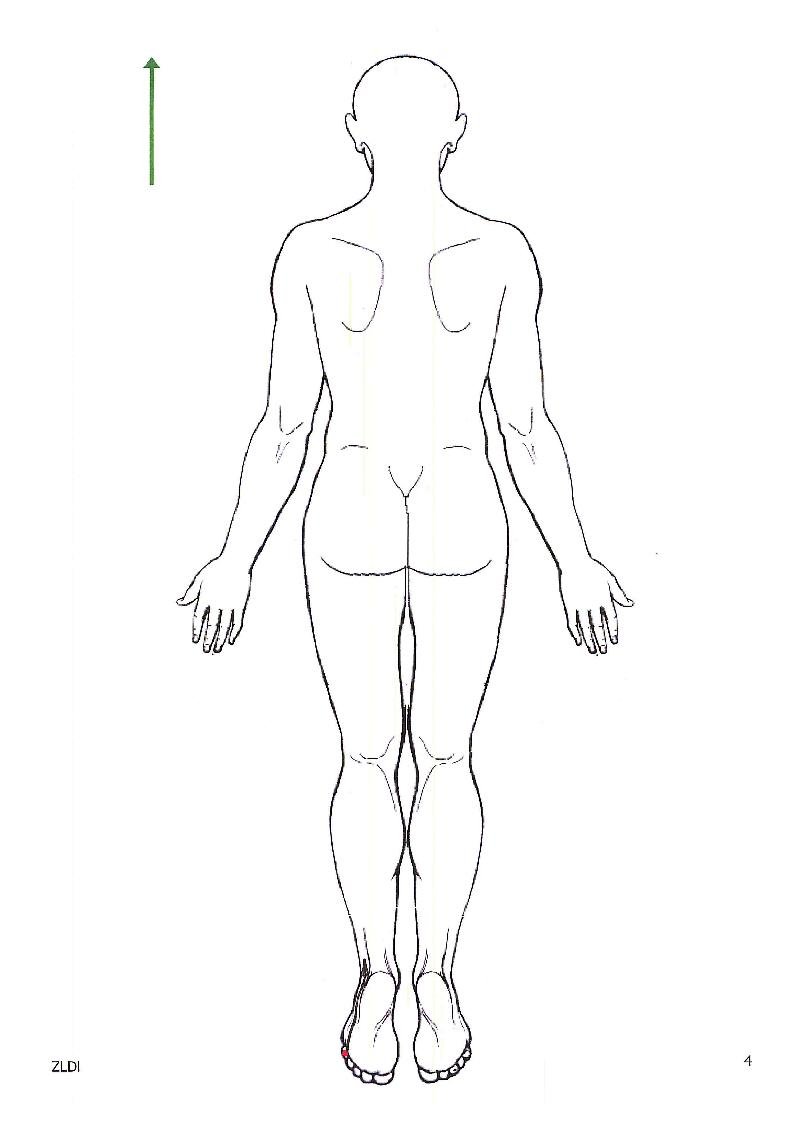

Supplement: S1 Raw Data — (ZIP) [file pone.0124808.s006.zip › Drawings - Imagined stimulation/toe_back/Subject_20_toe_back.jpg]

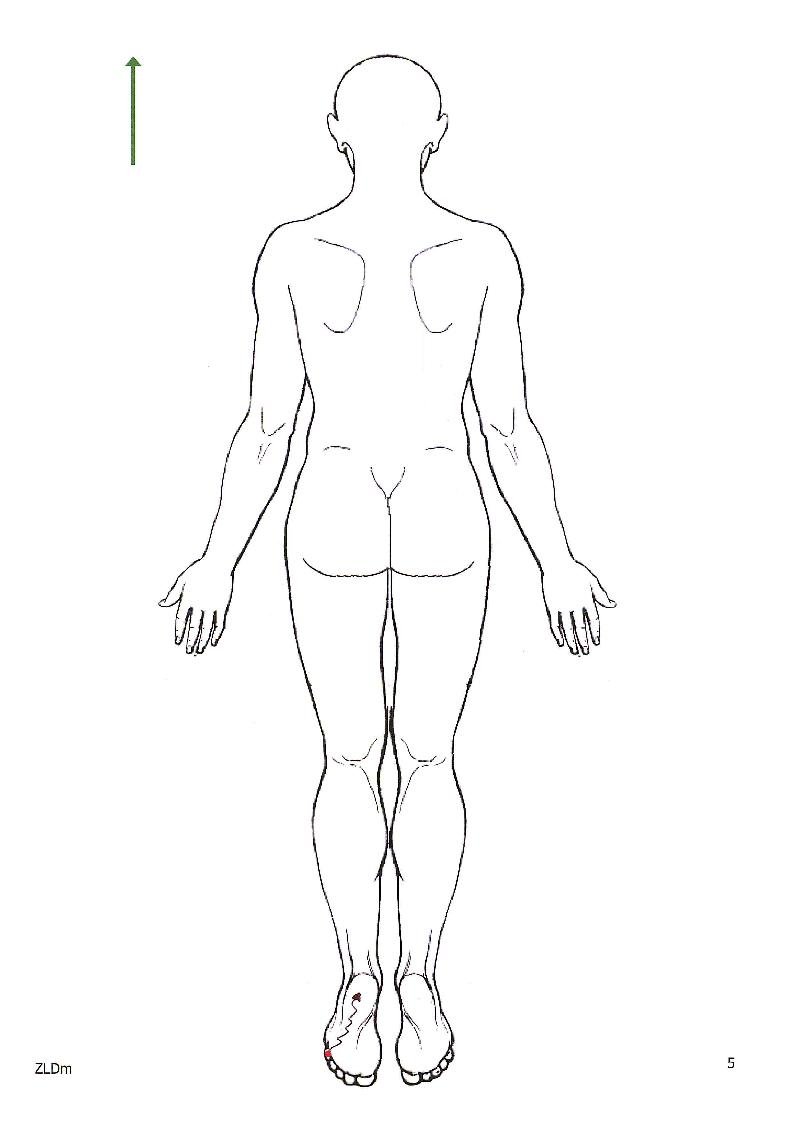

Supplement: S1 Raw Data — (ZIP) [file pone.0124808.s006.zip › Drawings - Imagined stimulation/toe_back/Subject_39_toe_back.jpg]

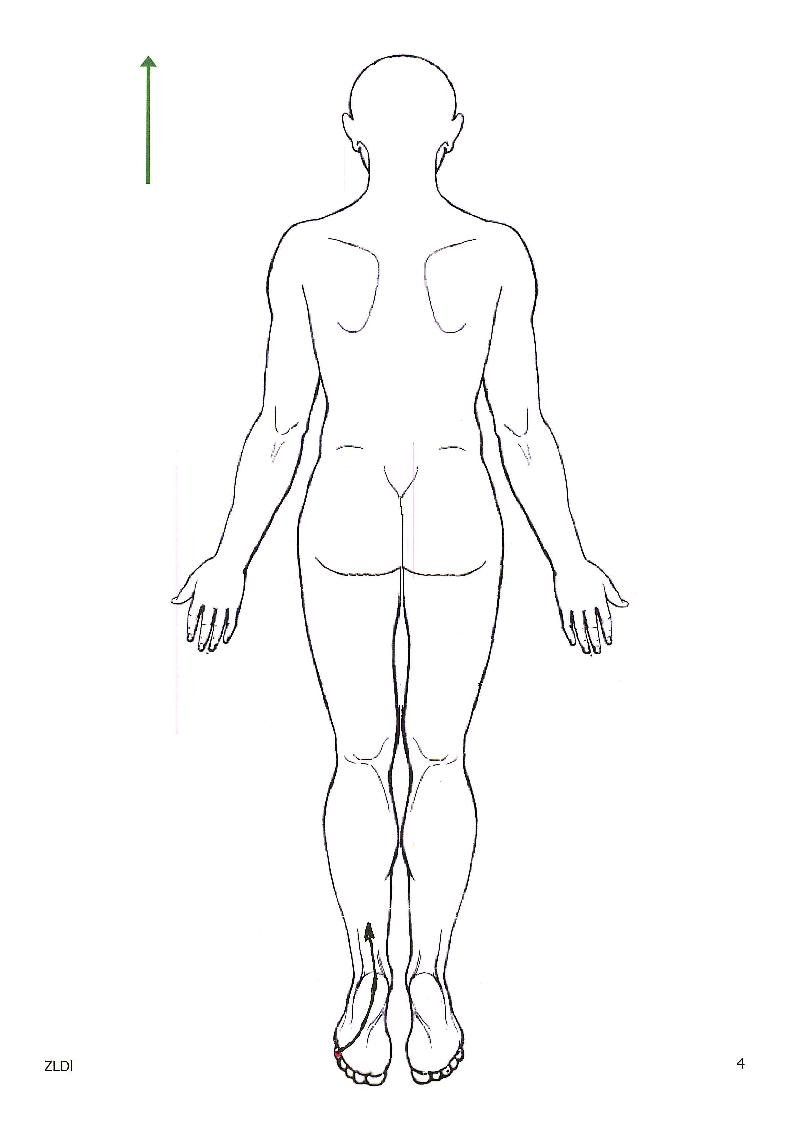

Supplement: S1 Raw Data — (ZIP) [file pone.0124808.s006.zip › Drawings - Imagined stimulation/toe_back/Subject_17_toe_back.jpg]

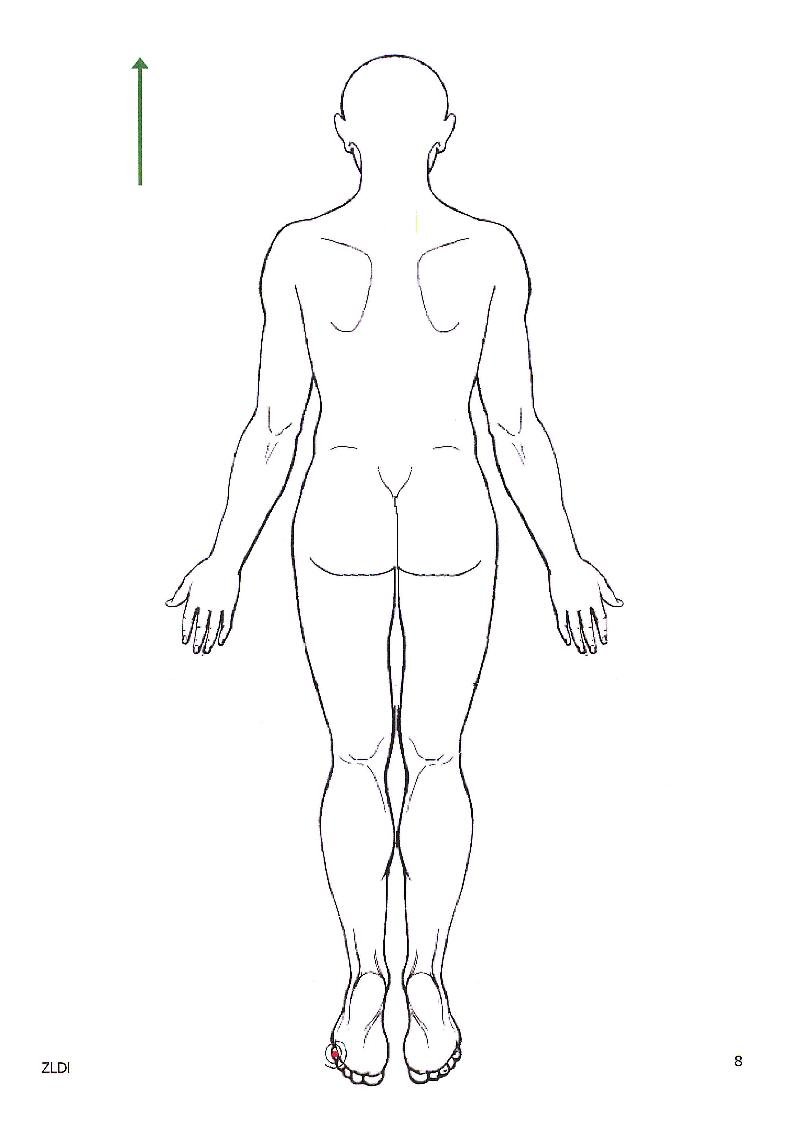

Supplement: S1 Raw Data — (ZIP) [file pone.0124808.s006.zip › Drawings - Imagined stimulation/toe_back/Subject_19_toe_back.jpg]

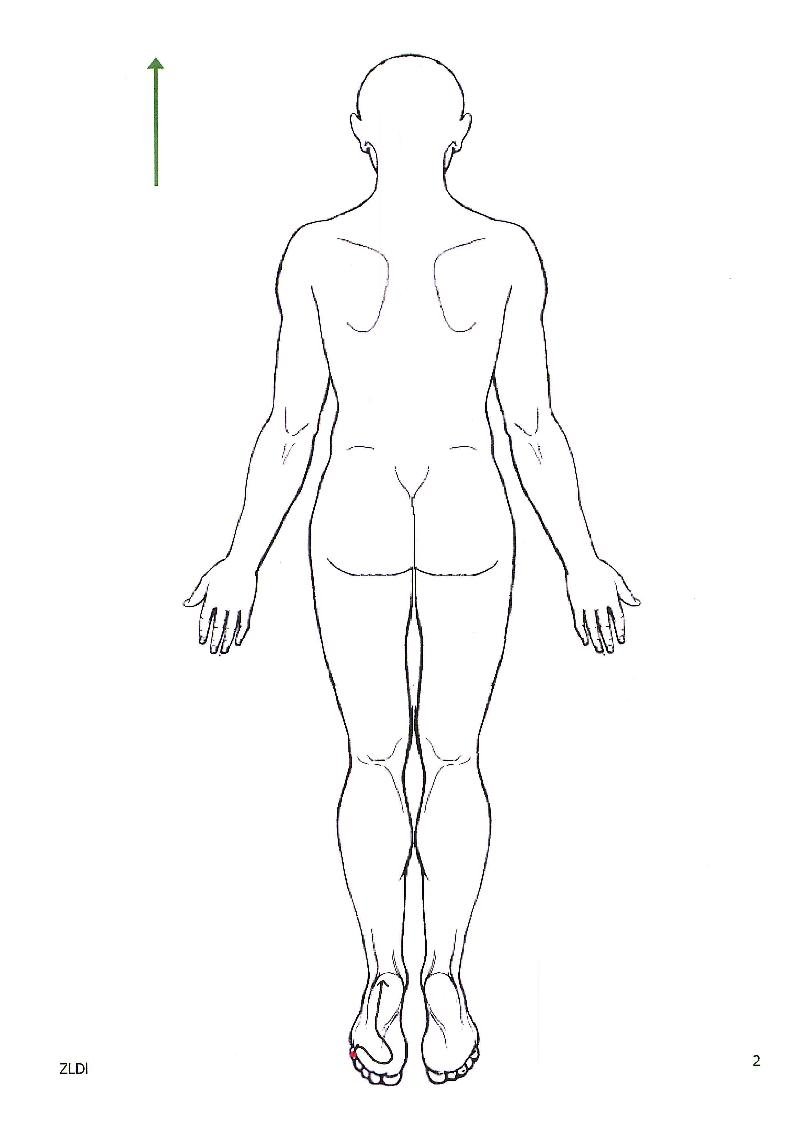

Supplement: S1 Raw Data — (ZIP) [file pone.0124808.s006.zip › Drawings - Imagined stimulation/toe_back/Subject_22_toe_back.jpg]

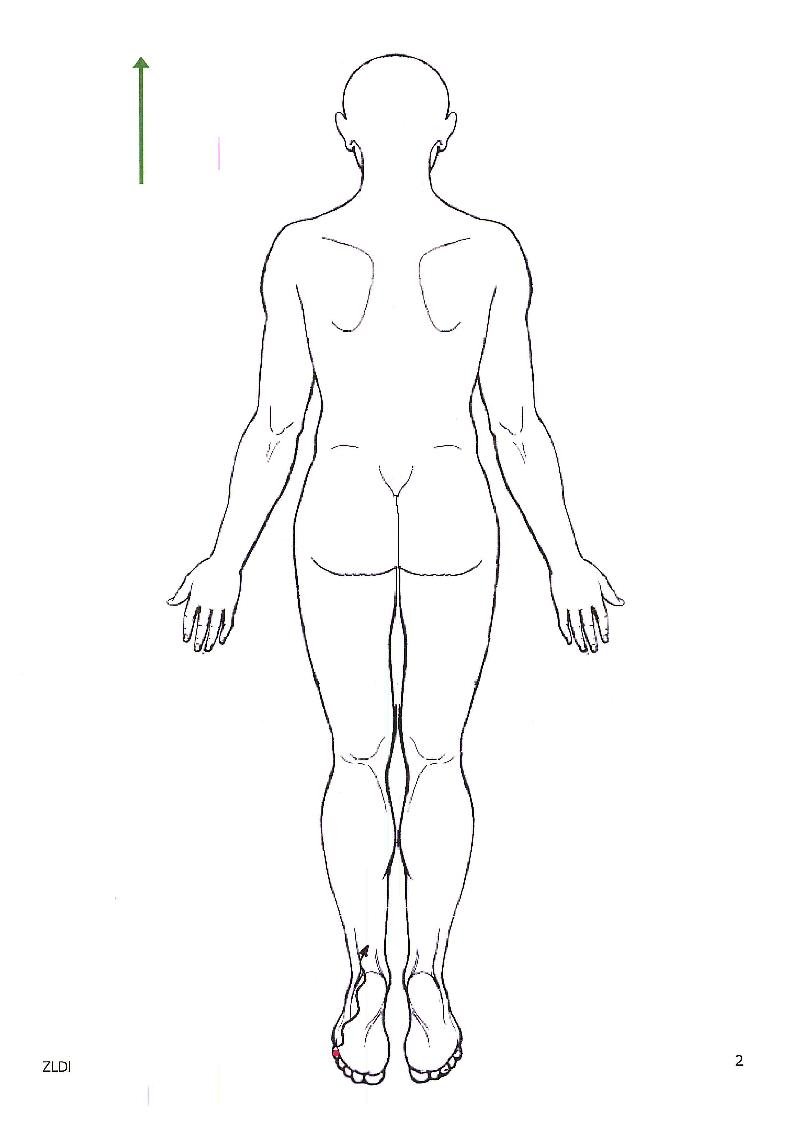

Supplement: S1 Raw Data — (ZIP) [file pone.0124808.s006.zip › Drawings - Imagined stimulation/toe_back/Subject_25_toe_back.jpg]

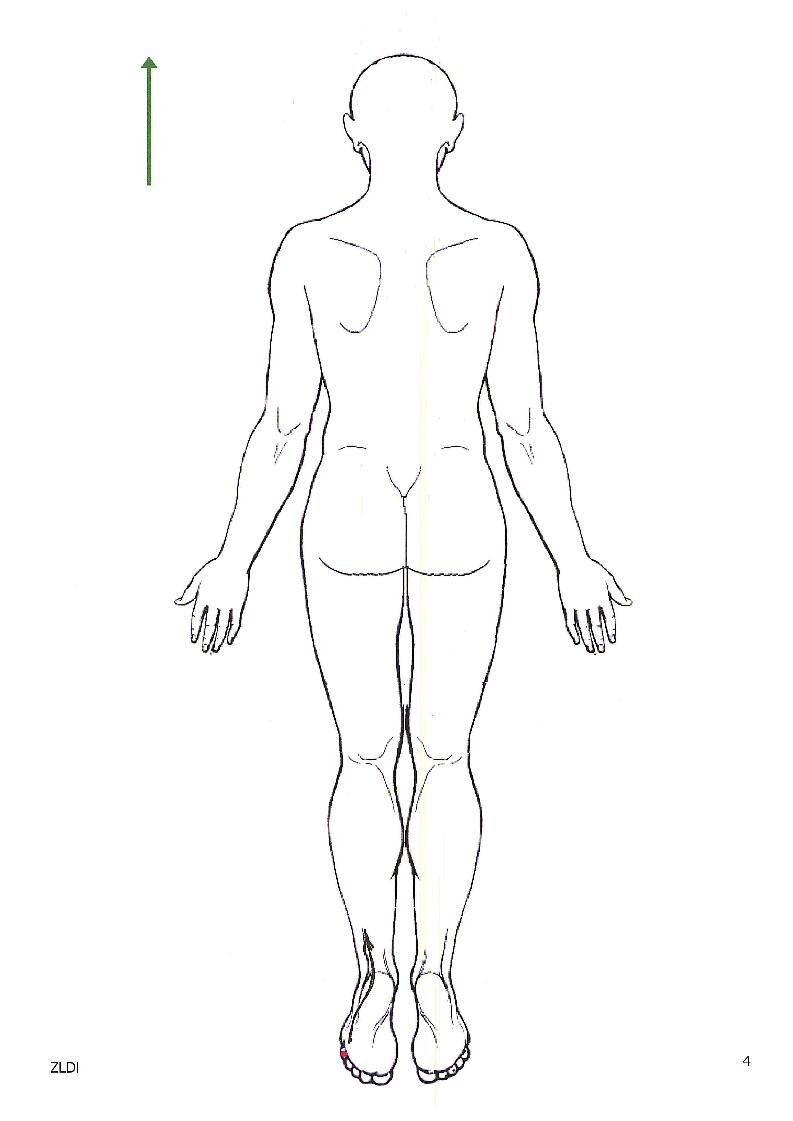

Supplement: S1 Raw Data — (ZIP) [file pone.0124808.s006.zip › Drawings - Imagined stimulation/toe_back/Subject_53_toe_back.jpg]

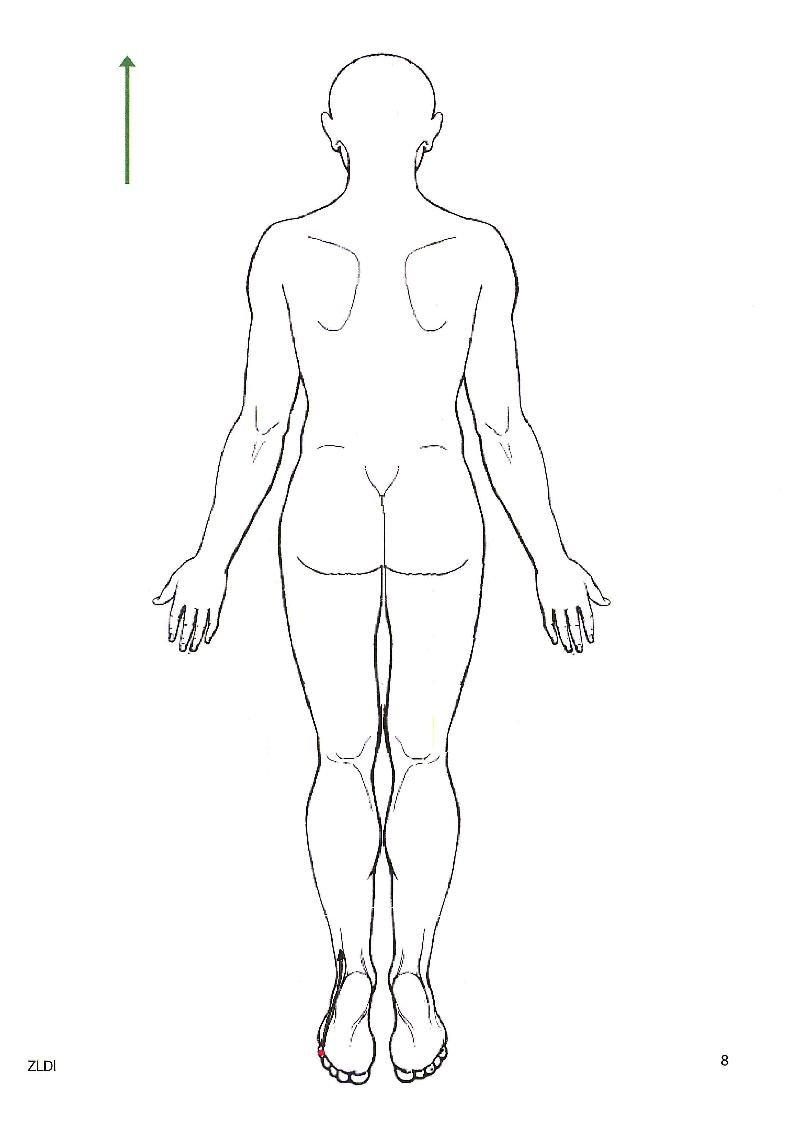

Supplement: S1 Raw Data — (ZIP) [file pone.0124808.s006.zip › Drawings - Imagined stimulation/toe_back/Subject_54_toe_back.jpg]

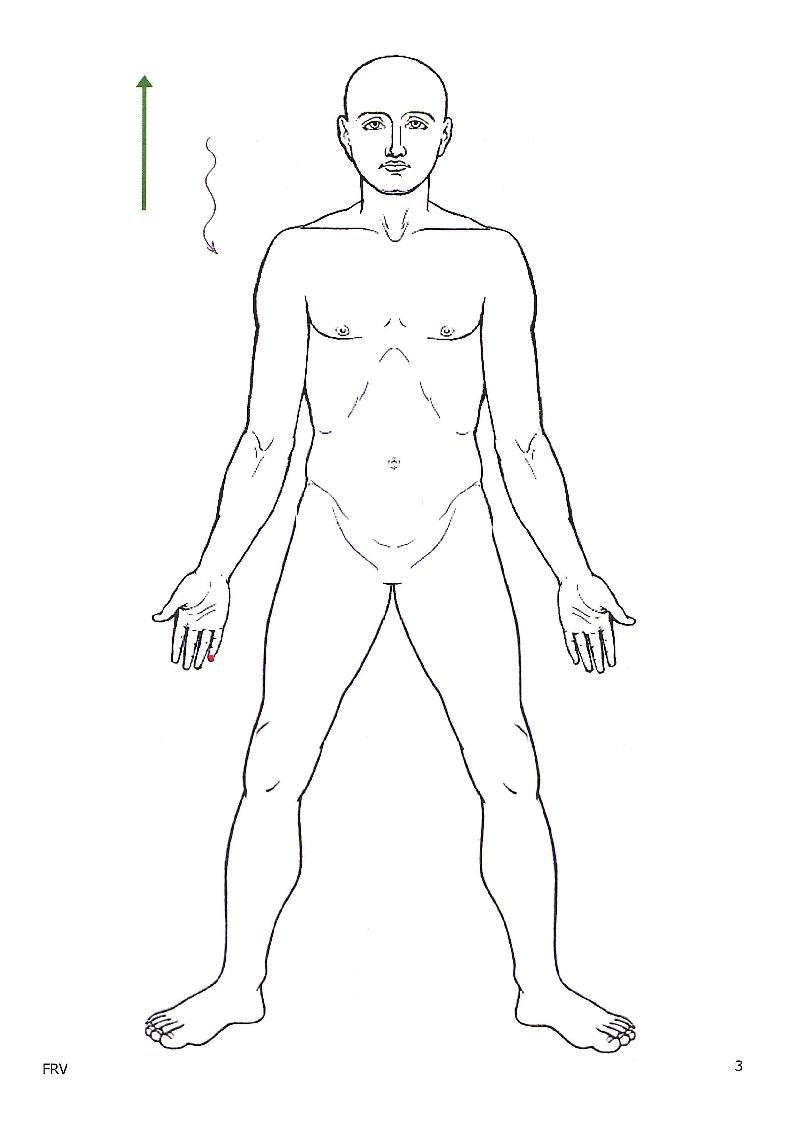

Supplement: S1 Raw Data — (ZIP) [file pone.0124808.s006.zip › Drawings - Imagined stimulation/finger_front/Subject_52_finger_front.jpg]

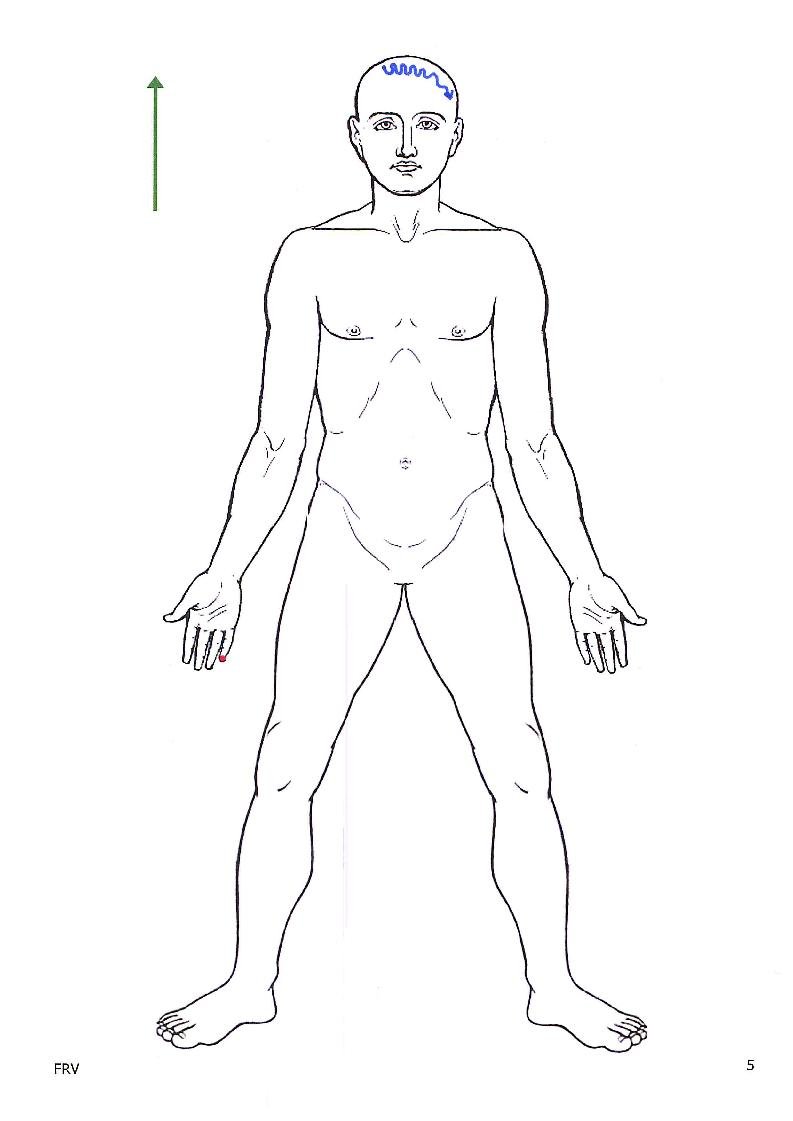

Supplement: S1 Raw Data — (ZIP) [file pone.0124808.s006.zip › Drawings - Imagined stimulation/finger_front/Subject_14_finger_front.jpg]

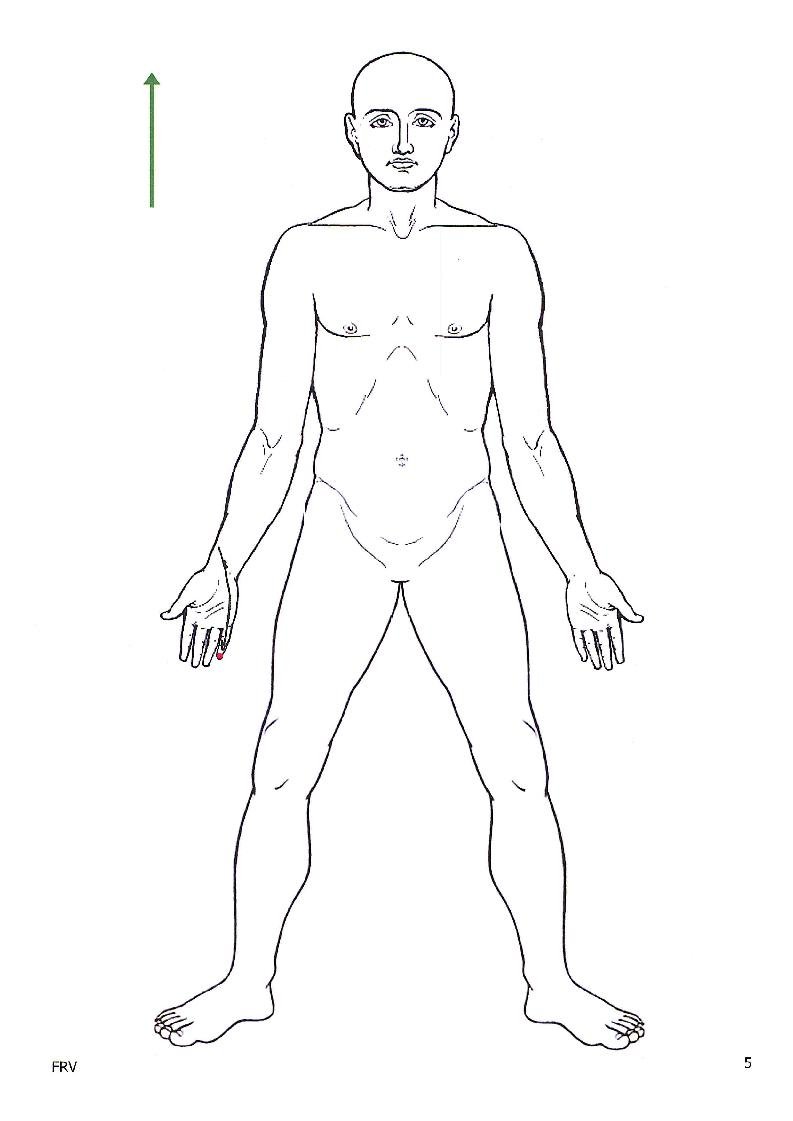

Supplement: S1 Raw Data — (ZIP) [file pone.0124808.s006.zip › Drawings - Imagined stimulation/finger_front/Subject_26_finger_front.jpg]

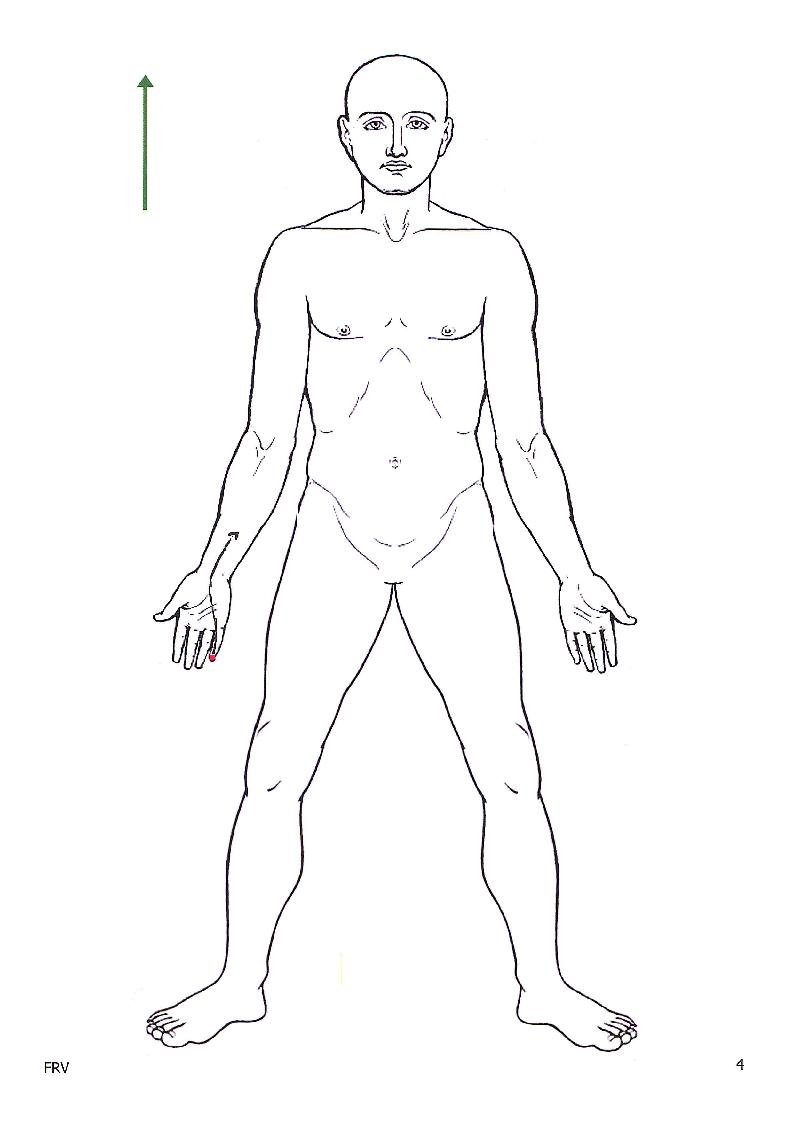

Supplement: S1 Raw Data — (ZIP) [file pone.0124808.s006.zip › Drawings - Imagined stimulation/finger_front/Subject_32_finger_front.jpg]

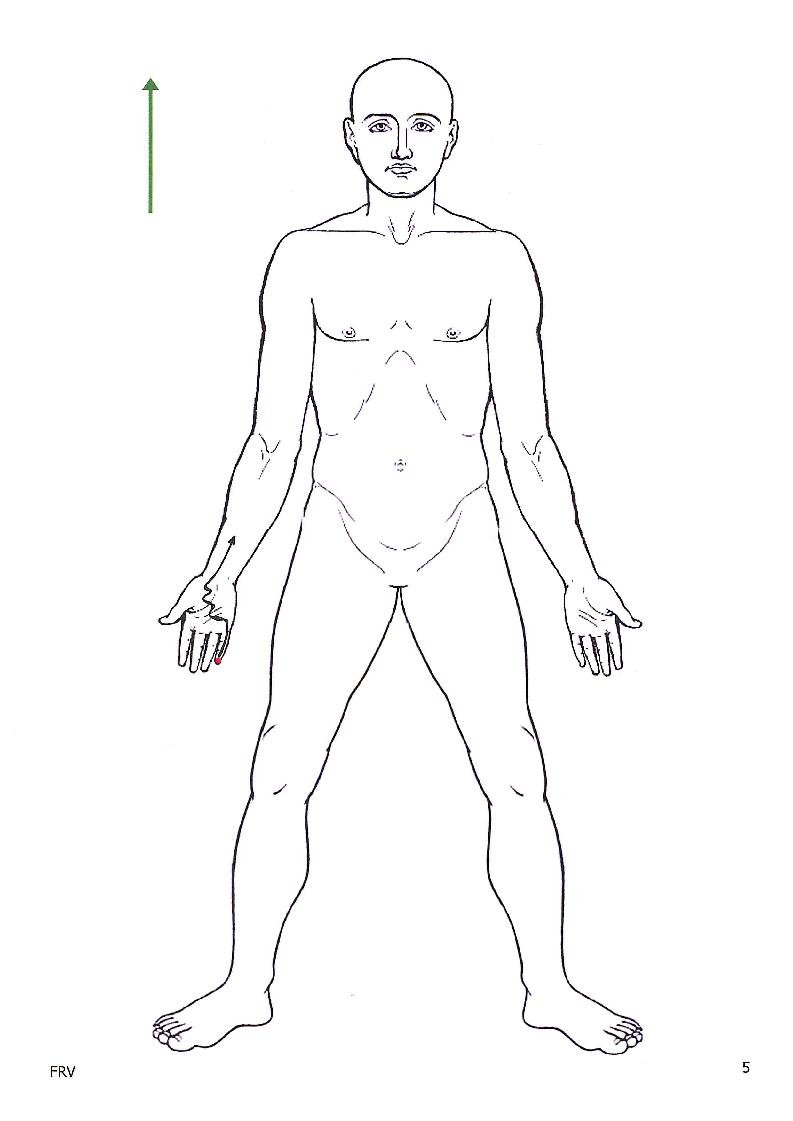

Supplement: S1 Raw Data — (ZIP) [file pone.0124808.s006.zip › Drawings - Imagined stimulation/finger_front/Subject_27_finger_front.jpg]

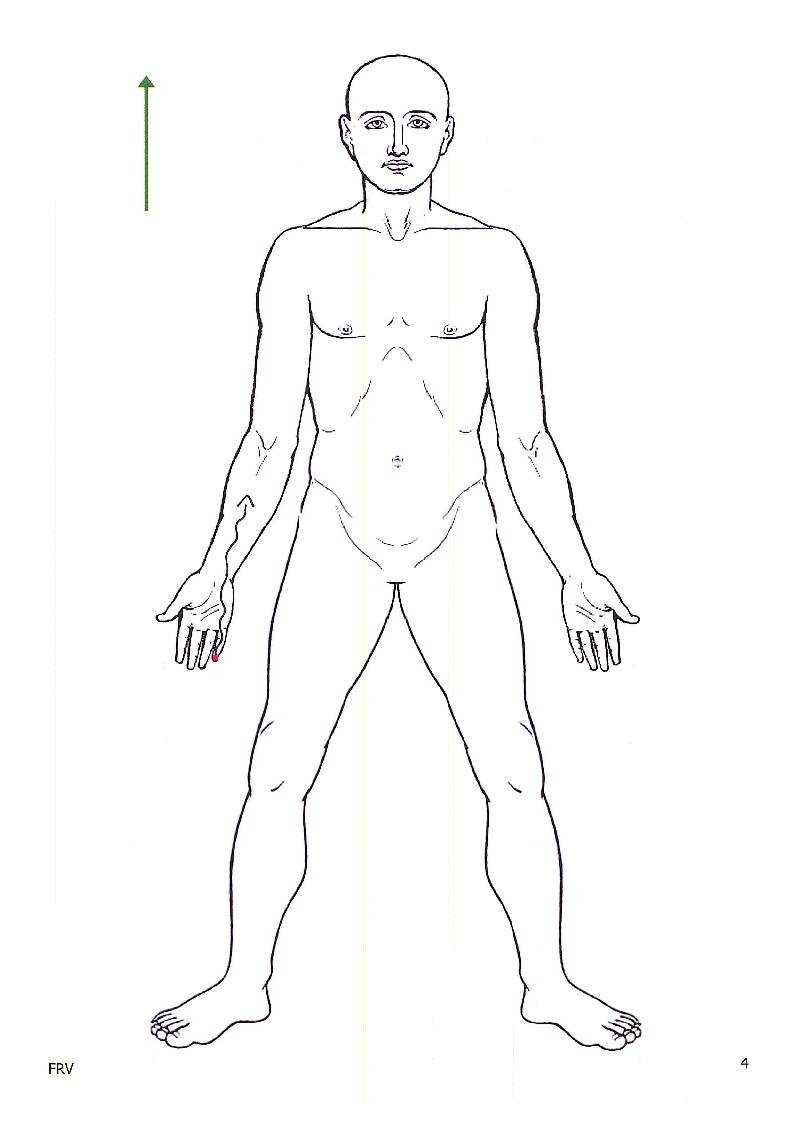

Supplement: S1 Raw Data — (ZIP) [file pone.0124808.s006.zip › Drawings - Imagined stimulation/finger_front/Subject_5_finger_front.jpg]

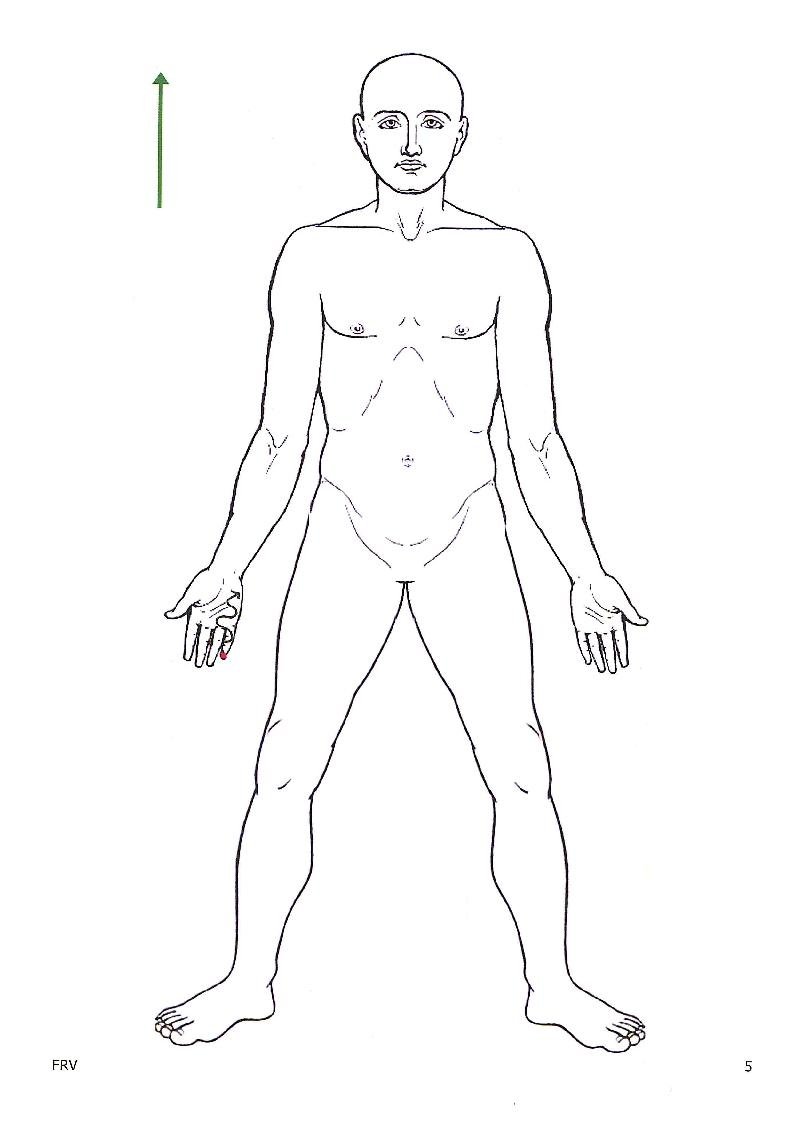

Supplement: S1 Raw Data — (ZIP) [file pone.0124808.s006.zip › Drawings - Imagined stimulation/finger_front/Subject_33_finger_front.jpg]

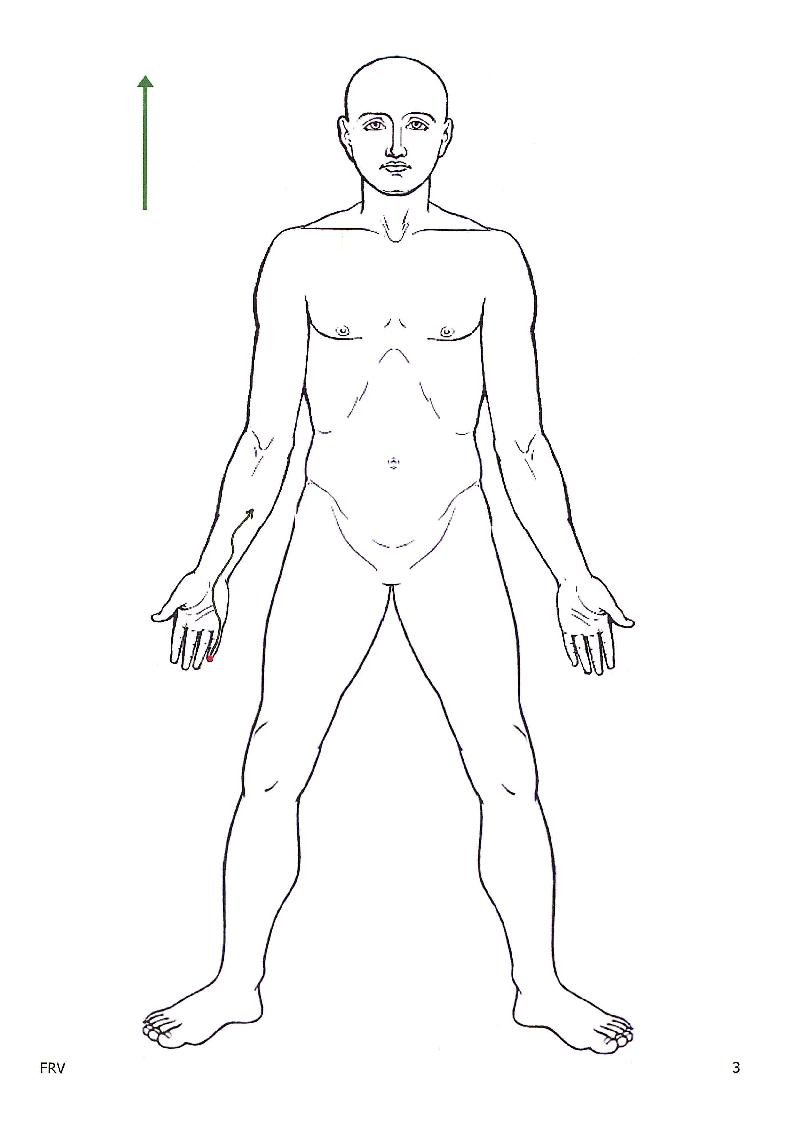

Supplement: S1 Raw Data — (ZIP) [file pone.0124808.s006.zip › Drawings - Imagined stimulation/finger_front/Subject_7_finger_front.jpg]

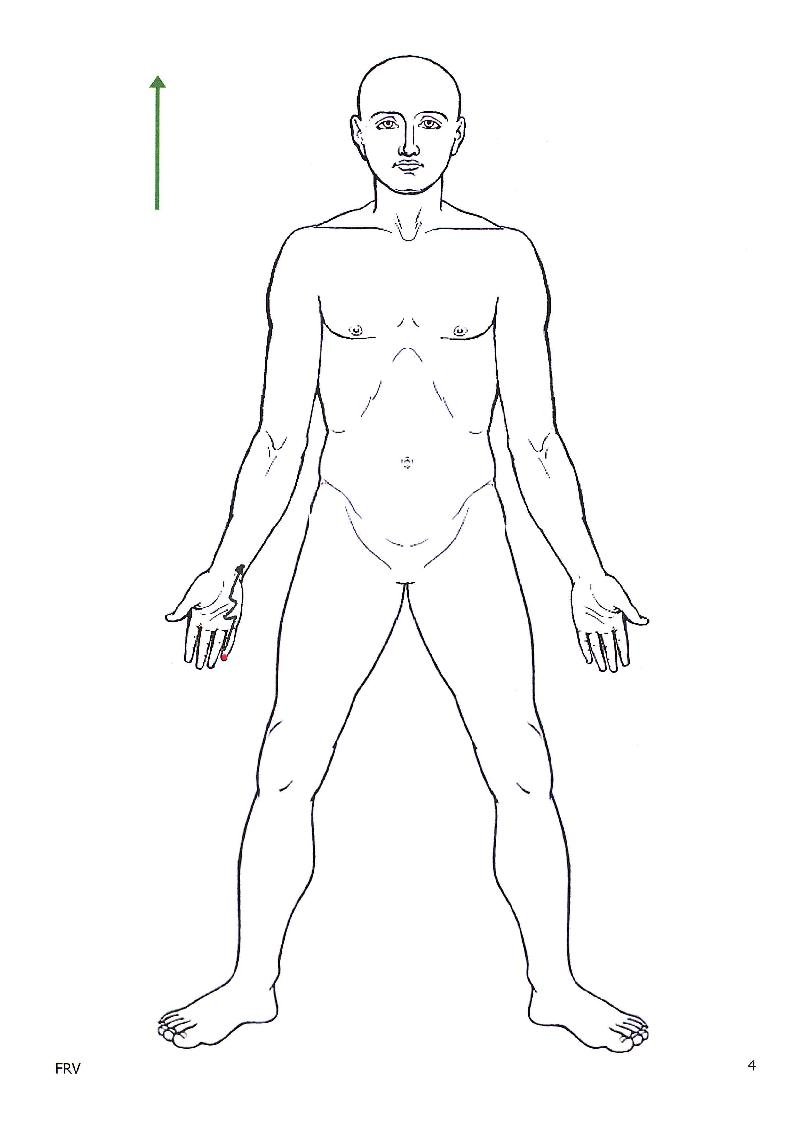

Supplement: S1 Raw Data — (ZIP) [file pone.0124808.s006.zip › Drawings - Imagined stimulation/finger_front/Subject_28_finger_front.jpg]

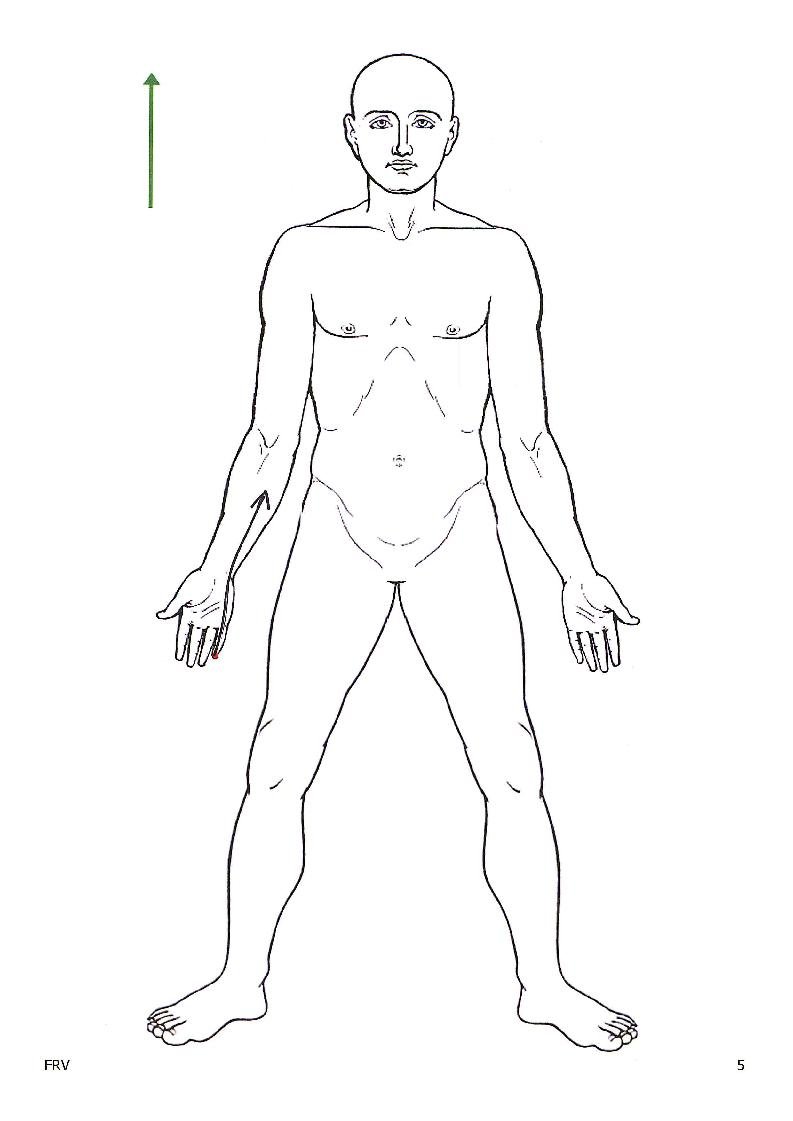

Supplement: S1 Raw Data — (ZIP) [file pone.0124808.s006.zip › Drawings - Imagined stimulation/finger_front/Subject_8_finger_front.jpg]

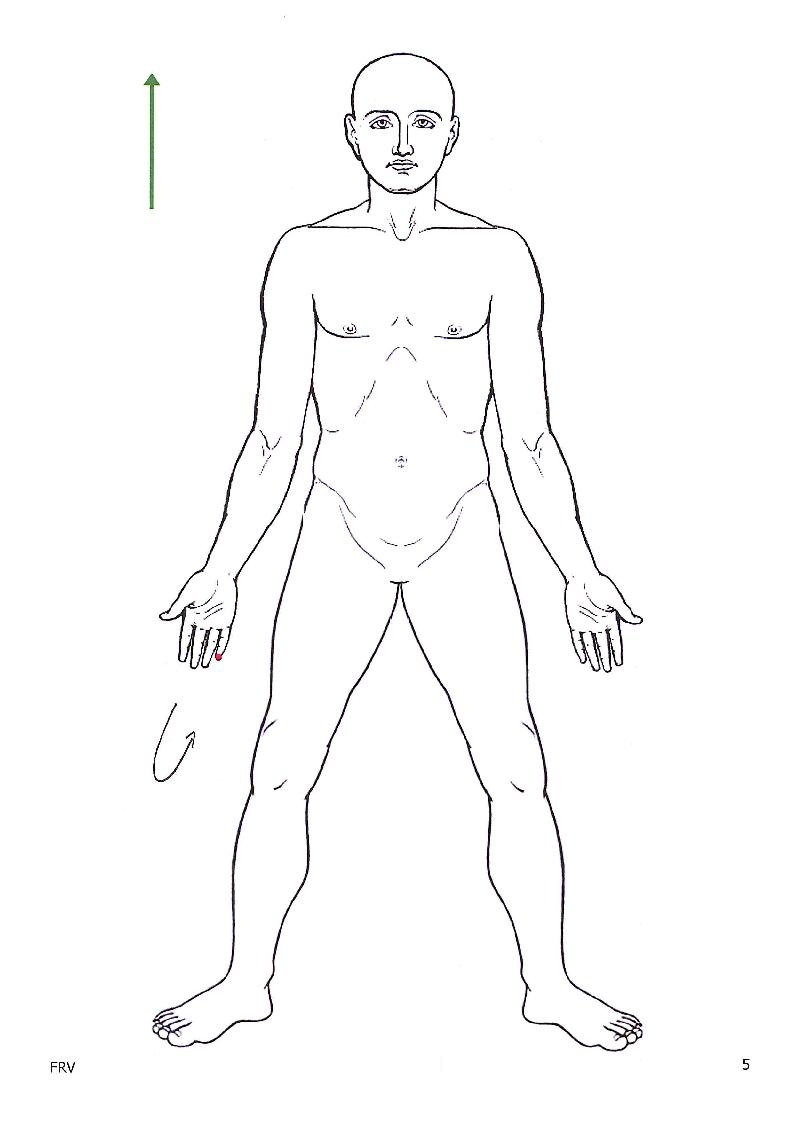

Supplement: S1 Raw Data — (ZIP) [file pone.0124808.s006.zip › Drawings - Imagined stimulation/finger_front/Subject_34_finger_front.jpg]

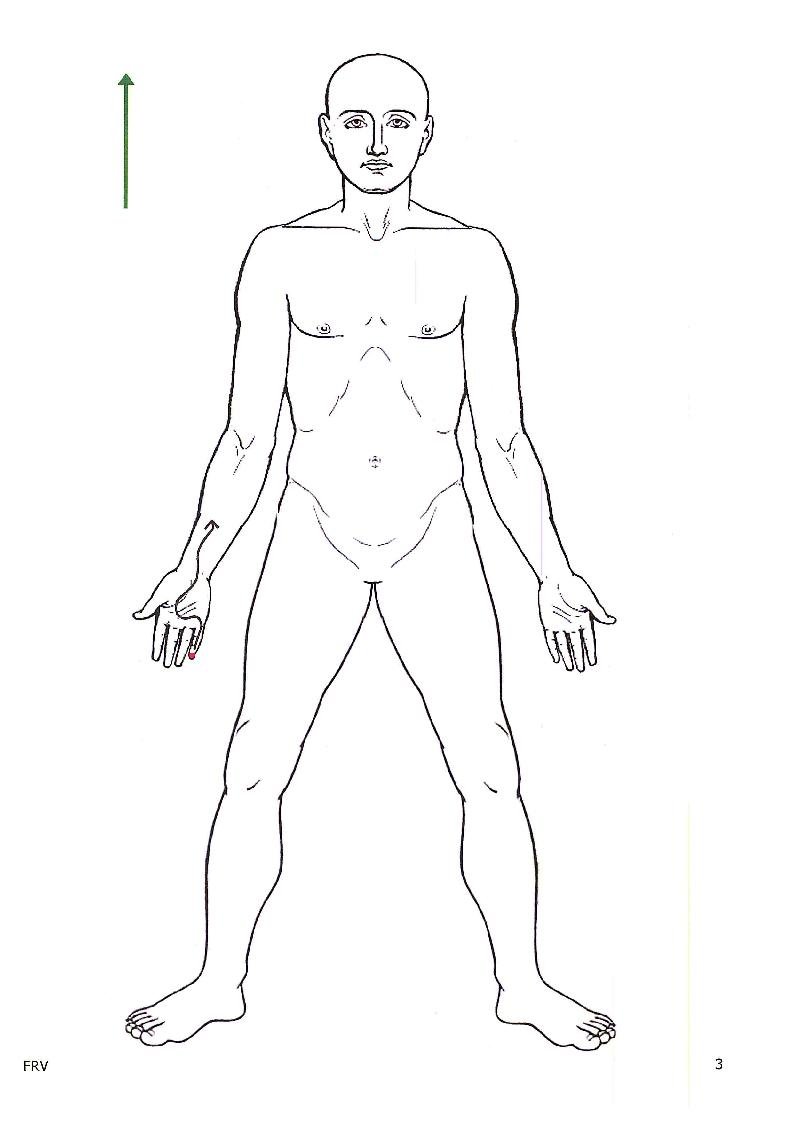

Supplement: S1 Raw Data — (ZIP) [file pone.0124808.s006.zip › Drawings - Imagined stimulation/finger_front/Subject_29_finger_front.jpg]

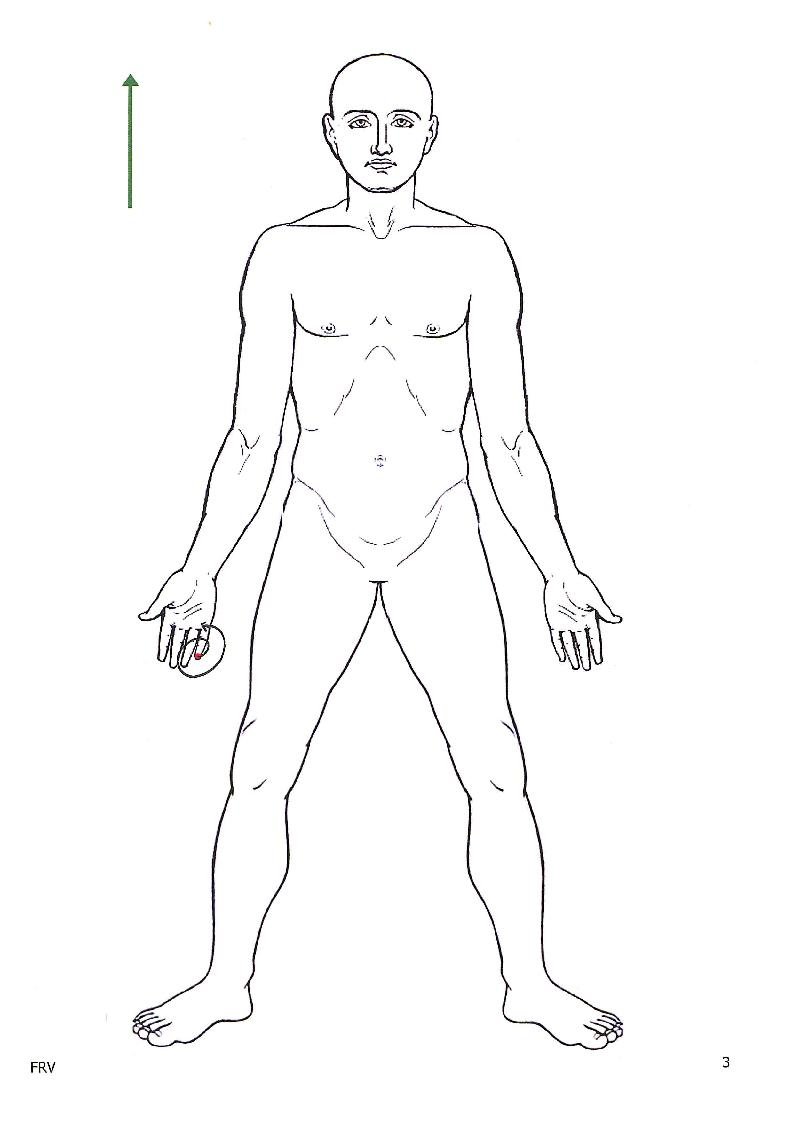

Supplement: S1 Raw Data — (ZIP) [file pone.0124808.s006.zip › Drawings - Imagined stimulation/finger_front/Subject_40_finger_front.jpg]

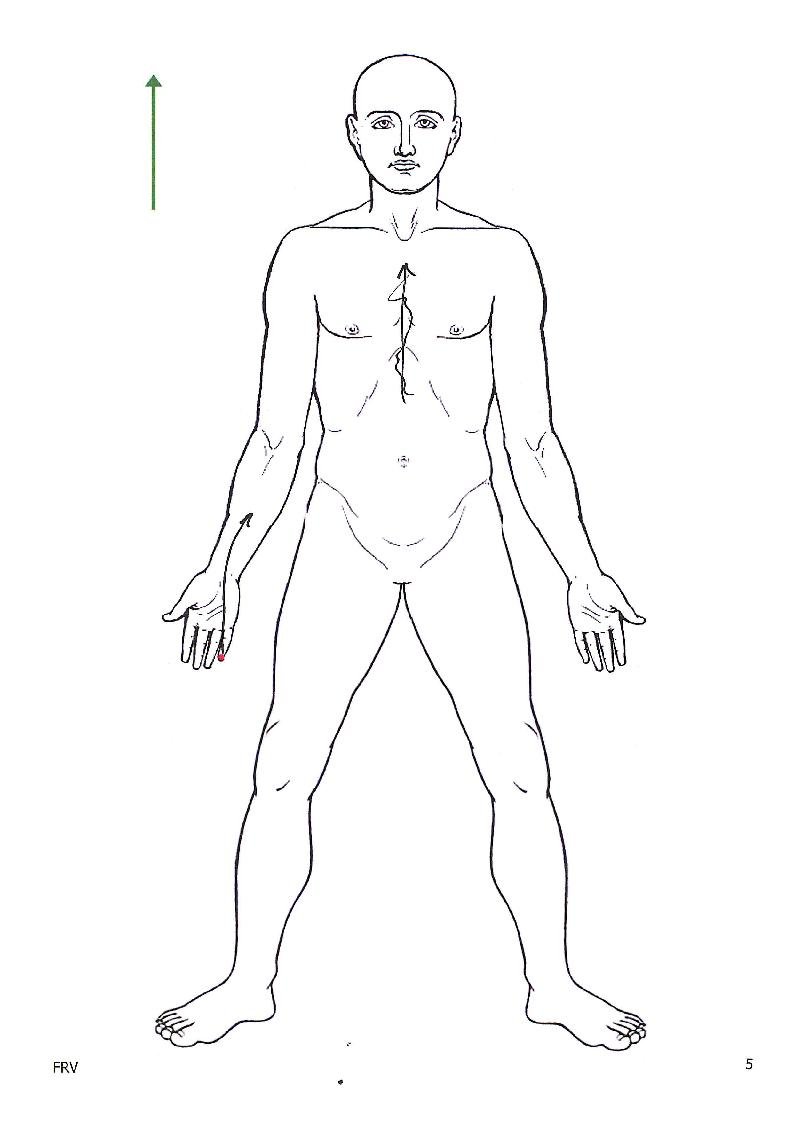

Supplement: S1 Raw Data — (ZIP) [file pone.0124808.s006.zip › Drawings - Imagined stimulation/finger_front/Subject_35_finger_front.jpg]

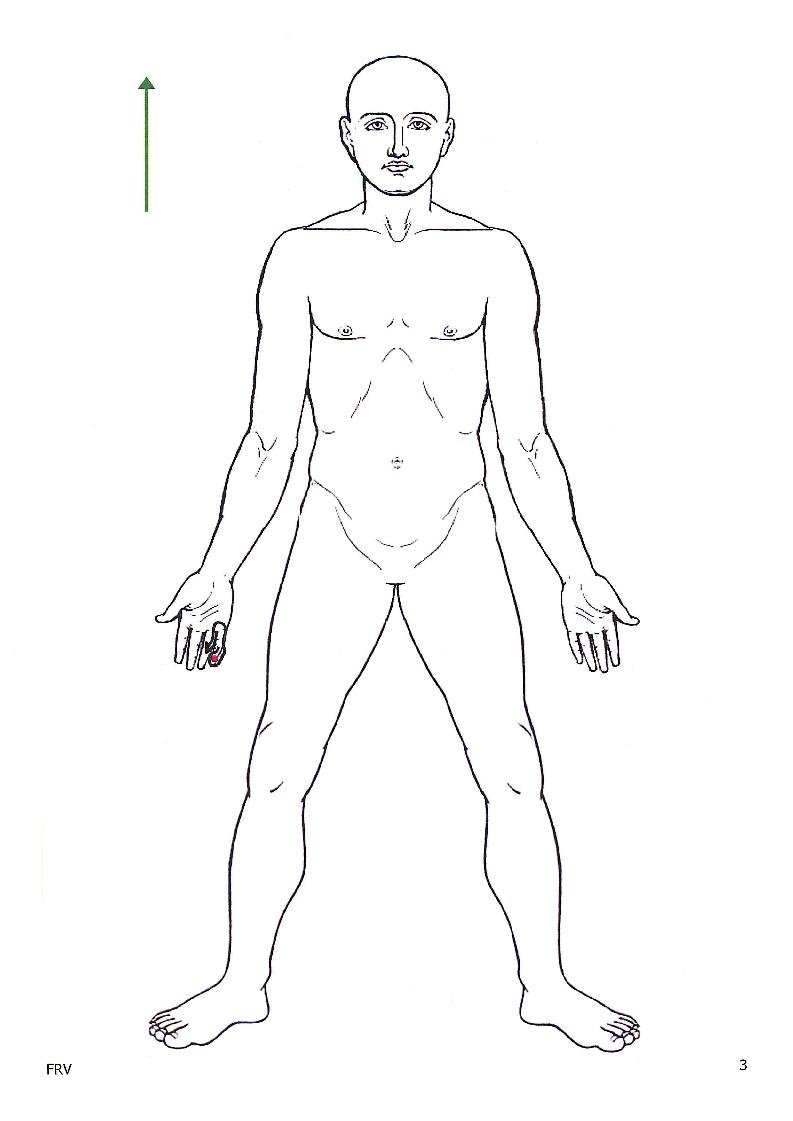

Supplement: S1 Raw Data — (ZIP) [file pone.0124808.s006.zip › Drawings - Imagined stimulation/finger_front/Subject_41_finger_front.jpg]

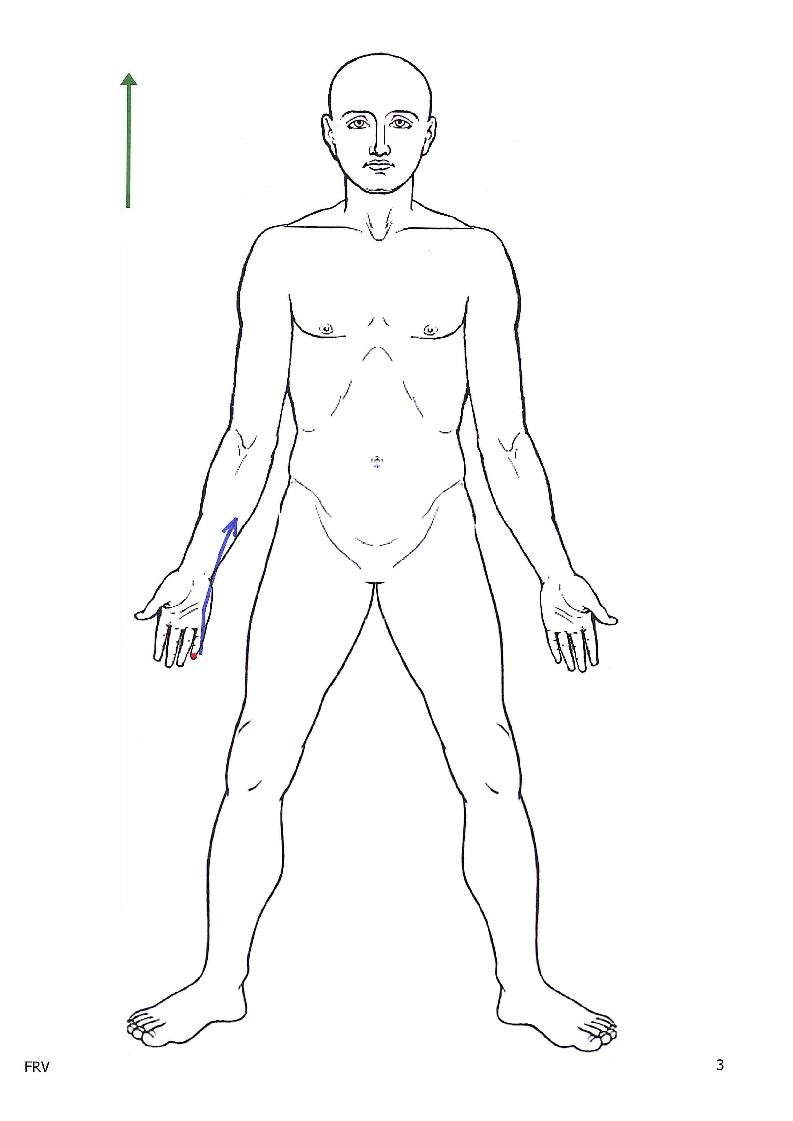

Supplement: S1 Raw Data — (ZIP) [file pone.0124808.s006.zip › Drawings - Imagined stimulation/finger_front/Subject_36_finger_front.jpg]

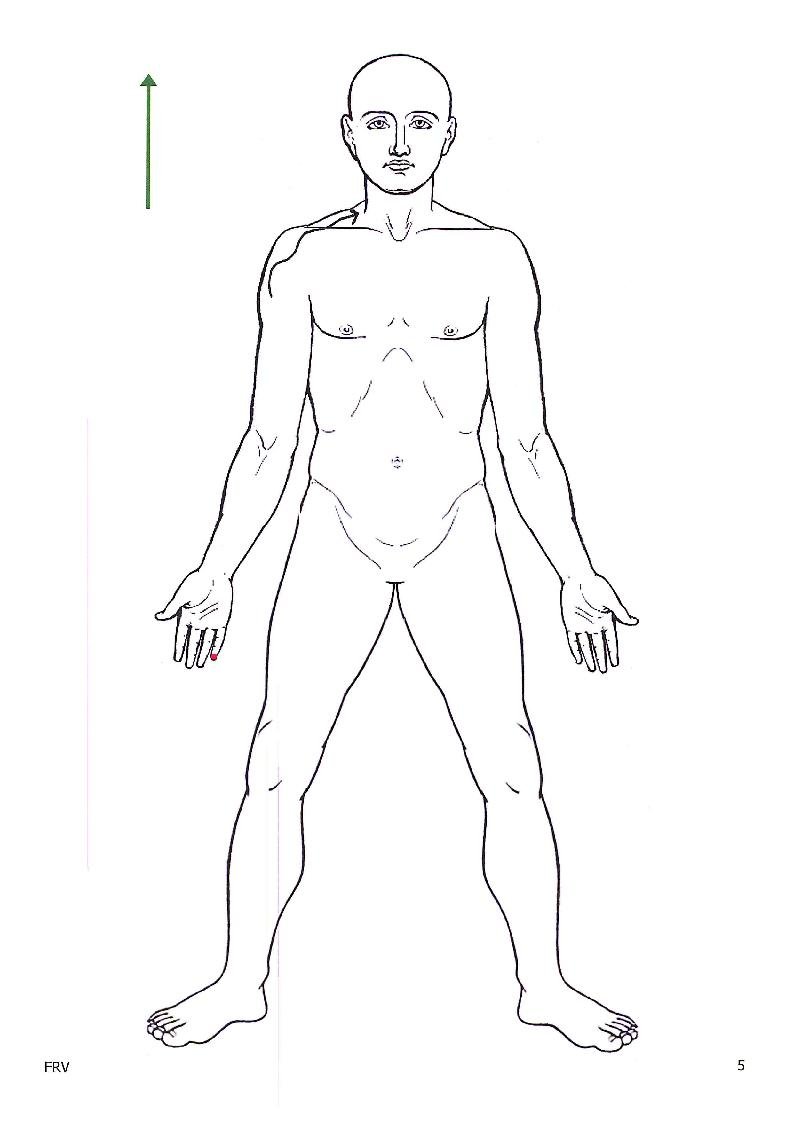

Supplement: S1 Raw Data — (ZIP) [file pone.0124808.s006.zip › Drawings - Imagined stimulation/finger_front/Subject_11_finger_front.jpg]

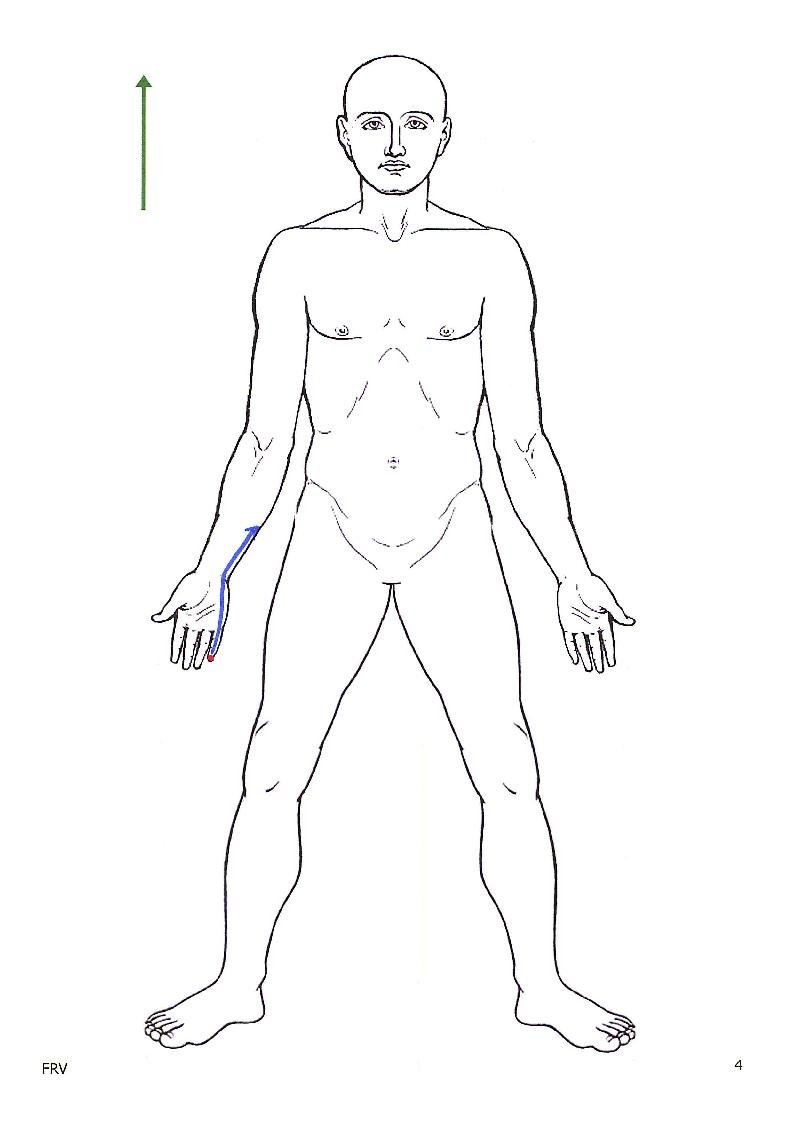

Supplement: S1 Raw Data — (ZIP) [file pone.0124808.s006.zip › Drawings - Imagined stimulation/finger_front/Subject_42_finger_front.jpg]

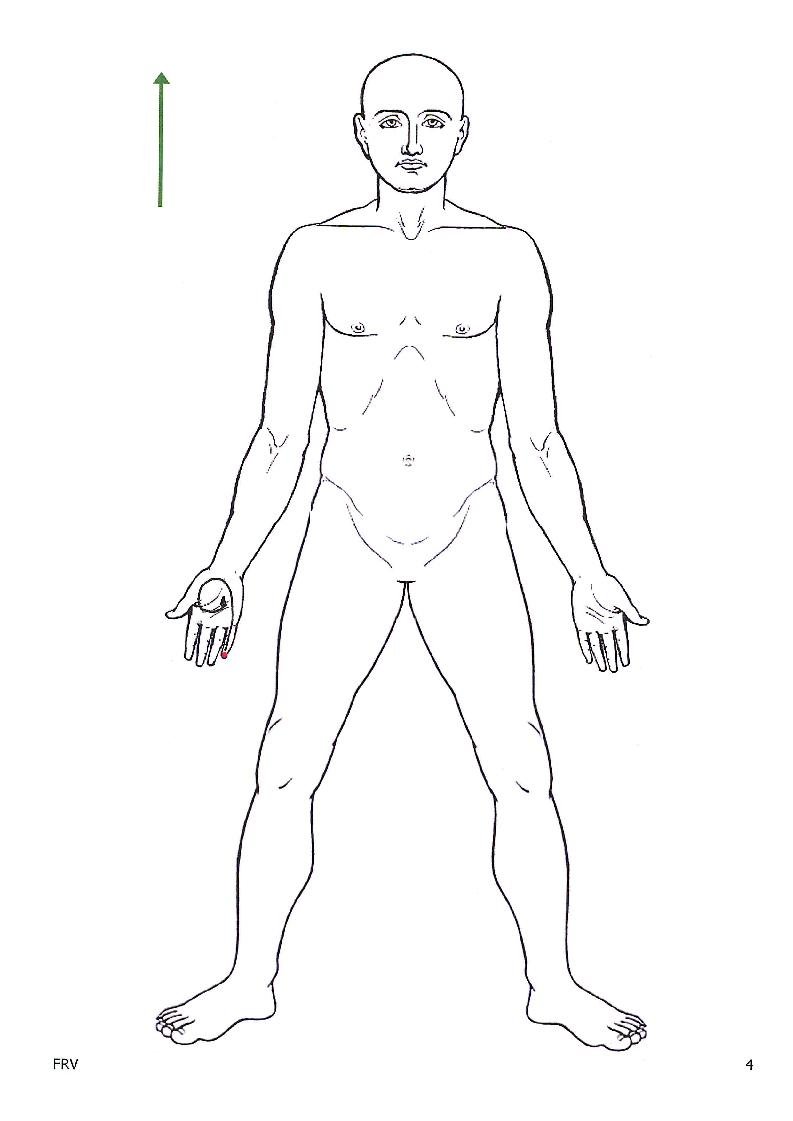

Supplement: S1 Raw Data — (ZIP) [file pone.0124808.s006.zip › Drawings - Imagined stimulation/finger_front/Subject_25_finger_front.jpg]

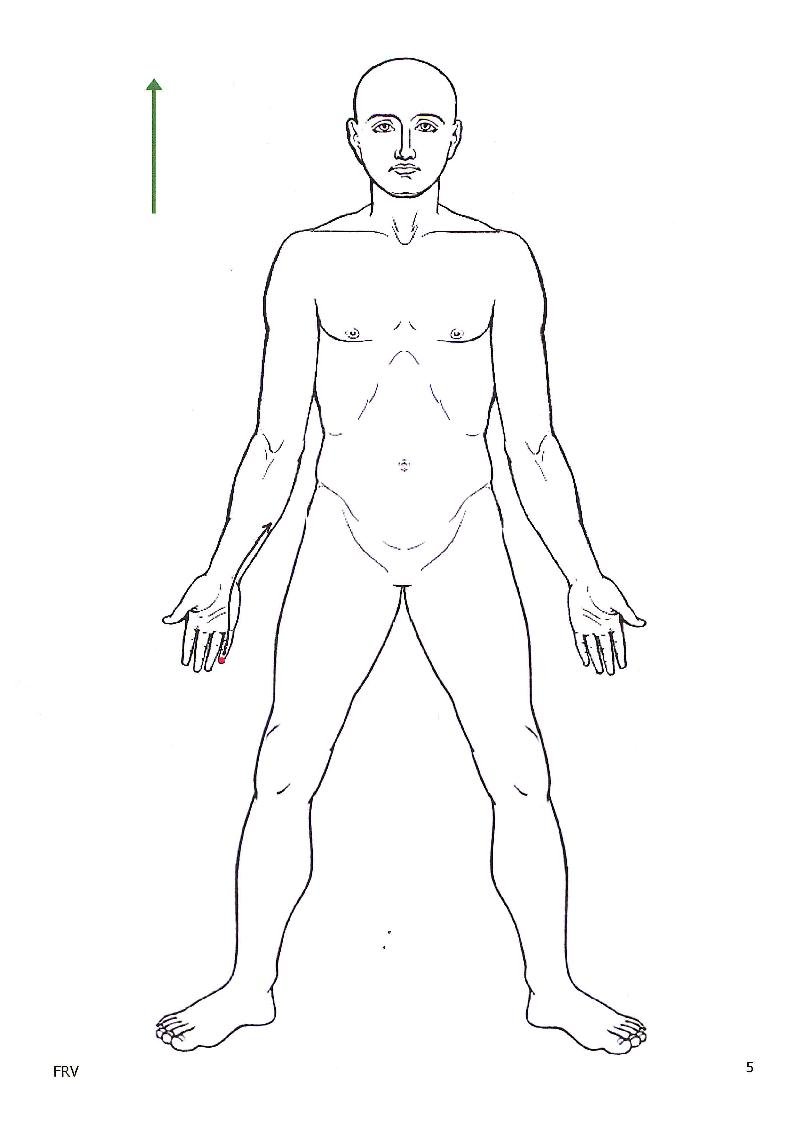

Supplement: S1 Raw Data — (ZIP) [file pone.0124808.s006.zip › Drawings - Imagined stimulation/finger_front/Subject_37_finger_front.jpg]

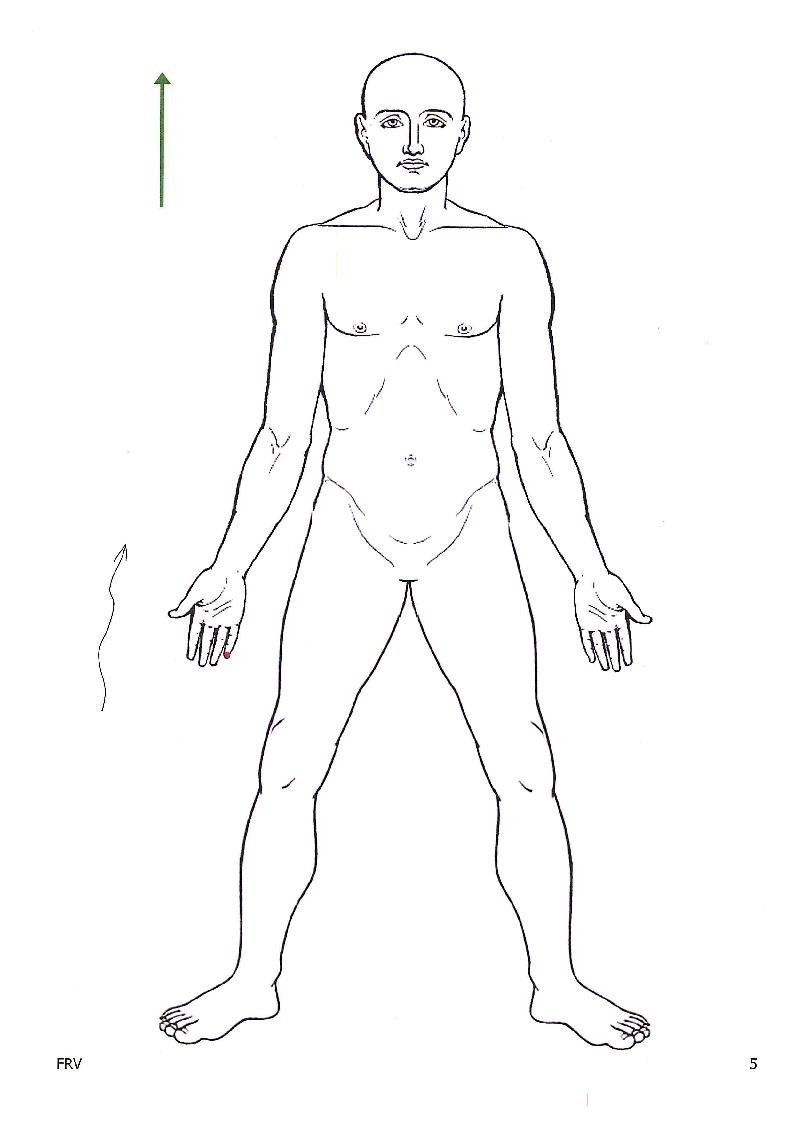

Supplement: S1 Raw Data — (ZIP) [file pone.0124808.s006.zip › Drawings - Imagined stimulation/finger_front/Subject_10_finger_front.jpg]

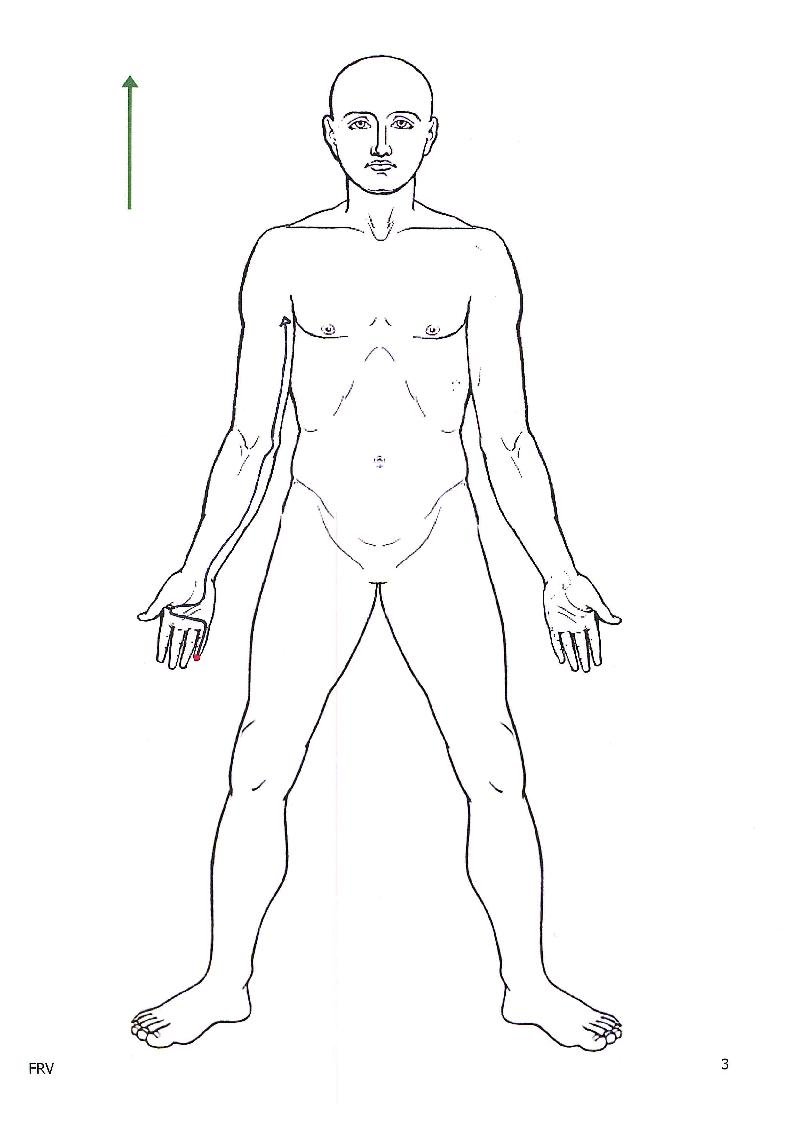

Supplement: S1 Raw Data — (ZIP) [file pone.0124808.s006.zip › Drawings - Imagined stimulation/finger_front/Subject_9_finger_front.jpg]

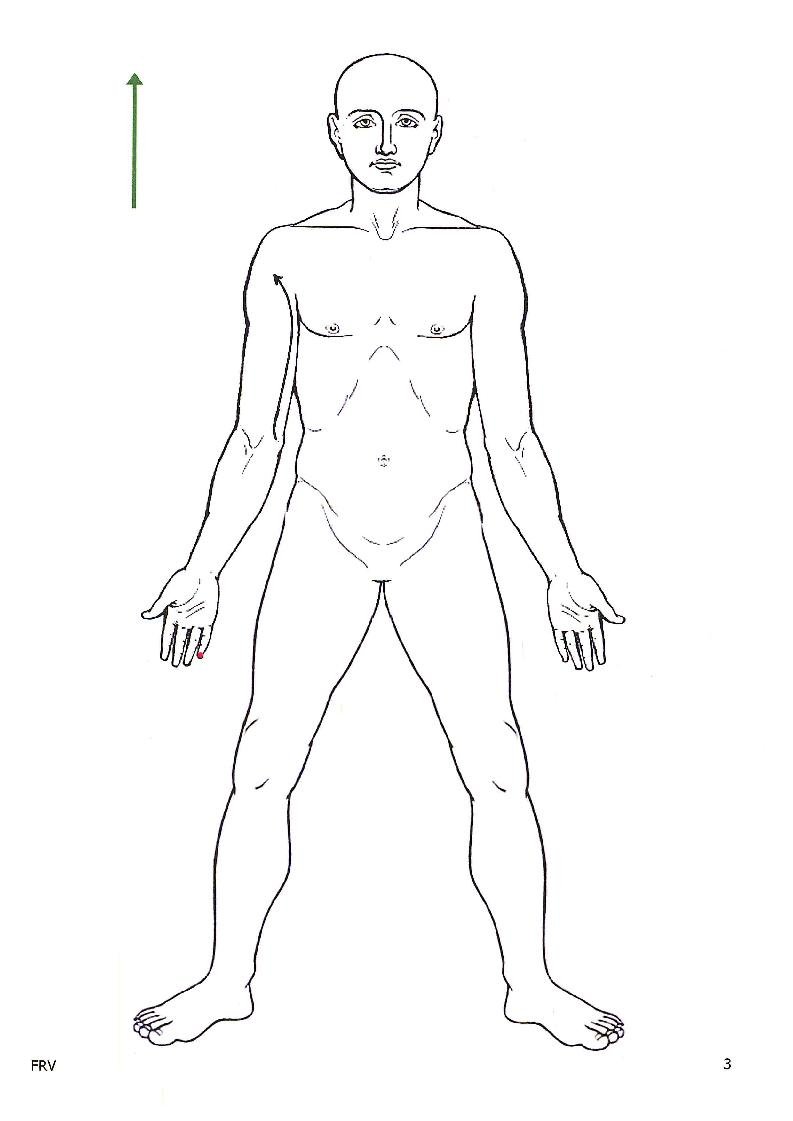

Supplement: S1 Raw Data — (ZIP) [file pone.0124808.s006.zip › Drawings - Imagined stimulation/finger_front/Subject_6_finger_front.jpg]

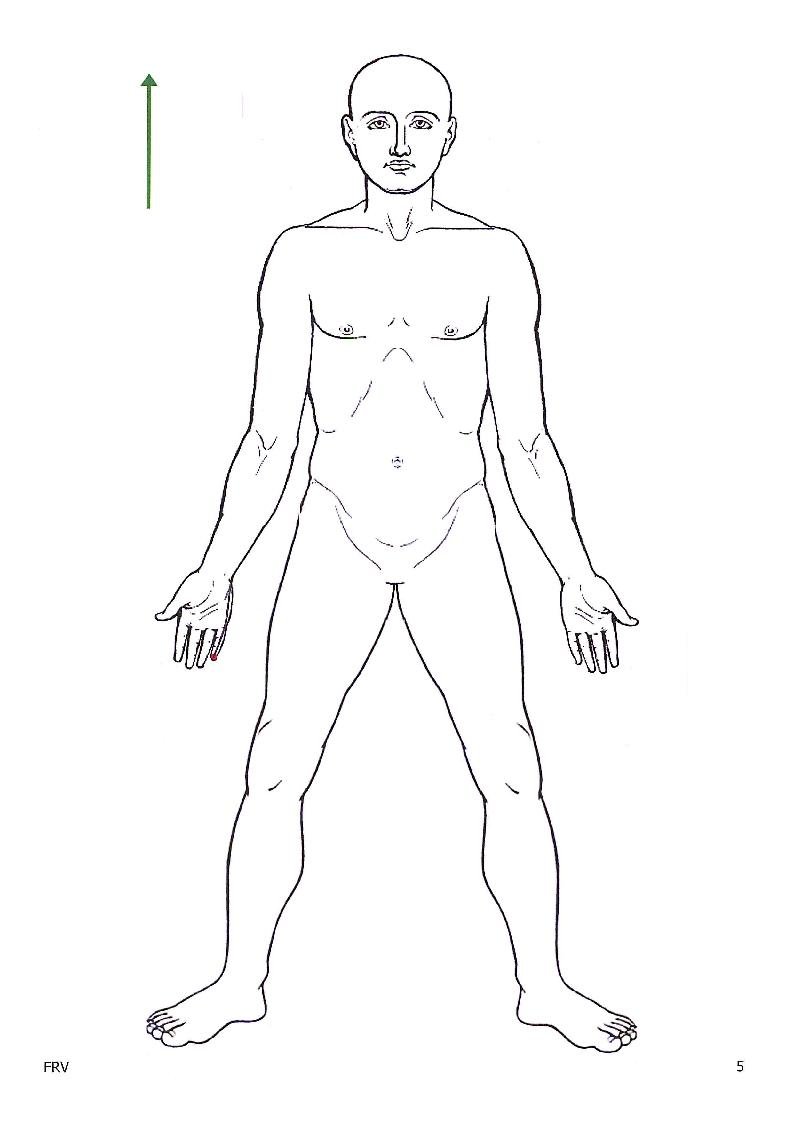

Supplement: S1 Raw Data — (ZIP) [file pone.0124808.s006.zip › Drawings - Imagined stimulation/finger_front/Subject_1_finger_front.jpg]

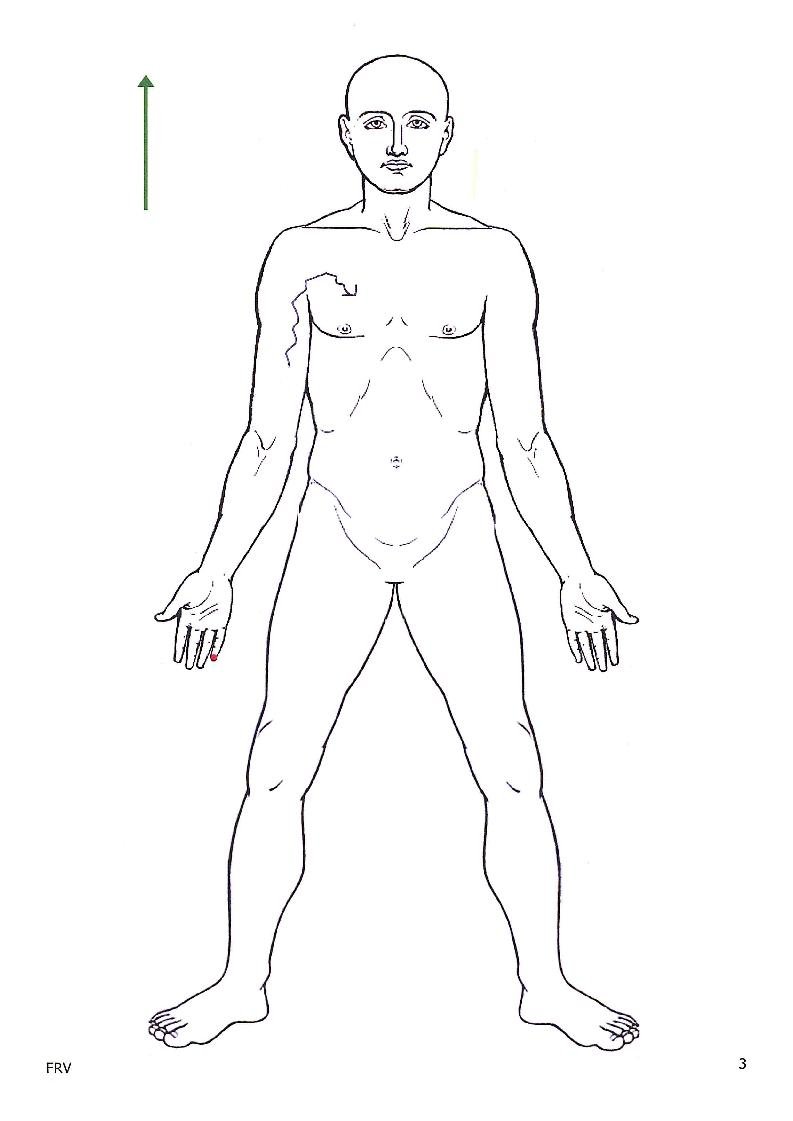

Supplement: S1 Raw Data — (ZIP) [file pone.0124808.s006.zip › Drawings - Imagined stimulation/finger_front/Subject_12_finger_front.jpg]

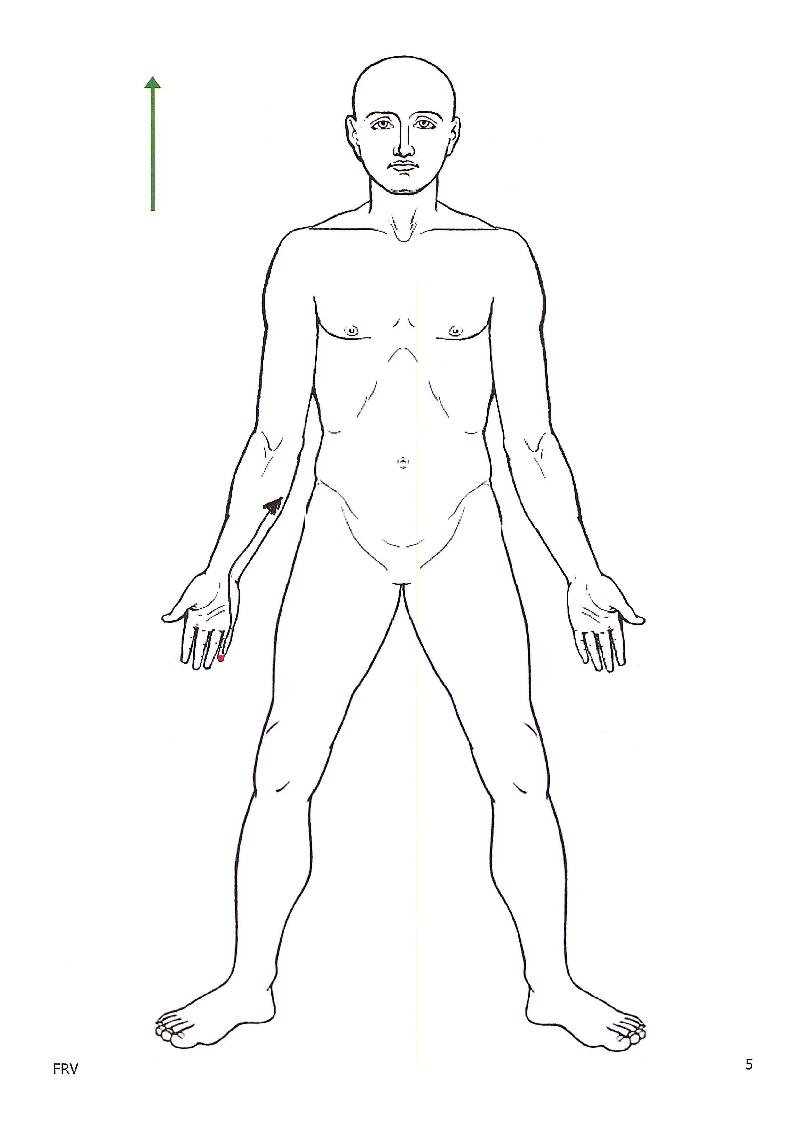

Supplement: S1 Raw Data — (ZIP) [file pone.0124808.s006.zip › Drawings - Imagined stimulation/finger_front/Subject_43_finger_front.jpg]

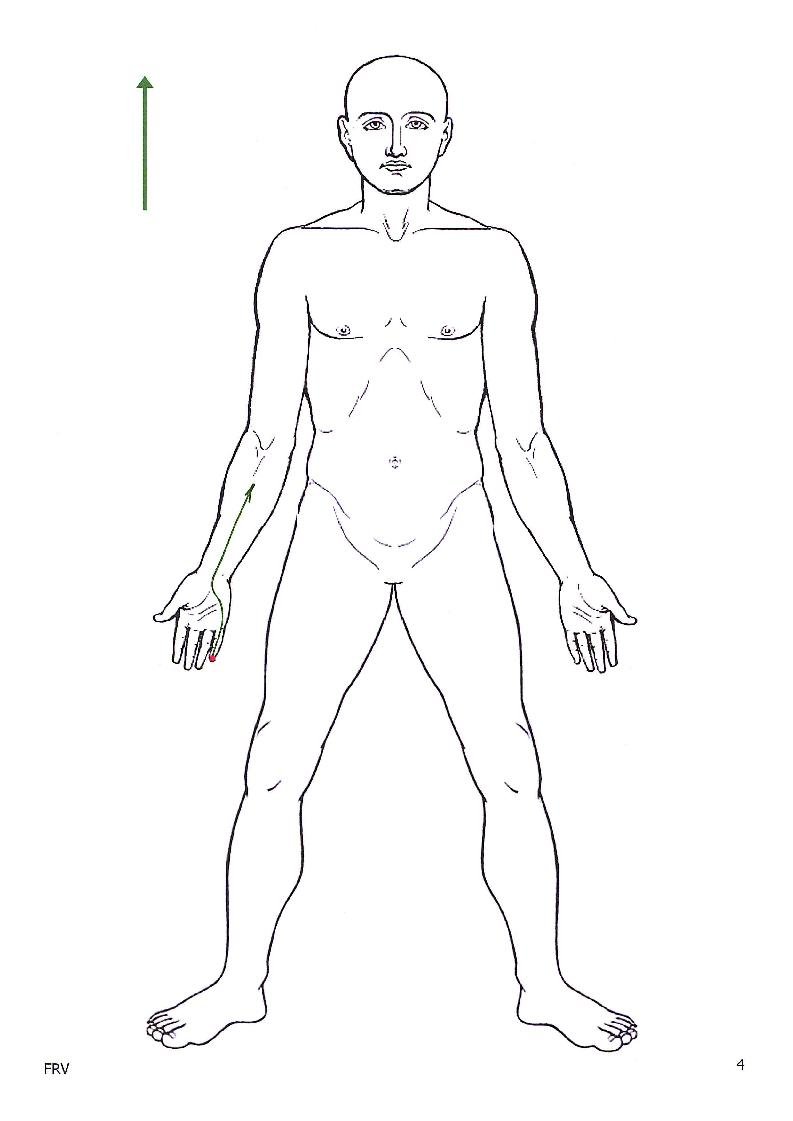

Supplement: S1 Raw Data — (ZIP) [file pone.0124808.s006.zip › Drawings - Imagined stimulation/finger_front/Subject_13_finger_front.jpg]
